# Photoredox-Catalyzed Enantioselective α-Deuteration of Azaarenes with D_2_O

**DOI:** 10.1016/j.isci.2019.06.007

**Published:** 2019-06-11

**Authors:** Tianju Shao, Yajuan Li, Nana Ma, Chunyang Li, Guobi Chai, Xiaowei Zhao, Baokun Qiao, Zhiyong Jiang

**Affiliations:** 1 Key Laboratory of Natural Medicine and Immuno-Engineering of Henan Province, Henan University, Kaifeng, Henan 475004, P. R. China; 2 Henan Key Laboratory of Organic Functional Molecules and Drug Innovation, School of Chemistry and Chemical Engineering, Henan Normal University, Xinxiang, Henan 453007, P. R. China; 3 Zhengzhou Tobacco Research of CNTC, Zhengzhou, Henan 450001, P. R. China

**Keywords:** Catalysis, Organic Chemistry, Chemical Synthesis

## Abstract

The site-specific incorporation of deuterium (D) into small molecules is frequently used to access isotopically labeled compounds with broad utility in many research areas, such as drug development, mechanistic studies, and NMR analyses. Nevertheless, the deuteration of a stereocenter in an enantioselective manner, which could slow the metabolism and improve the bioavailability of bioactive molecules, remains challenging owing to the lack of established catalytic methods. Here, we report an asymmetric α-deuteration strategy for azaarenes with inexpensive D_2_O as the deuterium source. A cooperative visible light-driven photoredox and chiral Brønsted acid–catalyzed system using a Hantzsch ester as the terminal reductant has been developed, which enables racemic α-chloro-azaarenes and prochiral azaarene-substituted ketones to experience a single-electron reduction–enantioselective deuteration process. The transition metal-free method provides important chiral α-deuterated azaarenes in satisfactory yields with good to excellent enantioselectivities (up to 99% ee) and substantial deuterium incorporation.

## Highlights

• Enantioselective deuteration enabled by photoredox asymmetric catalysis
• D_2_O as the deuterium source
• Azaarenes with a deuterated stereocenter
• Transition-metal-free catalyst system

## Graphical Abstract

**Figure:**
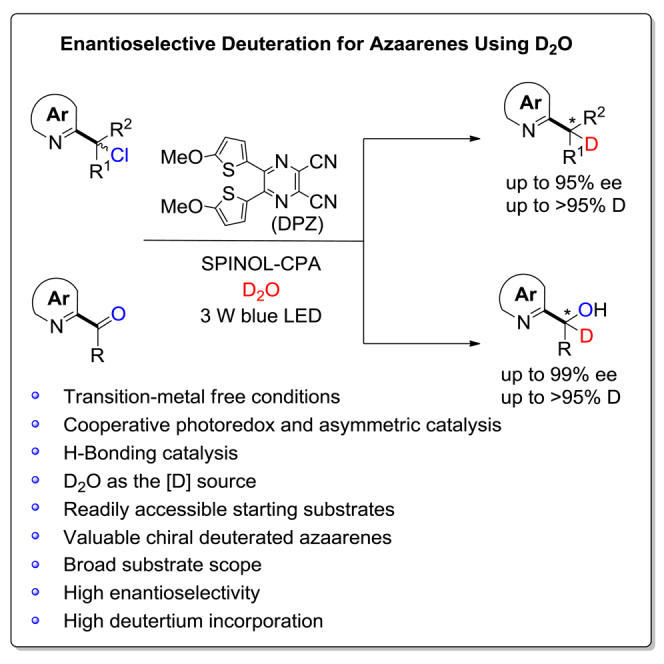

## Introduction

Deuterium (^2^H, D), an economical nonradioactive isotope, has been widely used in drug development, mechanistic studies, and NMR analyses (Elmore, 2009, Elmore and Bragg, 2015, Hall and Hanzlik, 1990, Nelson and Trager, 2003, MacDonald and Lu, 2002, Meanwell, 2011). A number of efficient deuterium-labeling techniques have been established through the increasing attention and contributions from various scientists (Alonso et al., 2002, Atzrodt et al., 2007, Loh et al., 2017, Zhang et al., 2019). The enantioselective incorporation of a deuterium atom on a stereocenter of a bioactive molecule could potentially slow its metabolism and improve its bioavailability (Maltais et al., 2009), but such processes remain underdeveloped (Hammadi et al., 1997, Lethu et al., 2018, Curran and Abraham, 1993, Curran and Ramamoorthy, 1993, Mohrig et al., 2011, Taglang et al., 2015, Hale and Szymczak, 2016, Palmer and Chirik, 2017, Zhao et al., 2012, Sakamoto et al., 2012, Sandoval et al., 2017). To date, few catalytic manifolds have been developed to accomplish this task (Taglang et al., 2015, Hale and Szymczak, 2016, Palmer and Chirik, 2017, Zhao et al., 2012, Sakamoto et al., 2012, Sandoval et al., 2017), and the existing systems of using the readily accessible racemic or prochiral feedstocks require prefabricated and expensive deuterium sources to attain excellent enantiofacial selectivity (Sakamoto et al., 2012, Sandoval et al., 2017). Azaarenes are ubiquitous in natural products and pharmaceuticals; however, to date, construction of their deuterated variants in enantioselective manner has not been reported.

Delivering a deuterium ion from inexpensive D_2_O to a prochiral tertiary carbanion in a chiral environment would be a promising strategy. Currently, H–D exchange reactions are among the main protocols for the preparation of deuterium-labeled compounds to meet the high demands (Atzrodt et al., 2007). The energy difference between de-protonation and de-deuteration that results in accumulation of deuterated species is subtle; therefore, achieving satisfactory enantioselectivity will be difficult using this strategy (Zhao et al., 2012). Recently, we reported the asymmetric construction of α-tertiary carbon stereocenters of electron-deficient azaarenes by developing a photoredox radical conjugate addition–enantioselective protonation reaction from vinylazaarenes (Yin et al., 2018). This study provided an important indication that single-electron reduction of the α-radical species is an irreversible strategy to generate α-carbanions of azaarenes. In photoredox catalysis (Yin et al., 2018, Prier et al., 2013, Shaw et al., 2016), the reductive dehalogenation of alkyl halides through a single-electron transfer (SET) fragmentation process enables the traceless formation of alkyl radicals (Hironaka et al., 1984, Staveness et al., 2016, Fukuzumi et al., 1990, Narayanam et al., 2009, Neumann et al., 2011, Tahara and Hisaeda, 2011, Maji et al., 2010, Li et al., 2018). Following our discovery, we explored the possibility of using this powerful strategy to generate α-radicals of azaarenes from α-halo-azaarenes. Notably, the ability of the subsequent process to cause protonation-like deuteration with D_2_O (Maji et al., 2010, Fan et al., 2018, Ju et al., 2018, Liao et al., 2018) but not hydrogen atom transfer (HAT) (Hironaka et al., 1984, Narayanam et al., 2009, Neumann et al., 2011) is crucial in the production of deuterated stereocenters. Meanwhile, to achieve high D incorporation, excess D_2_O might be utilized to compete with the H^+^ from the sacrificial reductant and reaction environment, but this excess reagent would pose a formidable challenge for chiral Brønsted acid catalysis, a promising platform for derivatizing azaarene-based substrates (Yin et al., 2018, Hepburn and Melchiorre, 2016, Proctor et al., 2018, Xu et al., 2018).

**Figure 1:**
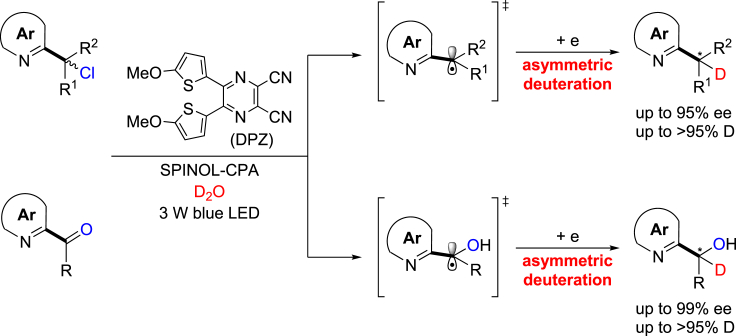
Outline of This Work

Herein, we demonstrate proof of this concept and report the first enantioselective reductive dechlorination–deuteration of α-chloro-azaarenes under the visible-light-driven cooperative photoredox asymmetric catalysis (Brimioulle et al., 2015, Skubi et al., 2016, Rono et al., 2013) of a dicyanopyrazine-derived chromophore (DPZ) as a photosensitizer and a 1,1′-spirobiindane-7,7′-diol (SPINOL)-based chiral phosphoric acid (CPA) with D_2_O as the deuterium source (Figure 1). A variety of valuable chiral α-deuterated azaarenes featuring various substituents on the stereocenters were obtained in high yields, with excellent D incorporation and with good to excellent enantioselectivities. More importantly, this catalytic system is very versatile showing compatibility with asymmetric reduction–deuteration of azaarene-substituted ketones, leading to important α-deuterated azaarene-based secondary alcohols with satisfactory results.

## Results and Discussion

### Reaction Optimization

Recognizing the paucity in viable catalytic methods to access the enantioenriched derivatives of α-aryl-α-branched alkyl-substituted quinolines, which possess significant bioactive potential (Kaila et al., 2008, Huang et al., 2010), we began our investigation using 2-(1-chloro-2-methyl-1-phenylpropyl)quinoline (**1a**) as the model substrate. The reduction potential of **1a** (*E*red 1/2 = − 0.639 V vs. SCE in CH_3_CN) indicated that our developed metal-free DPZ (*E*red 1/2 = −1.07 V vs SCE in CH_3_CN and −1.45 V vs SCE in CH_2_Cl_2_) will be able to reduce it through a reductive quenching cycle. Hence, we performed the initial study using 0.5 mol% DPZ, 1.5 equiv. of Hantzsch ester (HE) **HE-1** as the terminal reductant and 10 equiv. of D_2_O as the deuterium source. Desired α-deuterated quinoline **2a** was obtained in a 58% yield within 30 min (see entry 1, Table S1 in the Supplemental Information). The moderate D incorporation (53%) confirmed that the developed transformation might include a dechlorination-deuteration process. Subsequently, we evaluated a range of chiral H-bonding catalysts, reductants, and reaction parameters (see Tables S1, S2 and S3). The reaction performed in mesitylene at 25°C for 20 min in the presence of 1.0 mol% DPZ, 20 mol% SPINOL-CPA **C1**, 1.5 equiv. of **HE-1**, 1.0 equiv. of NaHCO_3_, and 150 equiv. of D_2_O afforded enantioenriched product **2a** in a 75% yield with 93% ee and >95% D incorporation (entry 1, Table 1). Catalysts **C2** and **C3** provided **2a** in 22% ee and 28% ee, respectively (entries 2–3), indicating that the substituents at the 6,6′-positions of SPINOL had a substantial influence on the enantioselectivity of the transformation. **HE-2**, with a *tert*-butyl ester, provided a slightly lower enantioselectivity (entry 4). When the reductant was changed from an HE to a tertiary amine, i.e., *i*Pr_2_EtN, the reaction became sluggish, leading to **2a** in 25% yield with 39% ee (entry 5). Other photoredox catalysts such as [Ir(ppy)_2_(dtbpy)]PF_6_ and 1,3-dicyano-2,4,5,6-tetrakis(*N*,*N*-diphenylamino)benzene (4DPAIPN) (Luo and Zhang, 2016) were tested, but no improvements in the yield and enantioselectivity were observed (entries 6–7). Using a stronger inorganic base, Na_2_CO_3_ instead of NaHCO_3_, as the acid-binding agent also deteriorated the reaction outcome (entry 8). Of note, the reaction conducted in the absence of catalyst **C1** generated **2a** in a similar yield and D incorporation, suggesting that a considerable competitive racemic background transformation is active (entry 9). The subsequent control experiments confirmed that DPZ, visible light, and the oxygen-free environment are indispensable to this deuteration reaction (entries 10–12).

**Table 1:** Optimization of the Reaction Conditions


Entry	Variation from Standard Conditions	Yield (%)^a^	ee (%)^b^	D Incorp. (%)^c^
1	None	75	93	>95
2	**C2** instead of **C1**	52	22	>95
3	**C3** instead of **C1**	69	28	>95
4	**HE-2** instead of **HE-1**	76	91	93
5	*i*Pr_2_EtN instead of **HE-1**	25	39	86
6	Ir(III)^d^ instead of DPZ	52	76	88
7	4DPAIPN instead of DPZ	43	73	90
8	Na_2_CO_3_ instead of NaHCO_3_	51	61	89
9	No **C1**	73	NA	>95
10	No DPZ	0	NA	NA
11	No light	0	NA	NA
12	Under air	0	NA	NA

NA, not available; 4DPAIPN, 1,3-dicyano-2,4,5,6-tetrakis(*N*,*N*-diphenylamino)benzene.

Also see Tables S1, S2, and S3.

The reaction was performed on a 0.05 mmol scale. The wavelength of the 3 W blue LED was 410–510 nm.

a Yield of isolated product.

b Determined by HPLC analysis on a chiral stationary phase.

c Determined by ^1^H NMR spectroscopy.

d Ir(III) = [Ir(ppy)_2_(dtbpy)]PF_6_.

### Substrate Scope with Respect to α-Chloro Azaarenes

**Figure 2:**
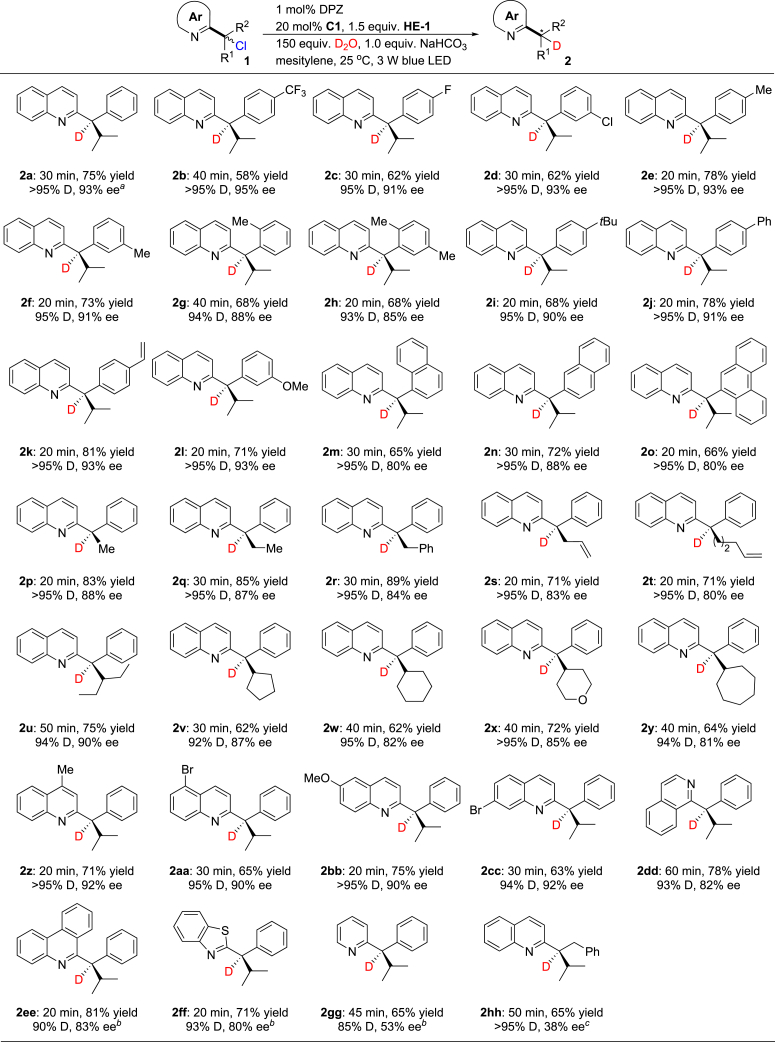
Enantioselective Dehalogenative Deuteration (0.1 mmol scale)

With the optimum reaction conditions in hand, the scope of this asymmetric α-deuteration of azaarenes was examined (Figure 2). A wide range of α-chloro-2-quinolines containing both aryl and alkyl groups on the sp^3^−C were evaluated. The transformations proceeded rapidly and smoothly, furnishing chiral products **2a**-**cc** in 58%–89% yields with 80%–95% ee and high levels of D incorporation within 20–40 min. The introduction of distinct electron-withdrawing or electron-donating substituents at the *para*- and *meta*-positions (**2b-f** and **2i-l**) of the α-aryl ring did not affect the excellent enantioselectivity. Methyl group at the ortho-position (**2g-h**) decreased the enantioselectivity slightly to 90% ee probably owing to steric hindrance. A similar steric effect was observed when bulkier fused aromatic rings, such as 1-naphthyl (**2m**), 2-naphthyl (**2n**), and 9-phenanthryl (**2o**), were in the α-position of the 2-quinolines. The catalytic system was also compatible with α-chloro-2-quinolines with diverse linear (**2p-t**), bulky branched (**2u**), and cyclic (**2v-y**) α-alkyl groups, based on the good to excellent enantioselectivities obtained with these substrates. With respect to the quinoline moiety, the installation of substituents on its 4-, 5-, and 6-positions resulted in the corresponding products **2z-cc** with excellent enantioselectivities. Other such α-chloro-azaarenes were also tested. Chiral α-deuterated 1-isoquinoline (**2dd**), 6-phenanthridine (**2ee**), and 2-benzothiazole (**2ff**) were obtained in 71%–81% yields with 80%–83% ee and excellent D incorporation, which highlights the generality of this catalysis platform. As an exception, when the azaarene was 2-pyridine, the corresponding product **2gg** was obtained with only moderate enantioselectivity. Noticeably, 4DPAIPN was used as the photoredox catalyst to furnish **2ee**, **2ff**, and **2gg** as DPZ could not promote the transformation. To define the substrate scope, the phenyl group of **1a** was changed as benzyl group; however, product **2hh** was achieved in 65% yield with >95% D incorporation but only 38% ee, which thus still represents a challenging task. The reaction to access **2a** on a 5.0 mmol scale gave a similar yield, enantioselectivity, and D incorporation (footnote *a*), suggesting the potential scalability of this method.

### Mechanistic Studies

**Figure 3:**
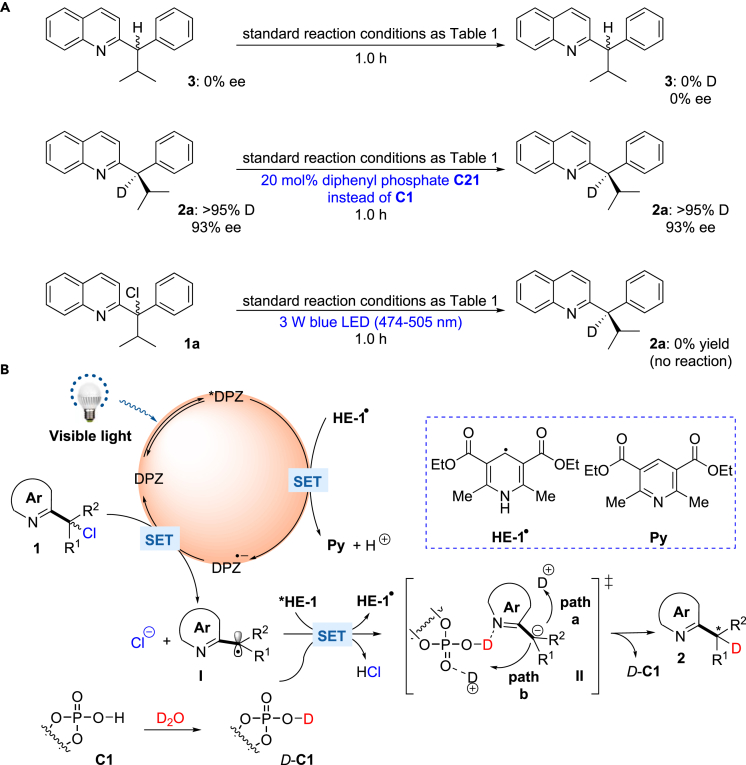
Control Experiments and the Proposed Mechanism (A) Control experiments for the H–D exchange of reduction product and the conversion of **1a** with an excitation wavelength from 474 to 505 nm. (B) The possible mechanism for the photoredox-catalyzed enantioselective α-deuteration of Azaarenes.

To probe the mechanism, compound **3**, i.e., non-deuterated racemic **2a**, was evaluated under the standard reaction conditions (Figure 3A). After 1 h, the ee value of **3** remained 0% and no deuterated product **2a** was observed. Similarly, chiral **2a** with 93% ee was examined using 20 mol% diphenyl phosphate **C21** instead of **C1**, and no changes in the enantiomeric purity or D incorporation were found. Consequently, the effects of H–D exchange for product **2** on the enantioselectivity and D incorporation could be excluded.

**Figure 4:**
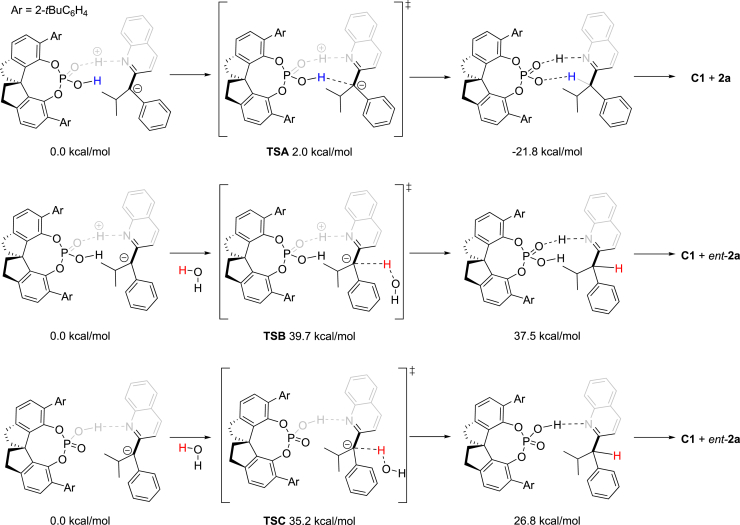
Calculated Δ*G* Values of Possible Pathways for the Formation of 2a or *ent*-2a

On the account of the excitation spectra of DPZ and **HE-1**, Stern-Volmer experiments were first conducted using an excitation wavelength of 415 nm (see Supplemental Information). Luminescence quenching of *DPZ (*E*^t^(S*/S^⋅−^) = +1.42 V vs. SCE in CH_3_CN and +0.91 V vs. SCE in CH_2_Cl_2_) by **HE-1** (*E*red 1/2 = +0.97 V vs. SCE in CH_3_CN) and *HE-1 (*E*^t^(S*/S^⋅−^) ≈ –2.28 V vs. SCE in CH_3_CN) (Jung et al., 2016) by **1a** (*E*red 1/2 = –0.639 V vs. SCE in CH_3_CN) was observed. Meanwhile, no measurement of *DPZ by **1a** in the absence and presence of **C1** was revealed. Since the excitation of **HE-1** to *HE-1 is impossible at λ > 445 nm according to its excitation spectrum, such experiments were performed with an excitation wavelength of 448 nm, indicating no measurable luminescence quenching of *DPZ by **HE-1** or **1a** and *HE-1 by **1a**. We also tested the transformation of **1a** with a laser line filter (CWL = 488 ± 2 nm, FWHM = 10 ± 2 nm, emission wavelengths from 474 to 505 nm, Figure 3A), and no reaction was found. The results suggest the indispensable role of *HE-1 in the reaction. Therefore, the catalytic cycle likely begins from the SET of *DPZ with trace **HE-1**^**⋅**^ (*E*red 1/2 ≈ –0.76 V vs. SCE), which is generated from *HE-1 and α-chloro-azaarenes **1** under irradiation with visible light (Figure 3B) (Cao et al., 2019). The success of 4DPAIPN (*E*red 1/2 = –1.52 V vs. SCE in CH_3_CN) (Luo and Zhang, 2016) but failure of DPZ in the transformations of 6-phenanthridine-derived (**1ee**, *E*red 1/2 = –0.963 V vs. SCE in CH_3_CN) and 2-benzothiazole-derived (**1ff**, *E*red 1/2 = –1.227 V vs. SCE in CH_3_CN) substrates demonstrates that DPZ^⋅−^ should engage in the SET reduction of **1** to furnish radical species **I**. It is also noteworthy that no transformation of **1a** was observed in the absence of DPZ (entry 10, Table 1). Accordingly, a HAT (Lee et al., 2017) or a reduction–protonation/deuteration process between **HE-1**^**⋅**^ and radical **I** seems unfavorable. To provide **HE-1**^**⋅**^ to propagate the photocatalytic cycle, the reduction of **I** by *HE-1 with a stronger reductive ability than **HE-1**^**⋅**^ thus represents the most likely approach. Owing to the free proton from catalyst **C1** and high concentration of D_2_O, deuterated **C1**, i.e., *D*-**C1**, would serve as a more stable hydrogen-bond donor to interact with the nitrogen atom in the azaarene, providing the enantiocontrolling environment for the deuteration of prochiral carbanion intermediate **II** to give chiral products **2** in high enantioselectivities. With respect to the enantioselective deuteration process, the intermediate **II** could directly abstract deuterium ion from the reaction system through the **pathway a**. However, the ability of P=O of CPA as Lewis base to grab a deuterium also leads to an alternative **pathway b** to deliver deuterium ion. To better understand the mechanism, DFT calculations were carried out (for computational details see Supplemental Information), and the calculated Δ*G* values are shown in Figure 4. The data suggest that the formation of (*R*)-**2a** through **TSA** only requires 2.0 kcal/mol, but affording (*S*)-**2a** through the proton from H_2_O (D_2_O) attacked by the α-anion of quinoline has to overcome high energy barriers (39.7 and 35.2 kcal/mol). This result is consistent with the experimental results. In this context, although chiral catalyst CPA cannot accelerate the transformations, it will act as a deuterium transfer interchange to enable the elusive enantioselective manifold with high enantioselectivity.

### Extension of the Method to Azaarene-Based Ketones

**Figure 5:**
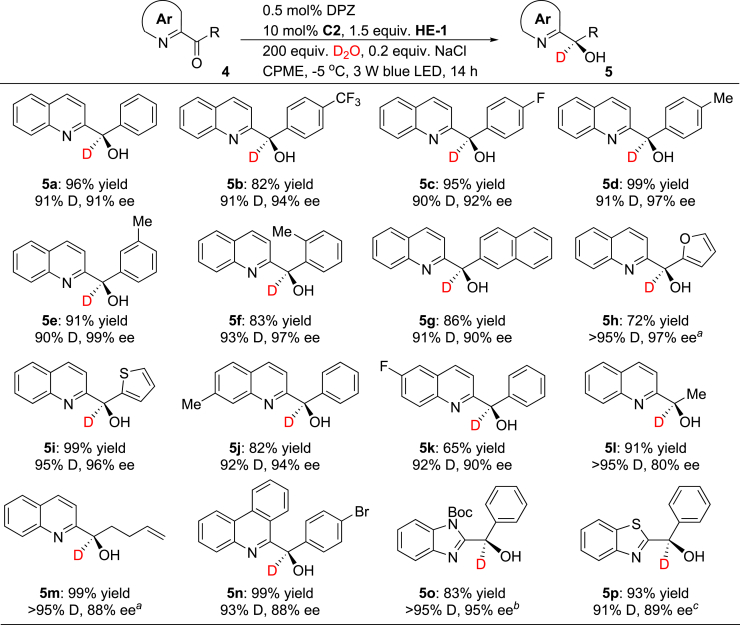
Enantioselective Reductive Deuteration (0.1 mmol scale)

Azaarene-substituted secondary alcohols, as another kind of α-tertiary carbon-based azaarene derivative, are also important building blocks of numerous pharmaceuticals and biologically active compounds (Rennison et al., 2007). The chiral transition metal-catalyzed asymmetric transfer hydrogenation of aryl *N*-heteroaryl ketones with sodium formate as a hydrogen source has been developed to access the chiral variants (Liu et al., 2018). However, this strategy is only suitable for aryl *N*-heteroaryl ketones and is likely not compatible to introduce deuterium on the stereocenters with D_2_O as the deuterium reagent. Given that azaarene-substituted ketones are readily accessible and abundant, we anticipated evaluating the possibility of this double single-electron reduction–enantioselective deuteration method for these feedstocks, to directly provide the enantioenriched α-deuterated azaarene-substituted secondary alcohols. Literature investigation showed that azaarene rings are more liable to reduction than carbonyls in azaarene-substituted ketone derivatives in the presence of CPA catalyst and HE reductant (Rueping and Antonchick, 2007). Thus, the chemoselectivity with enantioselectivity and D incorporation would constitute formidable challenges in the desired transformations. Phenyl(quinolin-2-yl)methanone (**4a**, *E*_p_^1^ = –0.905 V, *E*_p_^2^ = –1.434 V vs. SCE in CH_3_CN) was first selected to be examined under the standard reaction conditions (see Table 1). Although the desired product **5a** was obtained with moderate enantioselectivity (61% ee), the high yield (80%) and moderate D incorporation (72%) prompted us to explore a series of chiral CPAs and the reaction parameters (see Table S4). To our delight, when 0.5 mol% DPZ, 10 mol% CPA **C2**, 1.5 equiv. of **HE-1**, 200 equiv. of D_2_O, 0.2 equiv. of NaCl as an additive, and CMPE as the solvent were used, at −5°C, **5a** was achieved in 96% yield with 91% ee and 91% D incorporation (Figure 5). A series of 2-quinoline-substituted ketones were next examined, leading to chiral products **5b-m** in 65%–99% yields with 80%–99% ee and excellent D incorporation. Satisfactory results were achieved with fused aromatic (**5g**) and heteroaromatic (**5h-i**) rings as well as alkyls (**5l-m**) as the substituents of ketones underscore the generality of this catalytic system. Furthermore, ketones substituted with other azaarenes such as 6-phenanthridine (**5n**), *N*-Boc-2-benzimidazole (**5o**), and 2-benzothiazole (**5p**) were also compatible. It is worth mentioning that, although 2-(α-hydroxybenzyl)-benzimidazole (HBB) is a highly selective and potent inhibitor of picornavirus multiplication in cell culture, no catalytic method to access its optical pure isomer has been established (Tamm and Eggers, 1963, Kadin et al., 1964, Akihama et al., 1968, Tamm et al., 1969). The success in the synthesis of its chiral variant featuring a deuterium on the stereocenter, i.e., **5o**, highlights the significance of this methodology.

### Conclusion

In summary, we have developed the first catalytic asymmetric α-deuteration of azaarenes. Through cooperative, visible-light-driven photoredox and chiral H-bonding catalysis, both racemic α-chloro-azaarenes and prochiral azaarene-substituted ketones can undergo a double single-electron reduction–enantioselective deuteration process with a Hantzsch ester as the terminal reductant and inexpensive D_2_O as the deuterium source. The developed reaction can furnish a variety of important enantioenriched α-deuterated azaarenes in satisfactory yields with high D incorporation. Although excess D_2_O is used and a strong racemic background process occurs directly through the intrinsic redox catalytic cycle, good to excellent enantioselectivities were obtained, which confirms the efficacy of the catalytic system. Therefore, we anticipate that this work will inspire the pursuit of the D_2_O-enabled asymmetric construction of C–D bonds in various valuable molecules through this highly reactive radical-based dual catalysis platform.

### Limitations of the Study

In both reaction systems, pyridine-based substrates were not applicable given the unsatisfactory enantioselectivity. With respect to the dehalogenative deuteration, poor enantioselectivity of mono-azaarene-substituted alkyl chlorides represents another important limitation.

## Methods

All methods can be found in the accompanying Transparent Methods supplemental file.

## Data and Software Availability

The crystallography data have been deposited at the Cambridge Crystallographic Data Center (CCDC) under accession number CCDC: 1893142 (**8**) and 1902192 (**5n**) and can be obtained free of charge from www.ccdc.cam.ac.uk/getstructures.

## Supplemental Information

Document S1. Transparent Methods and Figures S1–S185

Table S1. Evaluate the Effect of BA, Amine, Base, and the Solvent on the Reductive Dechlorination–Deuteration of 1a, Related to Table 1

Table S2. Evaluate the Effect of PC and [D] Source on the Reductive Dechlorination–Deuteration of 1a, Related to Table 1

Table S3. Evaluate the Effect of Amine and Time on the Enantioselective Reduction of 1a, Related to Table 1

Table S4. Optimization of Asymmetric Reduction–Deuteration of 4a, Related to Figure 5

Table S5. Cartesian Coordinates for the Formation of (*R*)-2a Proceeding Transferred Proton of P=O–H of CPA via TSA Process, Related to Figures 3 and 4

Table S6. Cartesian Coordinates for the Formation of (*ent*)-2a Proceeding Transferred Proton of P–O–H of CPA via TSB Process, Related to Figures 3 and 4

Table S7. Cartesian Coordinates for the Formation of (*ent*)-2a Proceeding Transferred Proton of H2O via TSC Process, Related to Figures 3 and 4

Data S1. Crystal Data and Structure Refinement for 8, Related to Figure 2

Data S2. Crystal Data and Structure Refinement for 5n, Related to Figure 5
