# Supplementary material for: Photoredox-Catalyzed Enantioselective α-Deuteration of Azaarenes with D2O
Source: iScience. 2019 Jun 11;16:410–9. doi: 10.1016/j.isci.2019.06.007 (PMC6593145; doi:10.1016/j.isci.2019.06.007)
Supplement: Document S1. Transparent Methods and Figures S1–S185 [file mmc1.pdf]

ISCI, Volume *16*

Supplemental Information

Photoredox-Catalyzed Enantioselective α-Deuteration of Azaarenes with D_2O

Tianju Shao, Yajuan Li, Nana Ma, Chunyang Li, Guobi Chai, Xiaowei Zhao, Baokun Qiao, and Zhiyong Jiang

Supplemental Figures for ^{1}H and ^{13}C NMR Spectra and HPLC Spectra

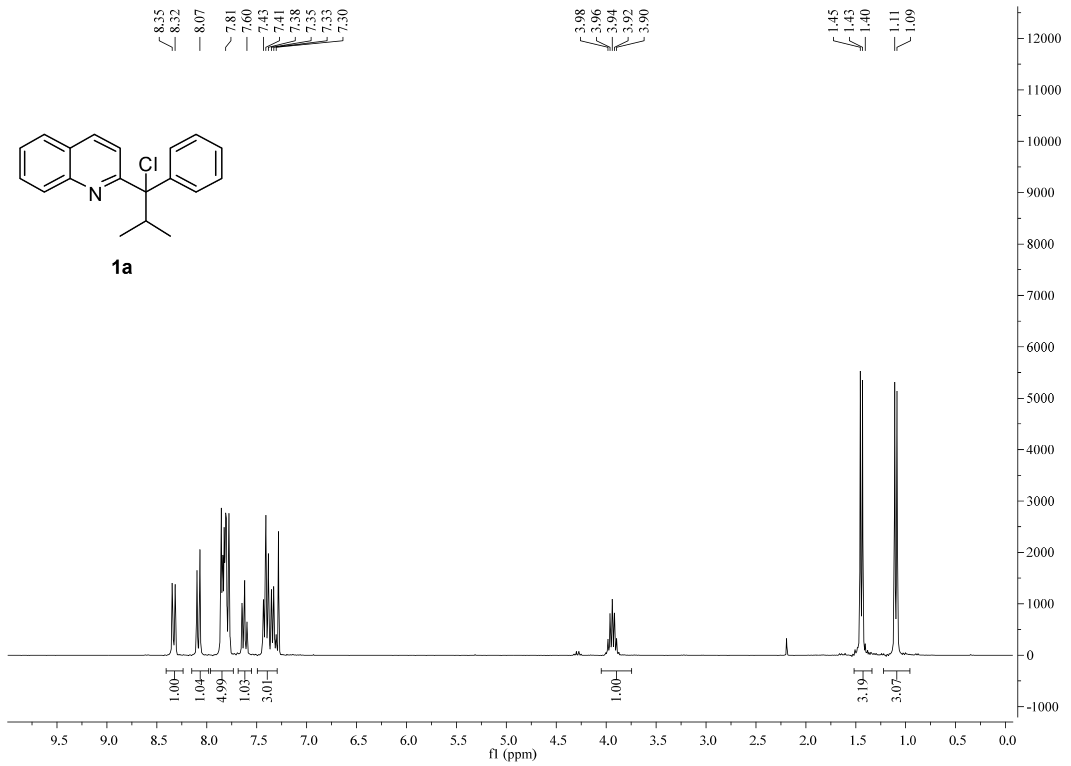

Figure S1. ^{1}H NMR spectrum for **1a**, related to **Figure 2**.

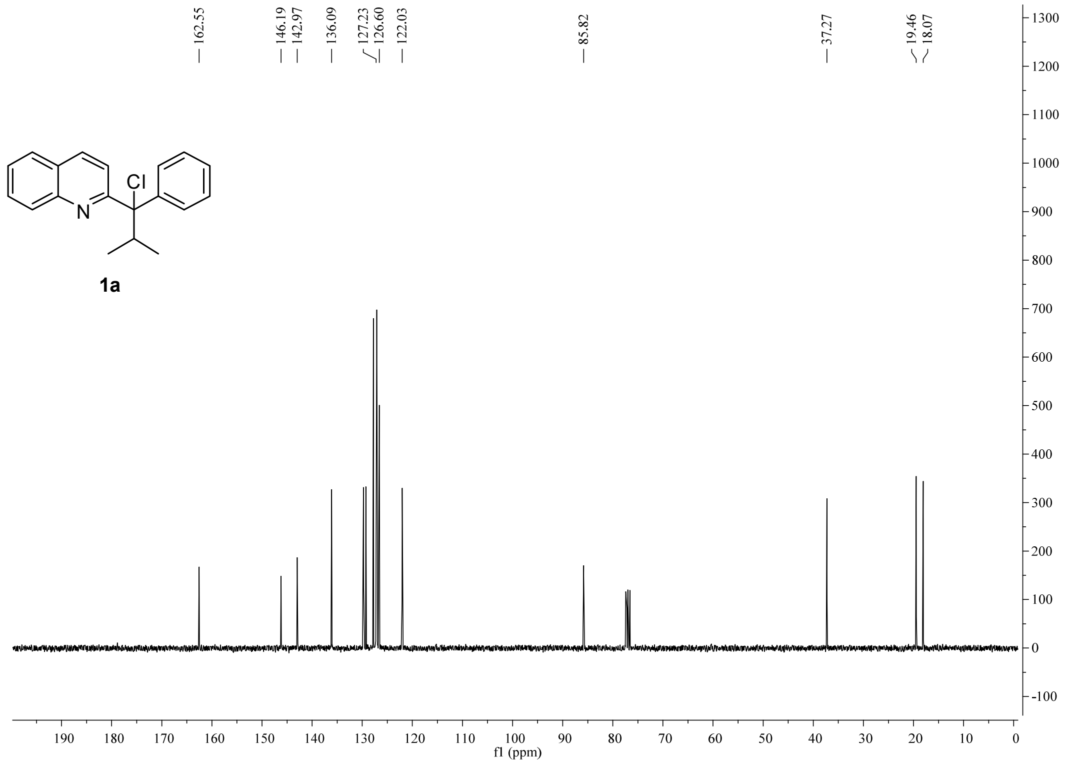

Figure S2. ^{13}C NMR spectrum for **1a**, related to **Figure 2**.

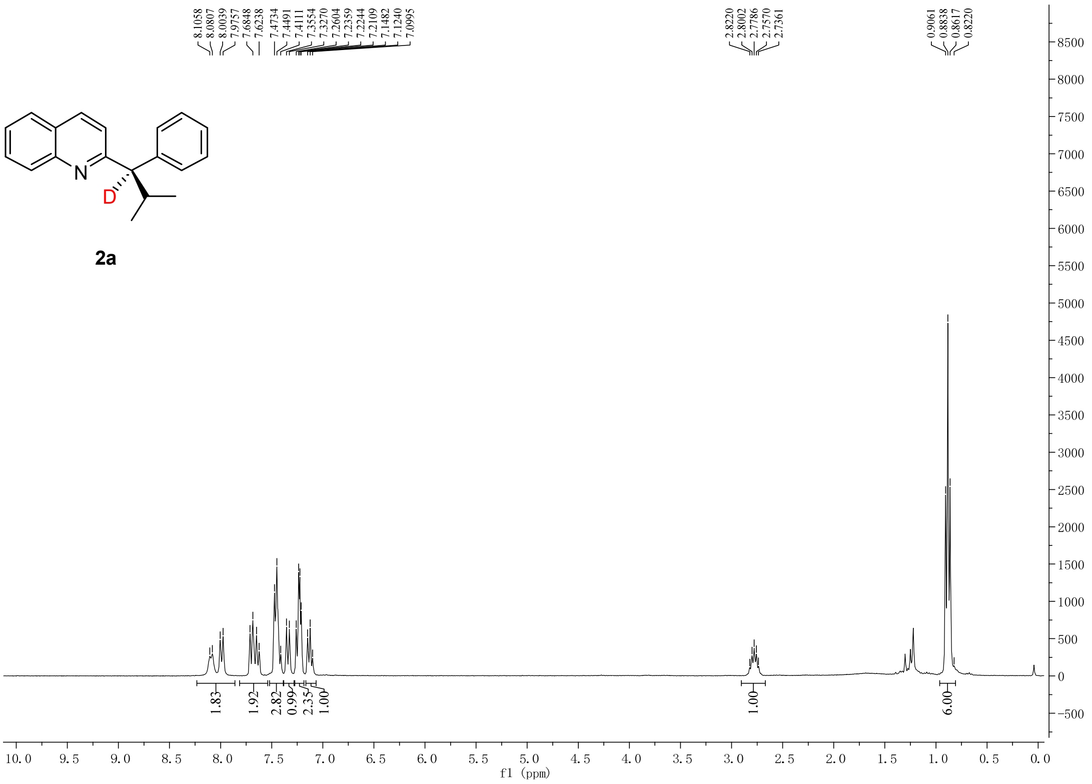

Figure S3. ^{1}H NMR spectrum for **2a**, related to **Figure 2**.

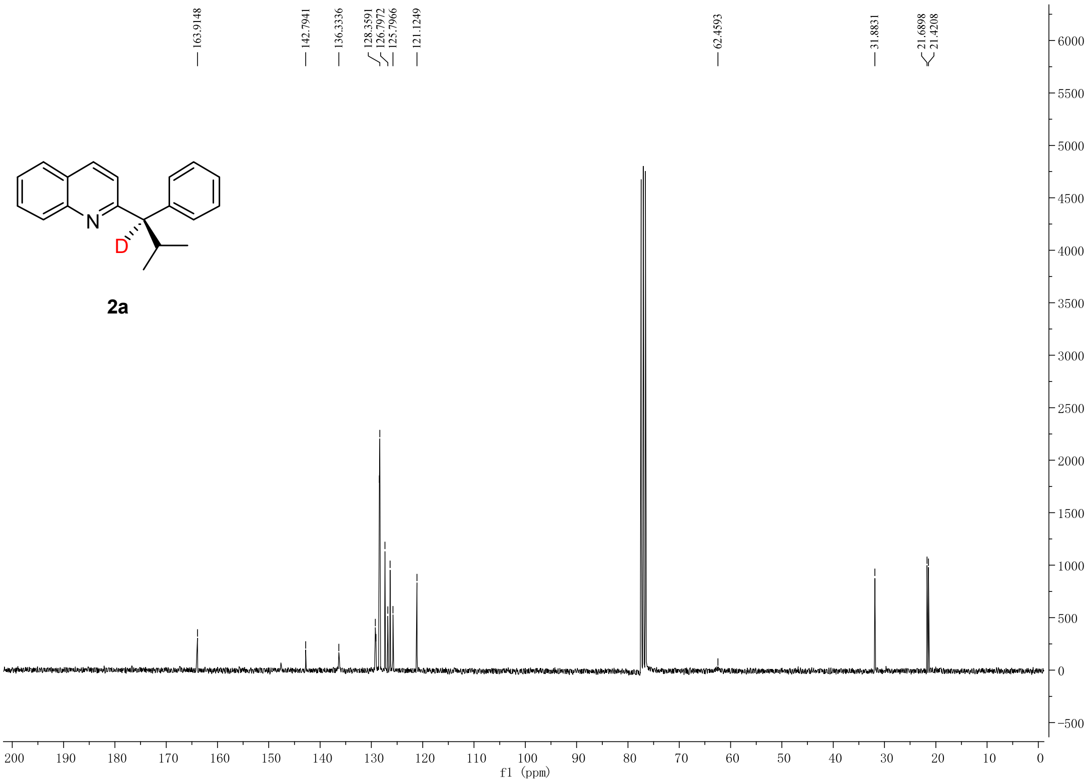

Figure S4. ^{13}C NMR spectrum for **2a**, related to **Figure 2**.

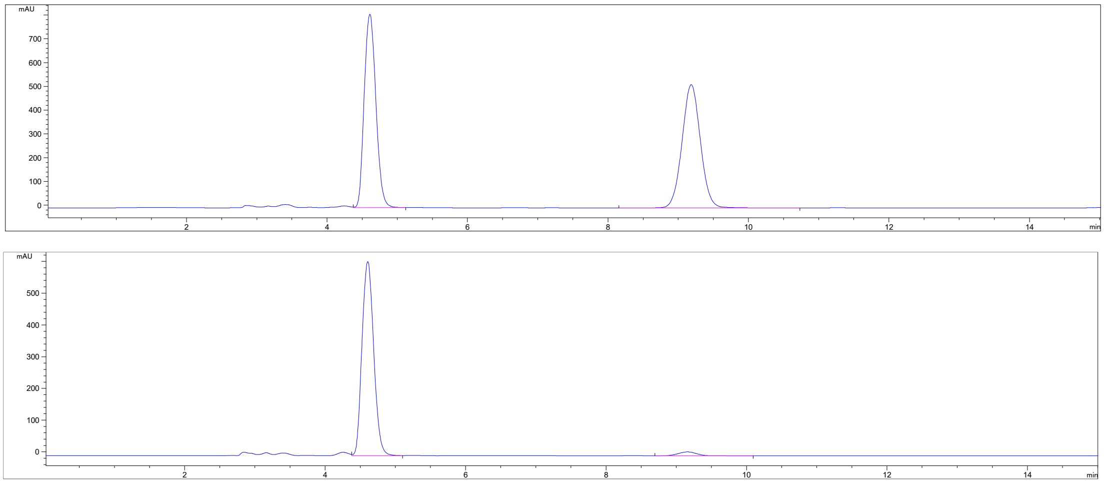

Figure S5. HPLC spectrum for **2a**, related to **Figure 2**.

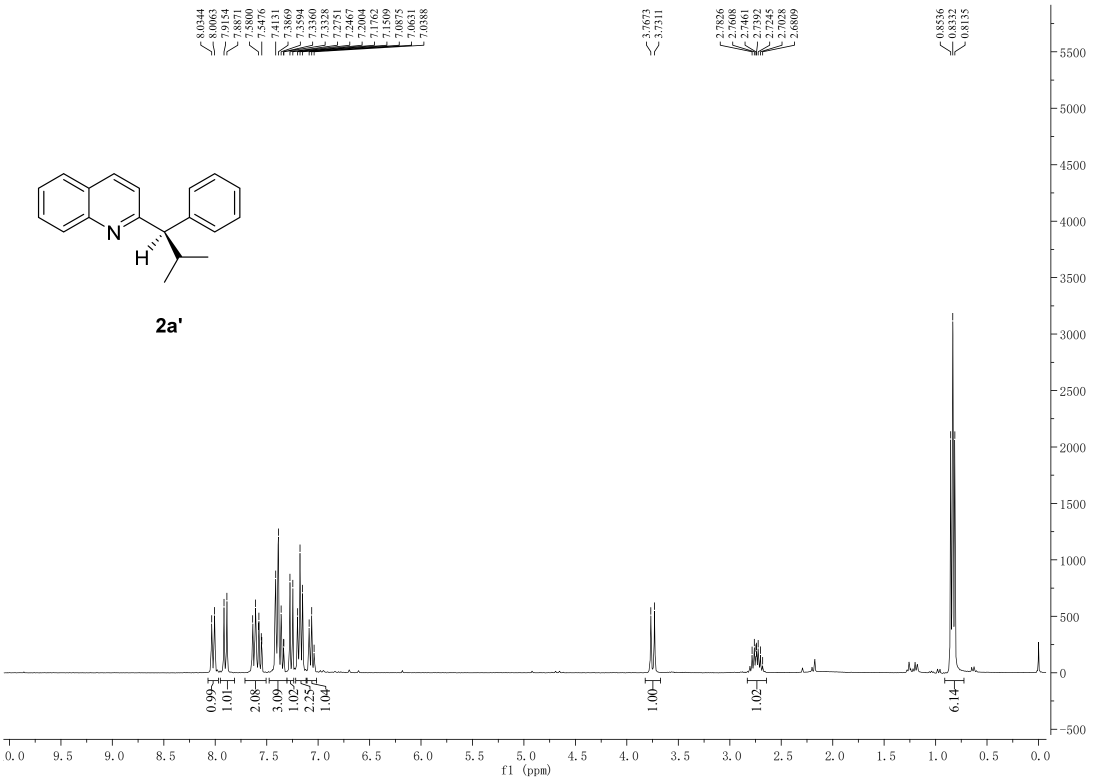

Figure S6. ^{1}H NMR spectrum for **2a'**, related to **Figure 2**.

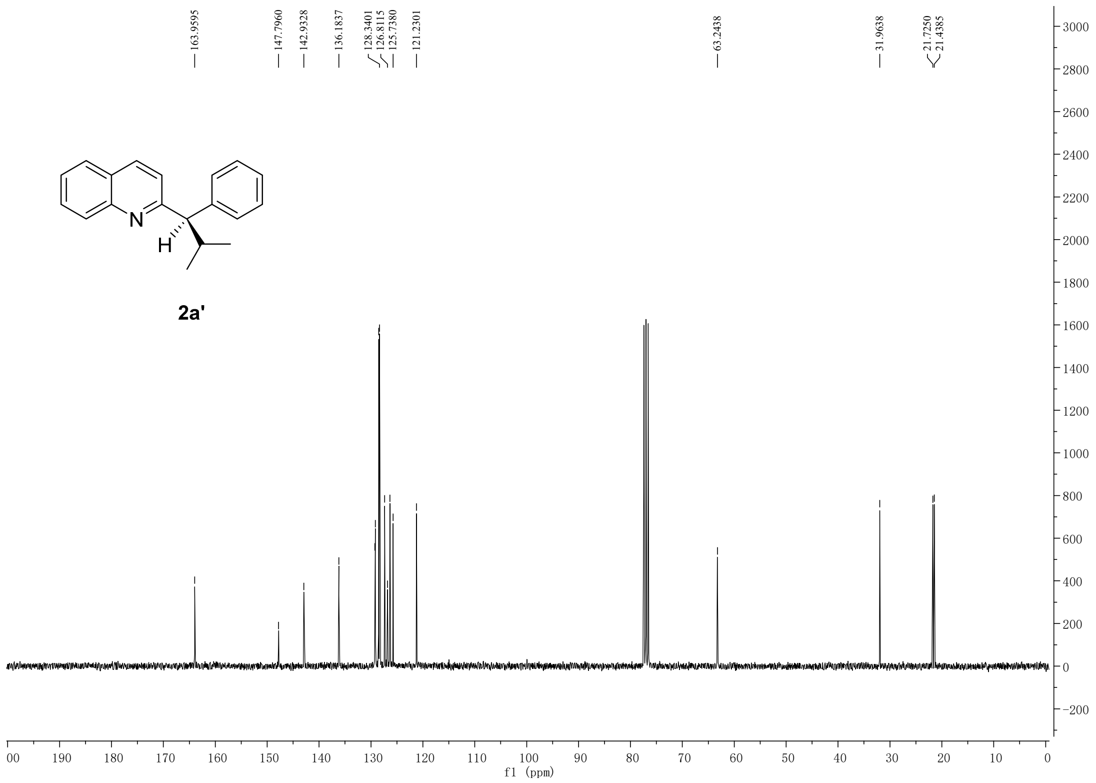

Figure S7. ^{13}C NMR spectrum for **2a’**, related to **Figure 2**.

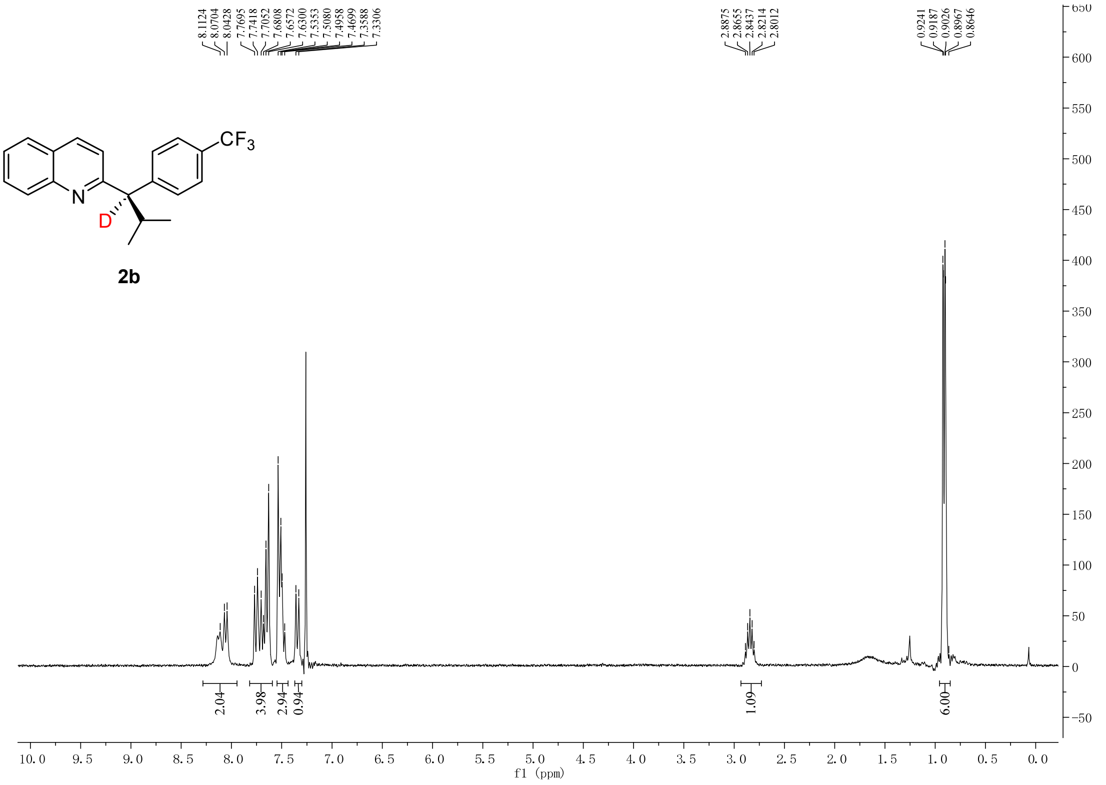

Figure S8. ^{1}H NMR spectrum for **2b**, related to **Figure 2**.

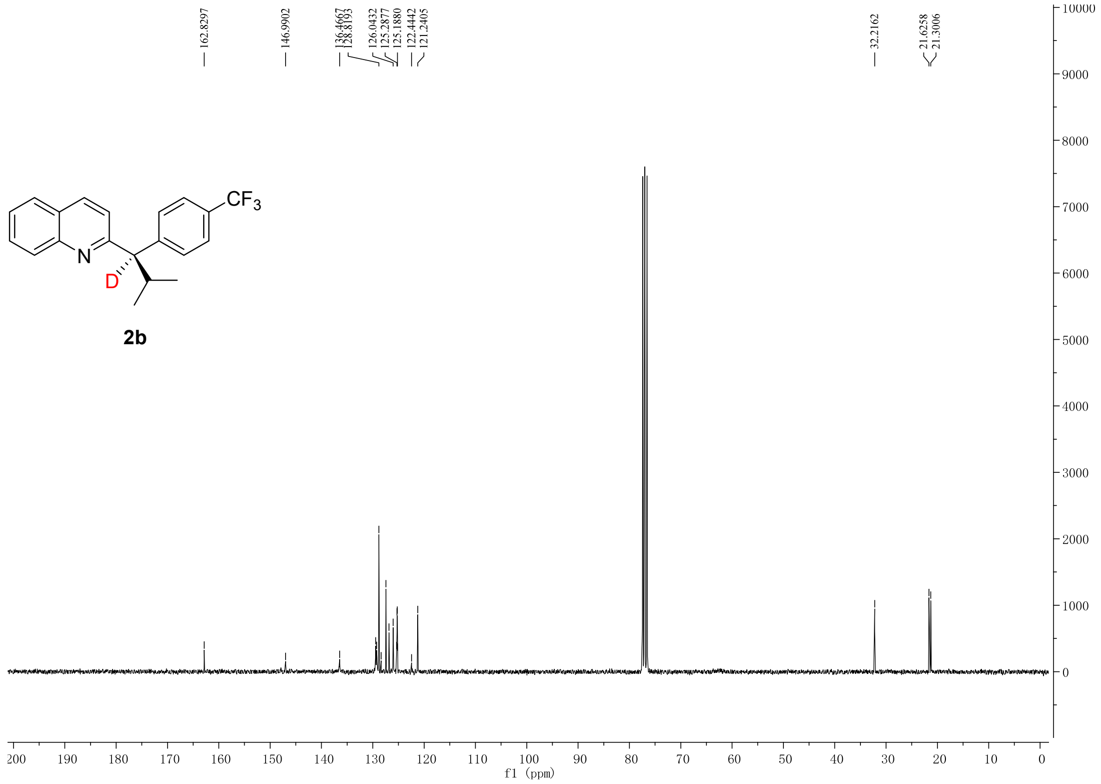

Figure S9. ^{13}C NMR spectrum for **2b**, related to **Figure 2**.

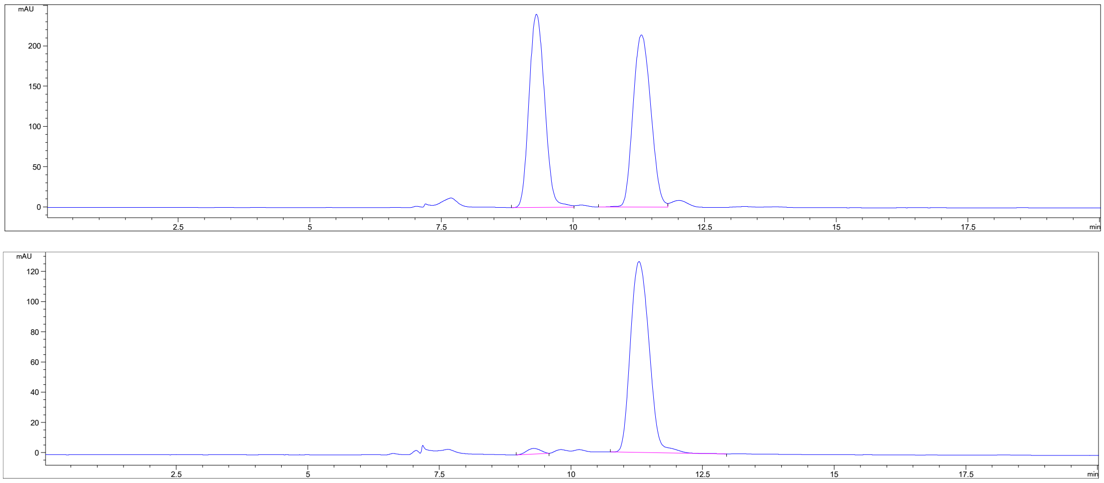

Figure S10. HPLC spectrum for **2b**, related to **Figure 2**.

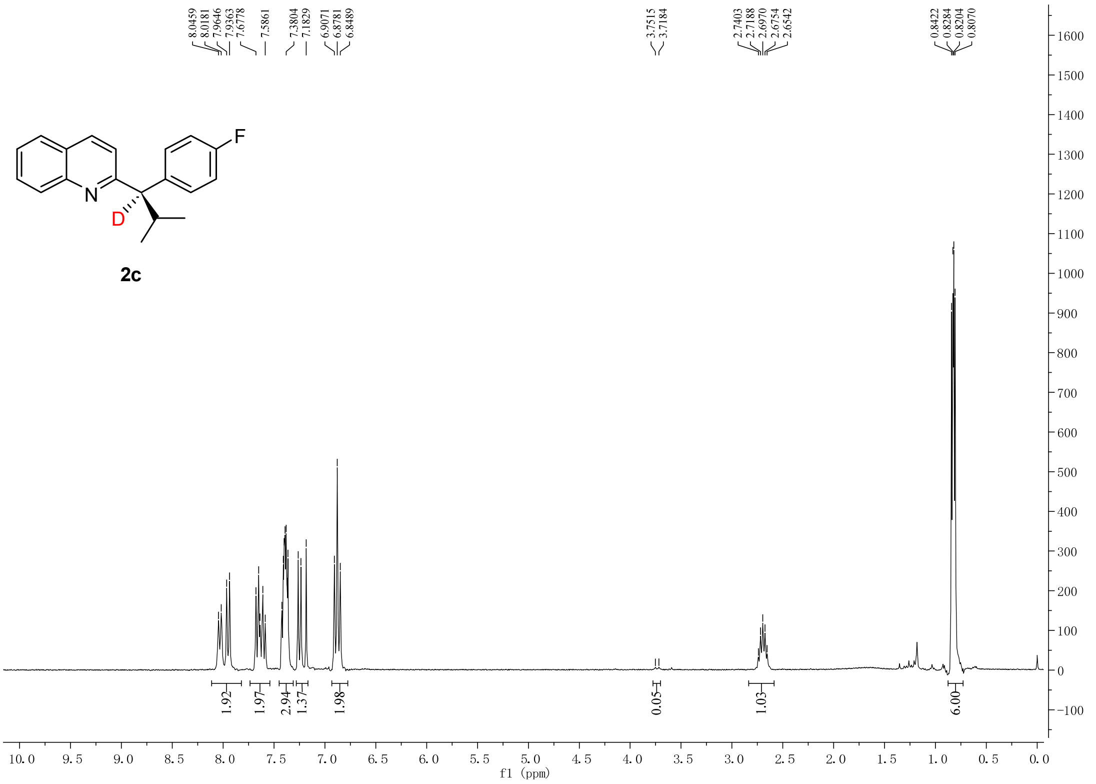

Figure S11. ^{1}H NMR spectrum for **2c**, related to **Figure 2**.

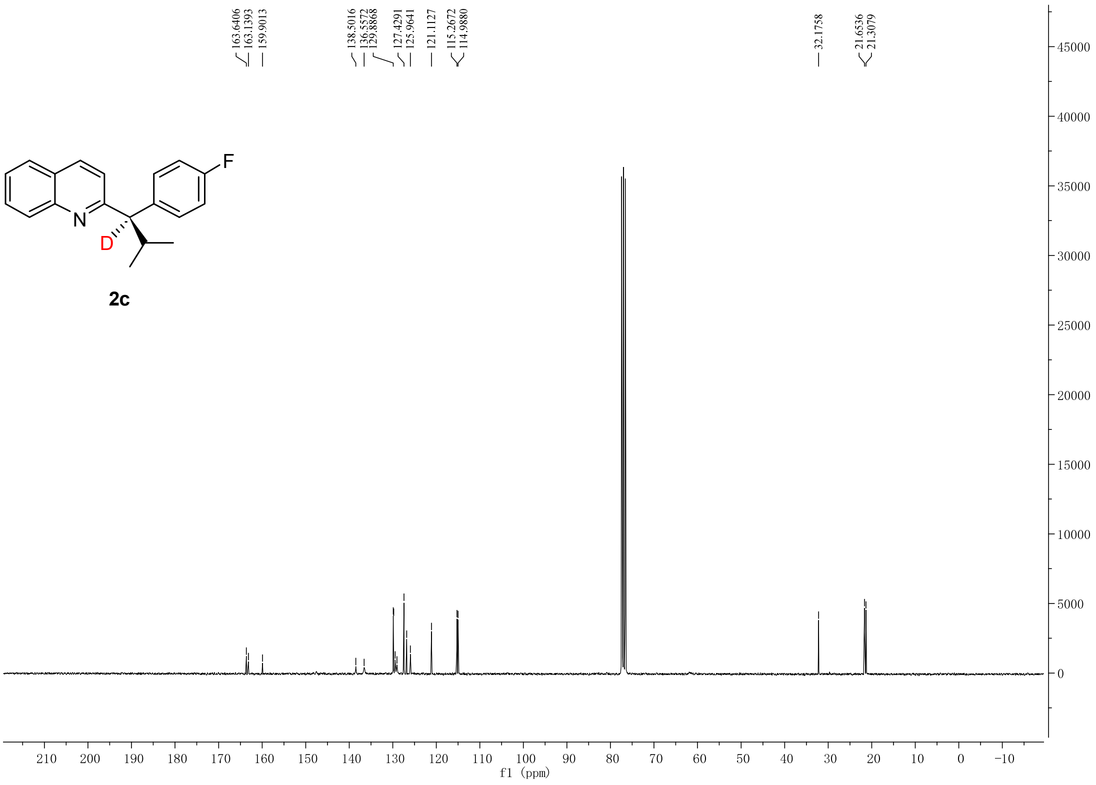

Figure S12. ^{13}C NMR spectrum for **2c**, related to **Figure 2**.

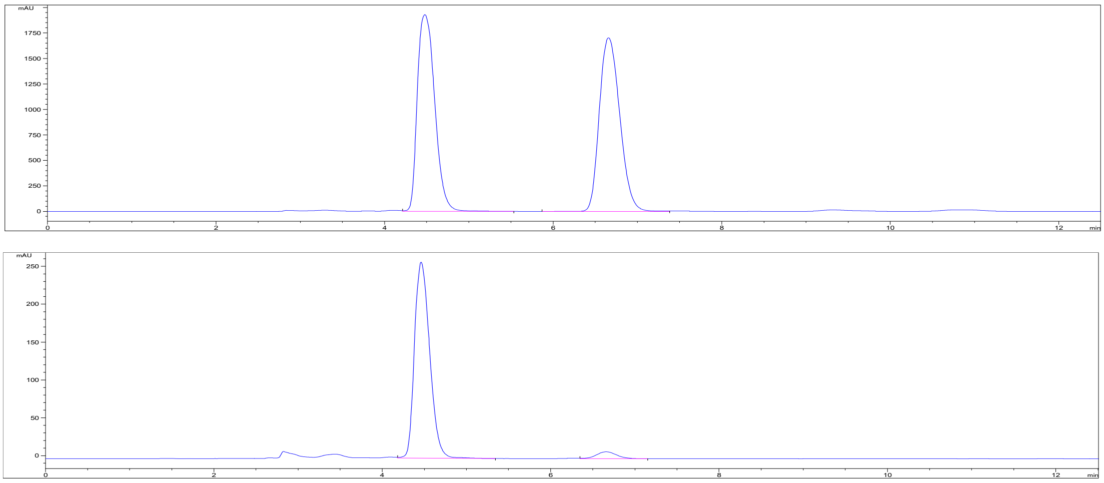

Figure S13. HPLC spectrum for **2c**, related to **Figure 2**.

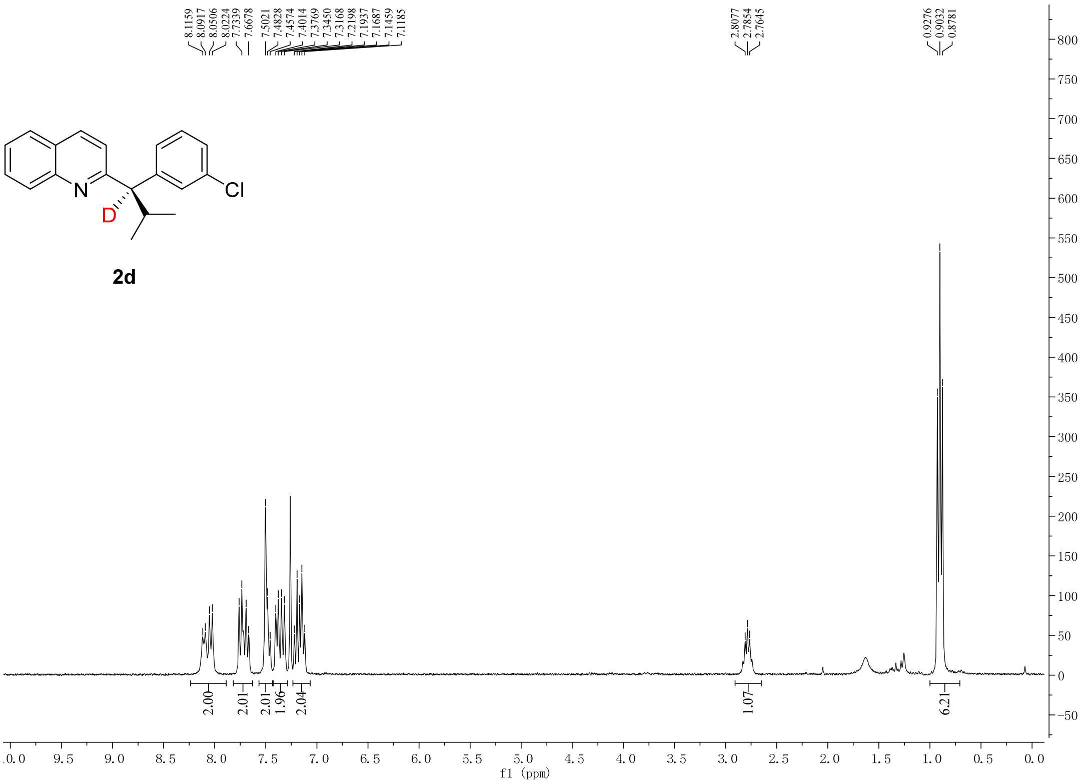

Figure S14. ^{1}H NMR spectrum for **2d**, related to **Figure 2**.

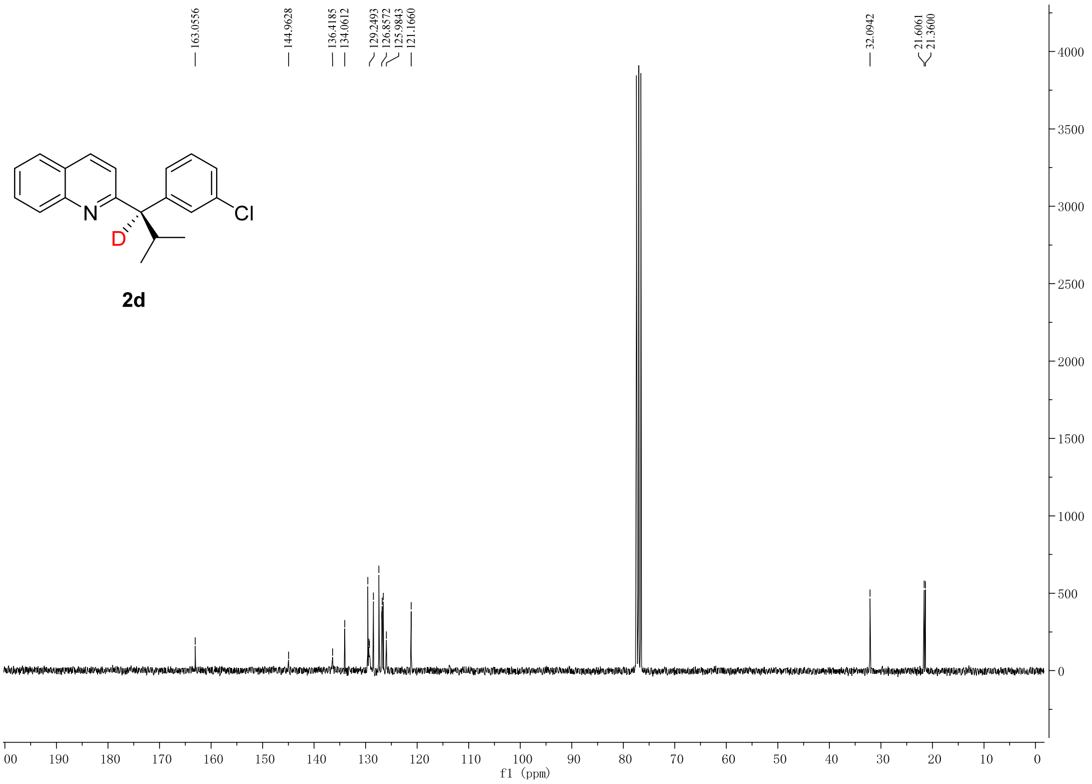

Figure S15. ^{13}C NMR spectrum for **2d**, related to **Figure 2**.

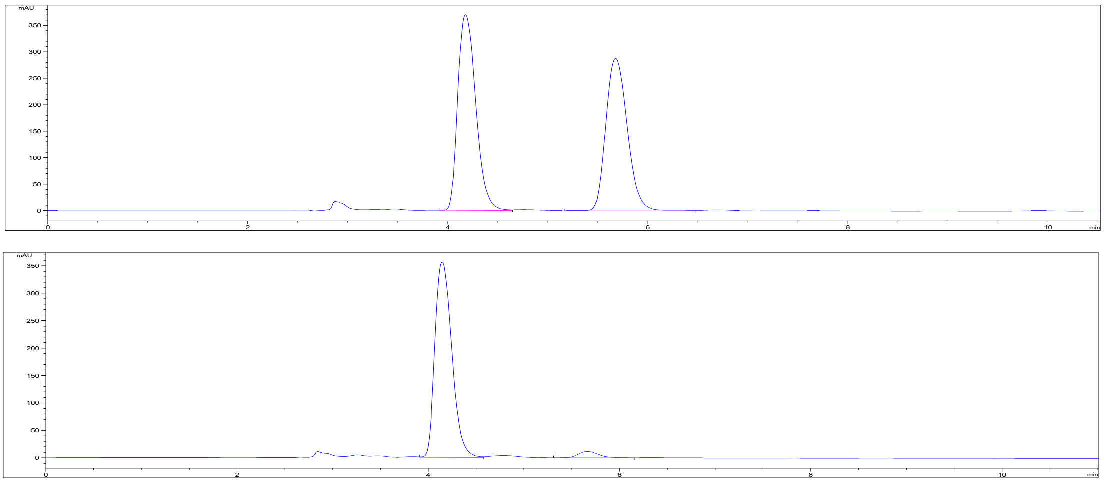

Figure S16. HPLC spectrum for **2d**, related to **Figure 2**.

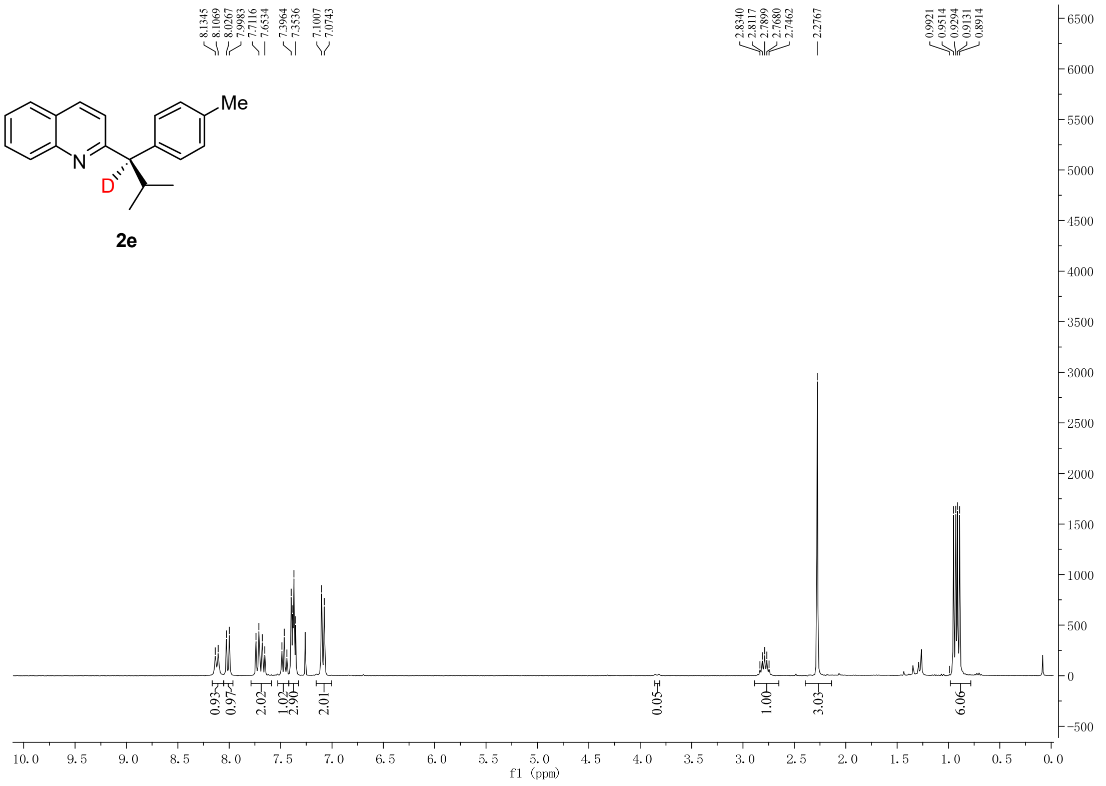

Figure S17. ^{1}H NMR spectrum for **2e**, related to **Figure 2**.

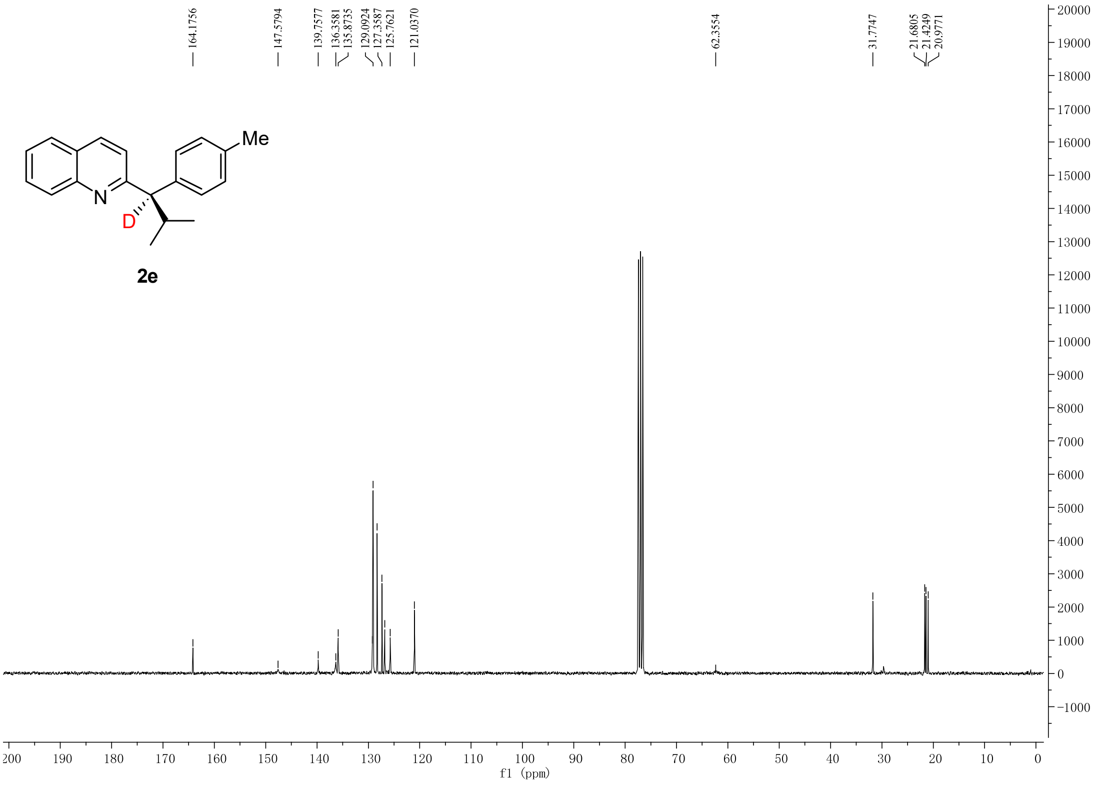

Figure S18. ^{13}C NMR spectrum for **2e**, related to **Figure 2**.

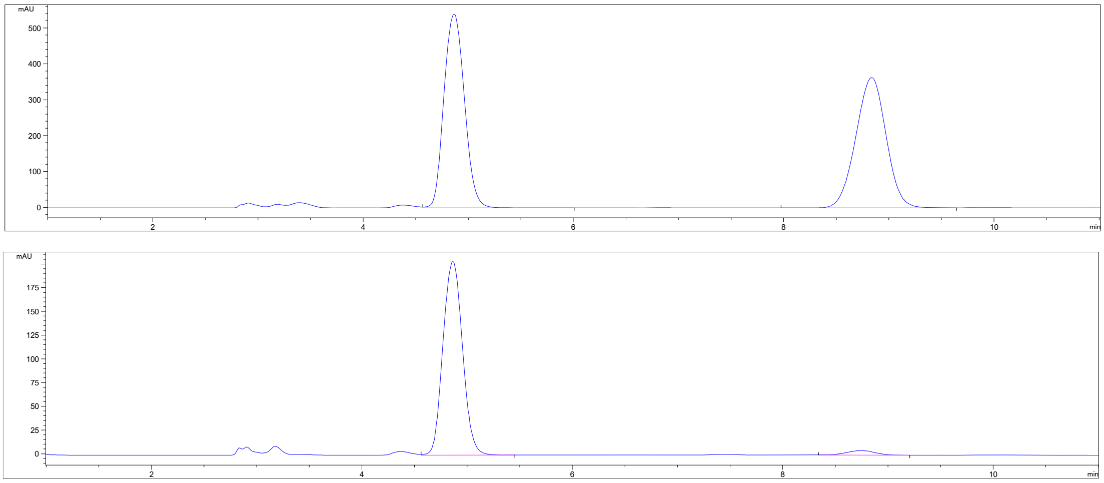

Figure S19. HPLC spectrum for **2e**, related to **Figure 2**.

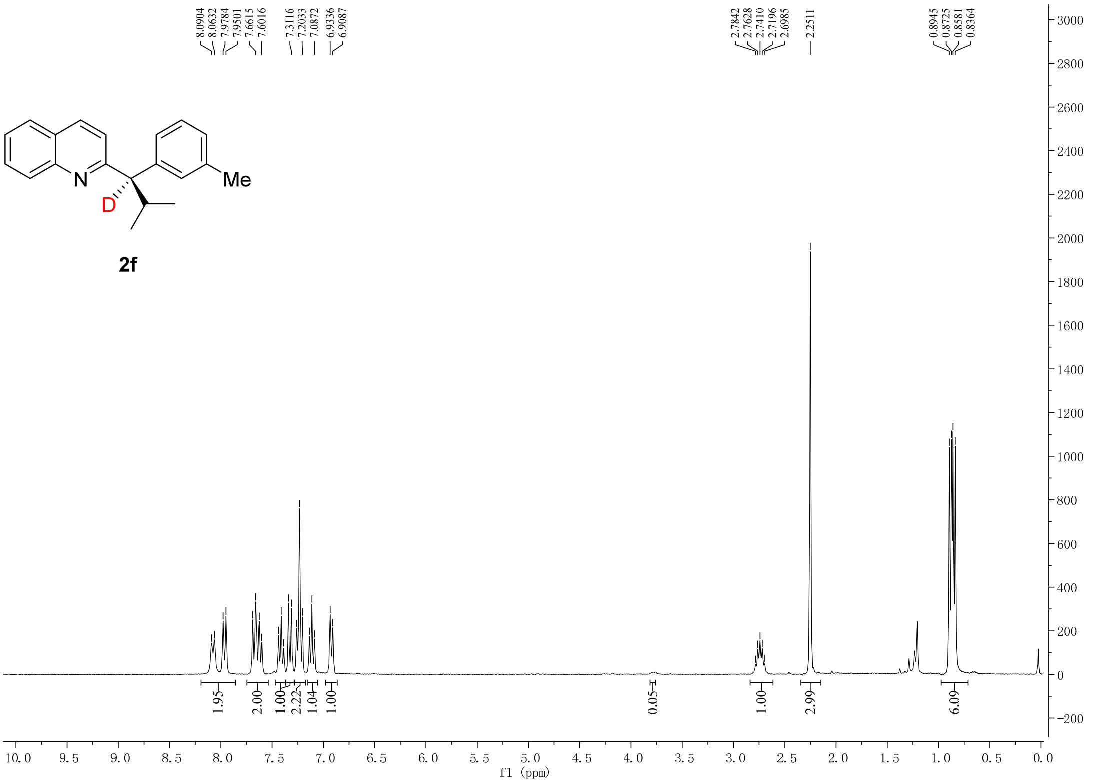

Figure S20. ^{1}H NMR spectrum for **2f**, related to **Figure 2**.

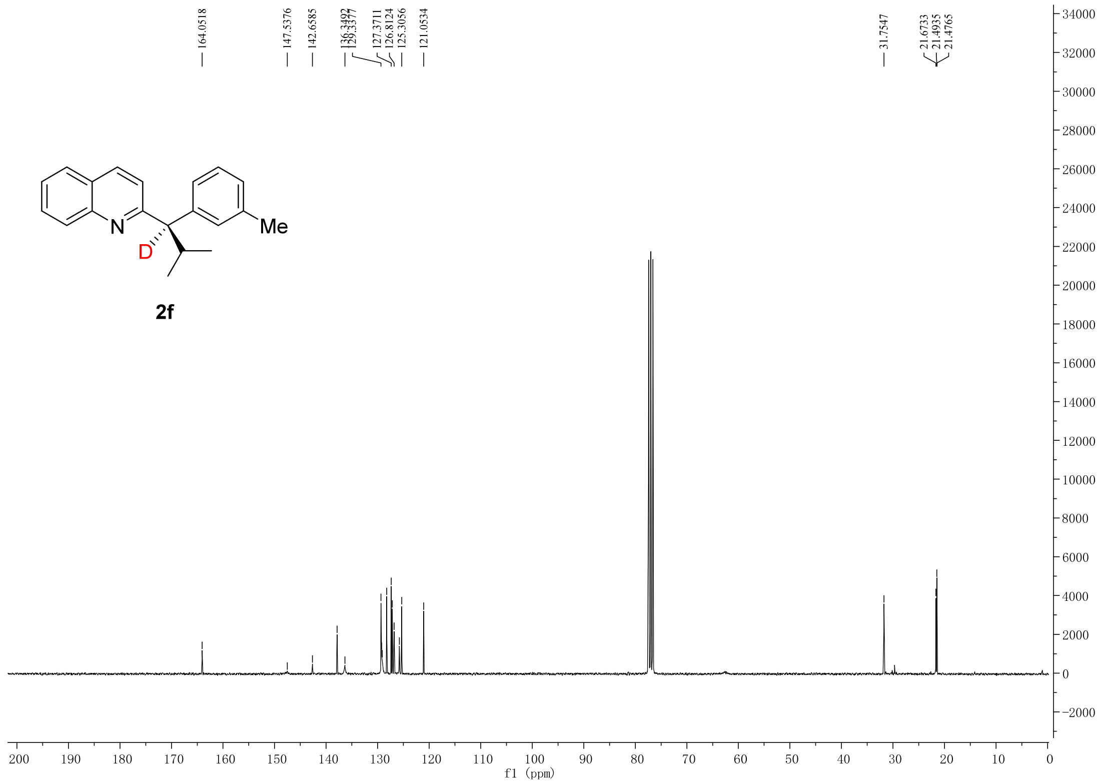

Figure S21. ^{13}C NMR spectrum for **2f**, related to **Figure 2**.

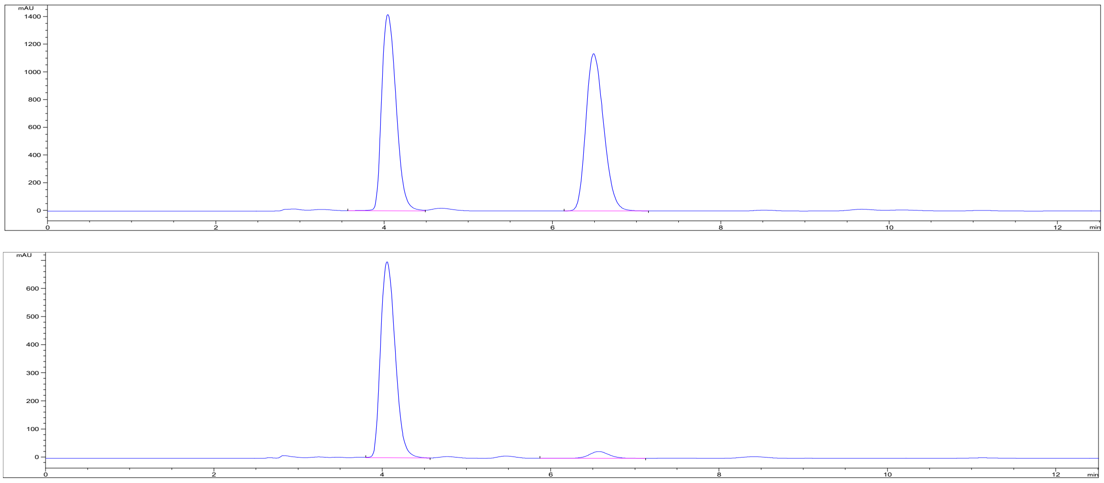

Figure S22. HPLC spectrum for **2f**, related to **Figure 2**.

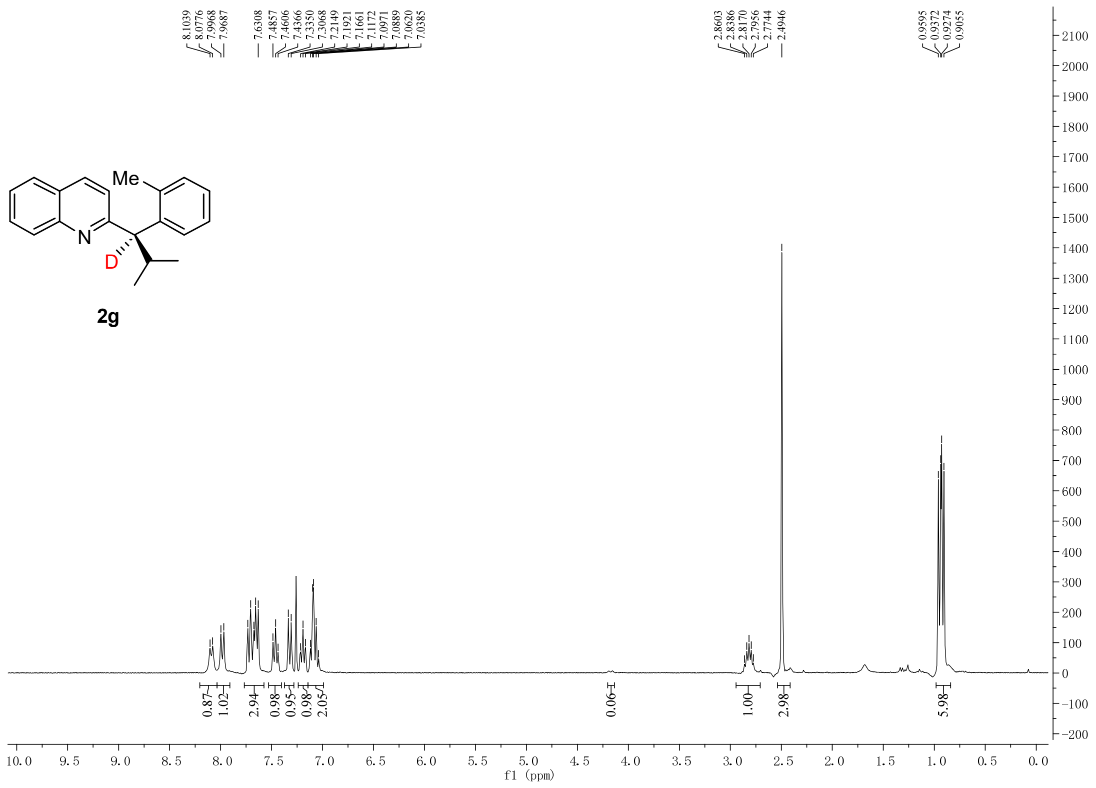

Figure S23. ^{1}H NMR spectrum for **2g**, related to **Figure 2**.

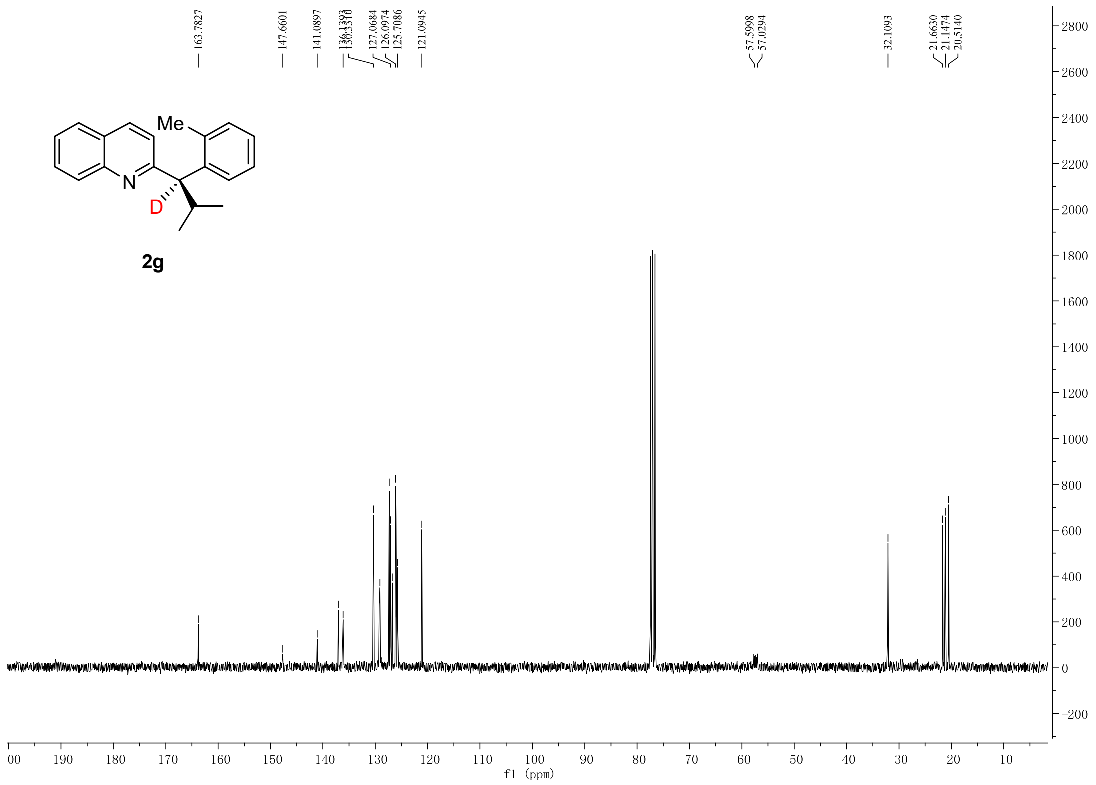

Figure S24. ^{13}C NMR spectrum for **2g**, related to **Figure 2**.

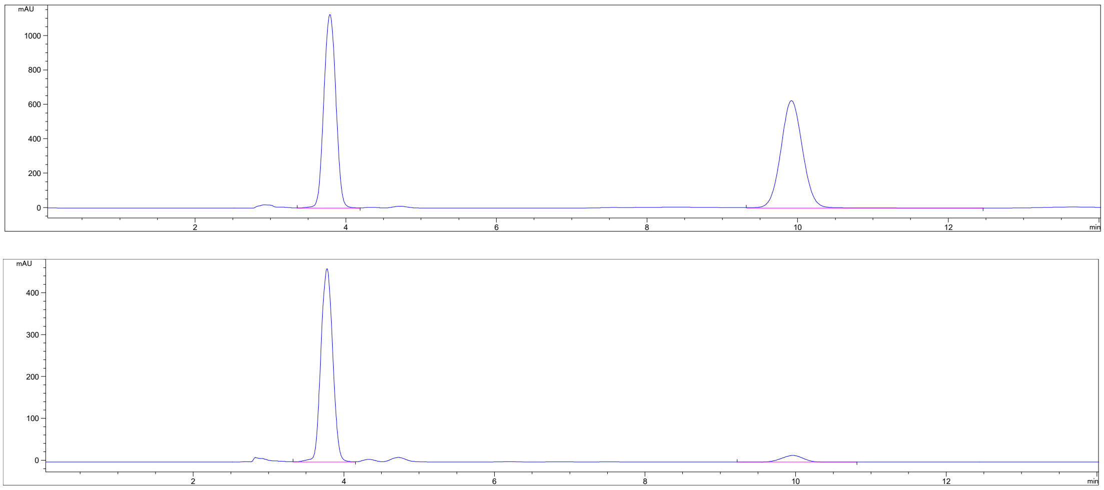

Figure S25. HPLC spectrum for **2g**, related to **Figure 2**.

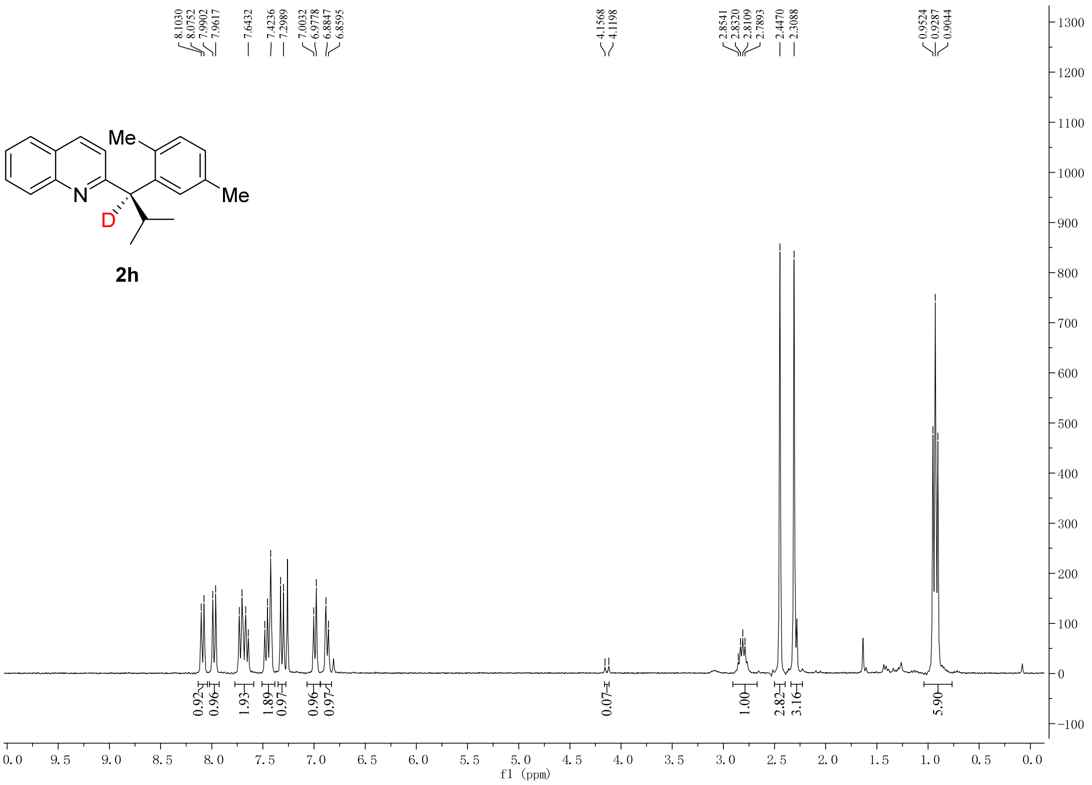

Figure S26. ^{1}H NMR spectrum for **2h**, related to **Figure 2**.

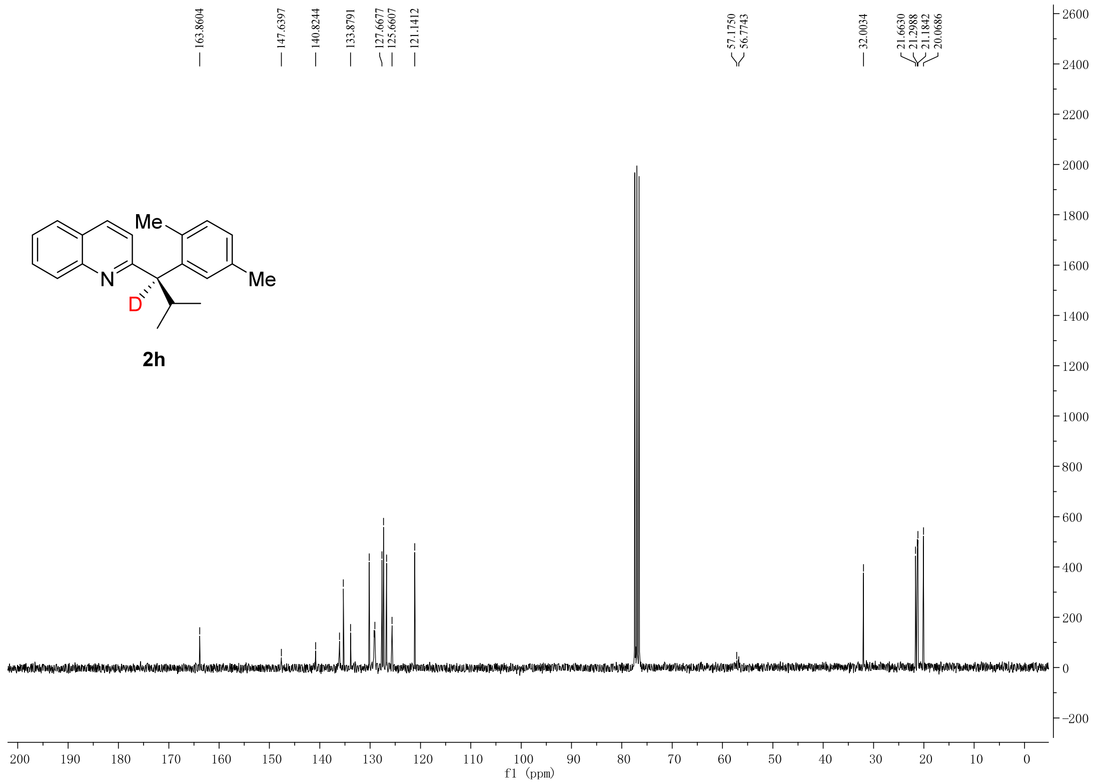

Figure S27. ^{13}C NMR spectrum for **2h**, related to **Figure 2**.

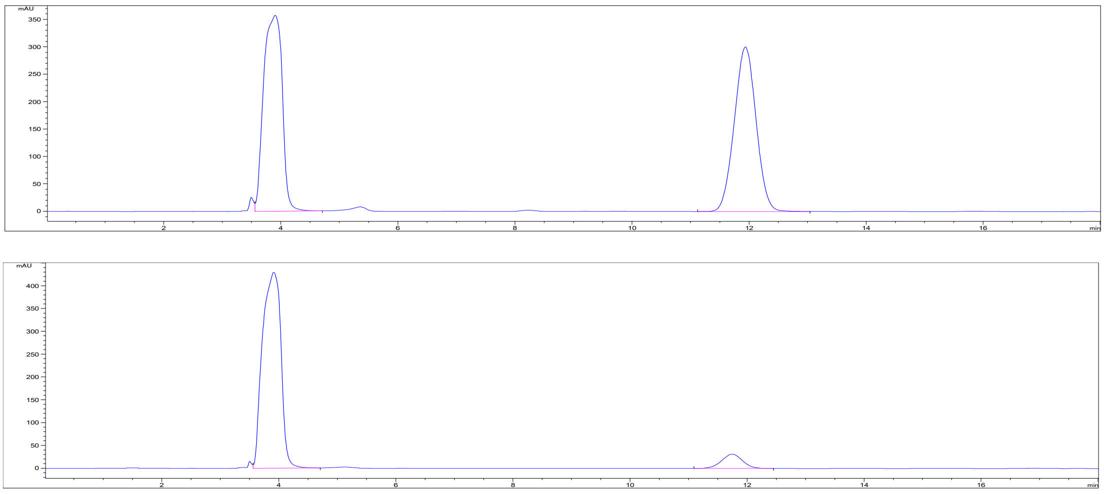

Figure S28. HPLC spectrum for **2h**, related to **Figure 2**.

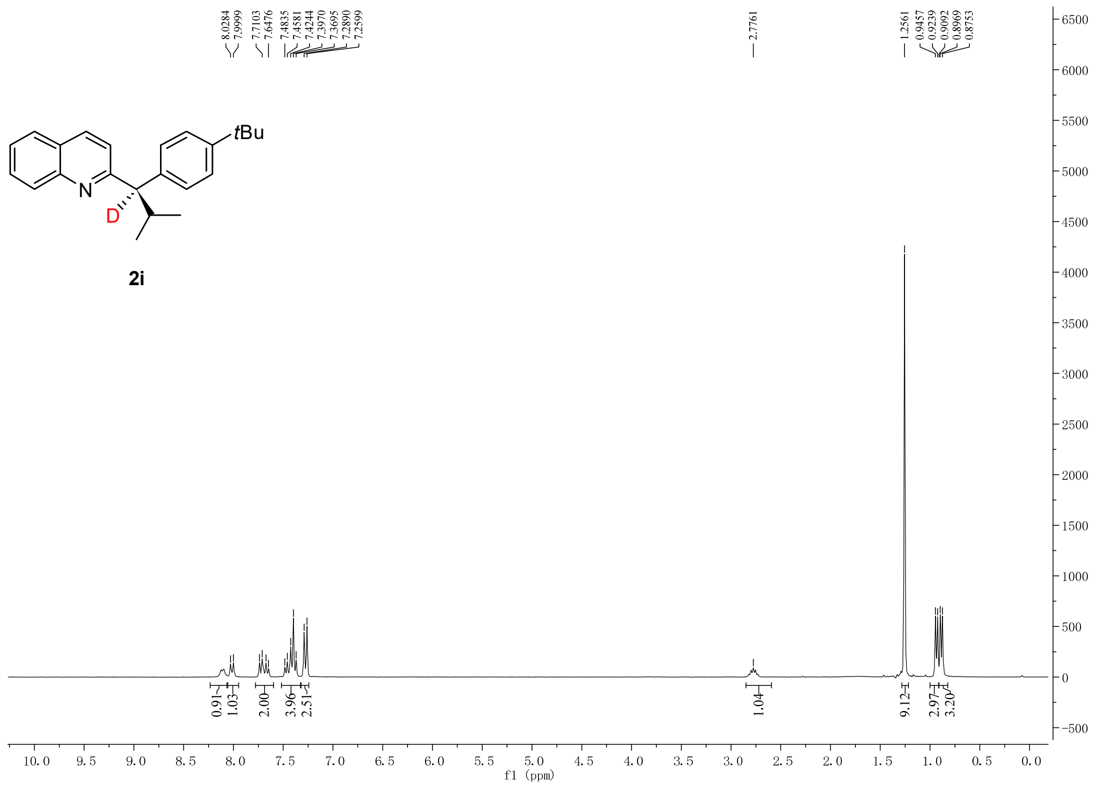

Figure S29. ^{1}H NMR spectrum for **2i**, related to **Figure 2**.

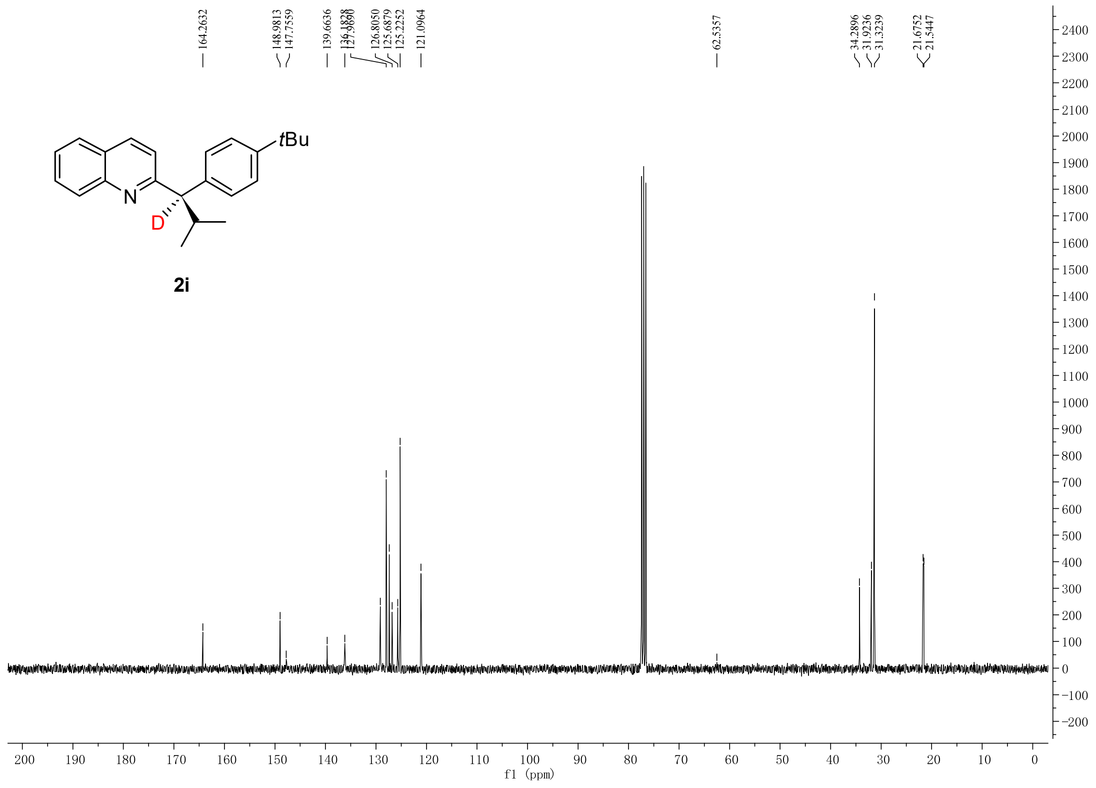

Figure S30. ^{13}C NMR spectrum for **2i**, related to **Figure 2**.

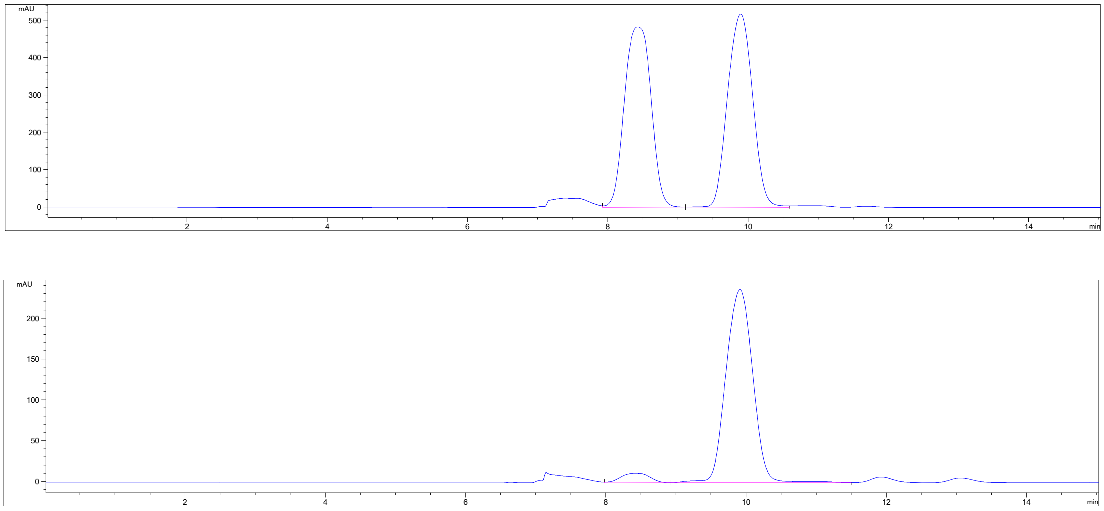

Figure S31. HPLC spectrum for **2i**, related to **Figure 2**.

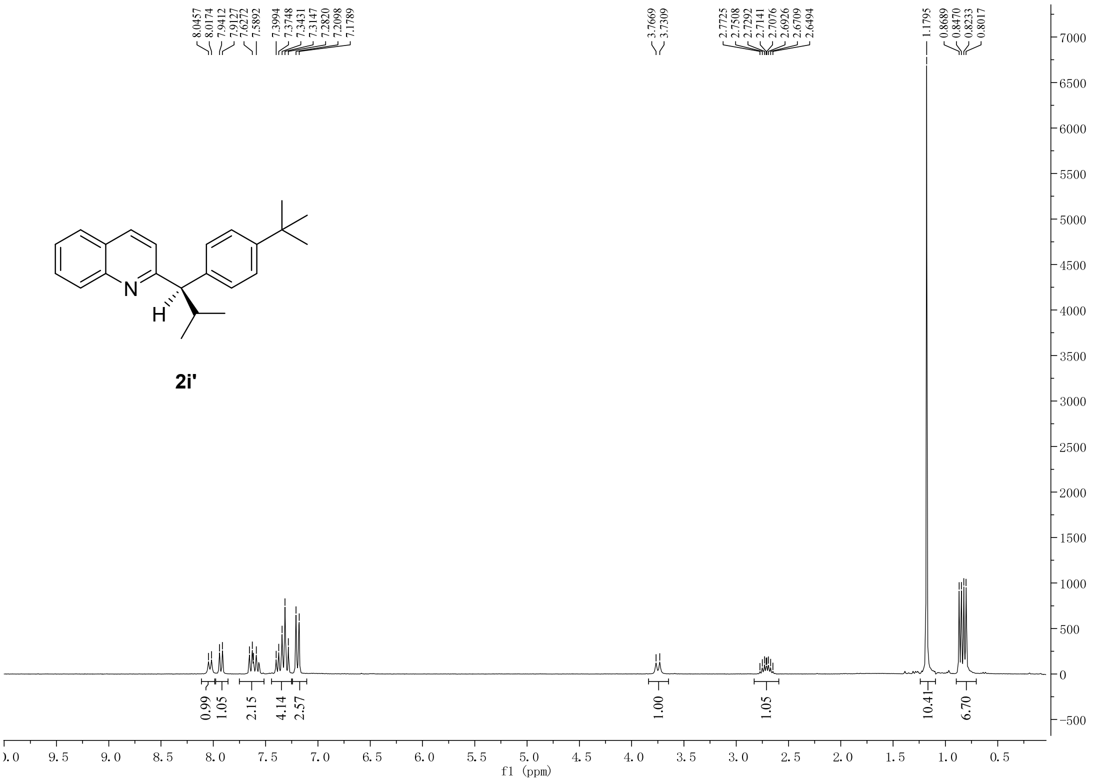

Figure S32. ^{1}H NMR spectrum for **2i'**, related to **Figure 2**.

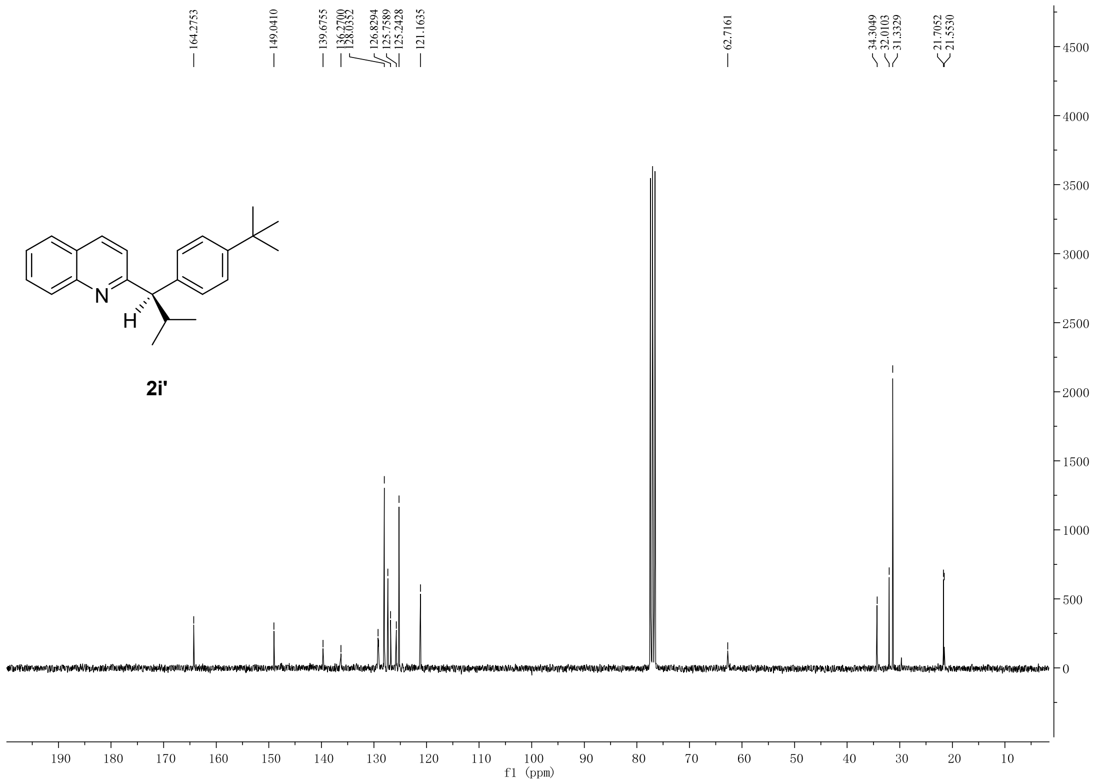

Figure S33. ^{13}C NMR spectrum for **2i'**, related to **Figure 2**.

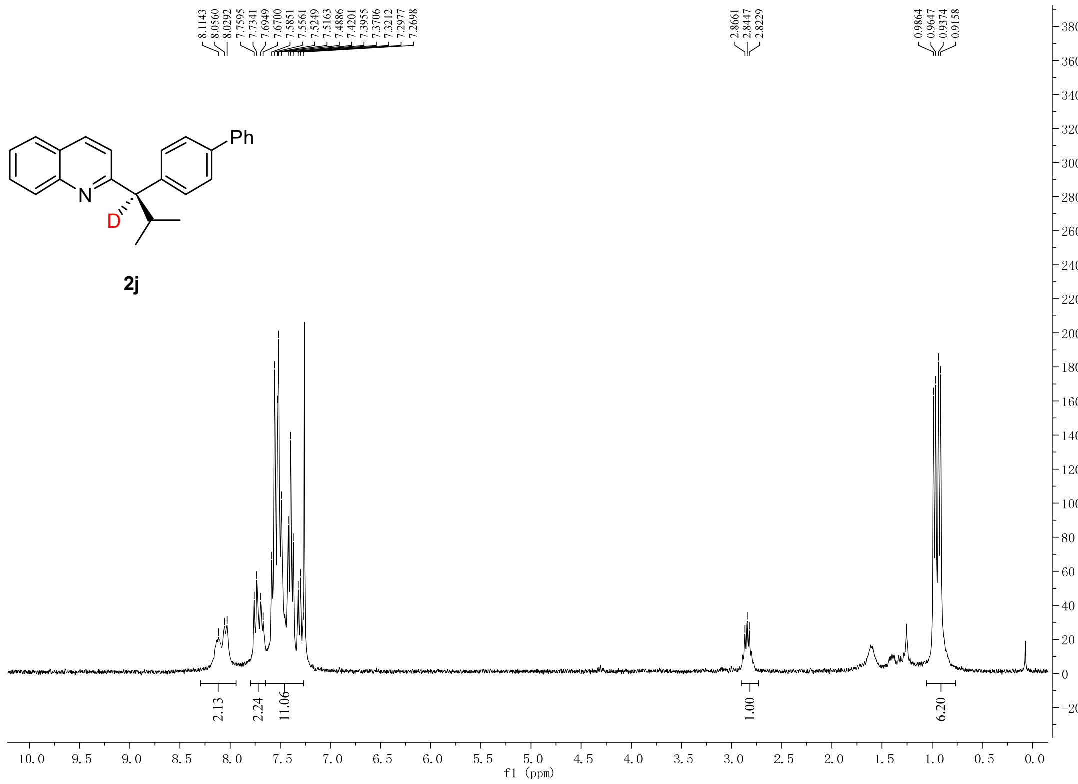

Figure S34. ^{1}H NMR spectrum for **2j**, related to **Figure 2**.

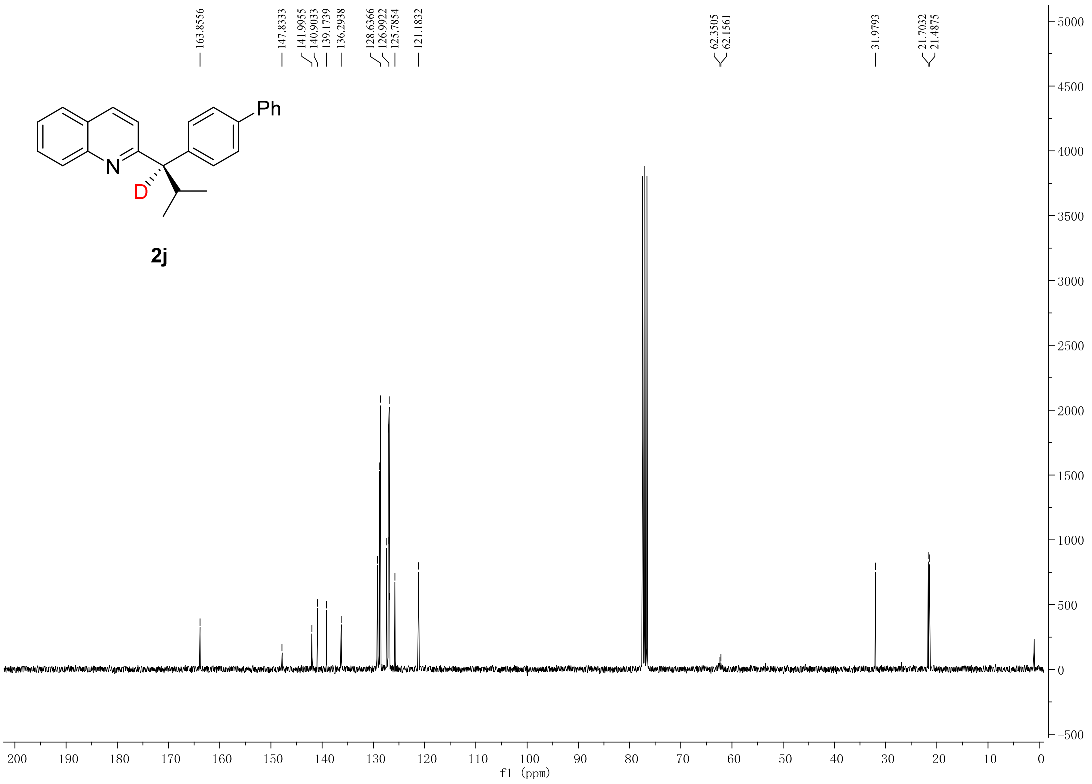

Figure S35. ^{13}C NMR spectrum for **2j**, related to **Figure 2**.

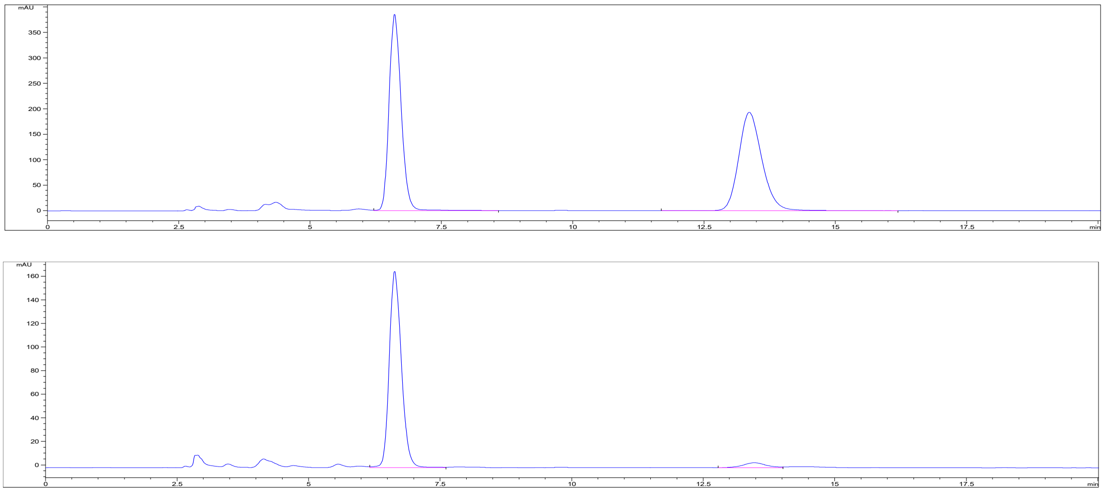

Figure S36. HPLC spectrum for **2j**, related to **Figure 2**.

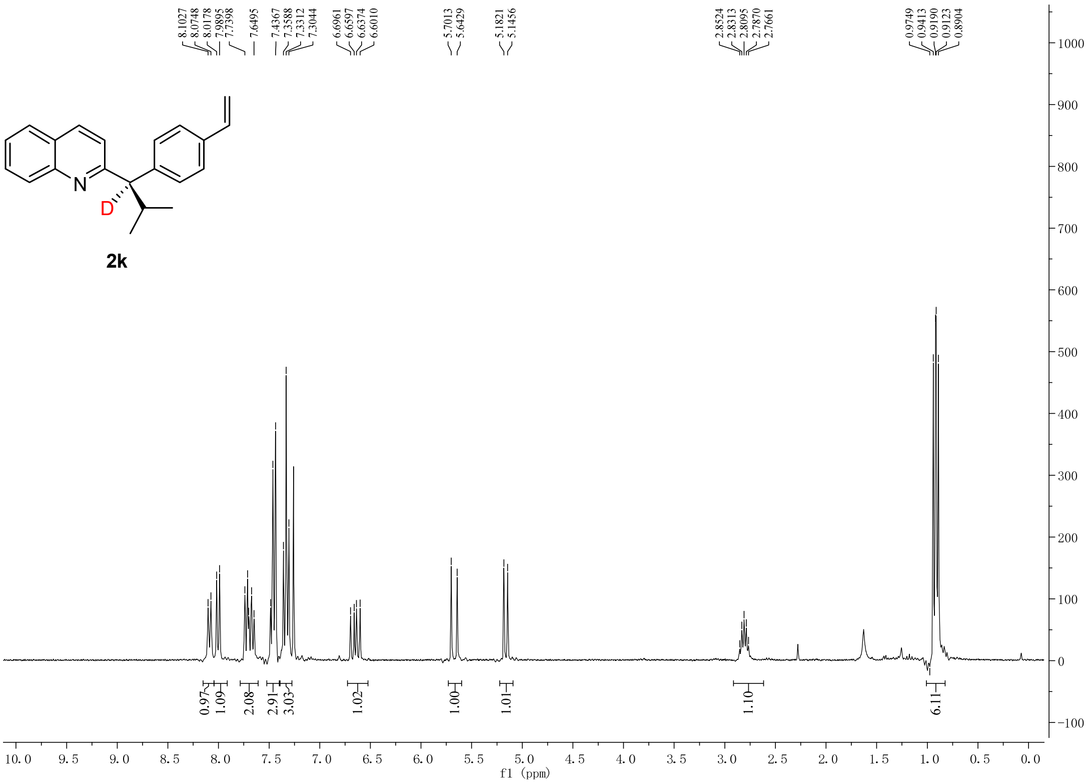

Figure S37. ^{1}H NMR spectrum for **2k**, related to **Figure 2**.

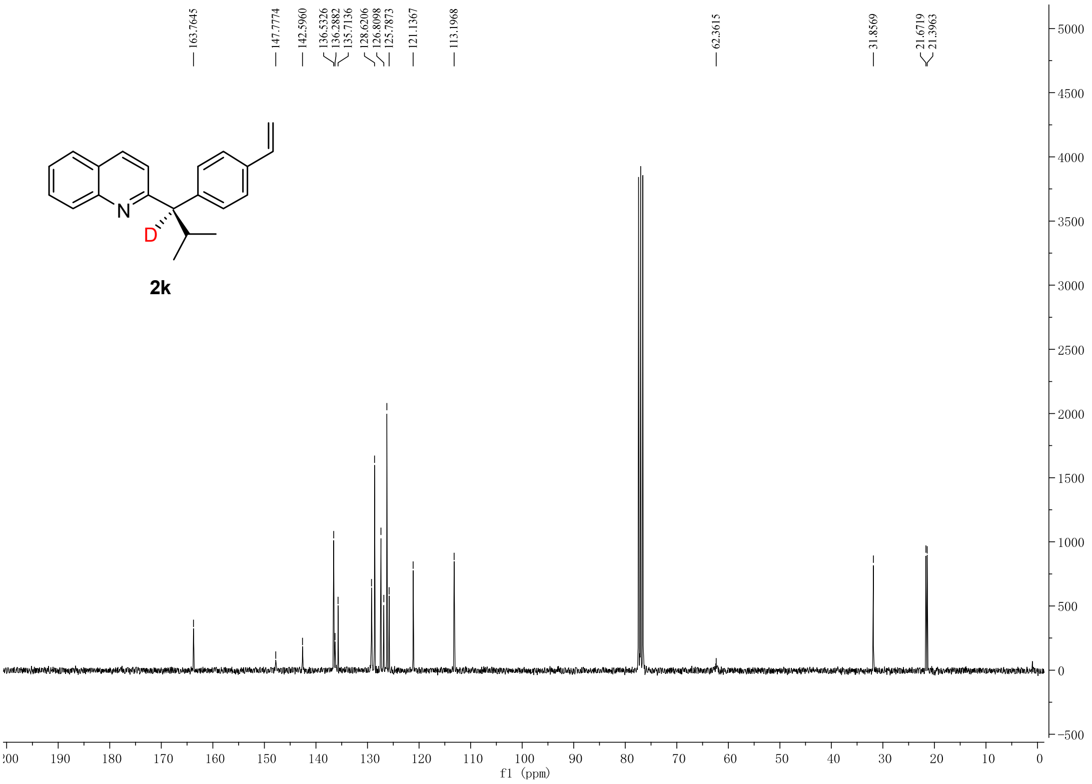

Figure S38. ^{13}C NMR spectrum for **2k**, related to **Figure 2**.

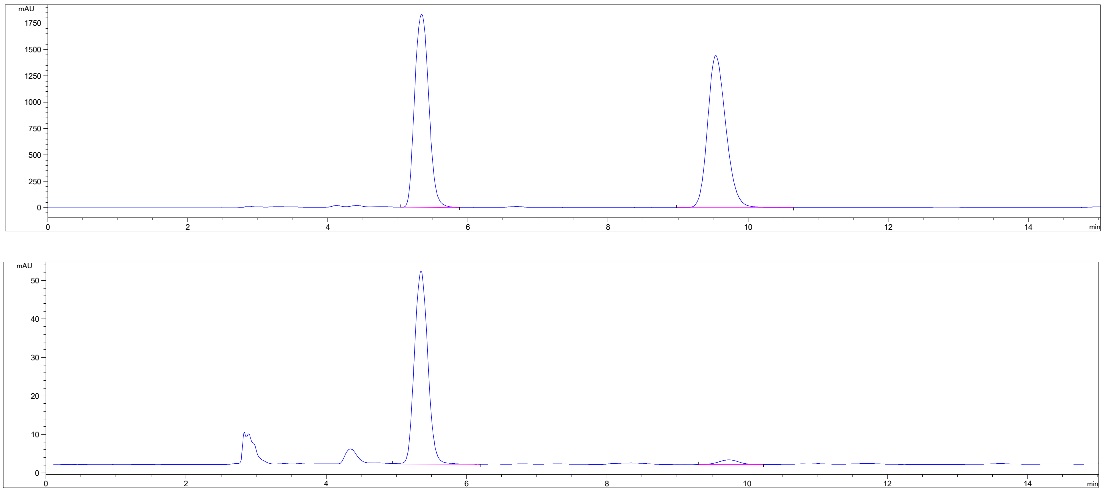

Figure S39. HPLC spectrum for **2k**, related to **Figure 2**.

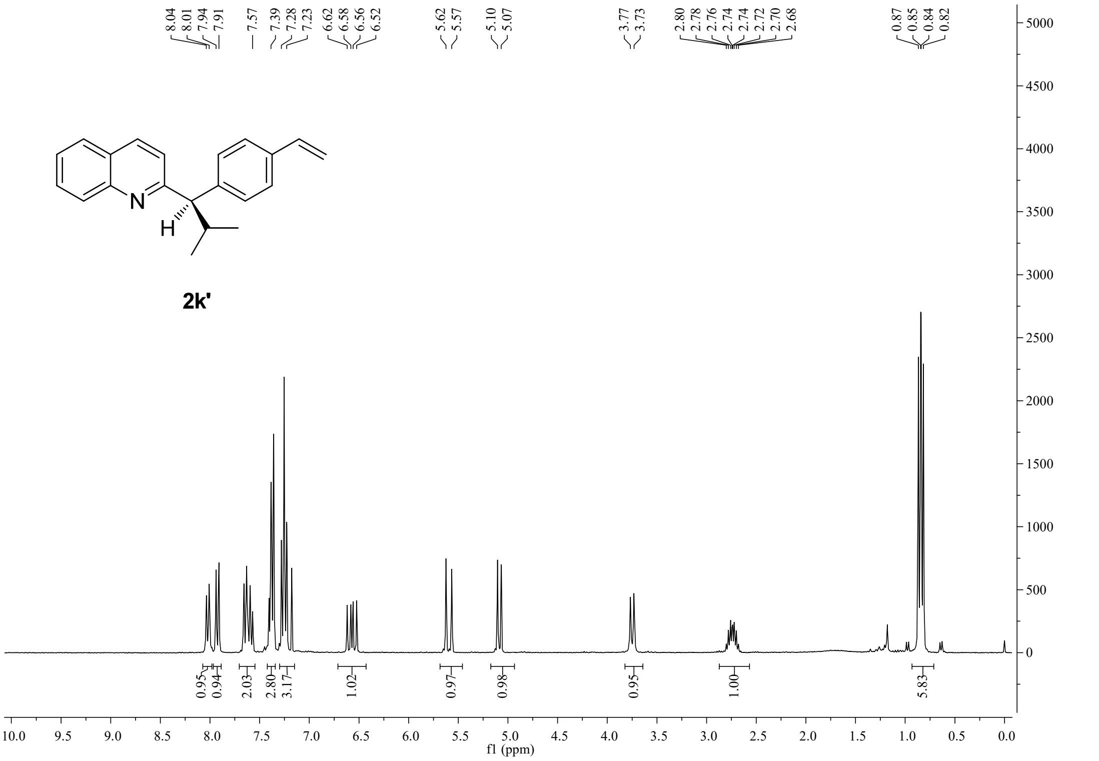

Figure S40. ^{1}H NMR spectrum for **2k'**, related to **Figure 2**.

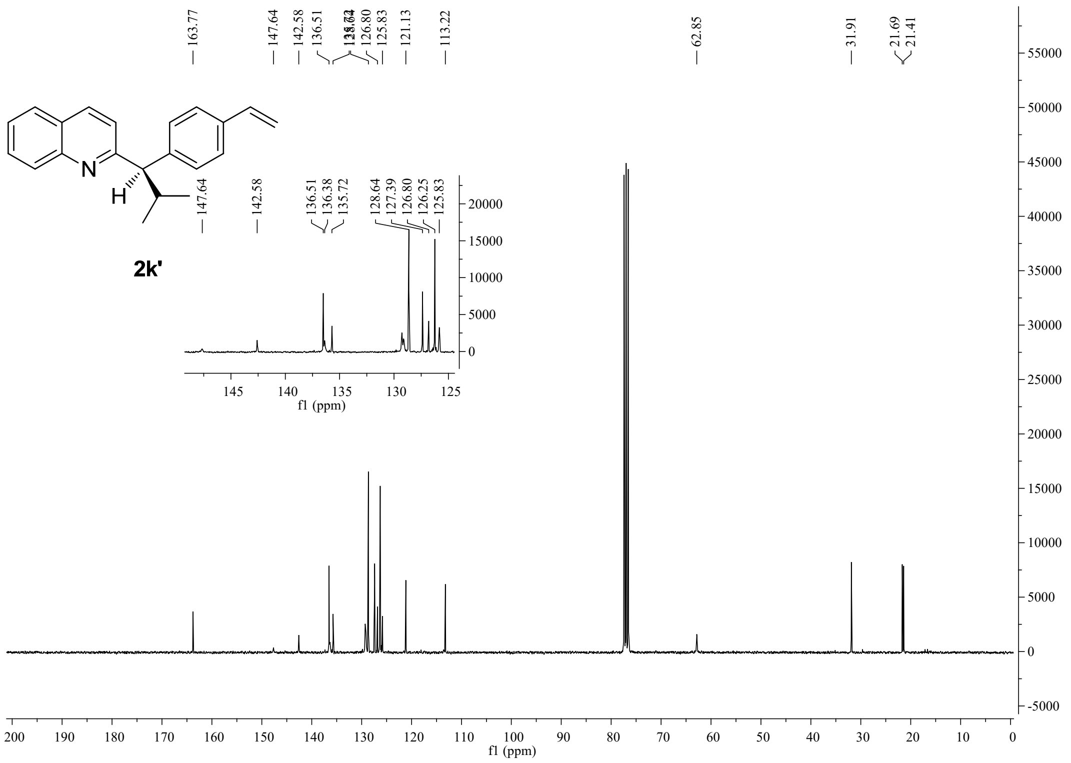

Figure S41. ^{13}C NMR spectrum for **2k'**, related to **Figure 2**.

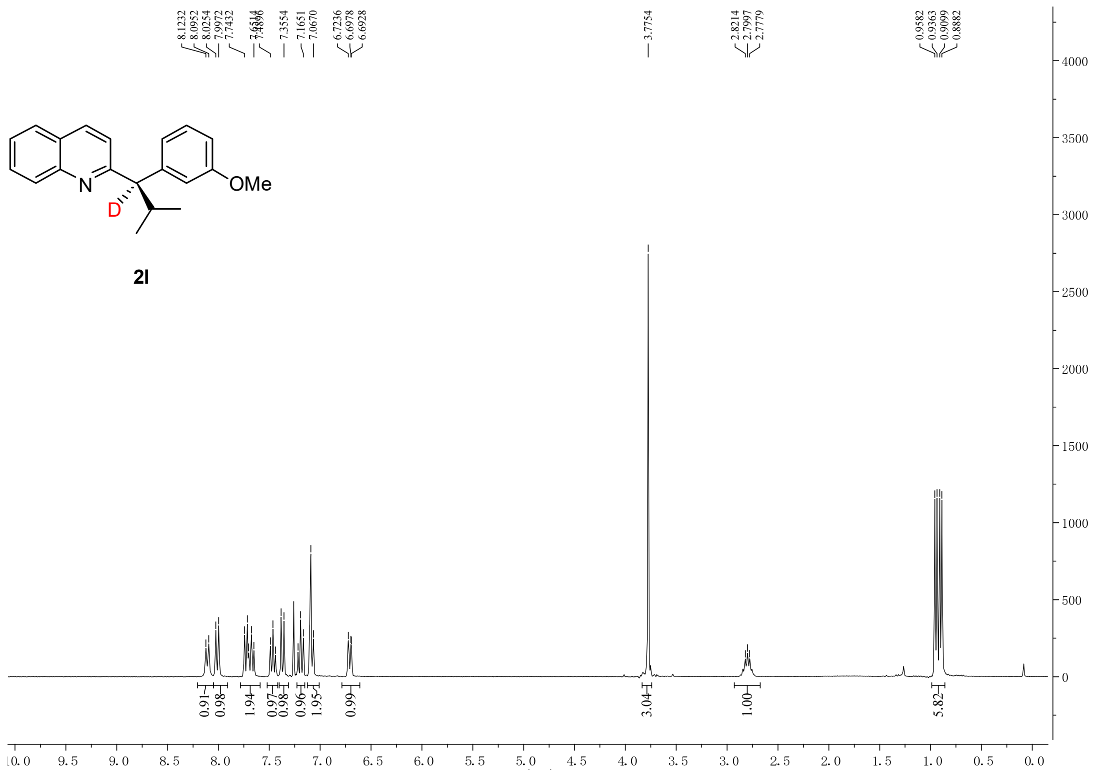

Figure S42. ^{1}H NMR spectrum for **2l**, related to **Figure 2**.

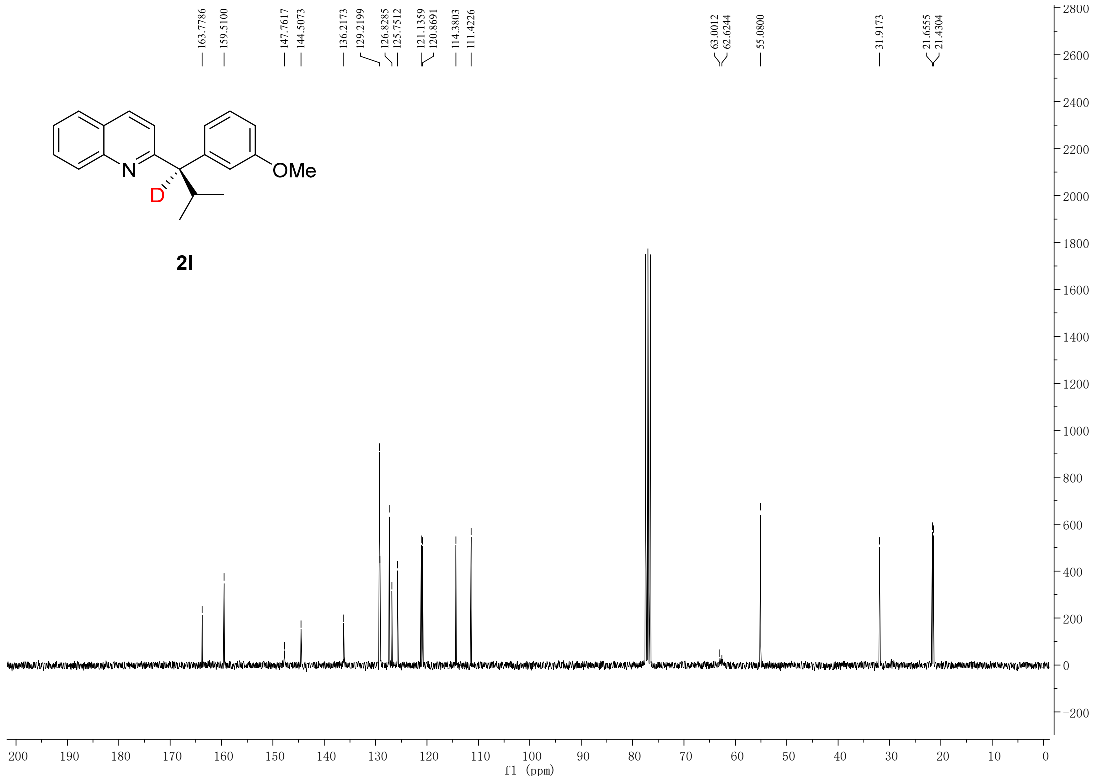

Figure S43. ^{13}C NMR spectrum for **2l**, related to **Figure 2**.

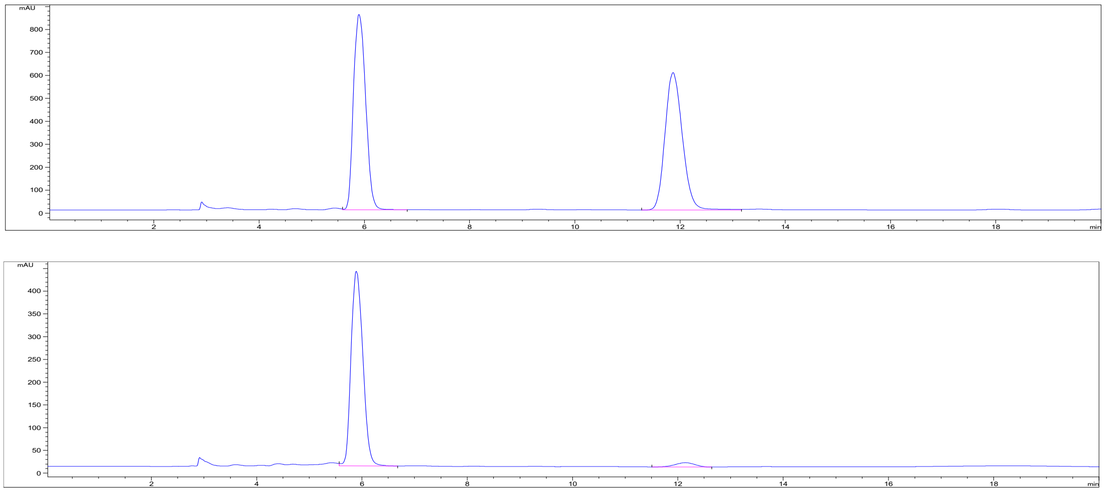

Figure S44. HPLC spectrum for **2l**, related to **Figure 2**.

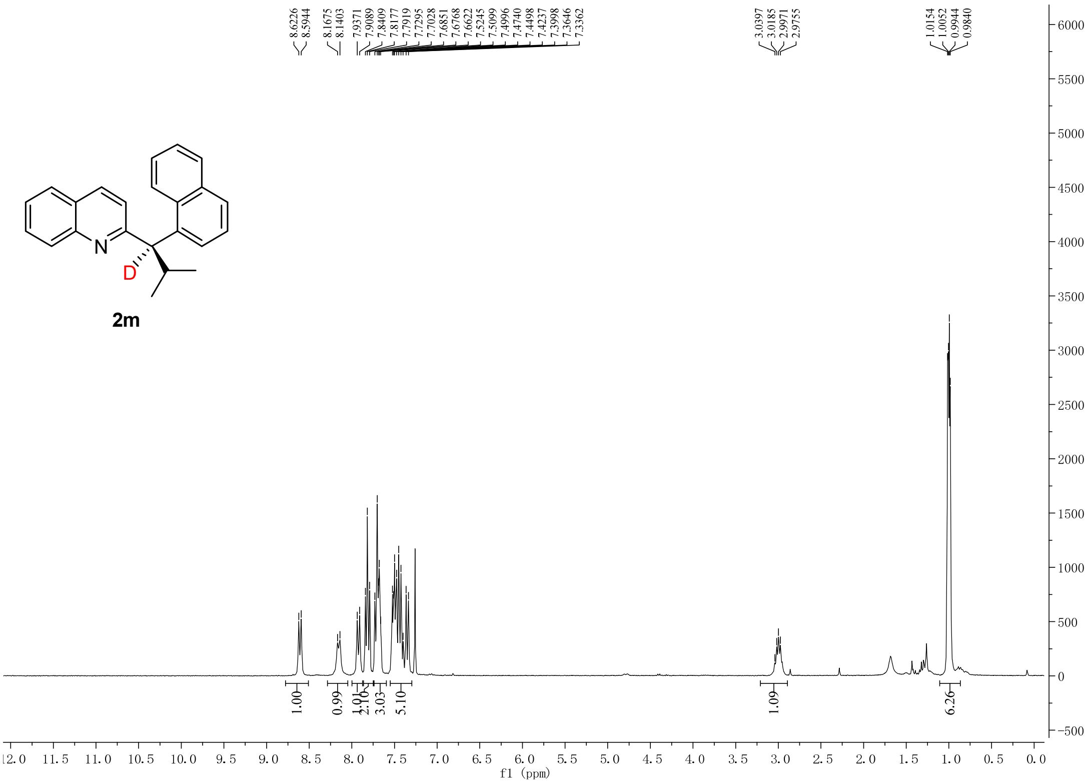

Figure S45. ^{1}H NMR spectrum for **2m**, related to **Figure 2**.

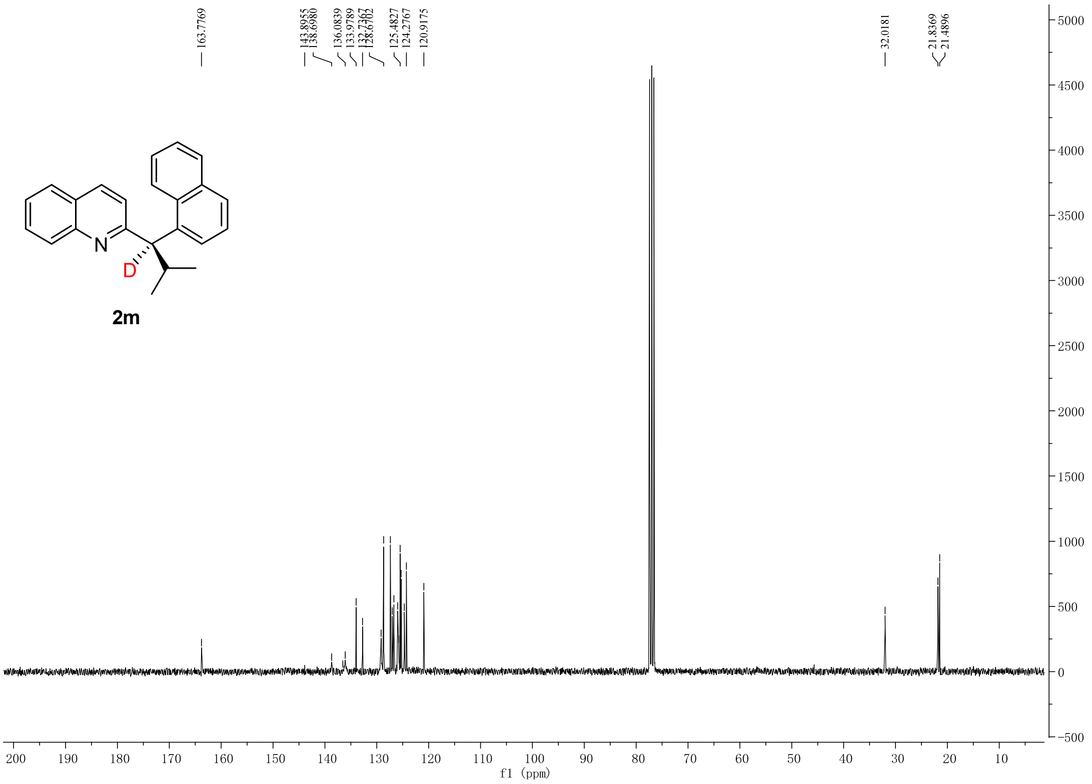

Figure S46. ^{13}C NMR spectrum for **2m**, related to **Figure 2**.

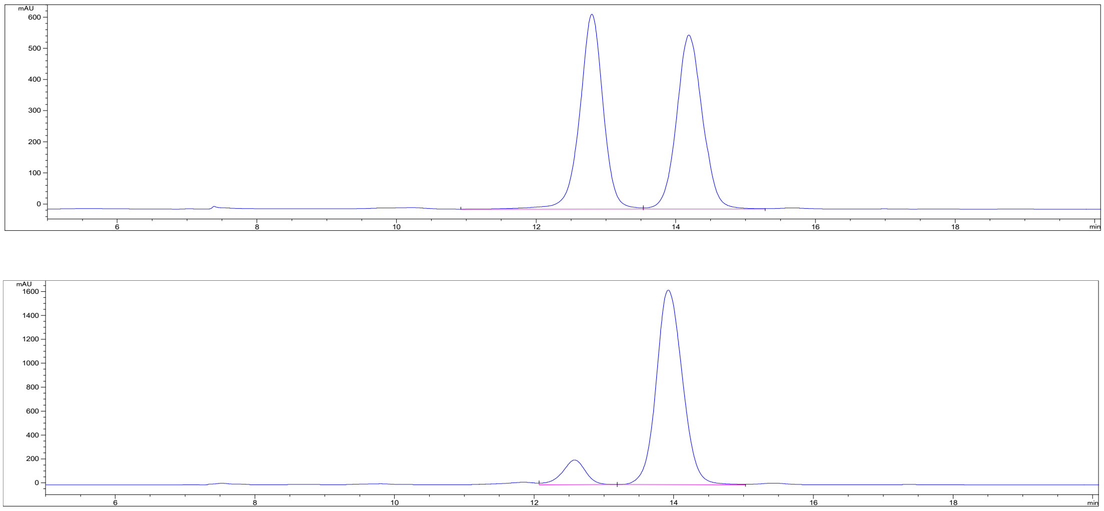

Figure S47. HPLC spectrum for **2m**, related to **Figure 2**.

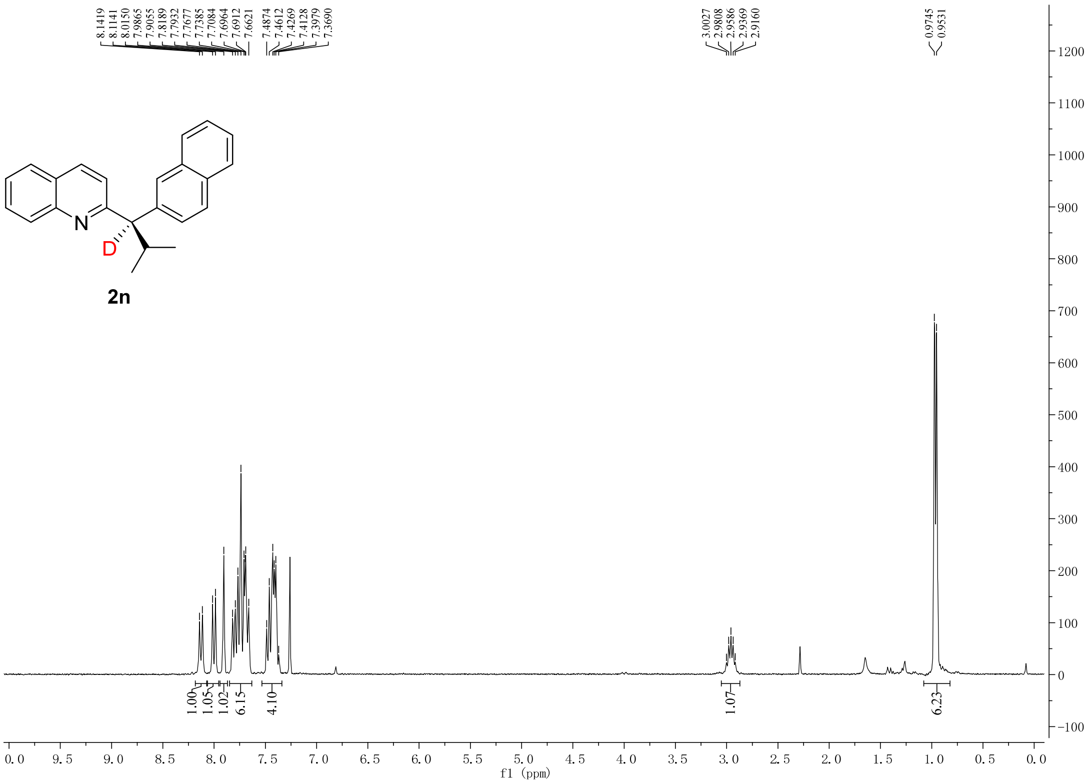

Figure S48. ^{1}H NMR spectrum for **2n**, related to **Figure 2**.

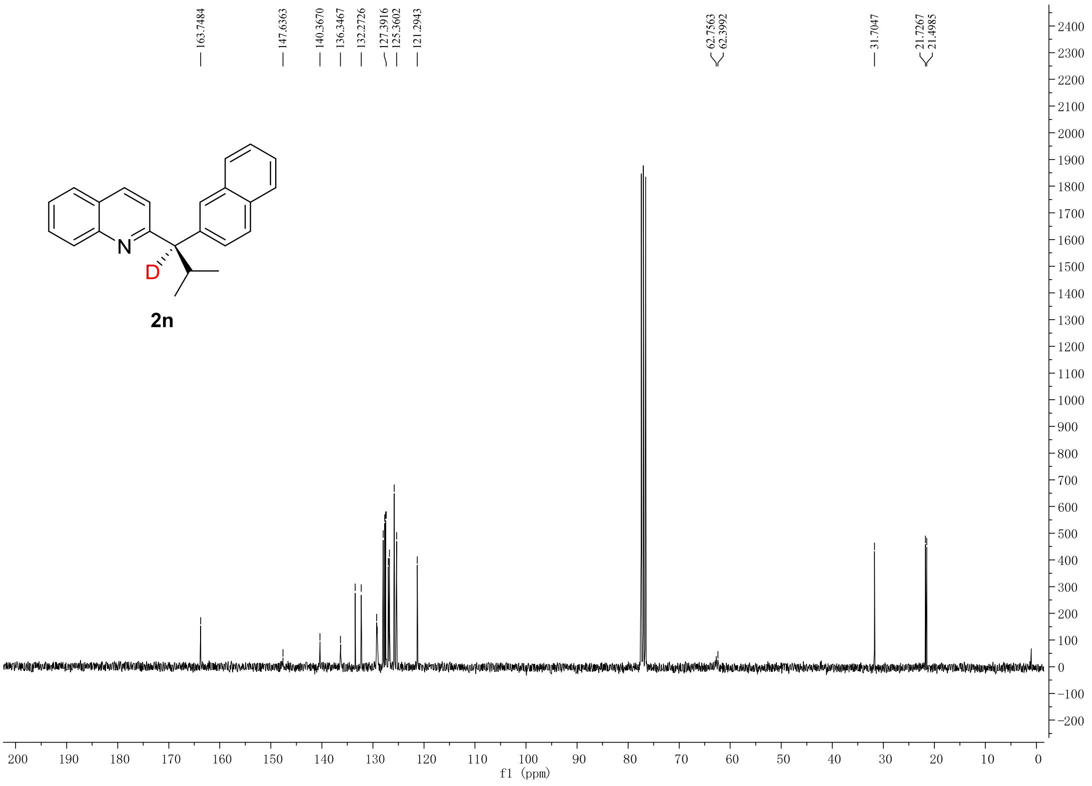

Figure S49. ^{13}C NMR spectrum for **2n**, related to **Figure 2**.

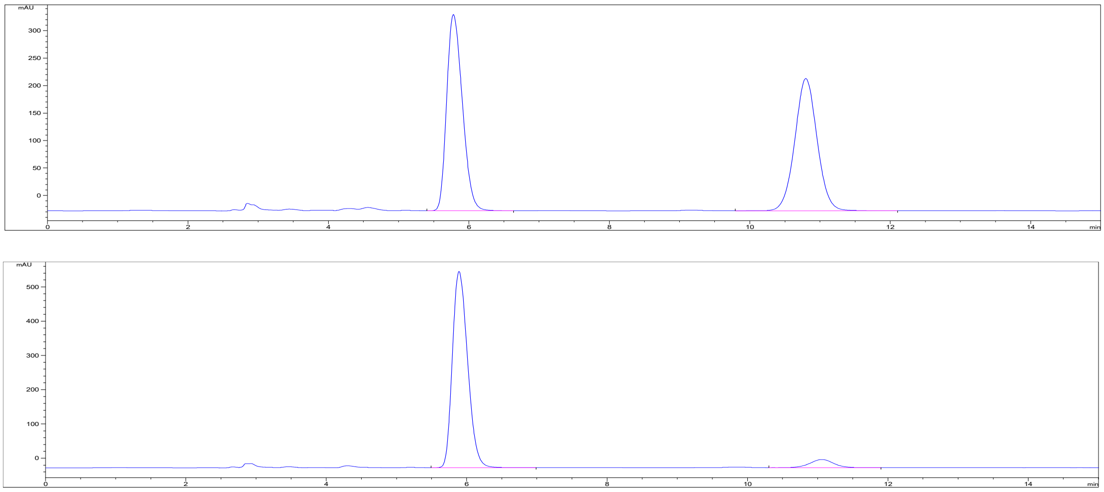

Figure S50. HPLC spectrum for **2n**, related to **Figure 2**.

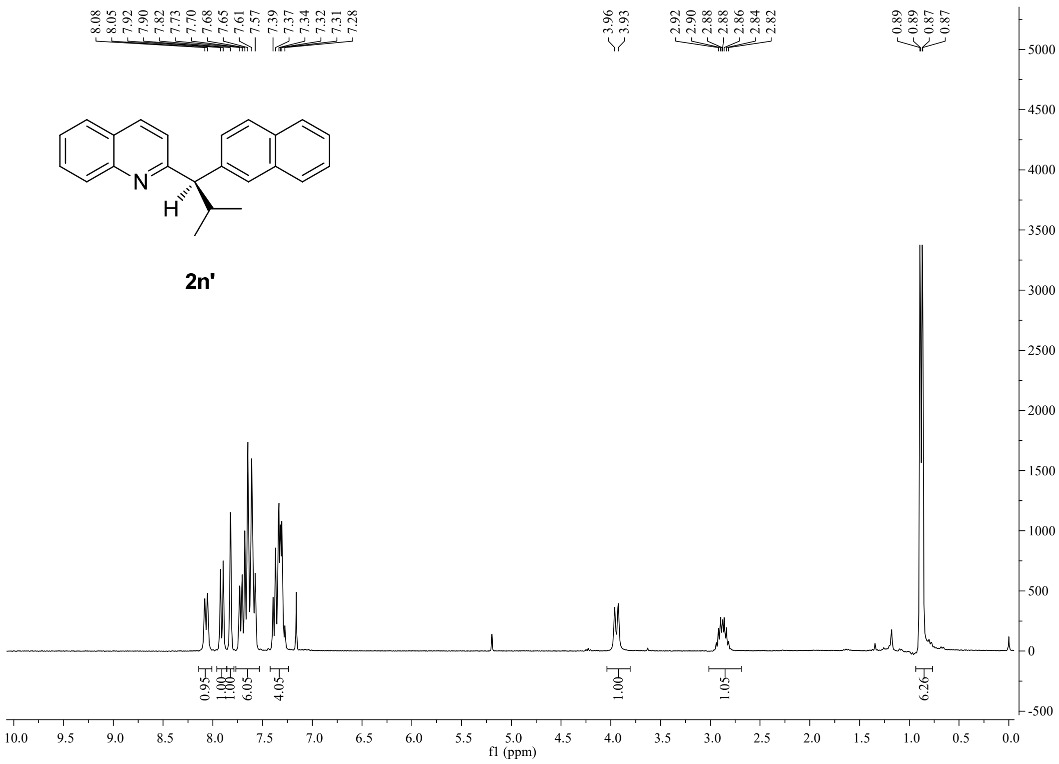

Figure S51. ^{1}H NMR spectrum for **2n'**, related to **Figure 2**.

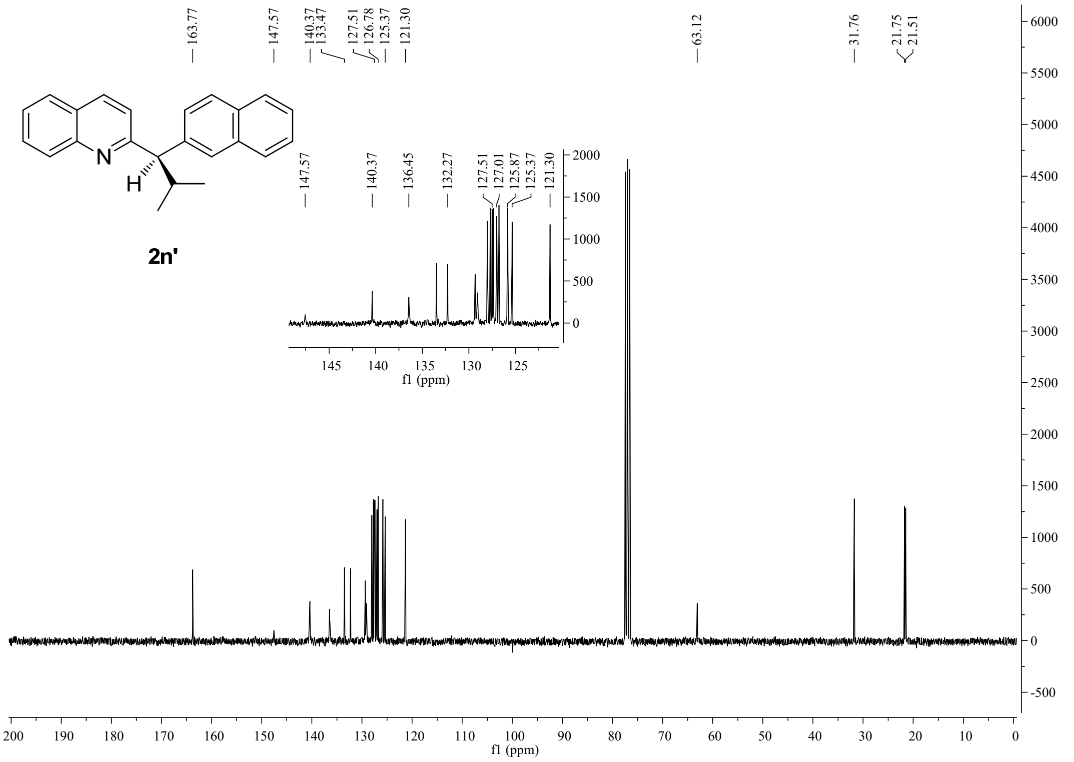

Figure S52. ^{13}C NMR spectrum for **2n'**, related to **Figure 2**.

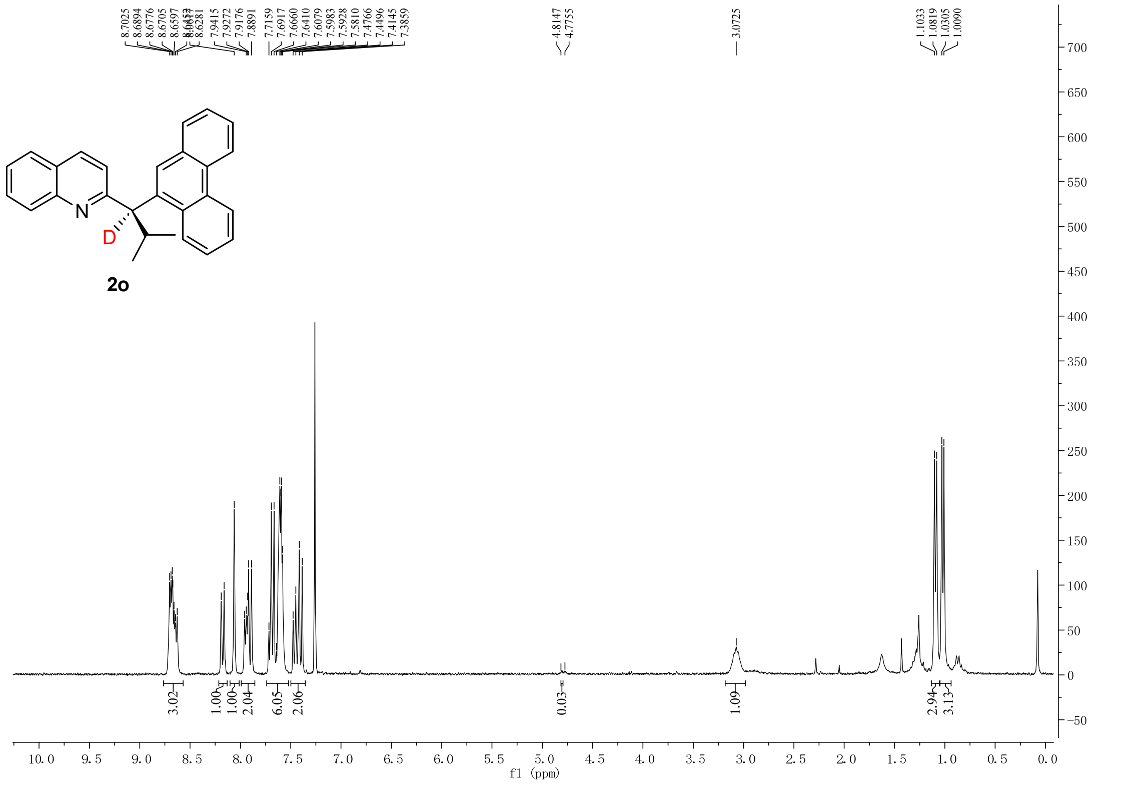

Figure S53. ^{1}H NMR spectrum for **2o**, related to **Figure 2**.

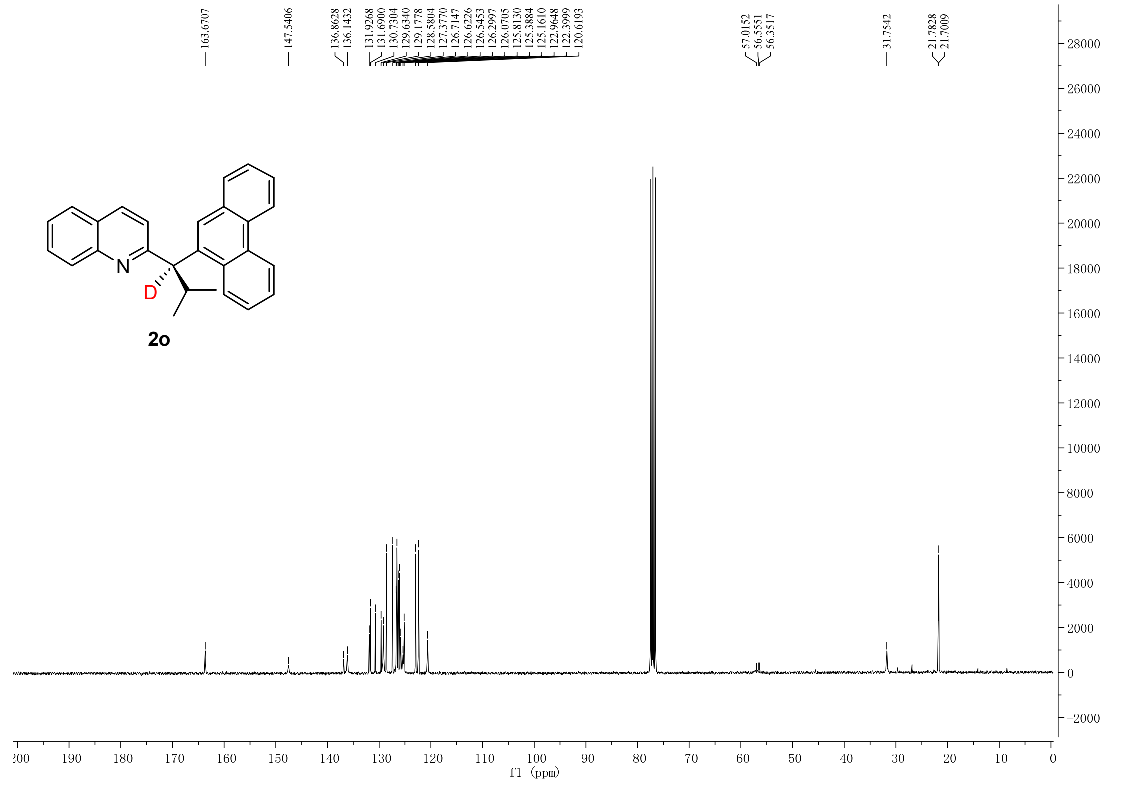

Figure S54. ^{13}C NMR spectrum for **2o**, related to **Figure 2**.

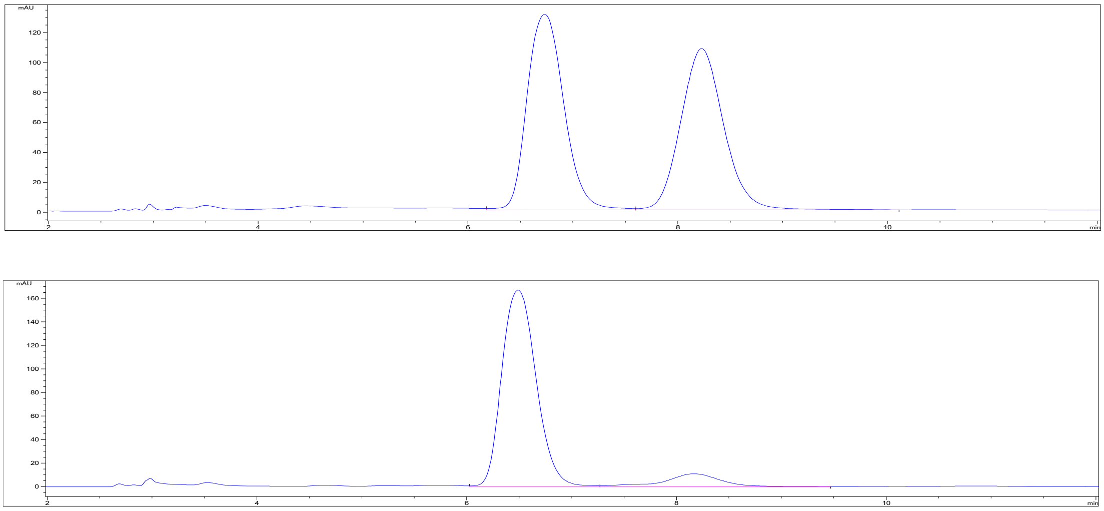

Figure S55. HPLC spectrum for **2o**, related to **Figure 2**.

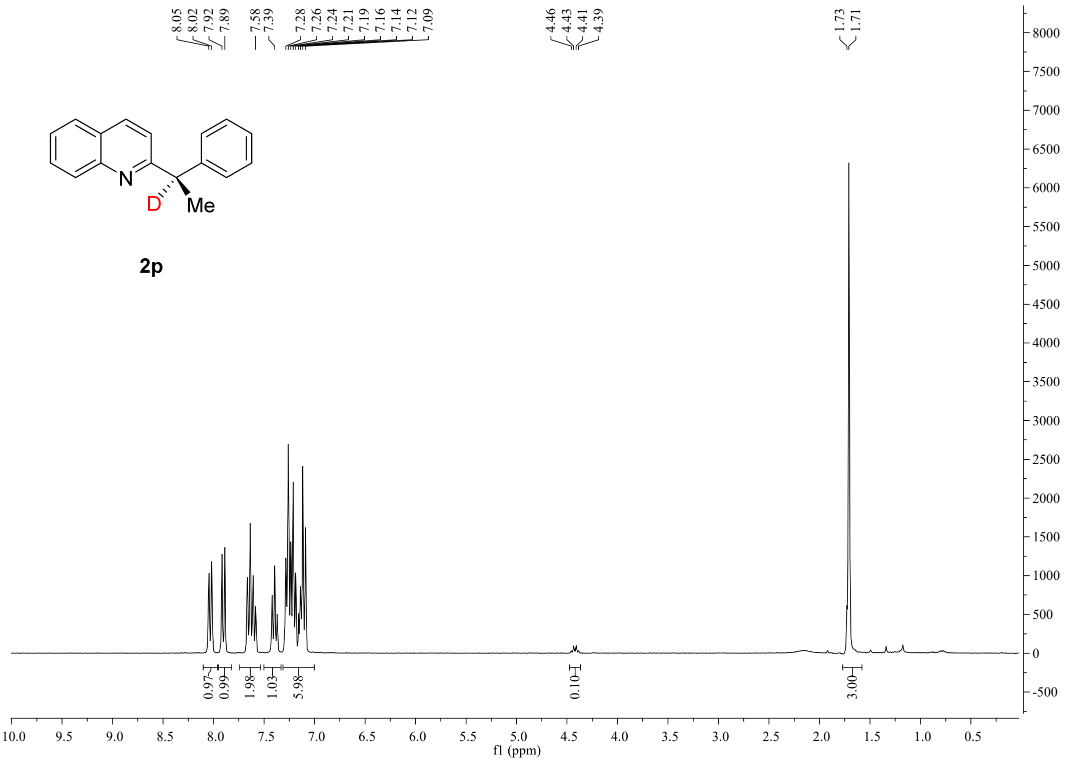

Figure S56. ^{1}H NMR spectrum for **2p**, related to **Figure 2**.

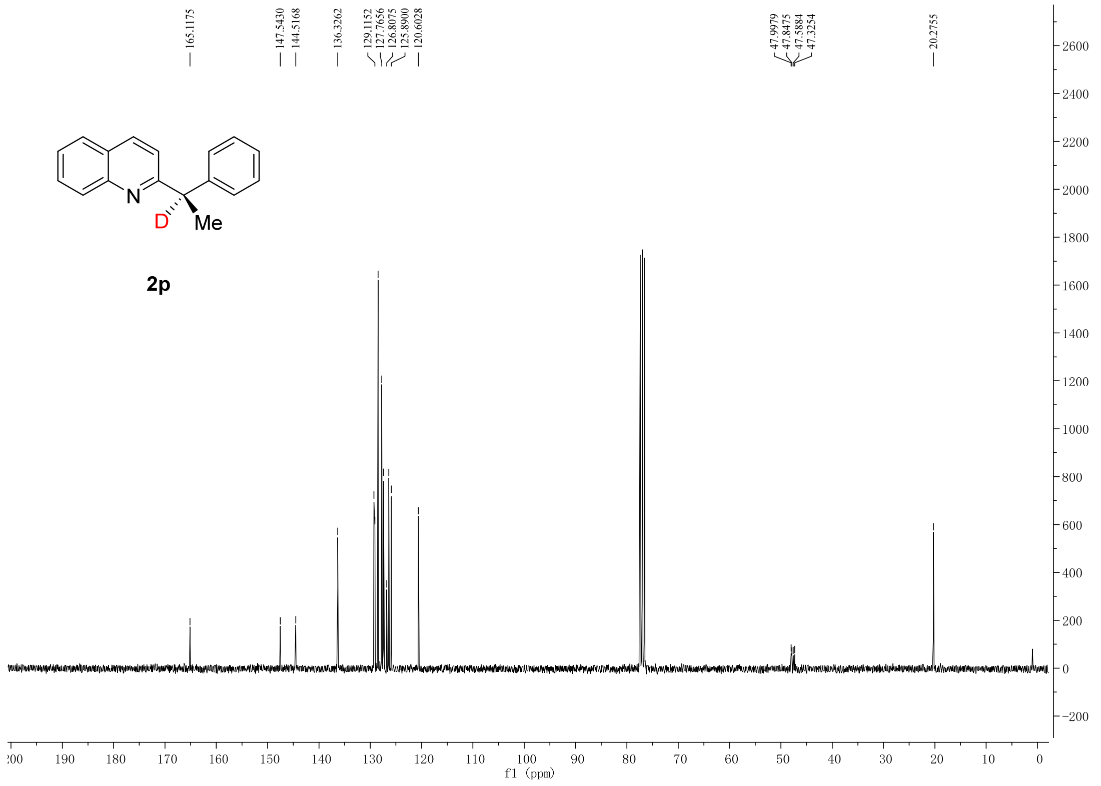

Figure S57. ^{13}C NMR spectrum for **2p**, related to **Figure 2**.

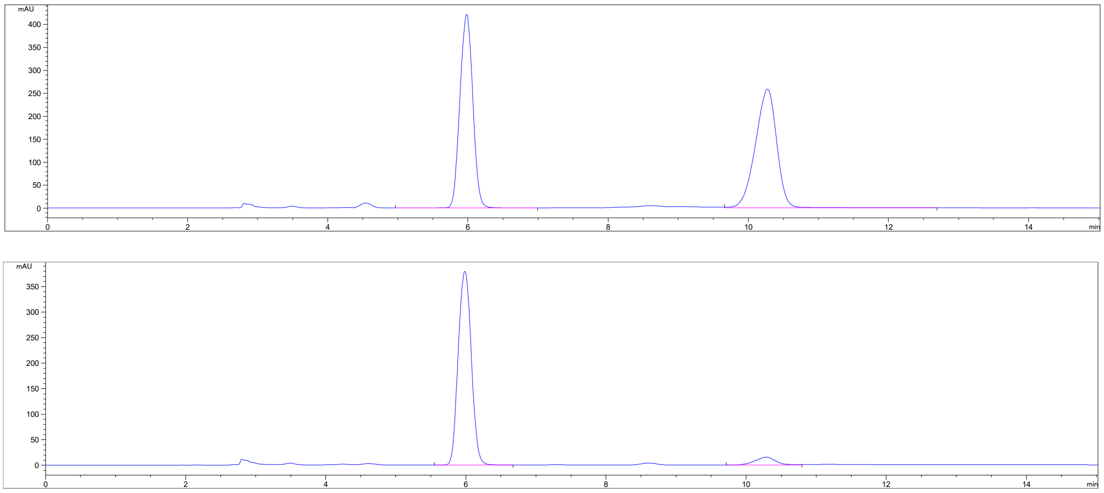

Figure S58. HPLC spectrum for **2p**, related to **Figure 2**.

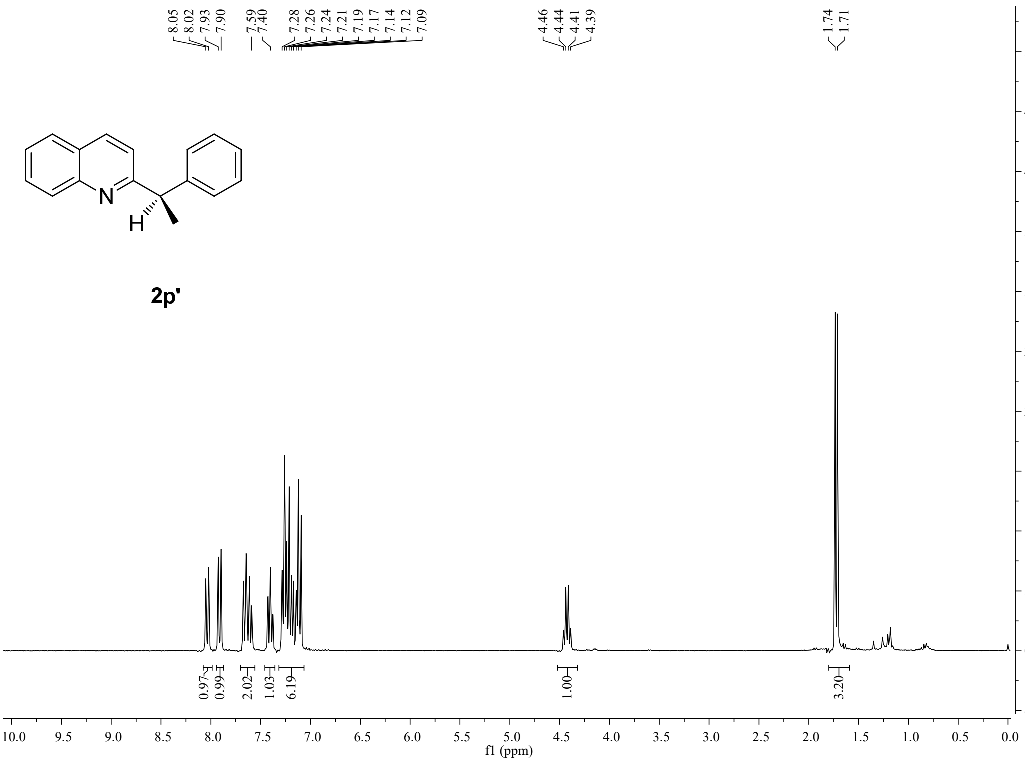

Figure S59. ^{1}H NMR spectrum for **2p'**, related to **Figure 2**.

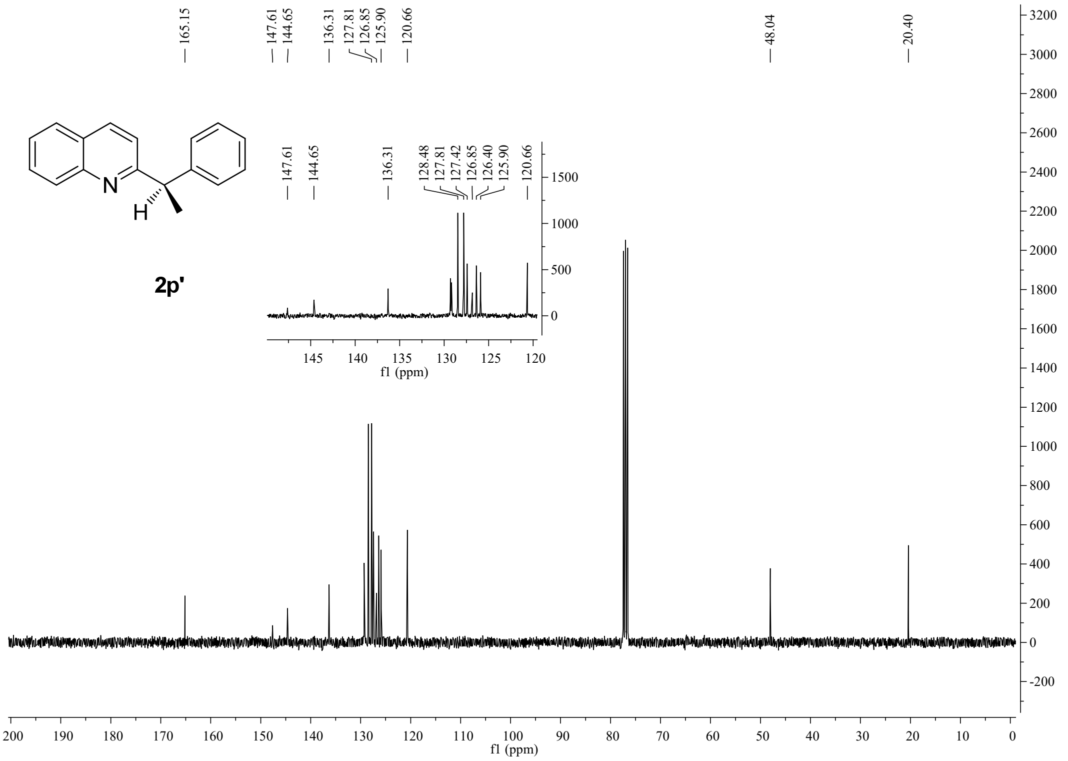

Figure S60. ^{13}C NMR spectrum for **2p'**, related to **Figure 2**.

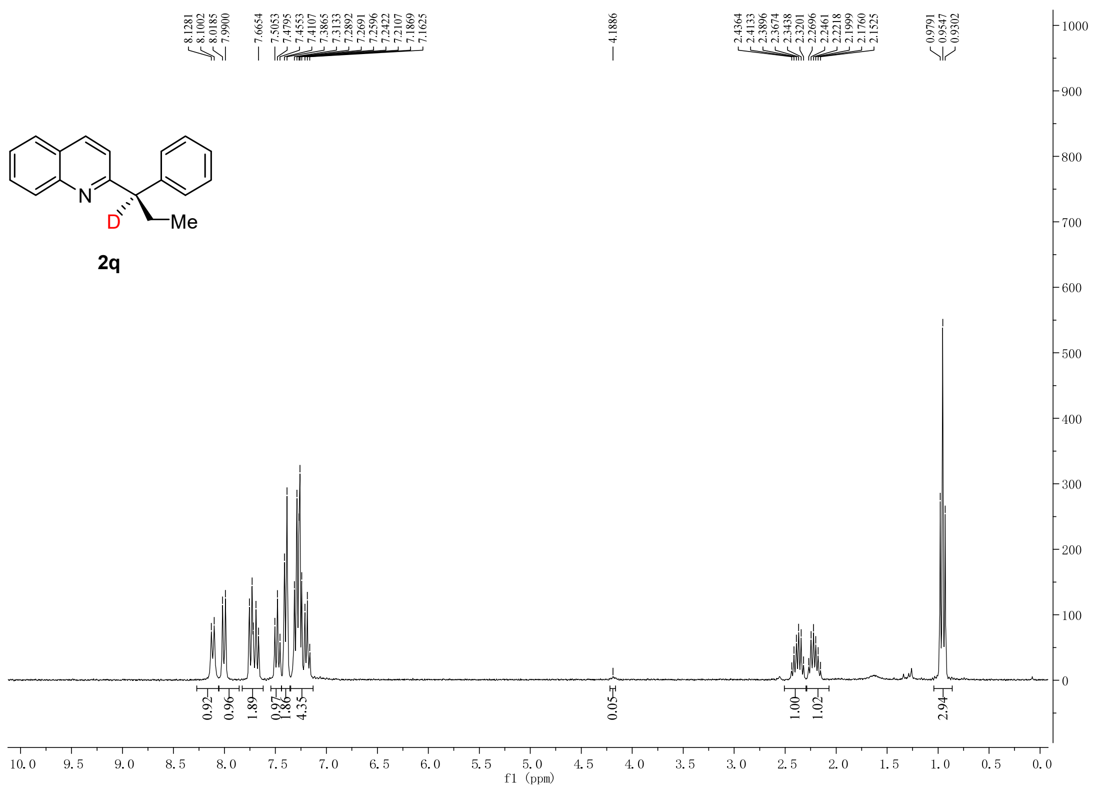

Figure S61. ^{1}H NMR spectrum for **2q**, related to **Figure 2**.

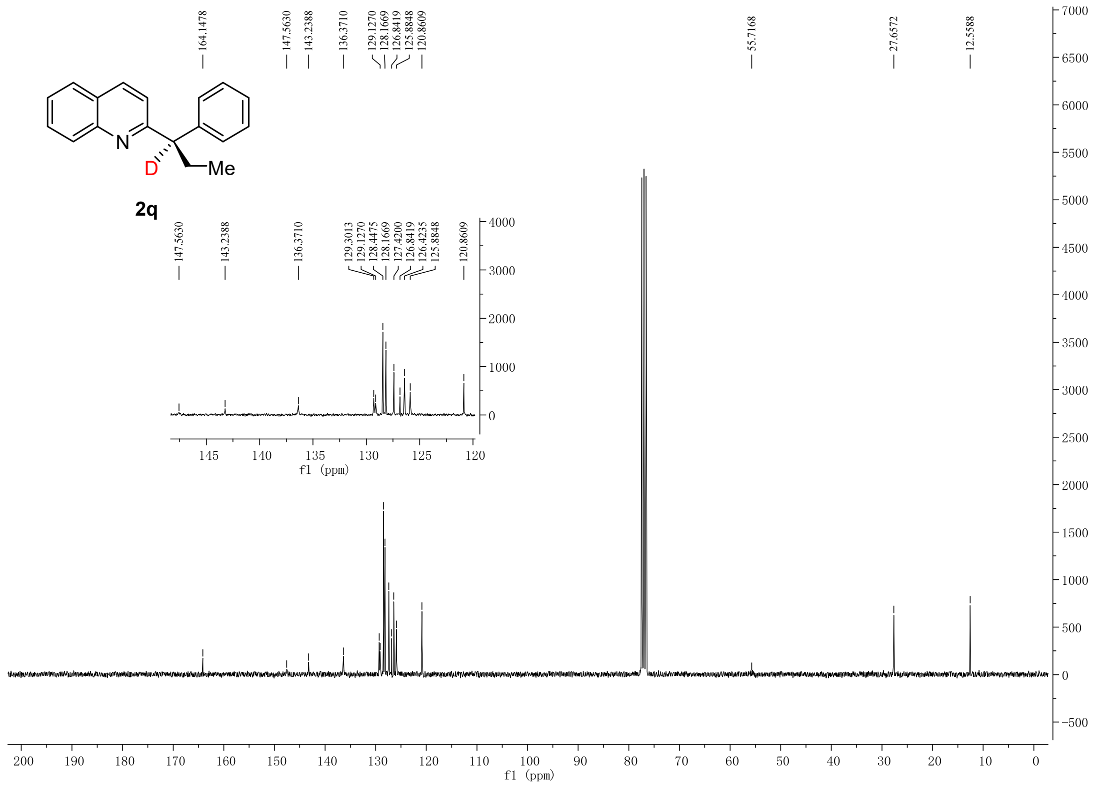

Figure S62. ^{13}C NMR spectrum for **2q**, related to **Figure 2**.

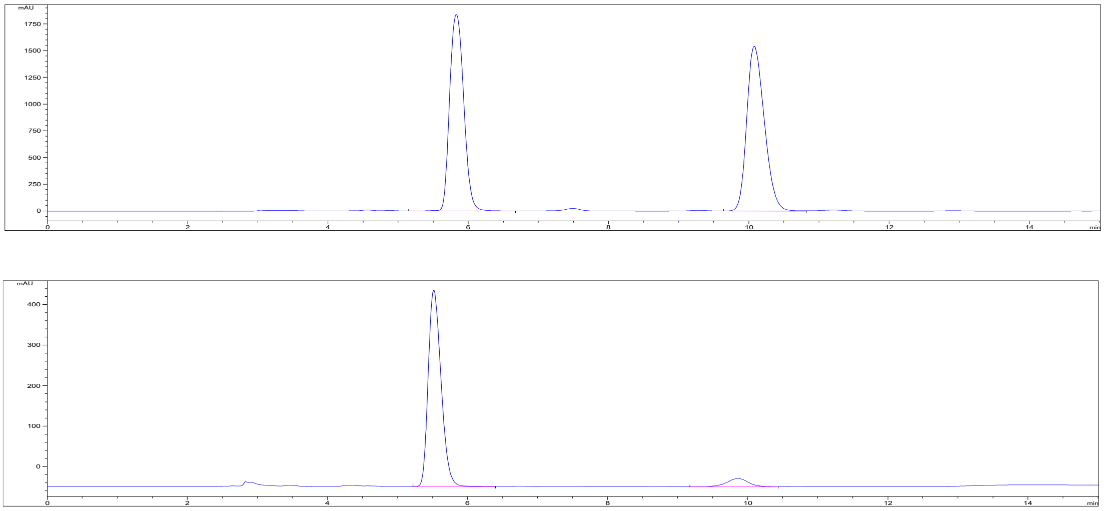

Figure S63. HPLC spectrum for **2q**, related to **Figure 2**.

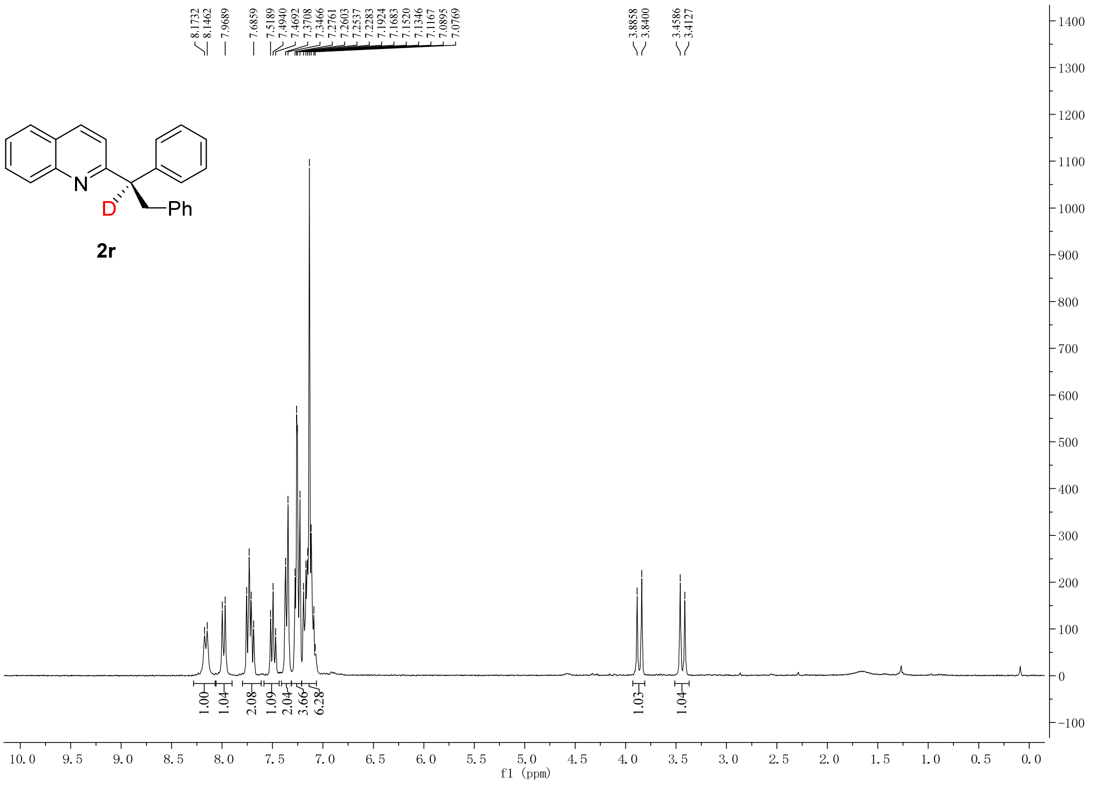

Figure S64. ^{1}H NMR spectrum for **2r**, related to **Figure 2**.

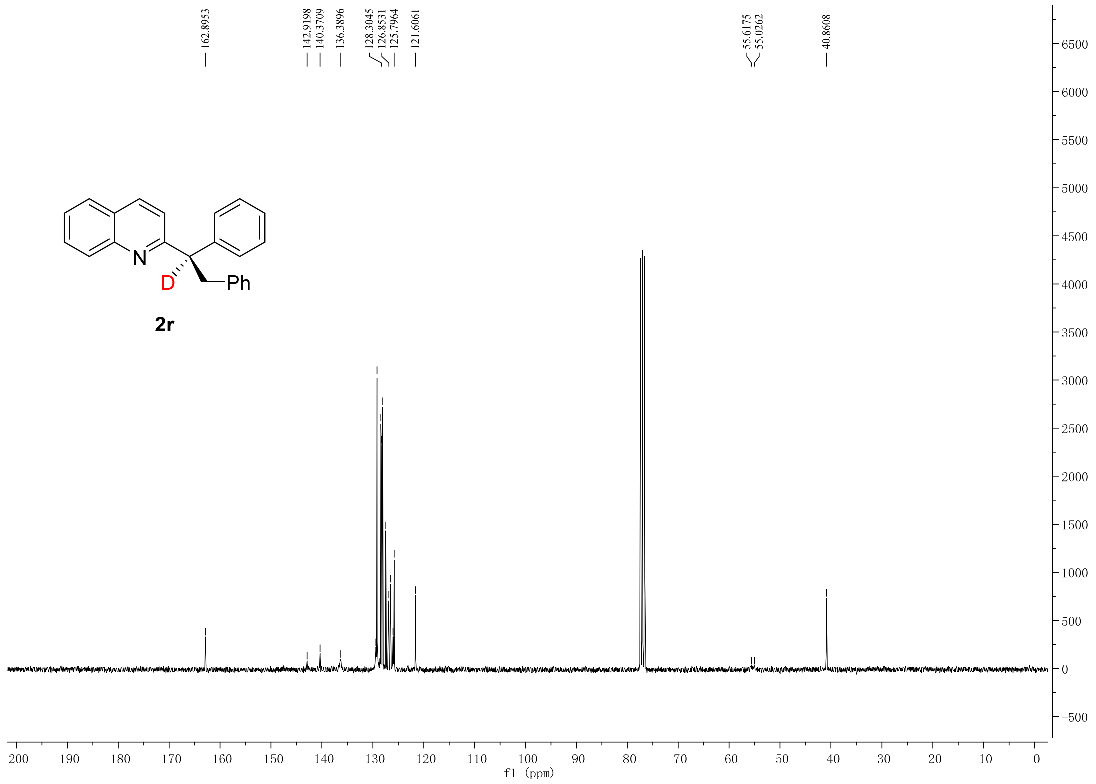

Figure S65. ^{13}C NMR spectrum for **2r**, related to **Figure 2**.

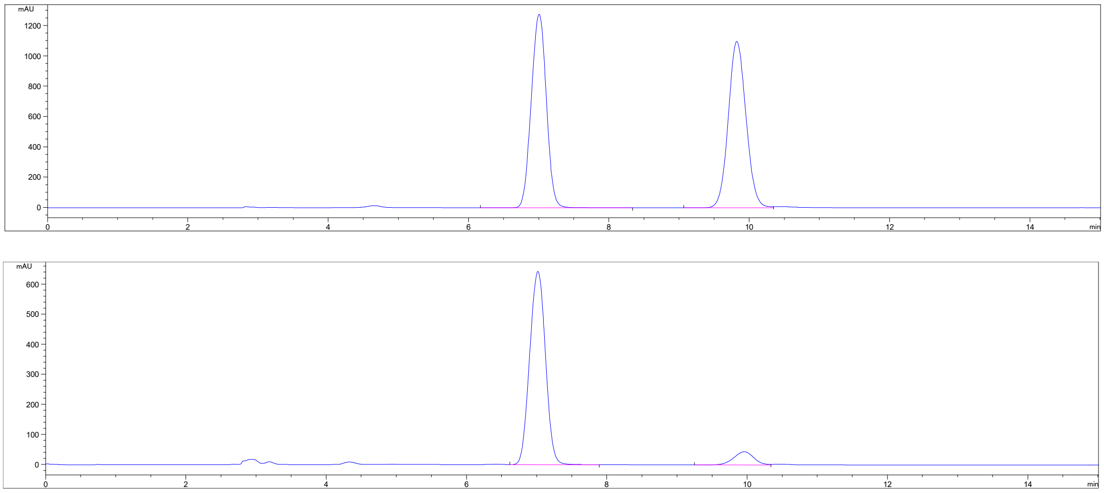

Figure S66. HPLC spectrum for **2r**, related to **Figure 2**.

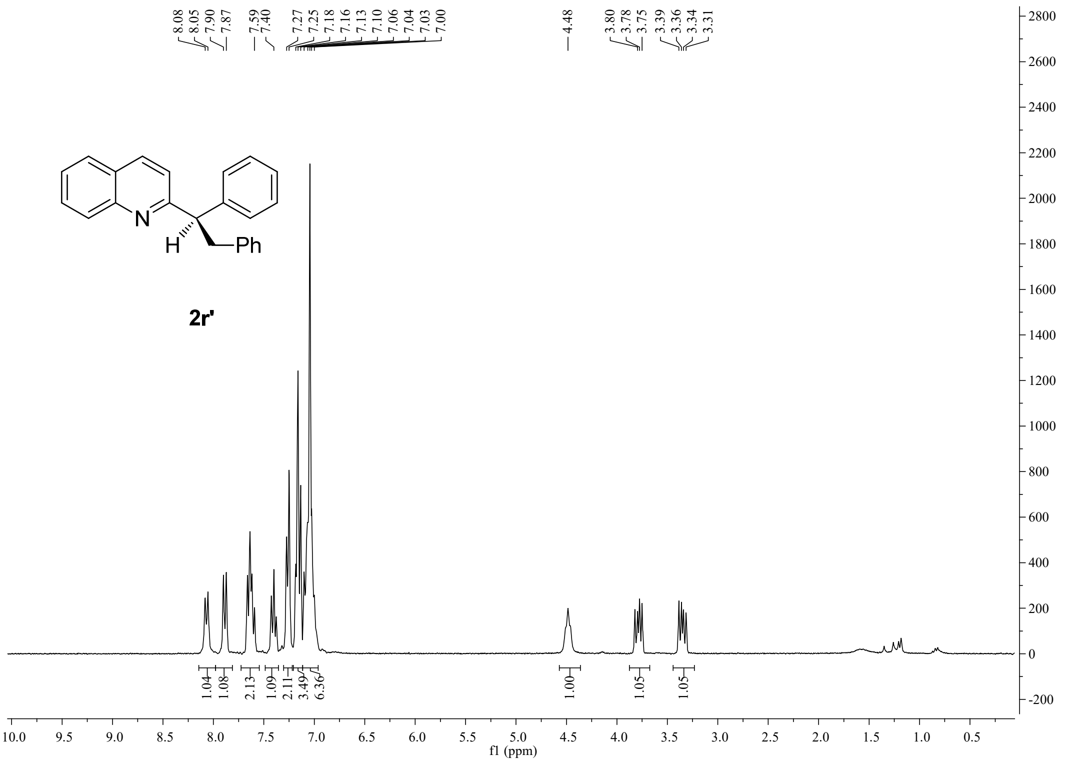

Figure S67. ^{1}H NMR spectrum for **2r’**, related to **Figure 2**.

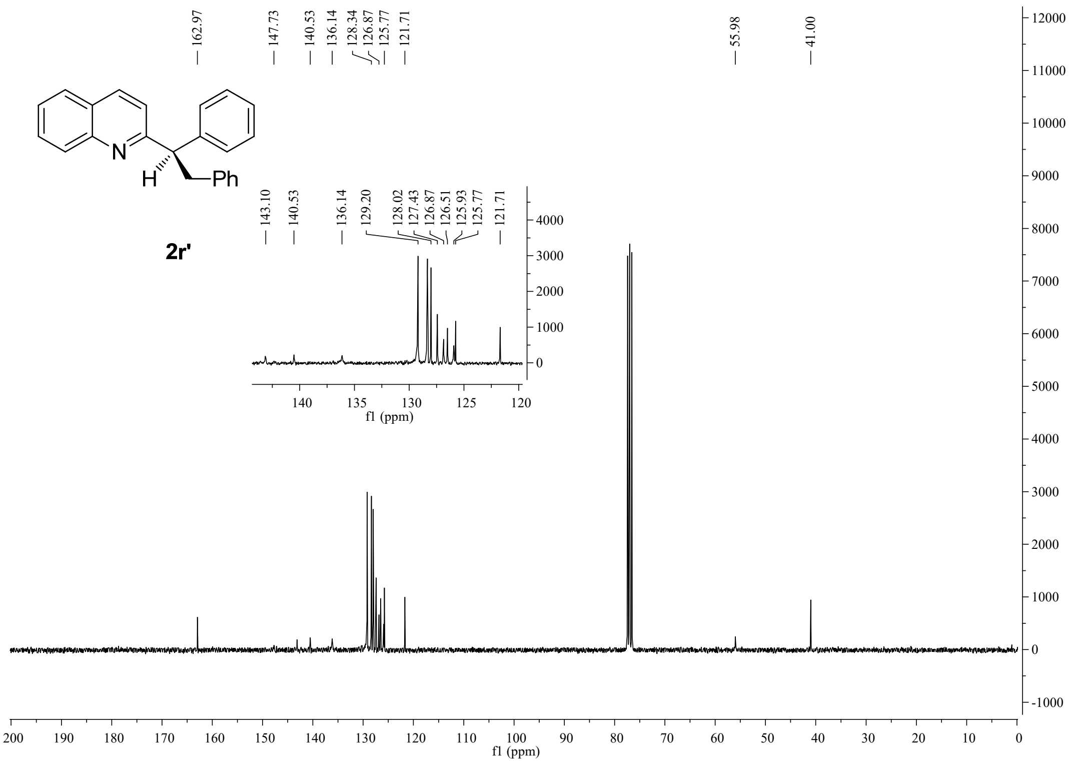

Figure S68. ^{13}C NMR spectrum for **2r’**, related to **Figure 2**.

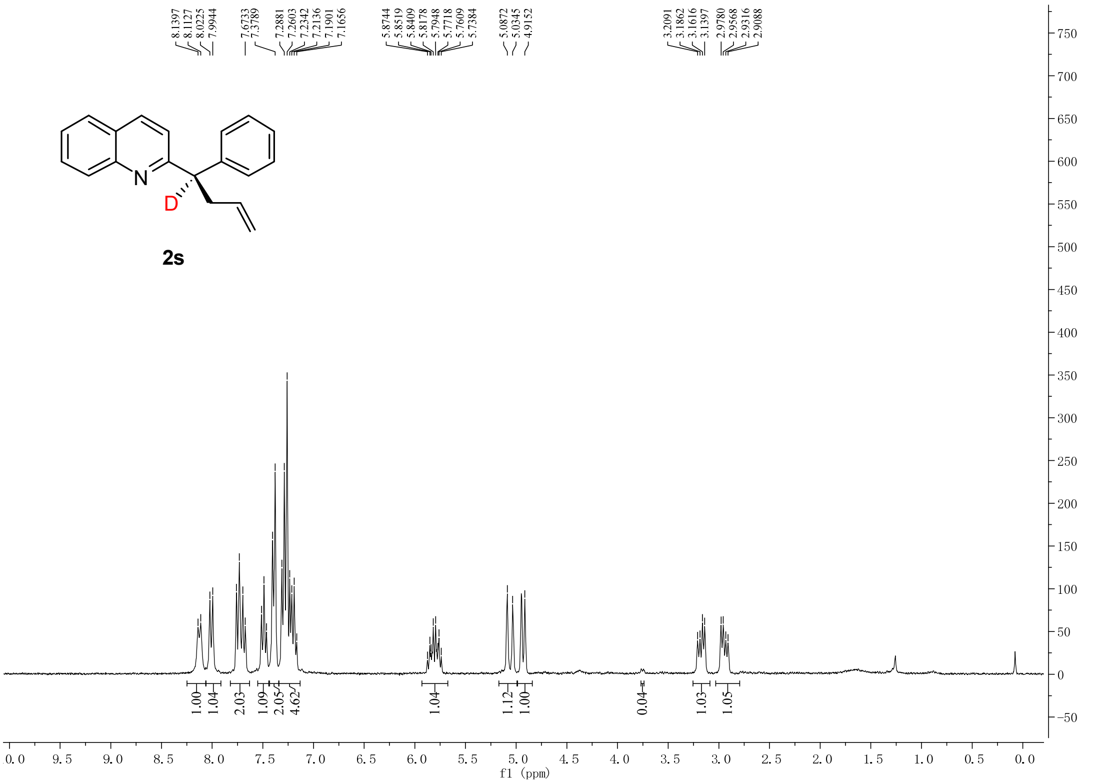

Figure S69. ^{1}H NMR spectrum for **2s**, related to **Figure 2**.

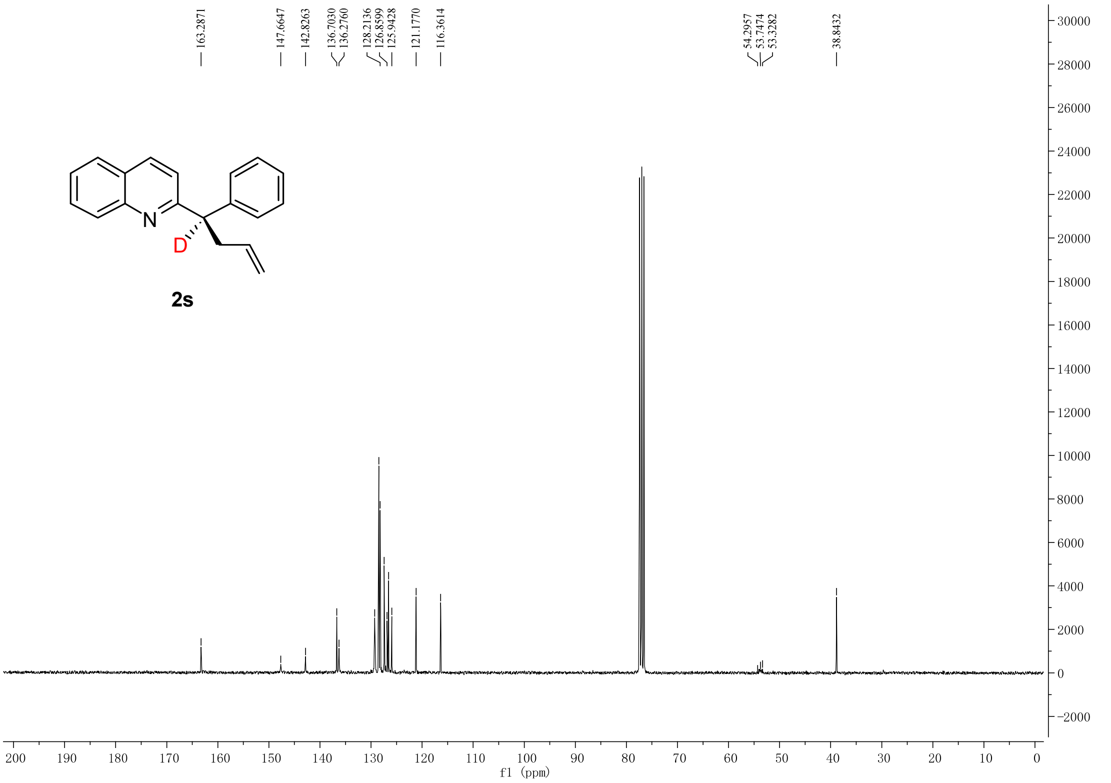

Figure S70. ^{13}C NMR spectrum for **2s**, related to **Figure 2**.

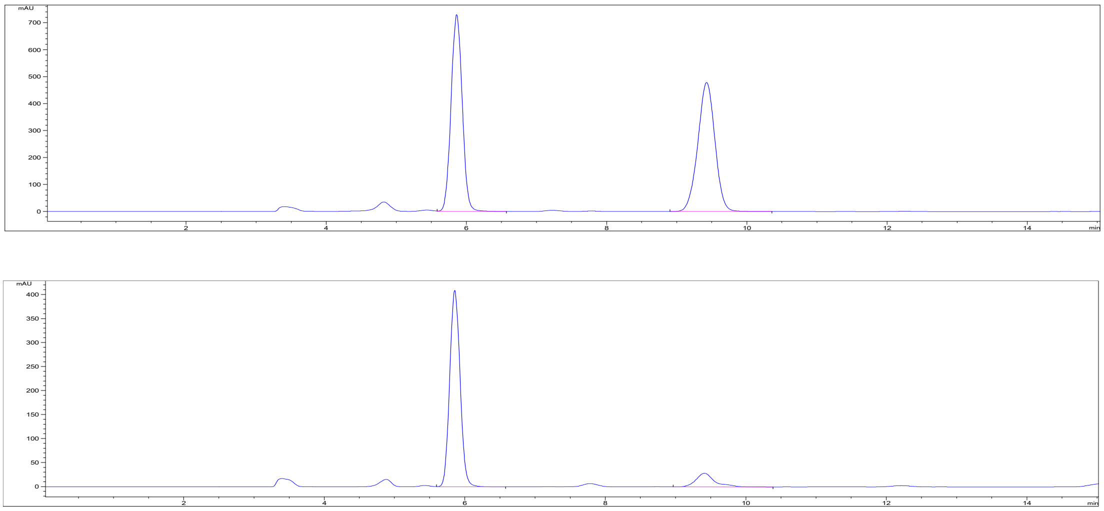

Figure S71. HPLC spectrum for **2s**, related to **Figure 2**.

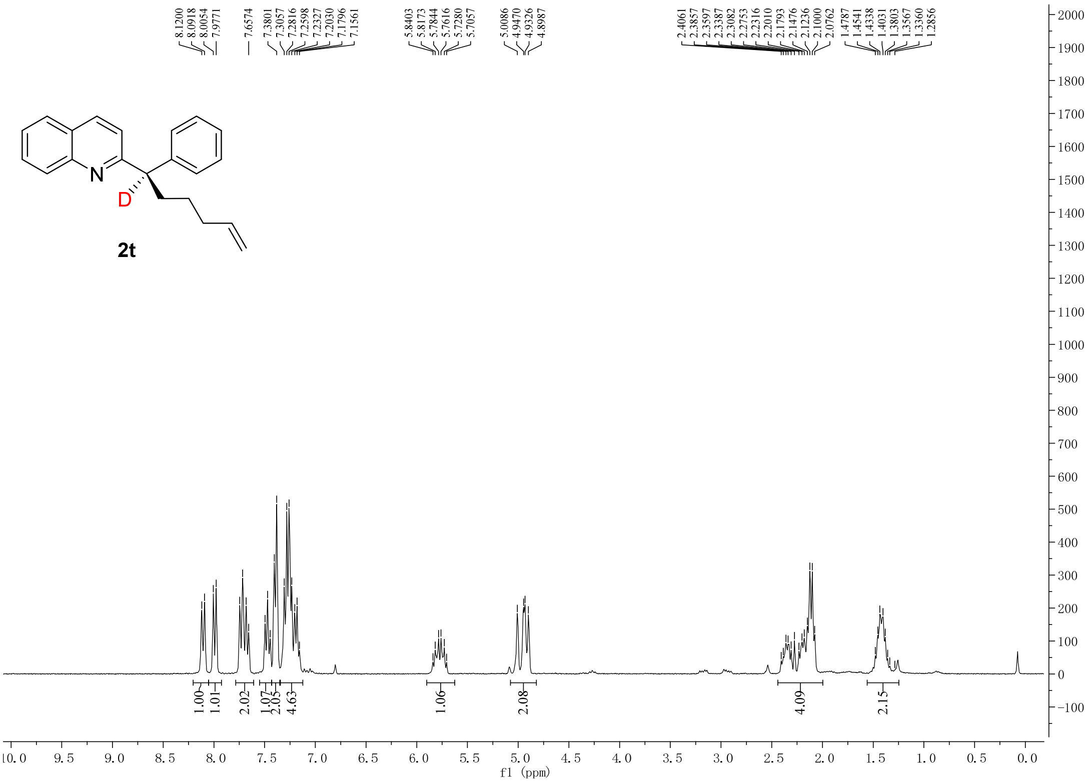

Figure S72. ^{1}H NMR spectrum for **2t**, related to **Figure 2**.

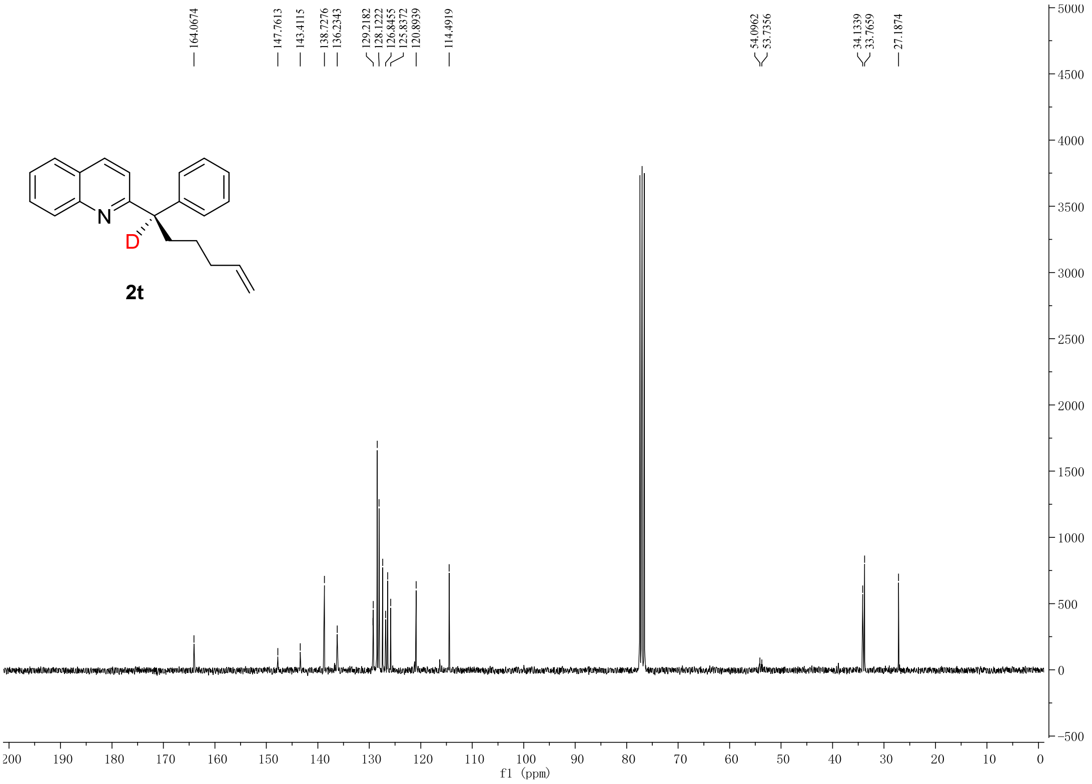

Figure S73. ^{13}C NMR spectrum for **2t**, related to **Figure 2**.

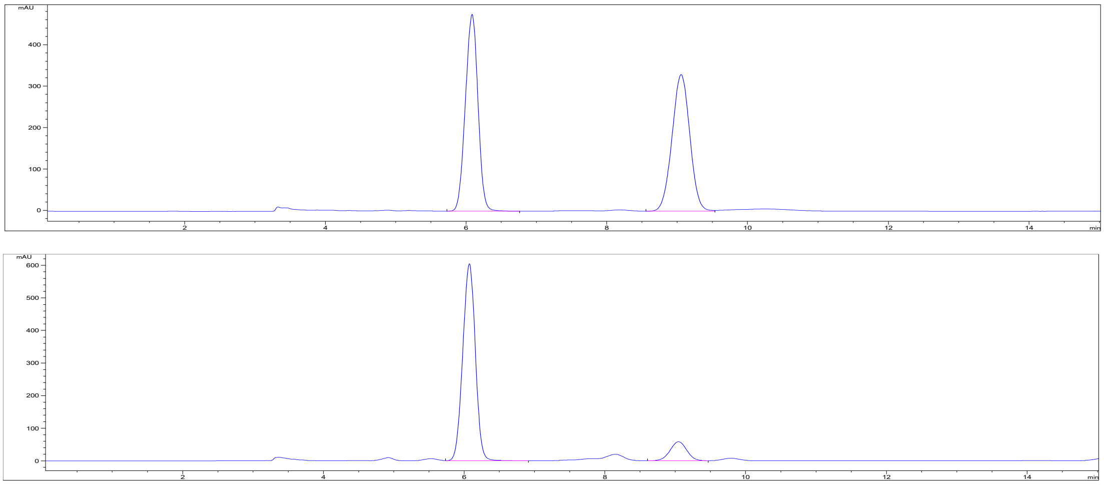

Figure S74. HPLC spectrum for **2t**, related to **Figure 2**.

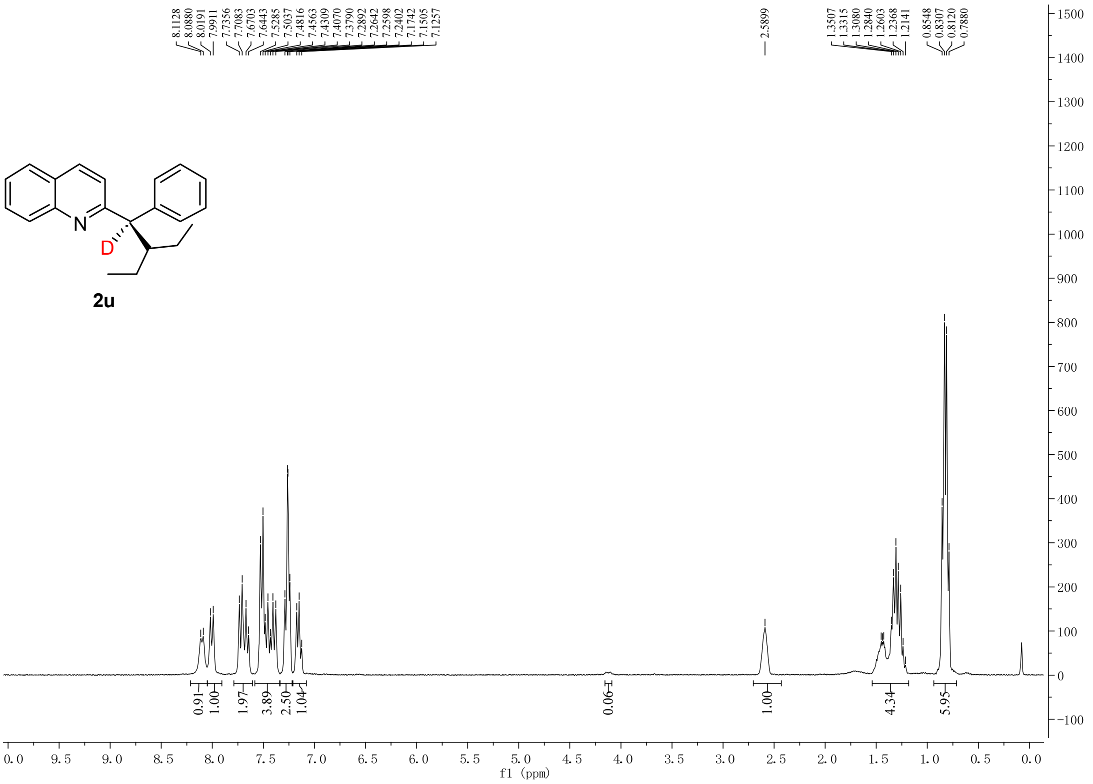

Figure S75. ^{1}H NMR spectrum for **2u**, related to **Figure 2**.

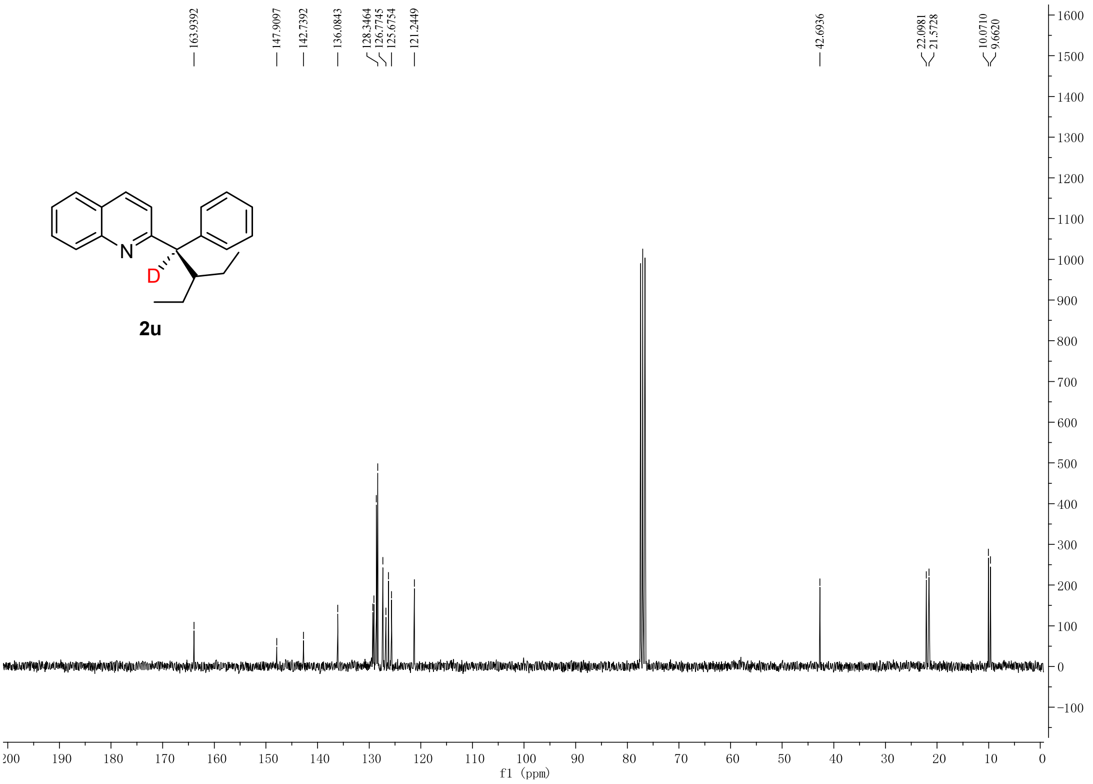

Figure S76. ^{13}C NMR spectrum for **2u**, related to **Figure 2**.

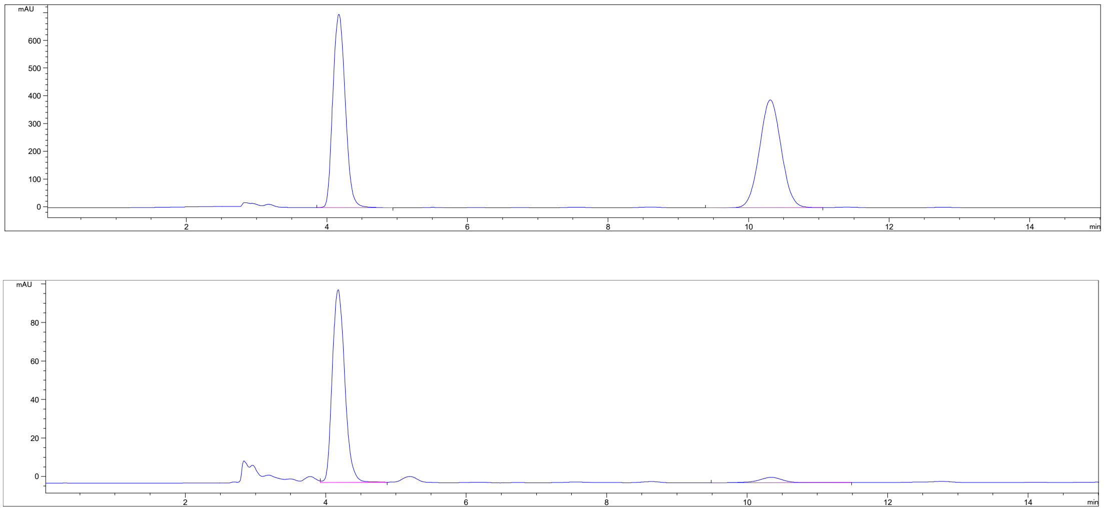

Figure S77. HPLC spectrum for **2u**, related to **Figure 2**.

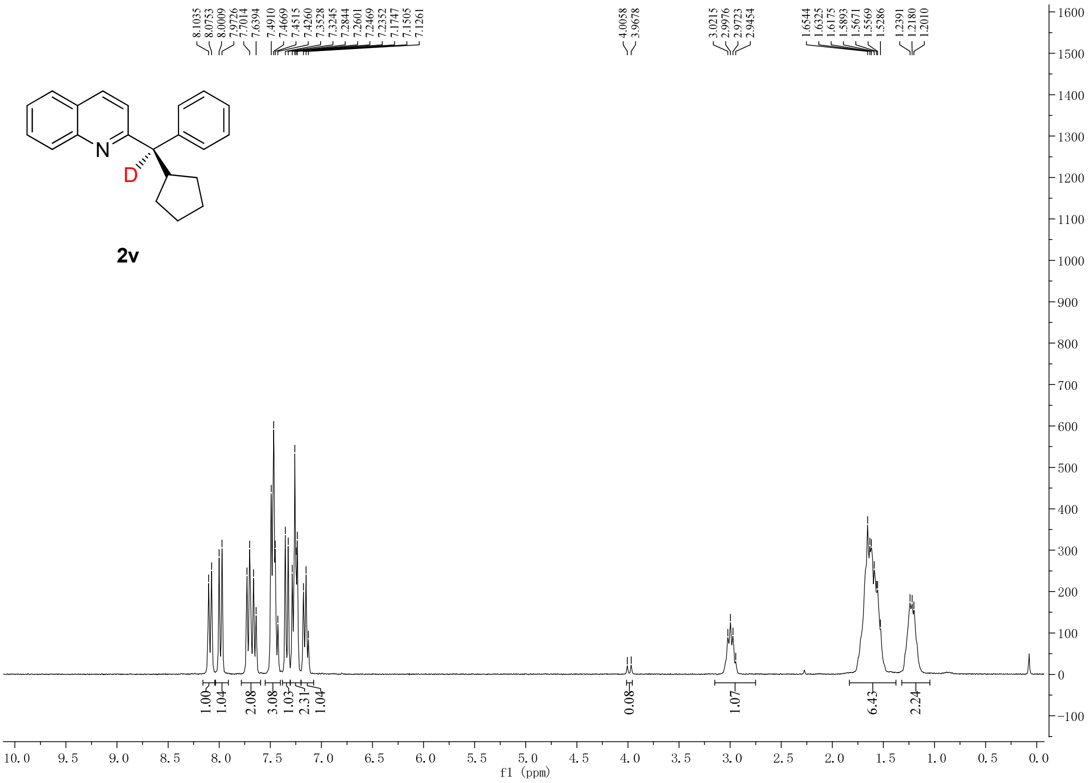

Figure S78. ^{1}H NMR spectrum for **2v**, related to **Figure 2**.

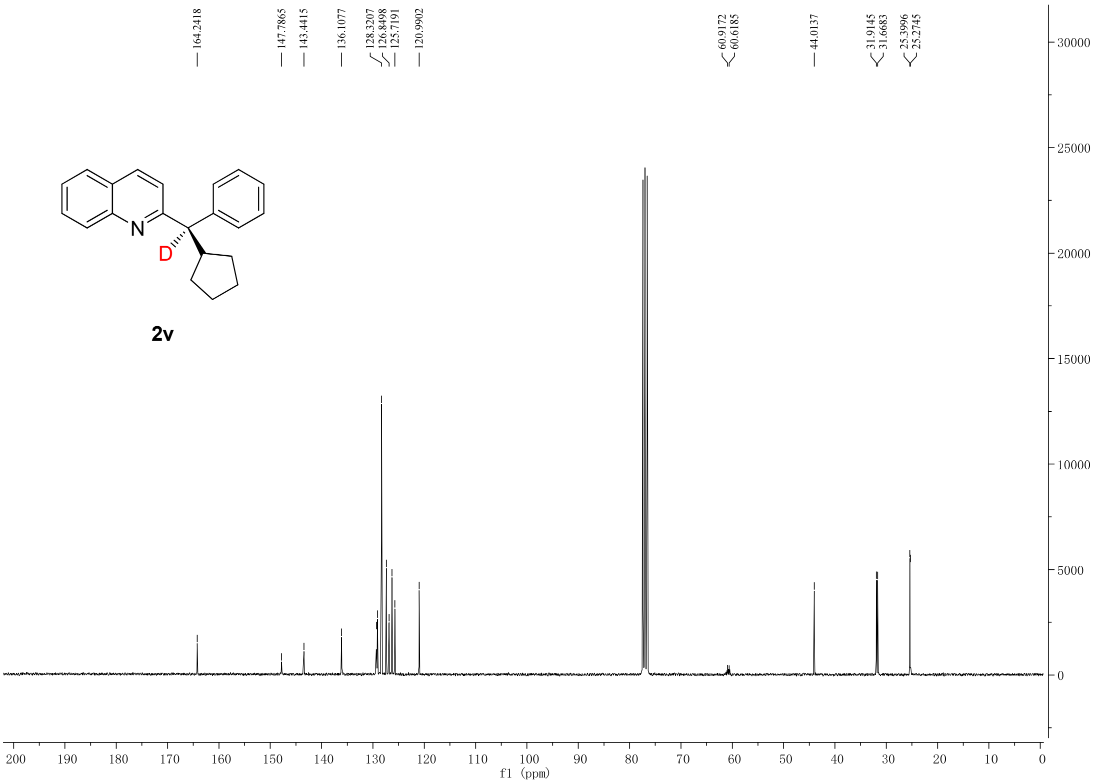

Figure S79. ^{13}C NMR spectrum for **2v**, related to **Figure 2**.

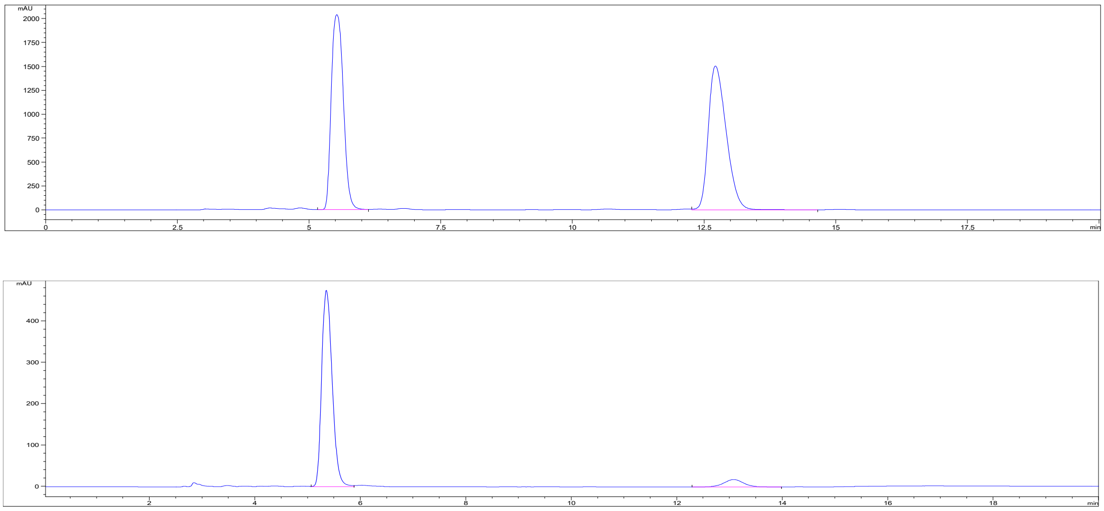

Figure S80. HPLC spectrum for **2v**, related to **Figure 2**.

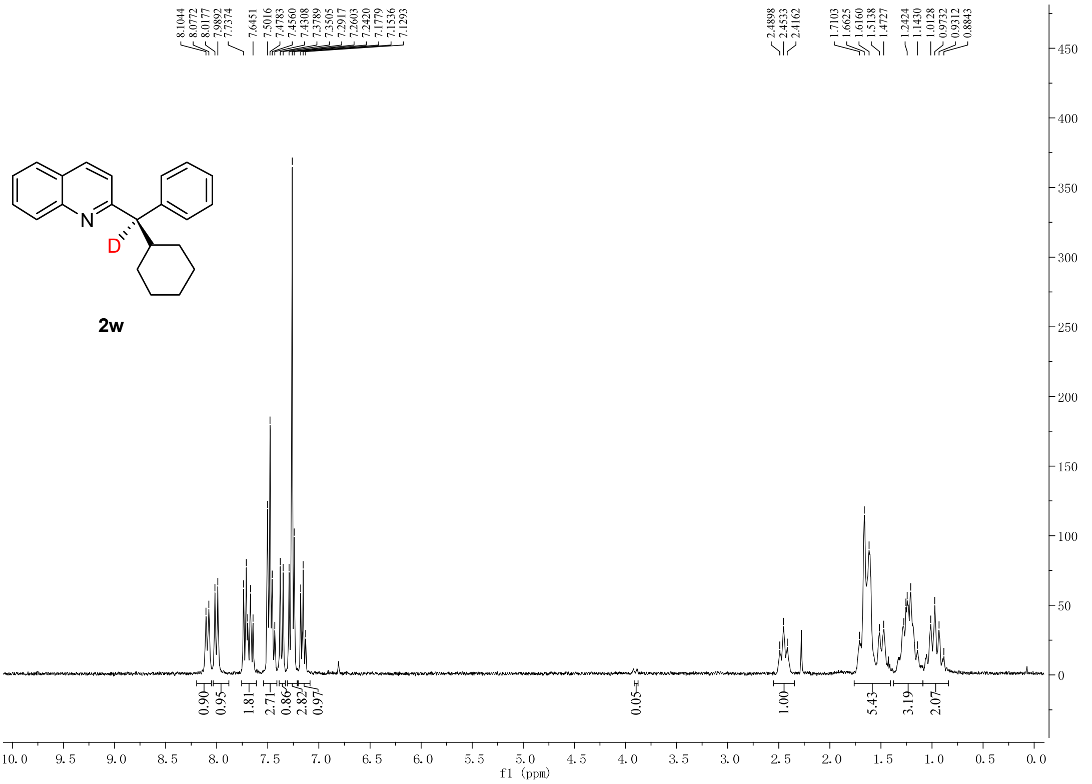

Figure S81. ^{1}H NMR spectrum for **2w**, related to **Figure 2**.

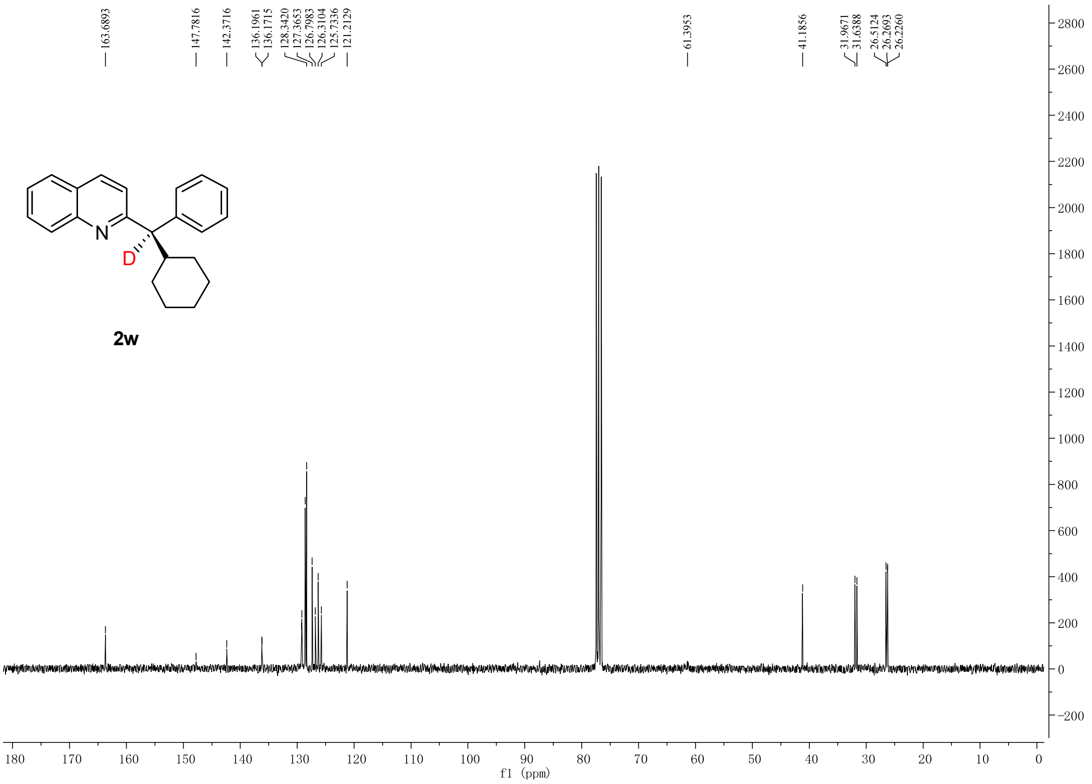

Figure S82. ^{13}C NMR spectrum for **2w**, related to **Figure 2**.

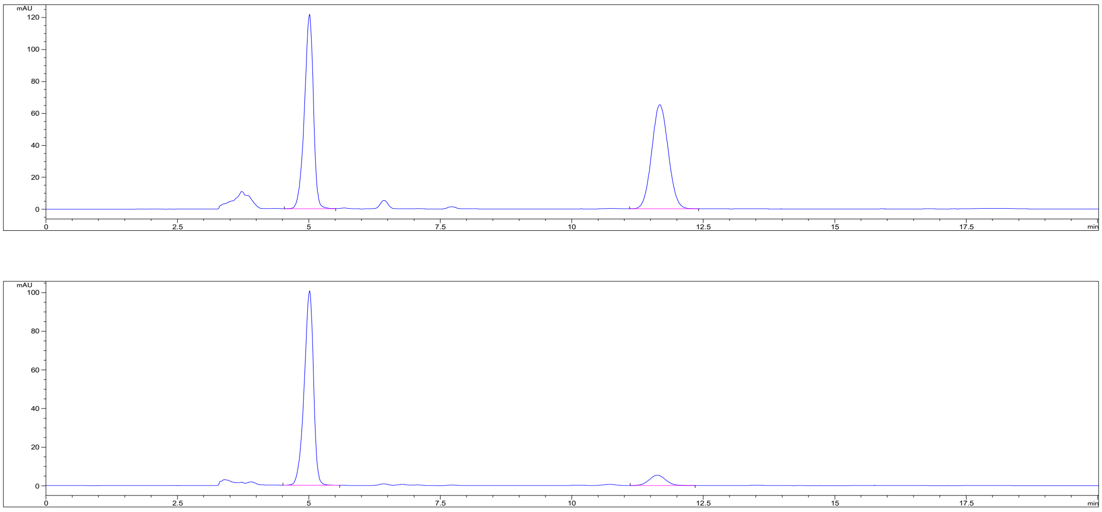

Figure S83. HPLC spectrum for **2w**, related to **Figure 2**.

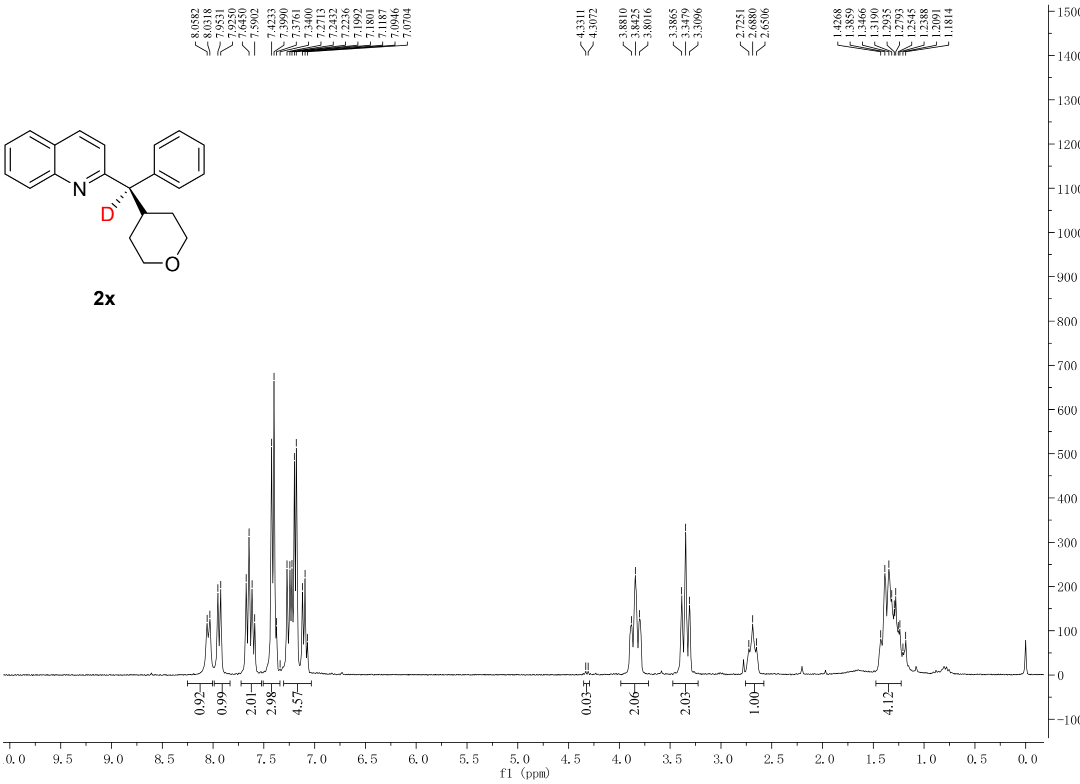

Figure S84. ^{1}H NMR spectrum for **2x**, related to **Figure 2**.

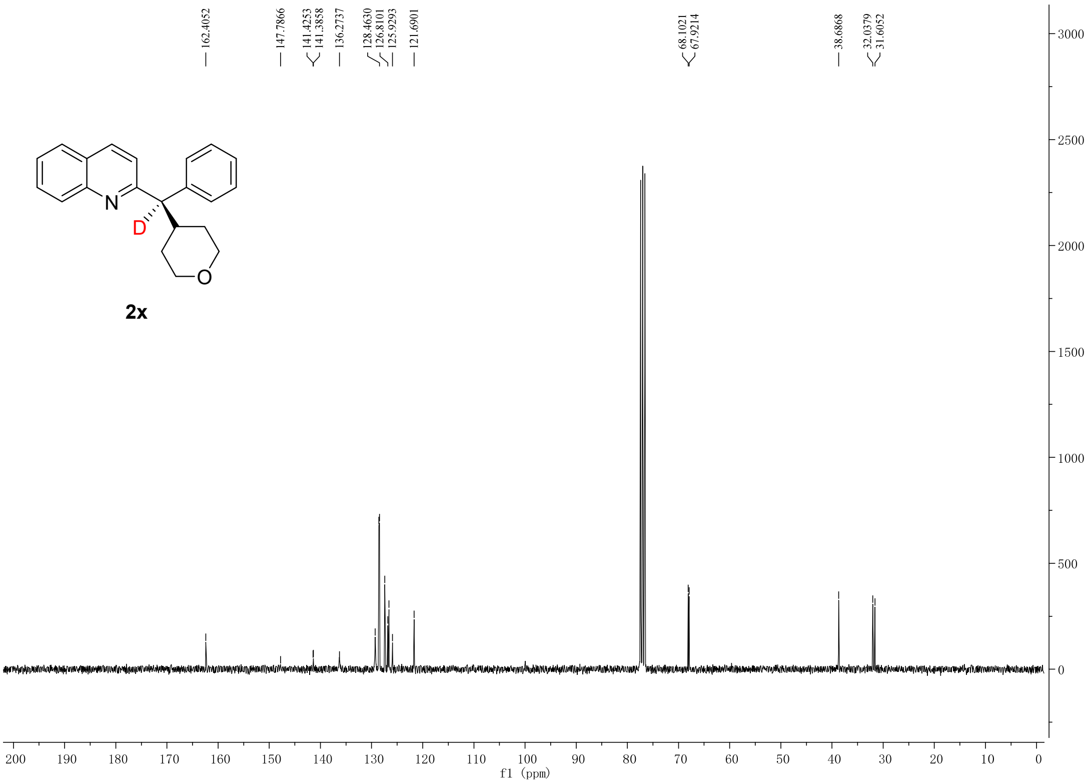

Figure S85. ^{13}C NMR spectrum for **2x**, related to **Figure 2**.

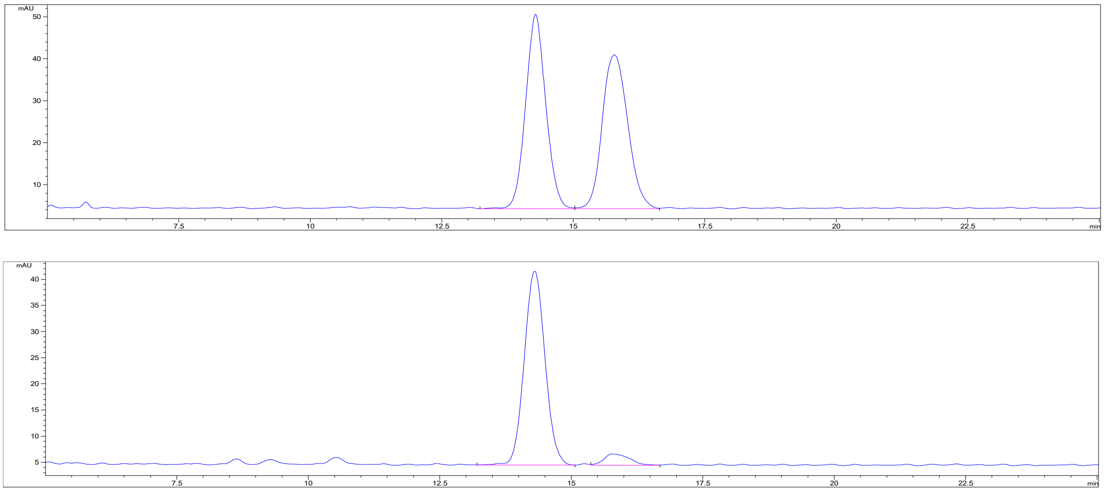

Figure S86. HPLC spectrum for **2x**, related to **Figure 2**.

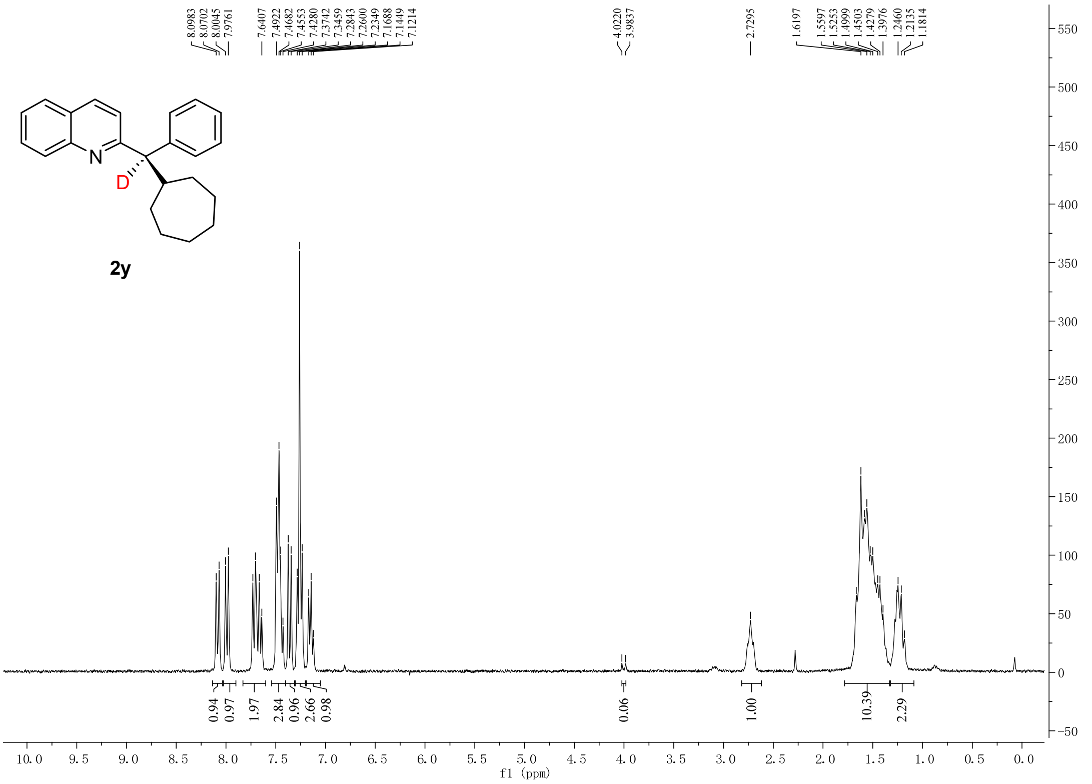

Figure S87. ^{1}H NMR spectrum for **2y**, related to **Figure 2**.

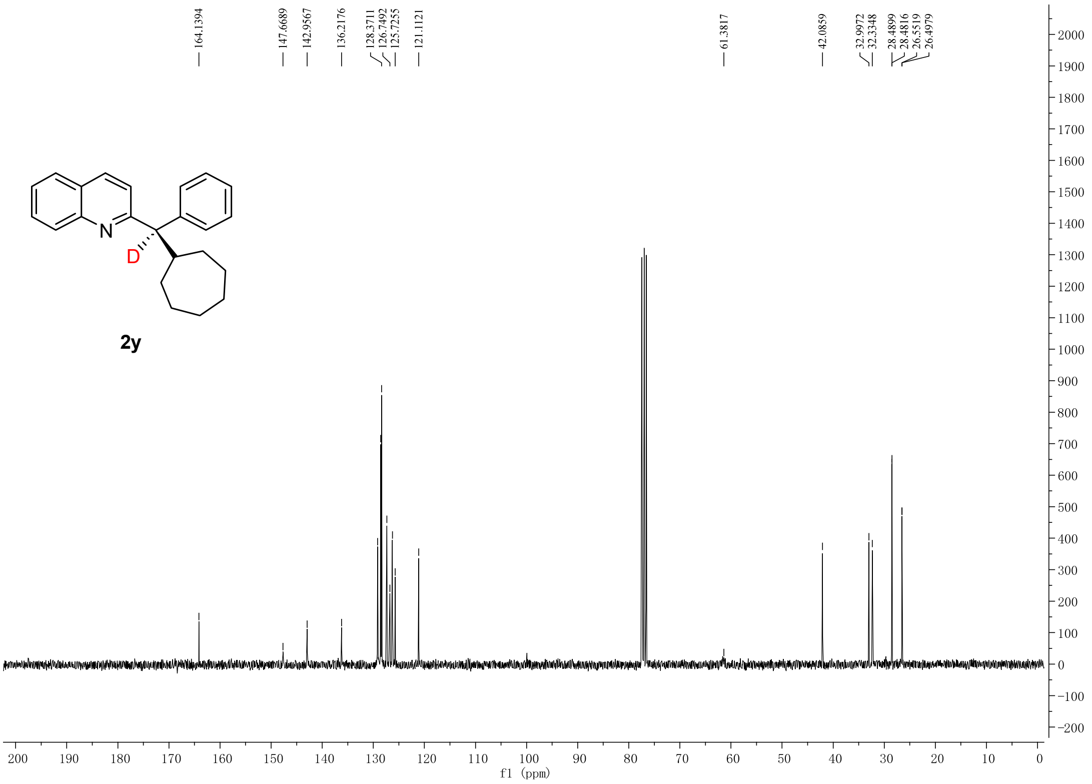

Figure S88. ^{13}C NMR spectrum for **2y**, related to **Figure 2**.

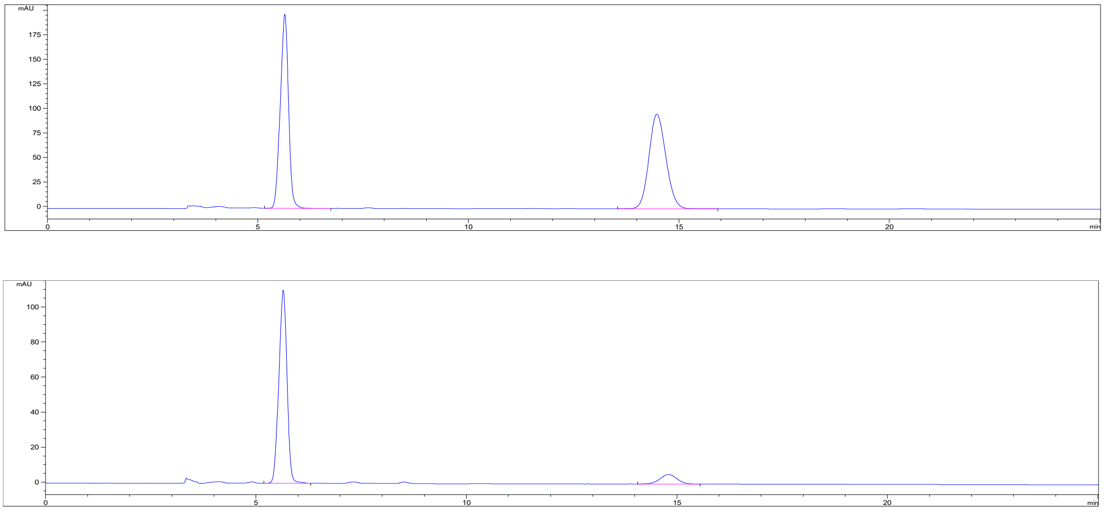

Figure S89. HPLC spectrum for **2y**, related to **Figure 2**.

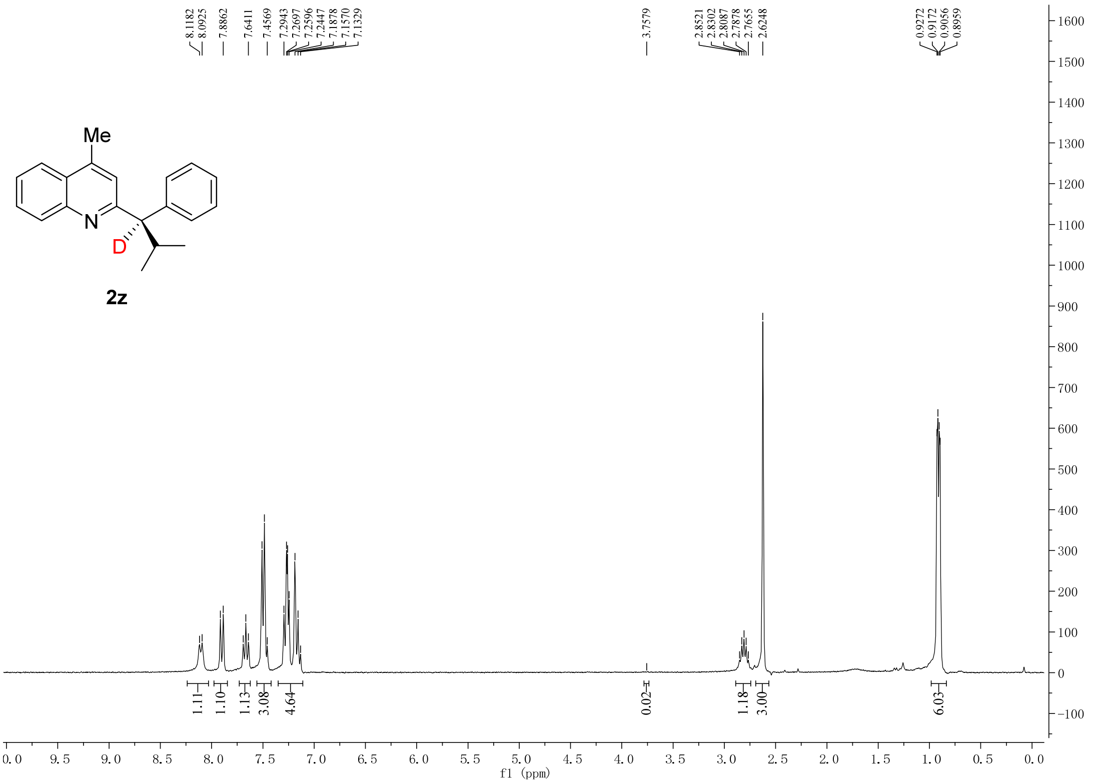

Figure S90. ^{1}H NMR spectrum for **2z**, related to **Figure 2**.

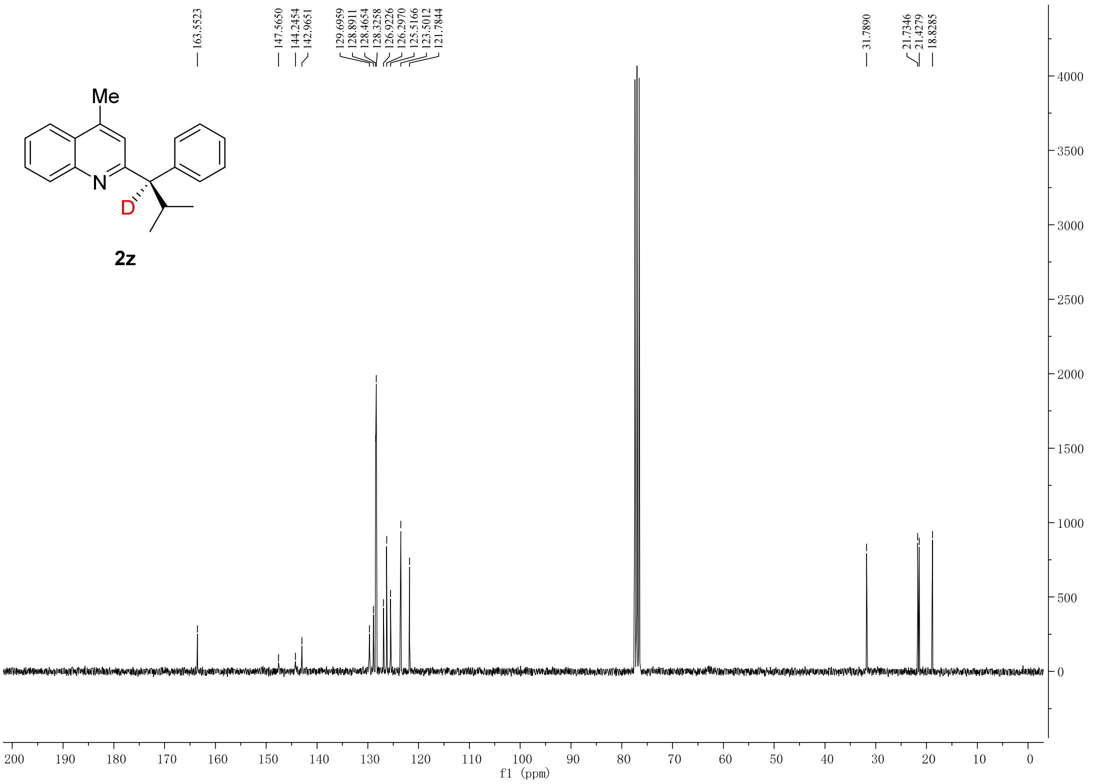

Figure S91. ^{13}C NMR spectrum for **2z**, related to **Figure 2**.

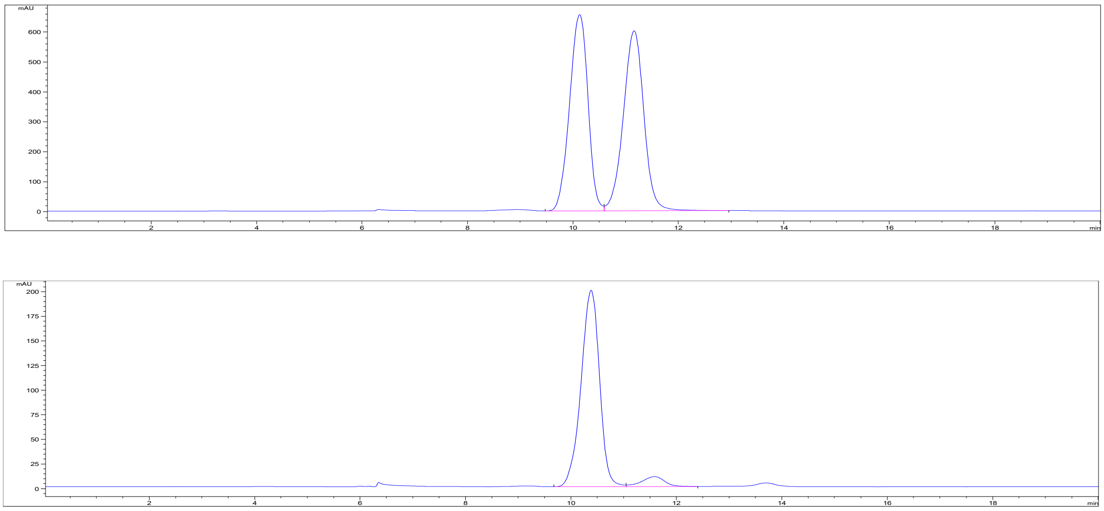

Figure S92. HPLC spectrum for **2z**, related to **Figure 2**.

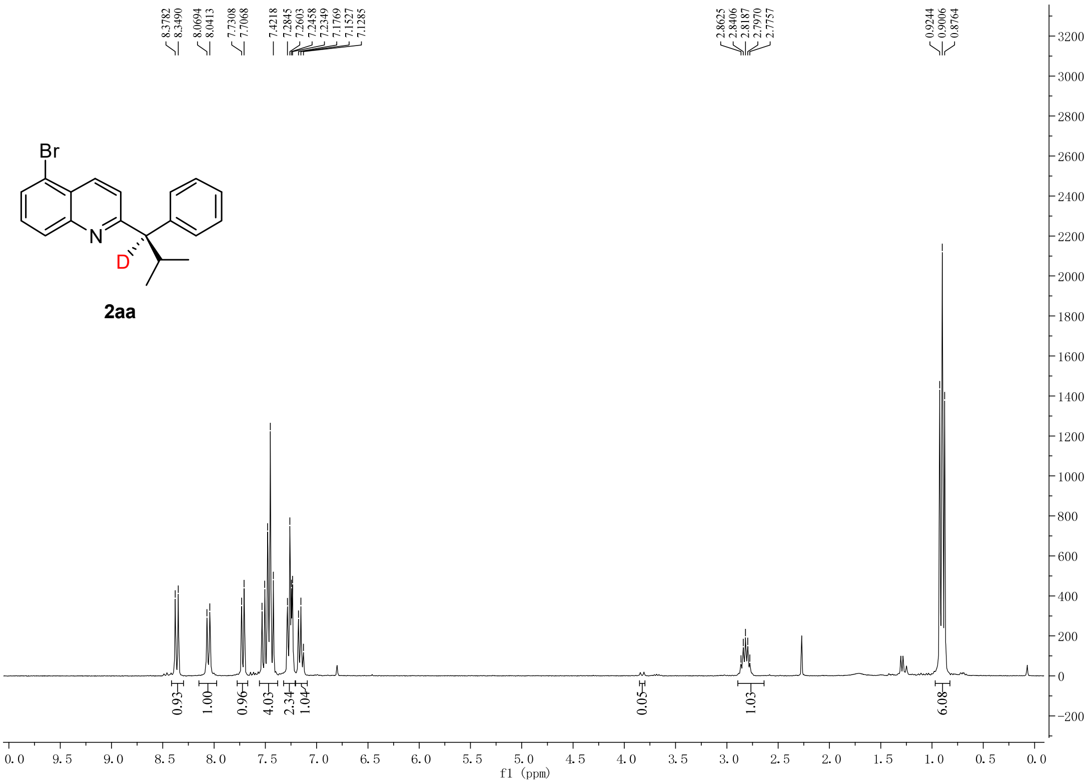

Figure S93. ^{1}H NMR spectrum for **2aa**, related to **Figure 2**.

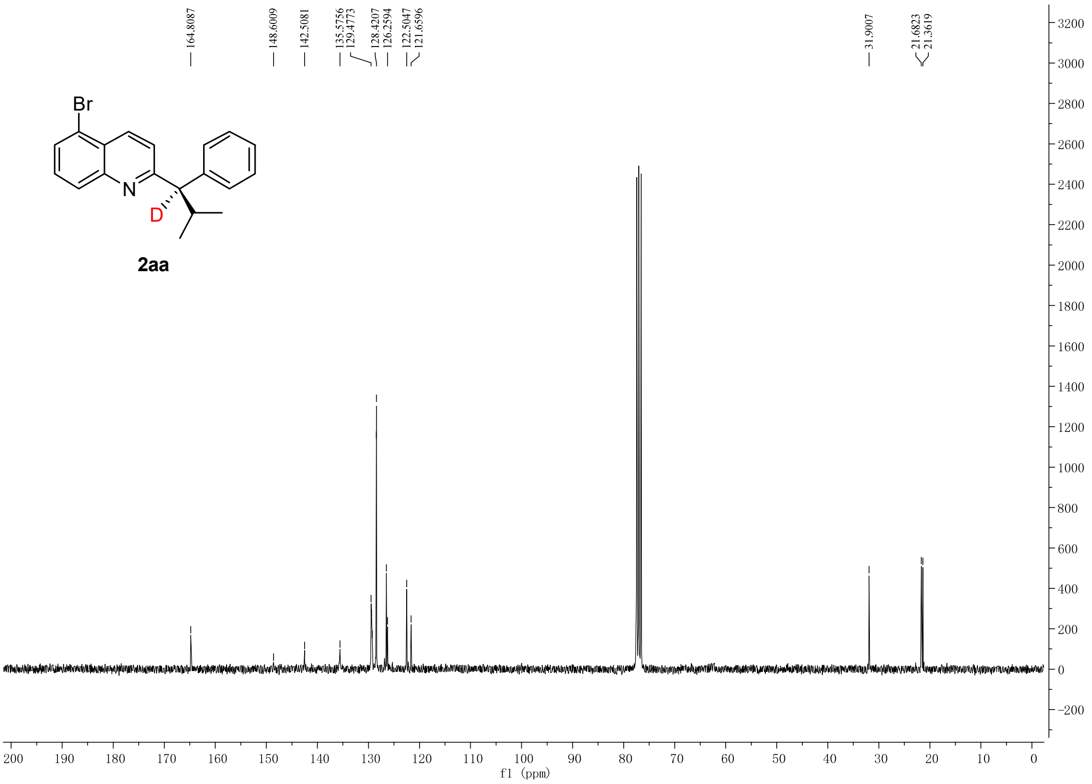

Figure S94. ^{13}C NMR spectrum for **2aa**, related to **Figure 2**.

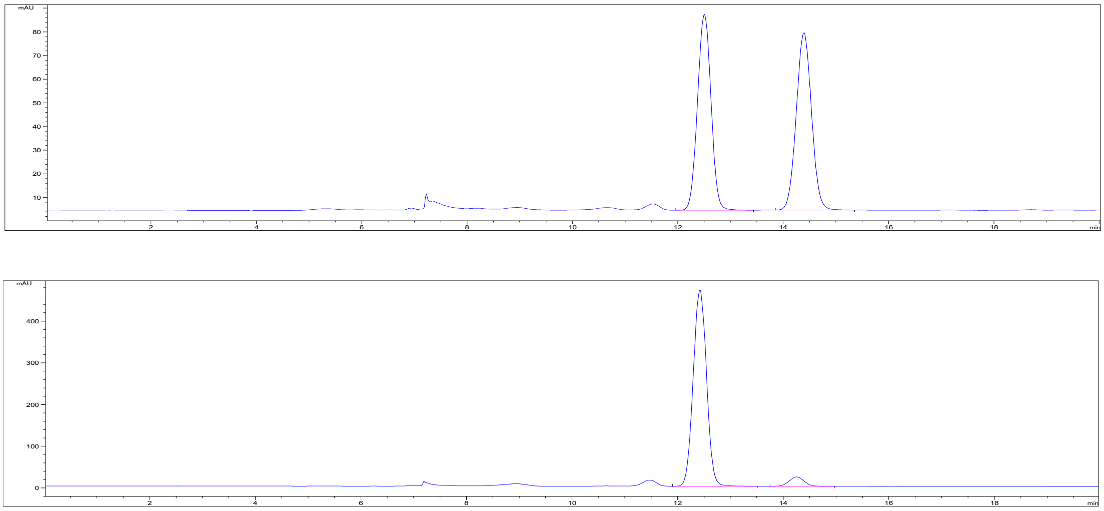

Figure S95. HPLC spectrum for **2aa**, related to **Figure 2**.

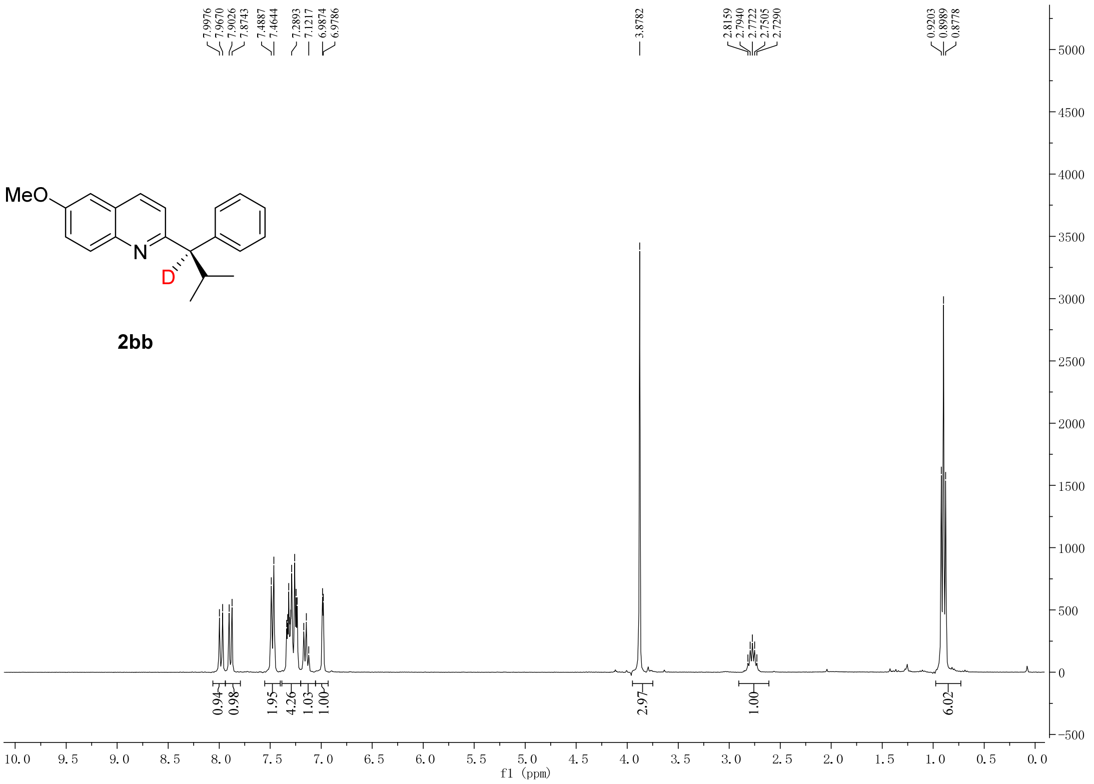

Figure S96. ^{1}H NMR spectrum for **2bb**, related to **Figure 2**.

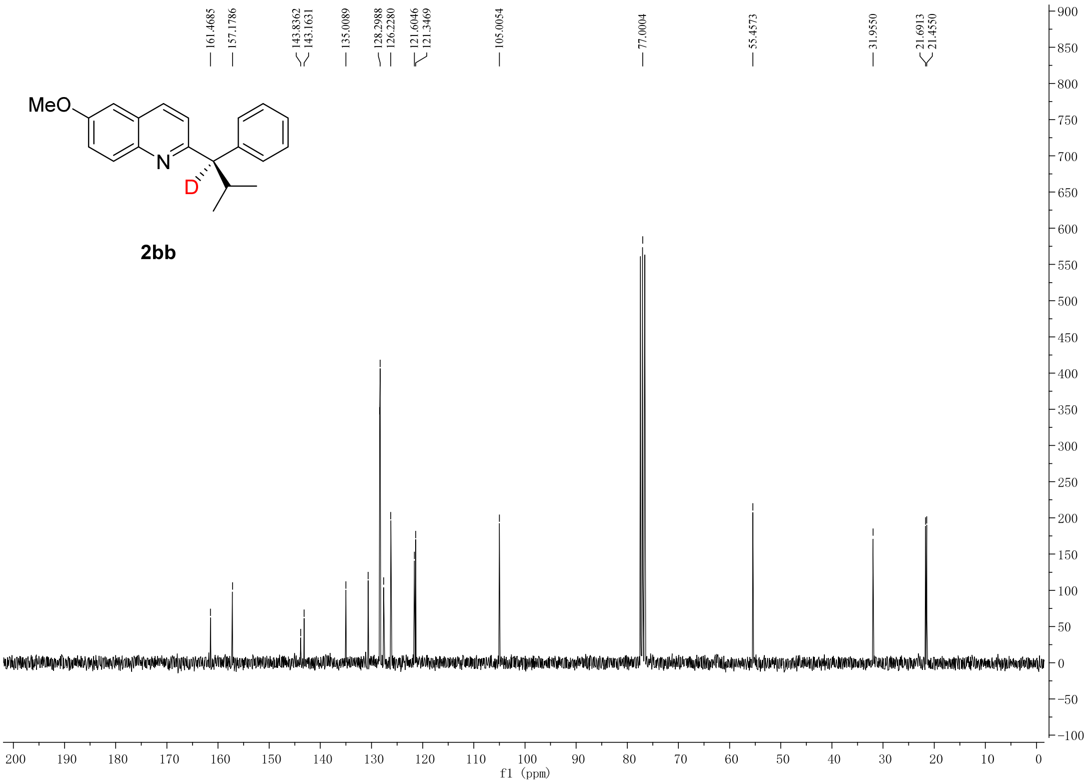

Figure S97. ^{13}C NMR spectrum for **2bb**, related to **Figure 2**.

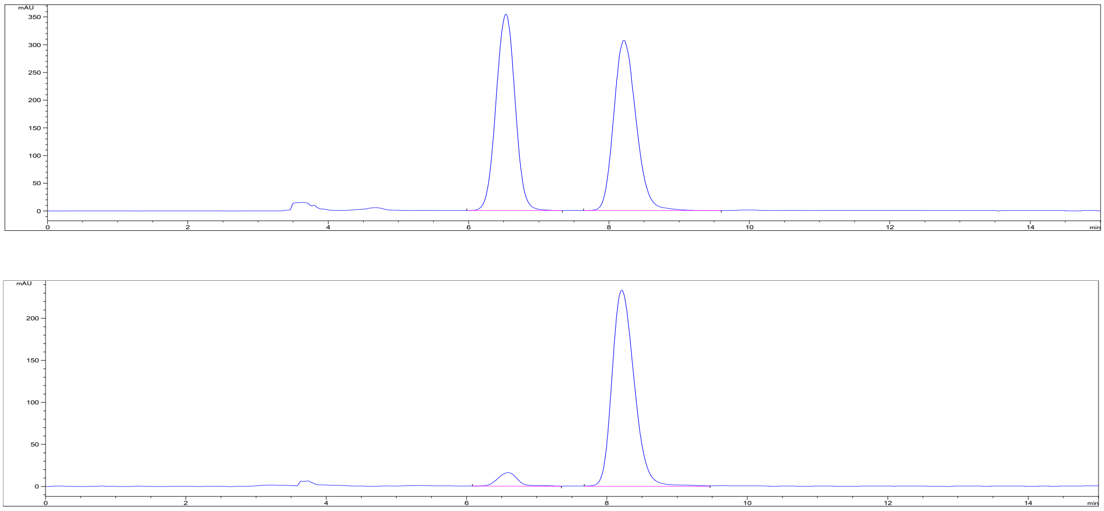

Figure S98. HPLC spectrum for **2bb**, related to **Figure 2**.

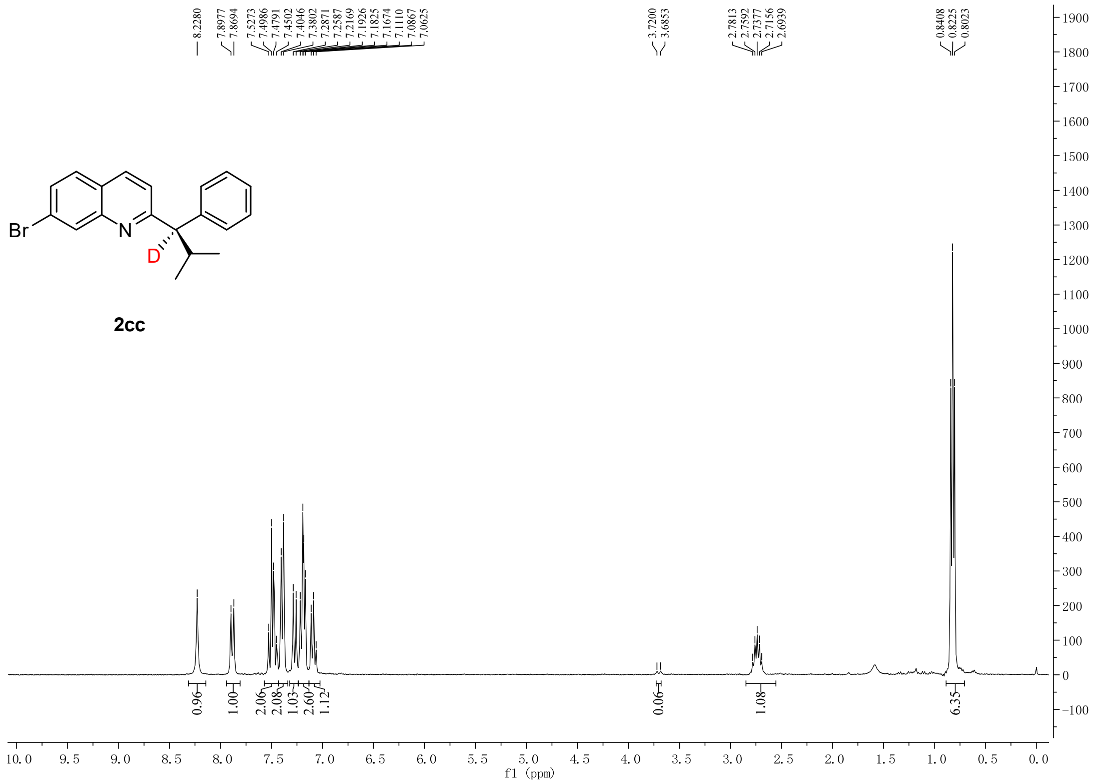

Figure S99. ^{1}H NMR spectrum for **2cc**, related to **Figure 2**.

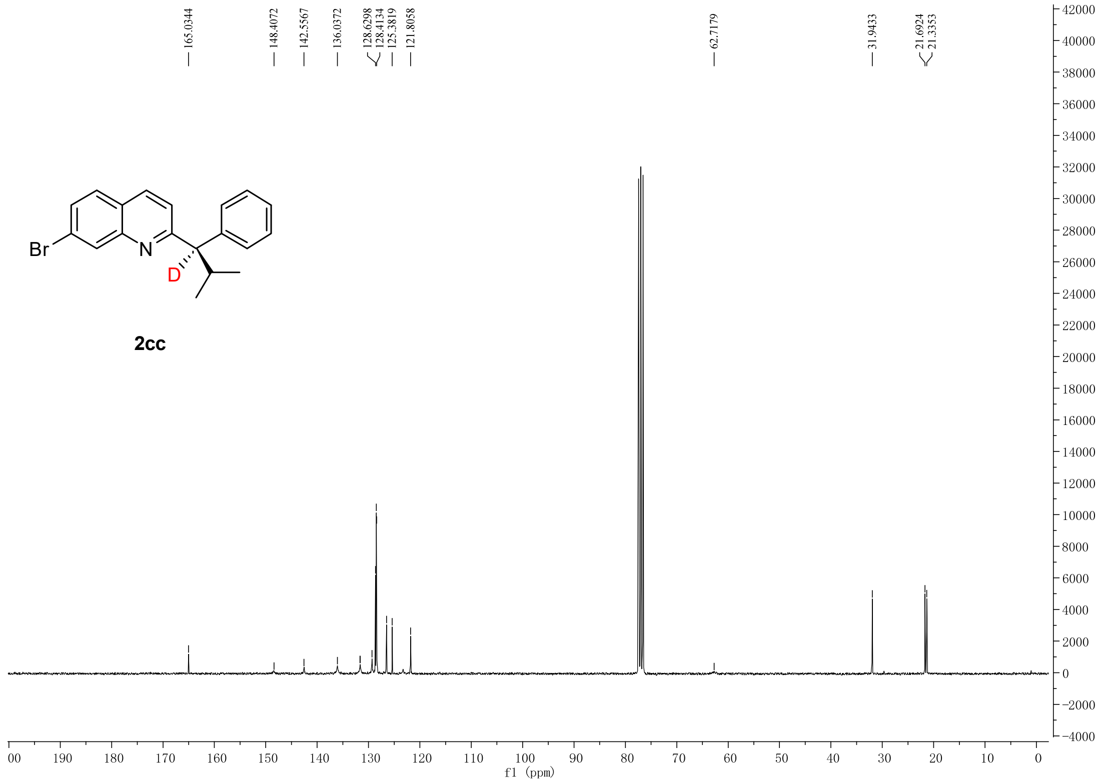

Figure S100. ^{13}C NMR spectrum for **2cc**, related to **Figure 2**.

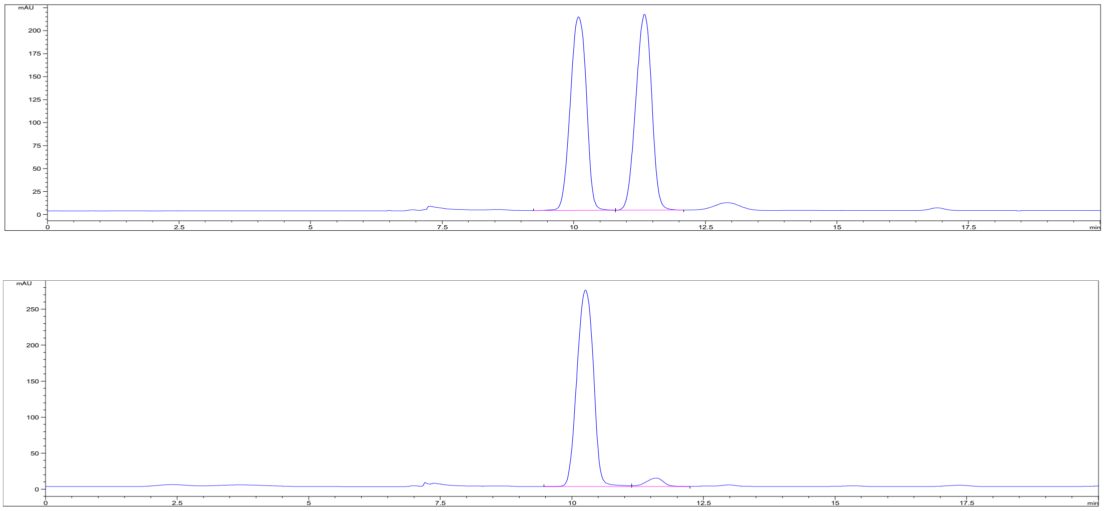

Figure S101. HPLC spectrum for **2cc**, related to **Figure 2**.

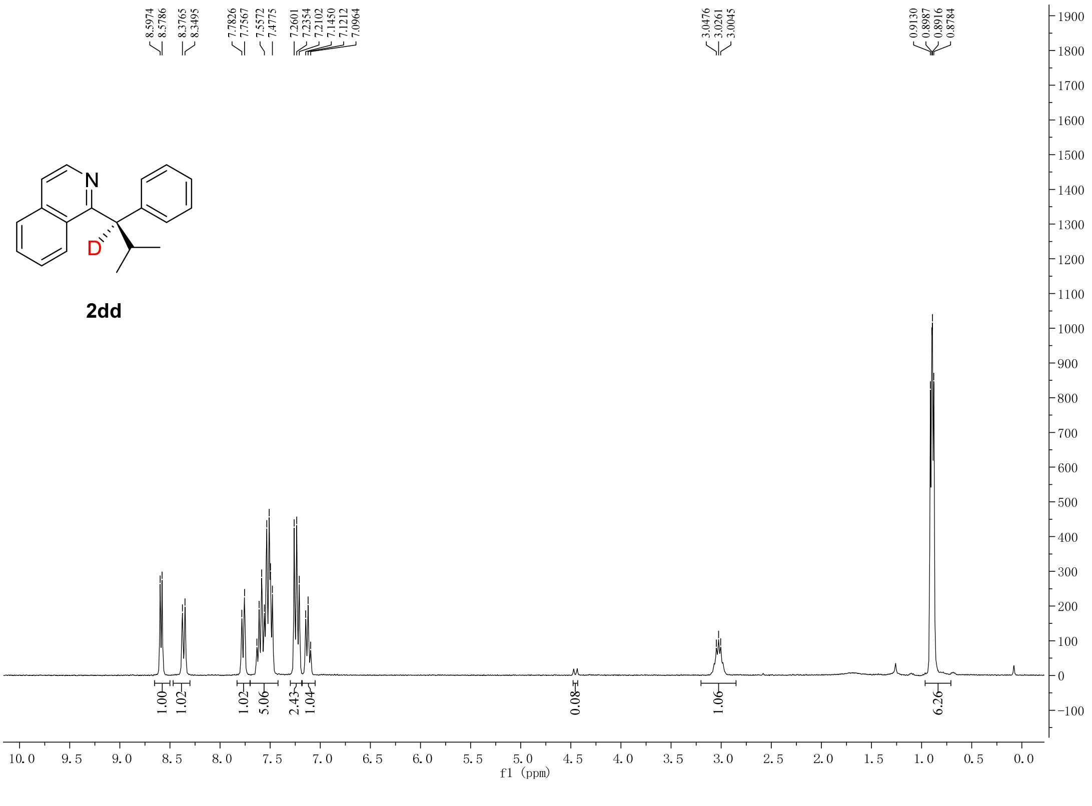

Figure S102. ^{1}H NMR spectrum for **2dd**, related to **Figure 2**.

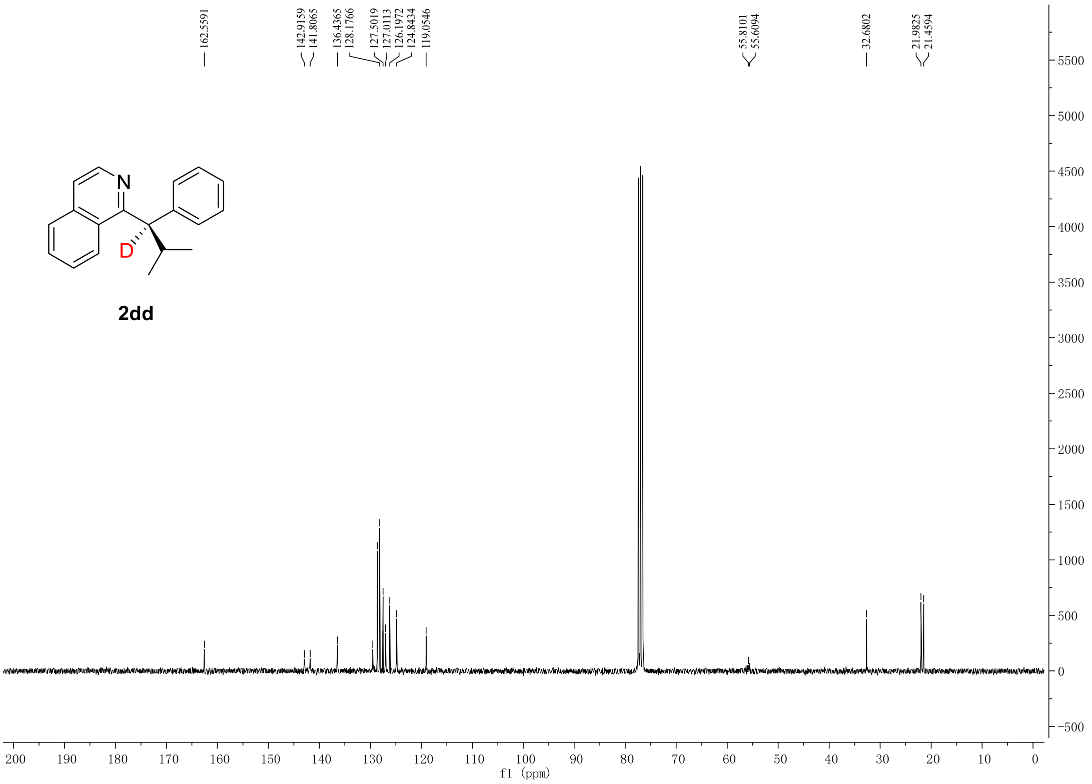

Figure S103. ^{13}C NMR spectrum for **2dd**, related to **Figure 2**.

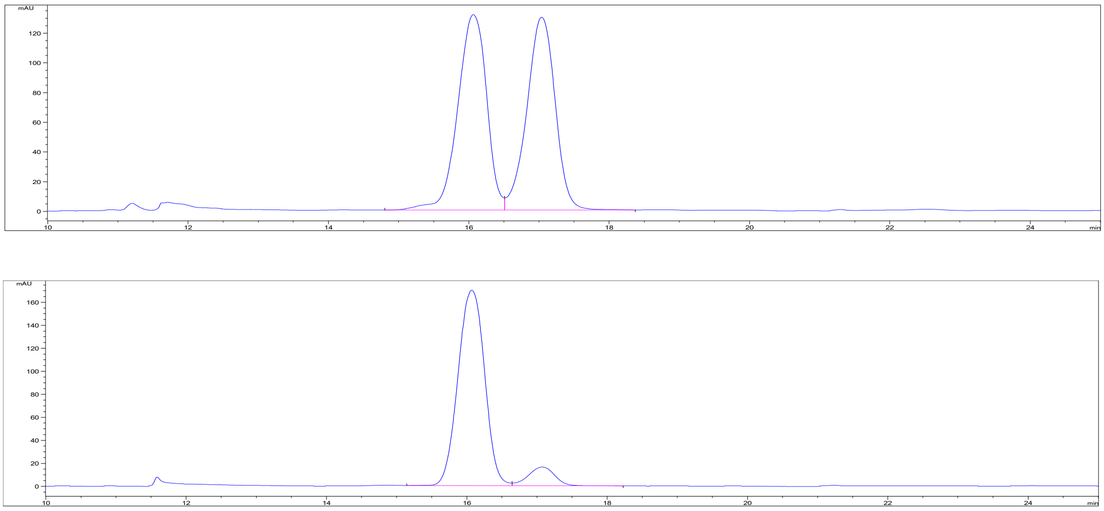

Figure S104. HPLC spectrum for **2dd**, related to **Figure 2**.

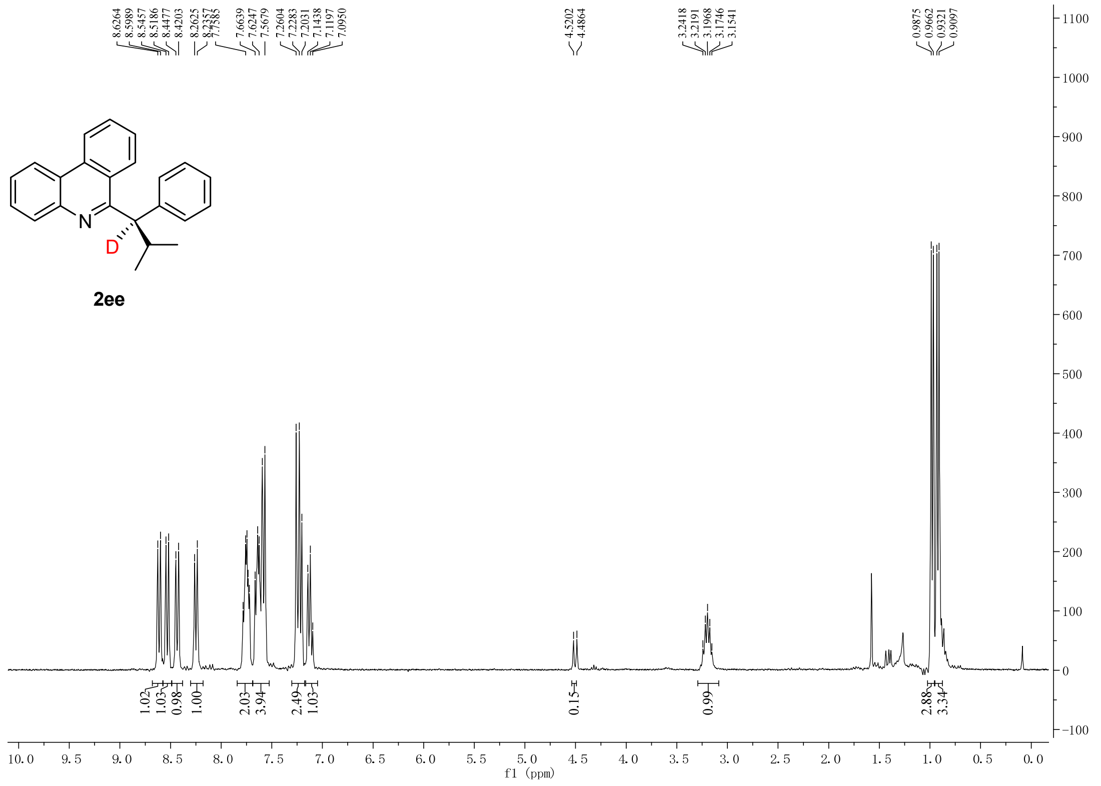

Figure S105. ^{1}H NMR spectrum for **2ee**, related to **Figure 2**.

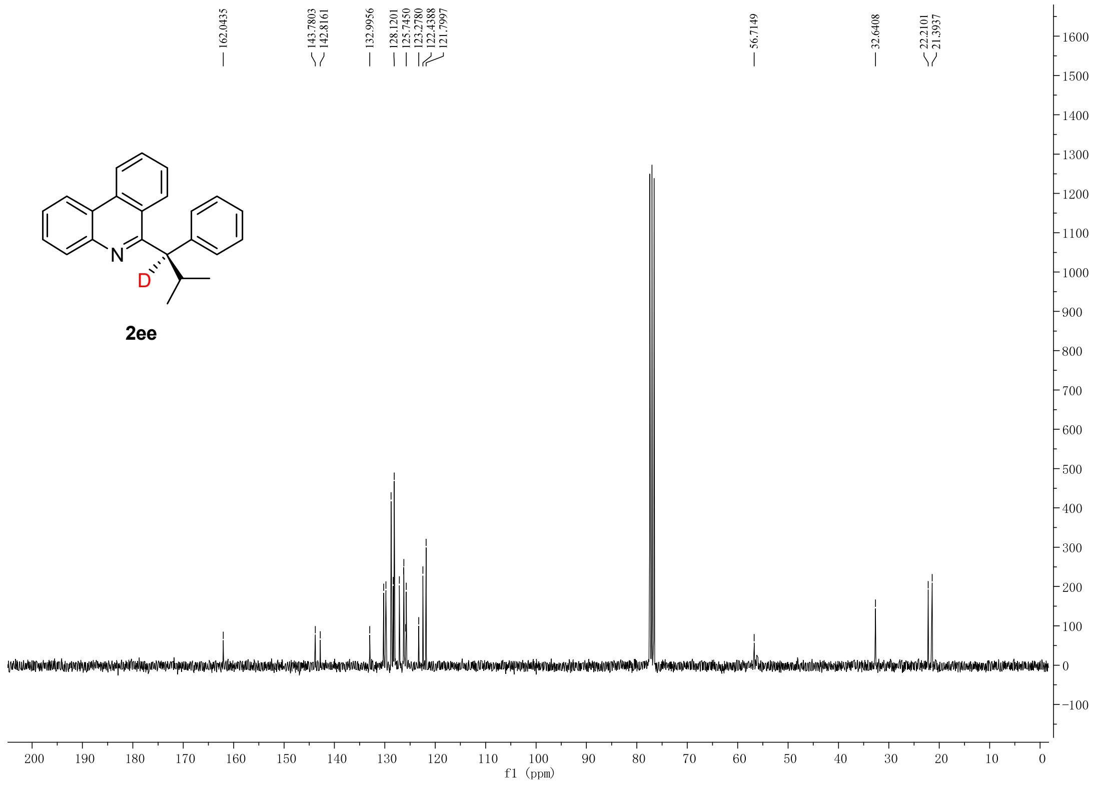

Figure S106. ^{13}C NMR spectrum for **2ee**, related to **Figure 2**.

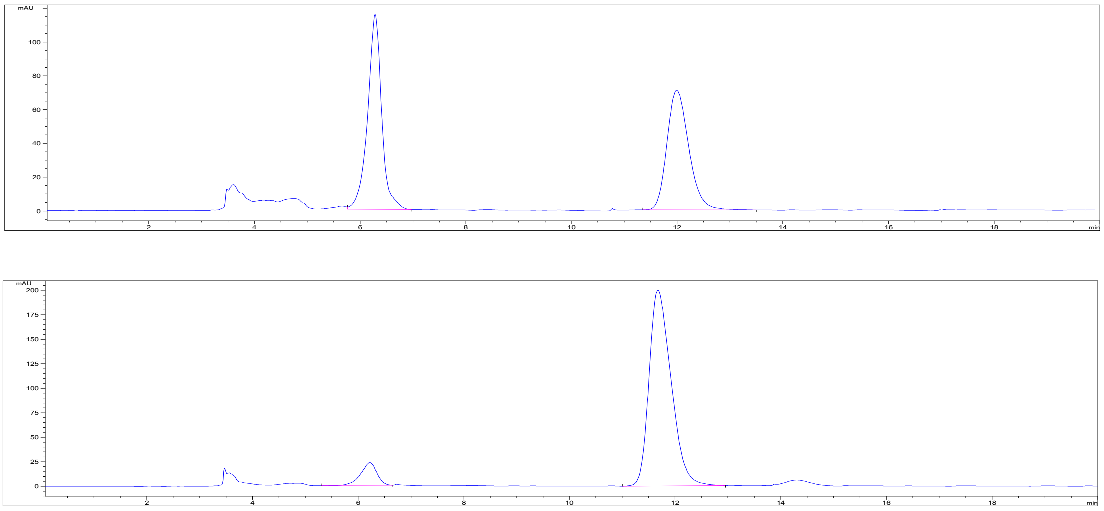

Figure S107. HPLC spectrum for **2ee**, related to **Figure 2**.

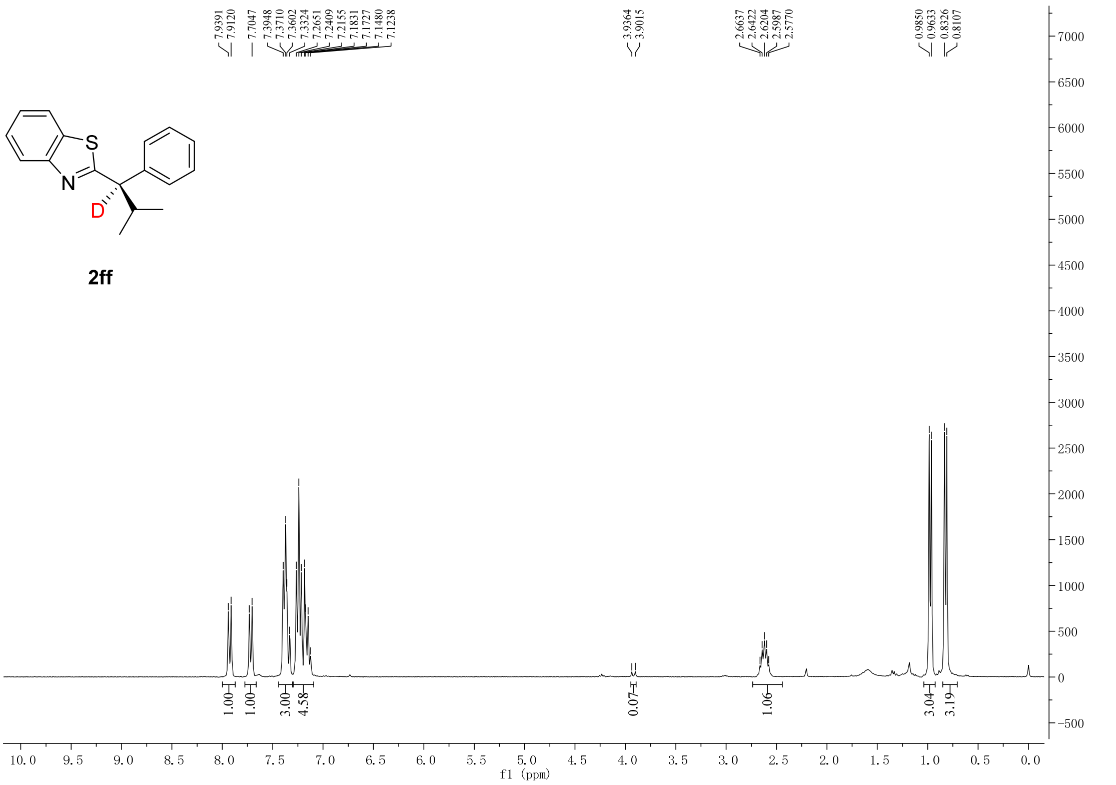

Figure S108. ^{1}H NMR spectrum for **2ff**, related to **Figure 2**.

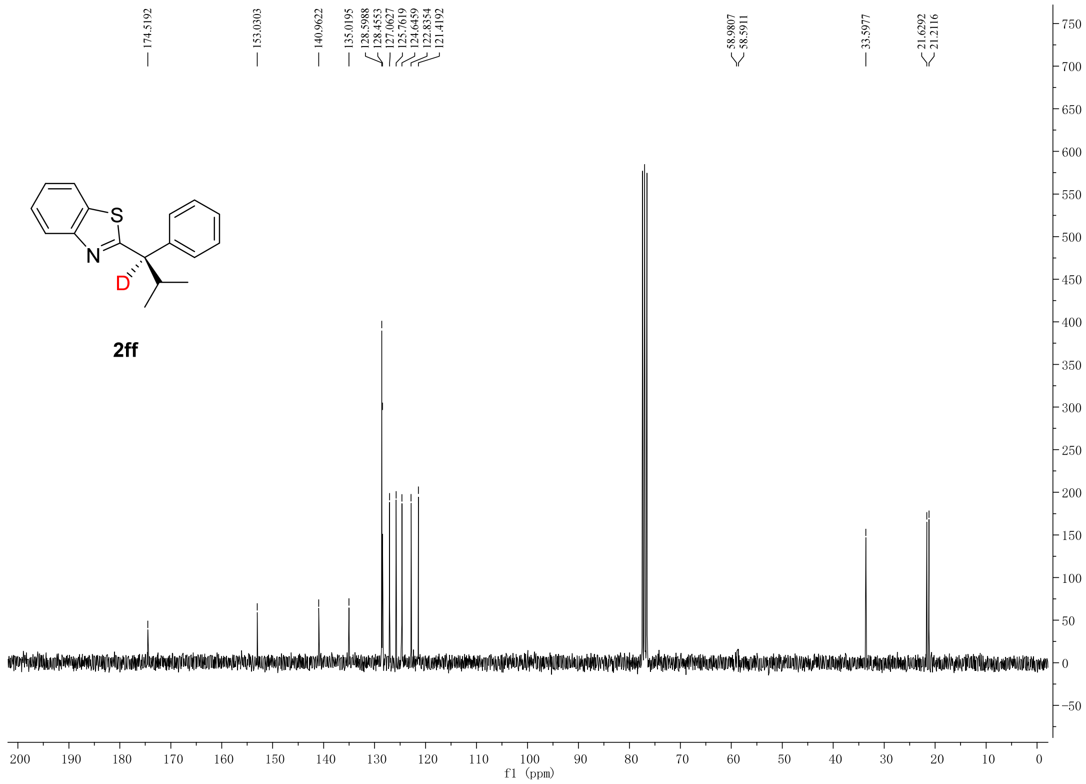

Figure S109. ^{13}C NMR spectrum for **2ff**, related to **Figure 2**.

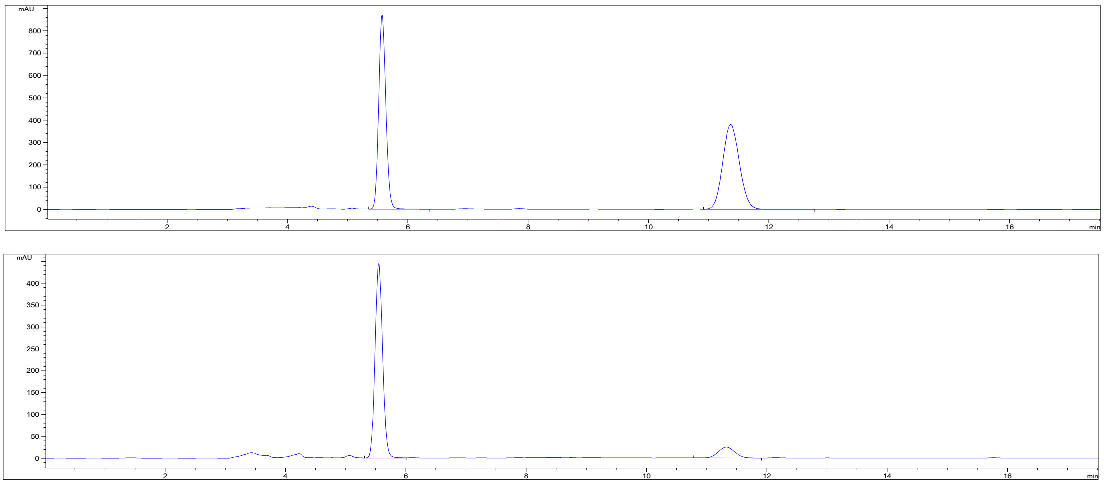

Figure S110. HPLC spectrum for **2ff**, related to **Figure 2**.

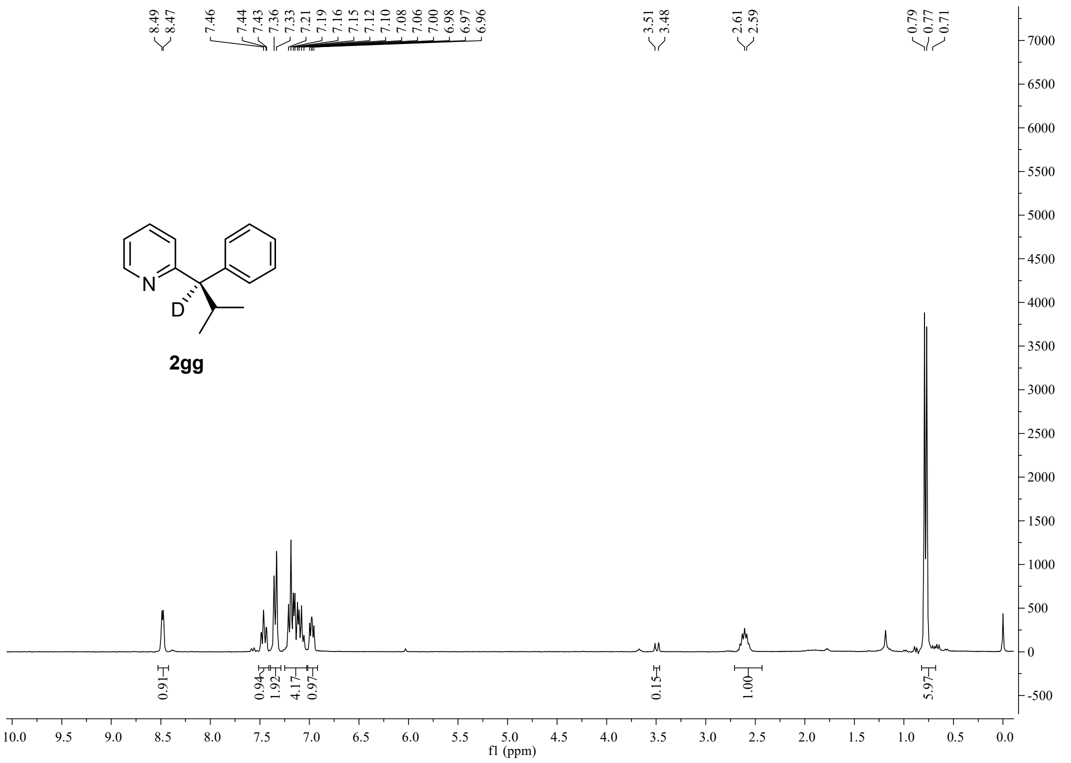

Figure S111. ^{1}H NMR spectrum for **2gg**, related to **Figure 2**.

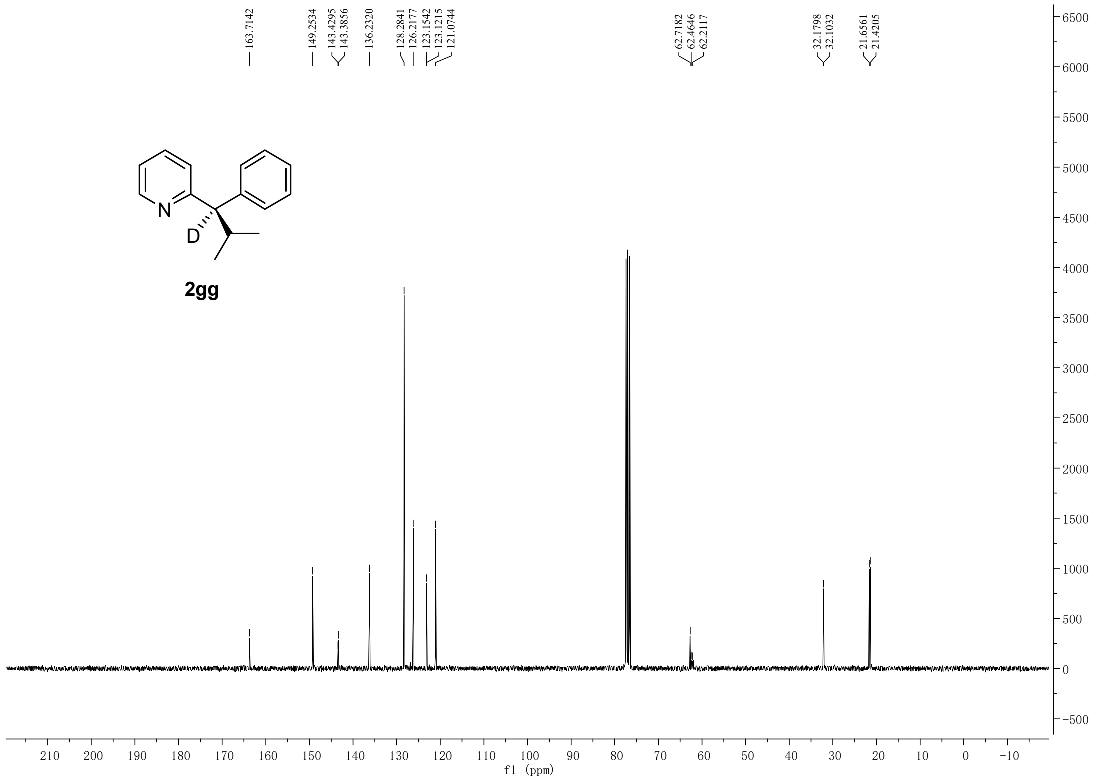

Figure S112. ^{13}C NMR spectrum for **2gg**, related to **Figure 2**.

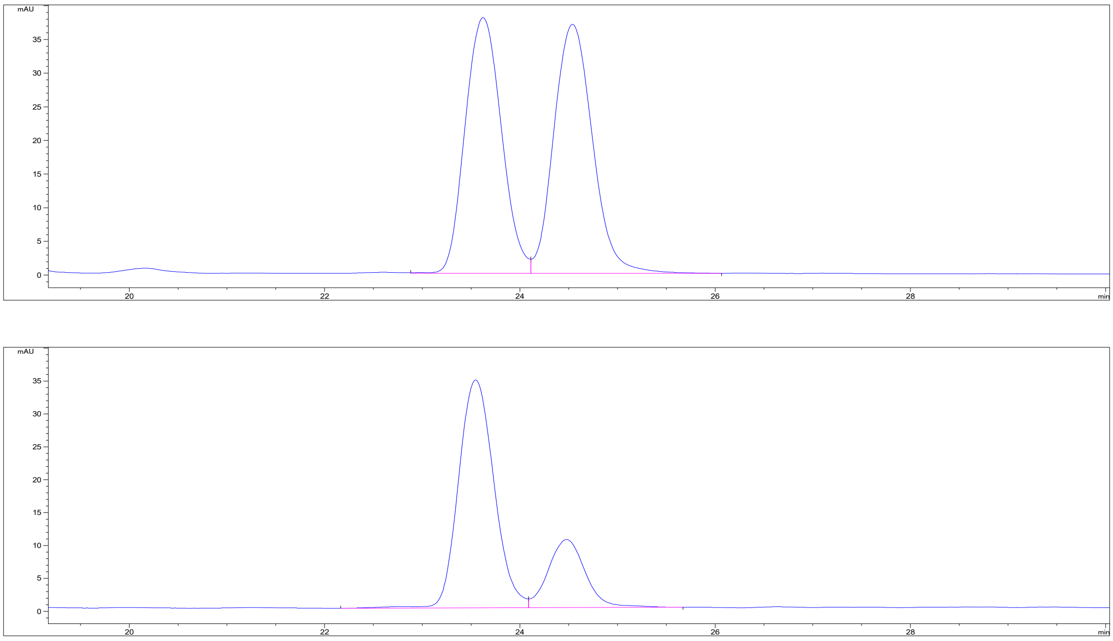

Figure S113. HPLC spectrum for **2gg**, related to **Figure 2**.

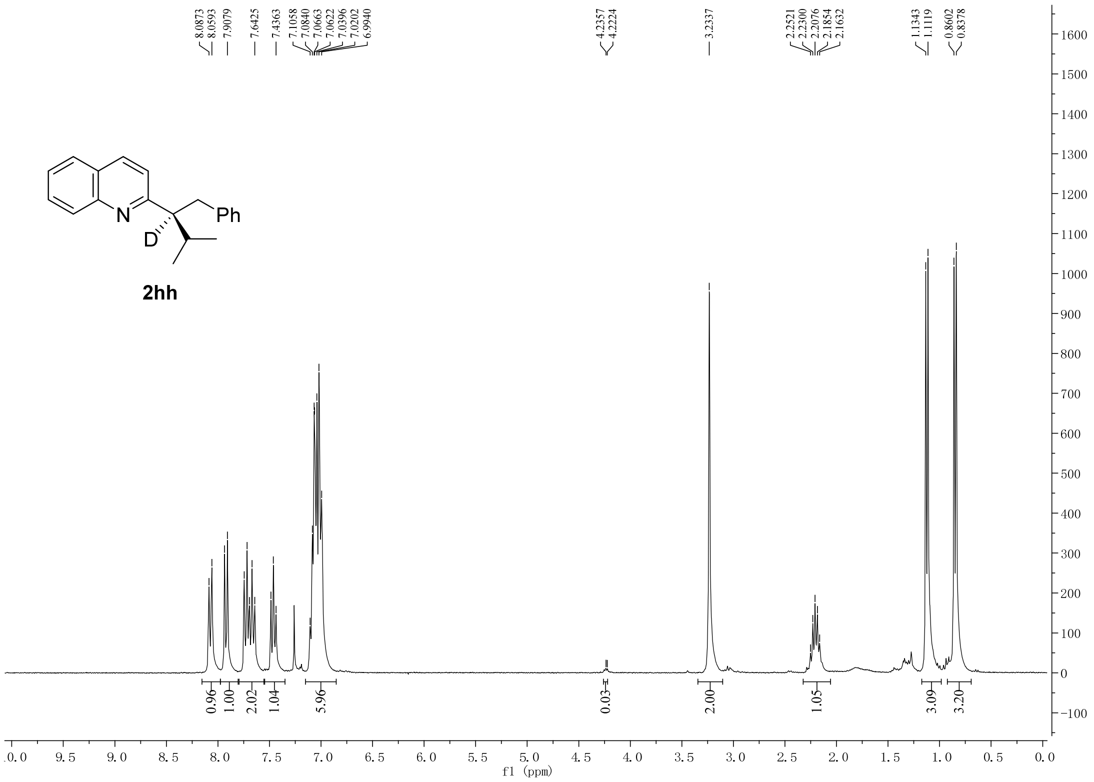

Figure S114. ^{1}H NMR spectrum for **2hh**, related to **Figure 2**.

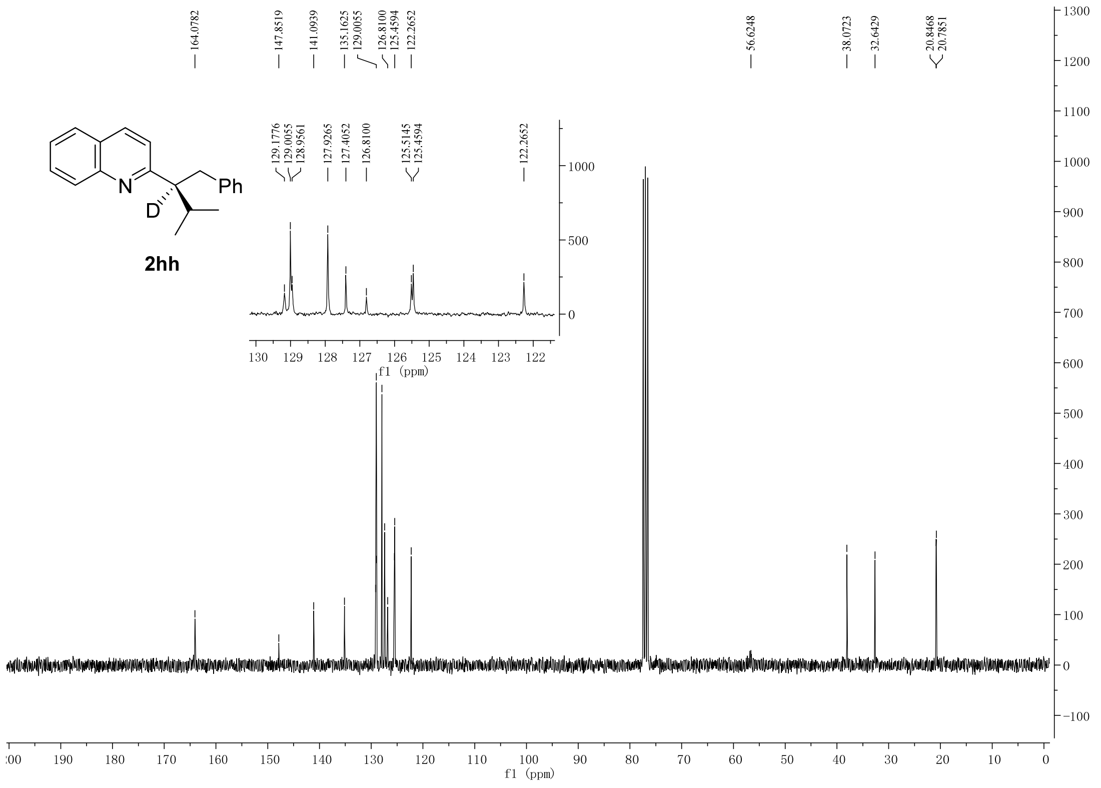

Figure S115. ^{13}C NMR spectrum for **2hh**, related to **Figure 2**.

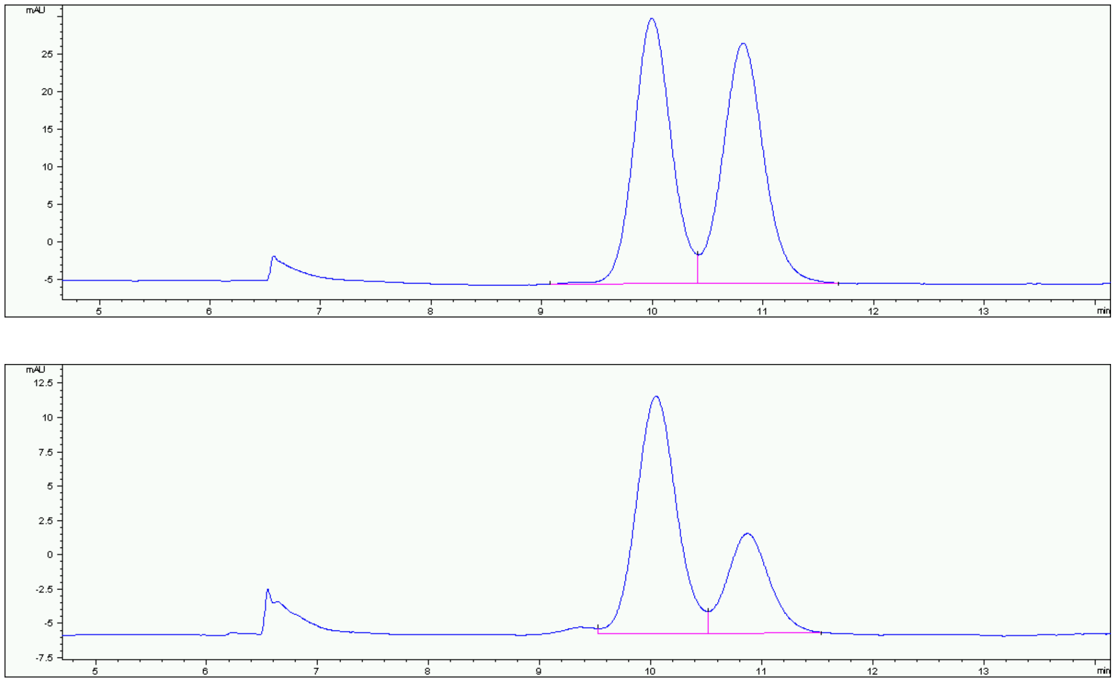

Figure S116. HPLC spectrum for **2hh**, related to **Figure 2**.

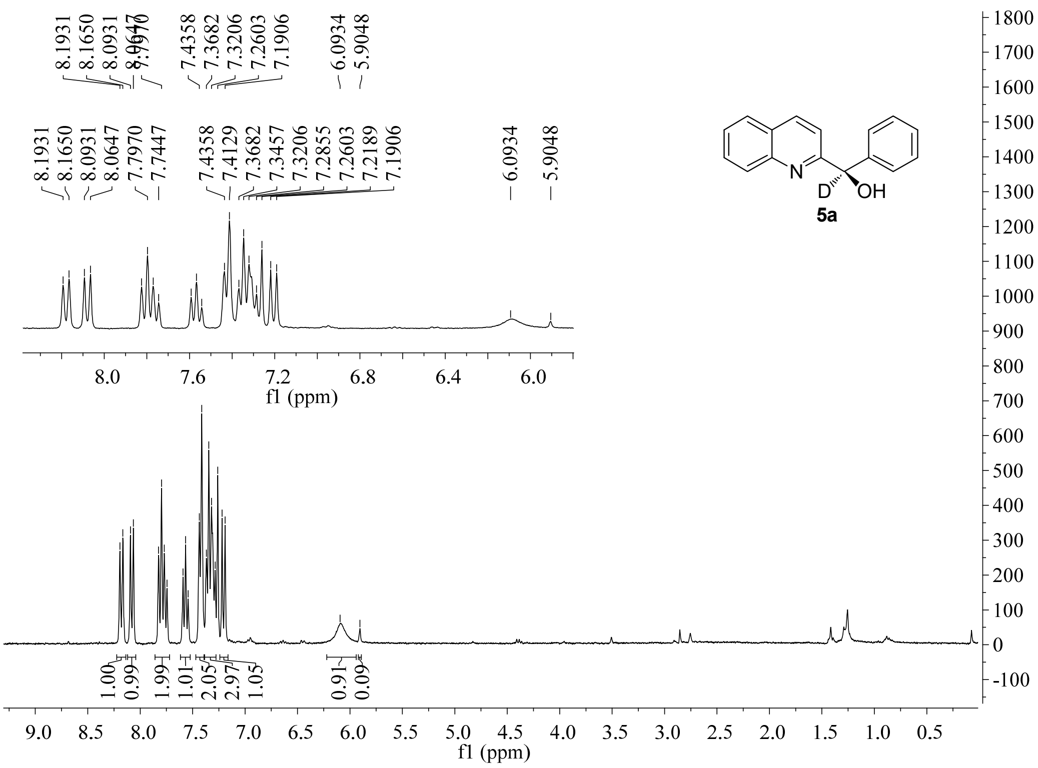

Figure S117. ^{1}H NMR spectrum for **5a**, related to **Figure 5**.

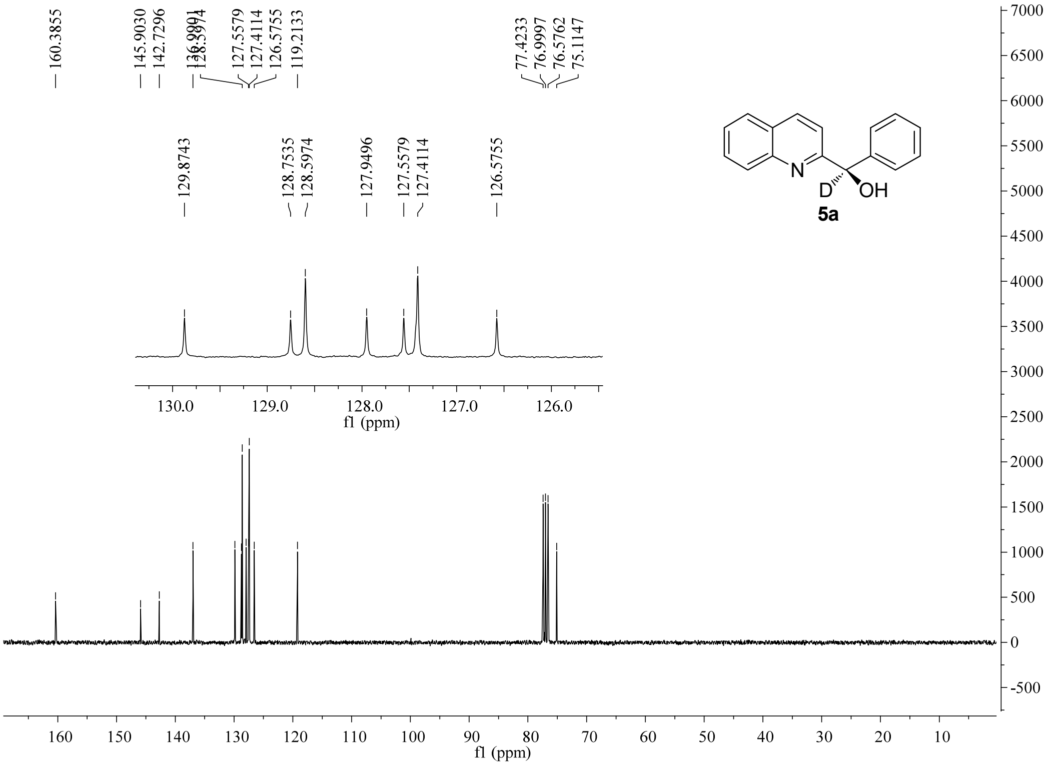

Figure S118. ^{13}C NMR spectrum for **5a**, related to **Figure 5**.

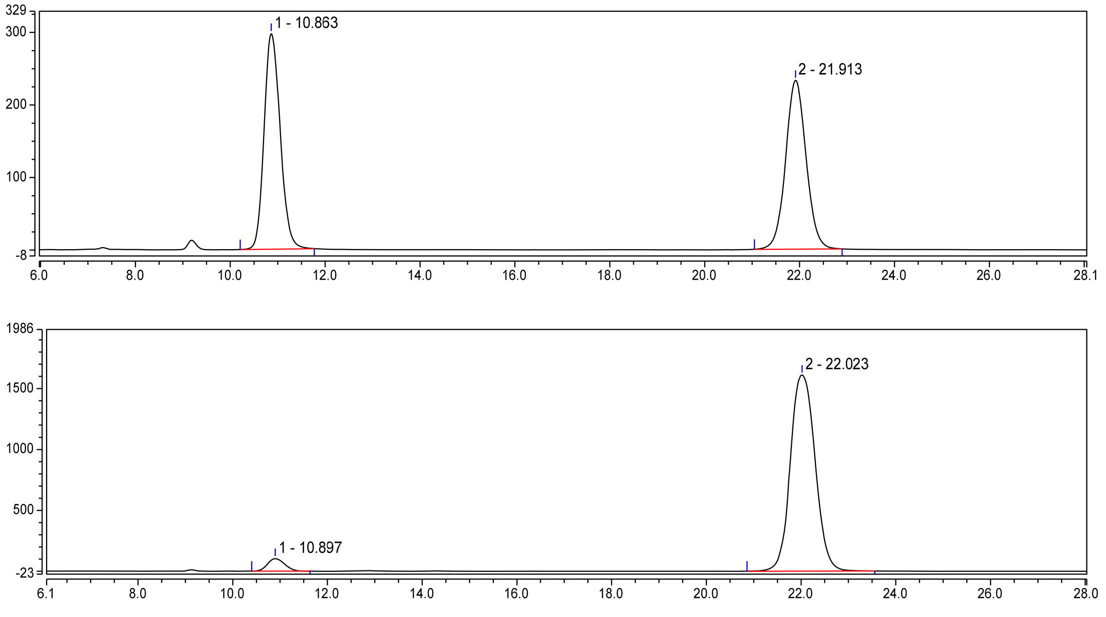

Figure S119. HPLC spectrum for **5a**, related to **Figure 5**.

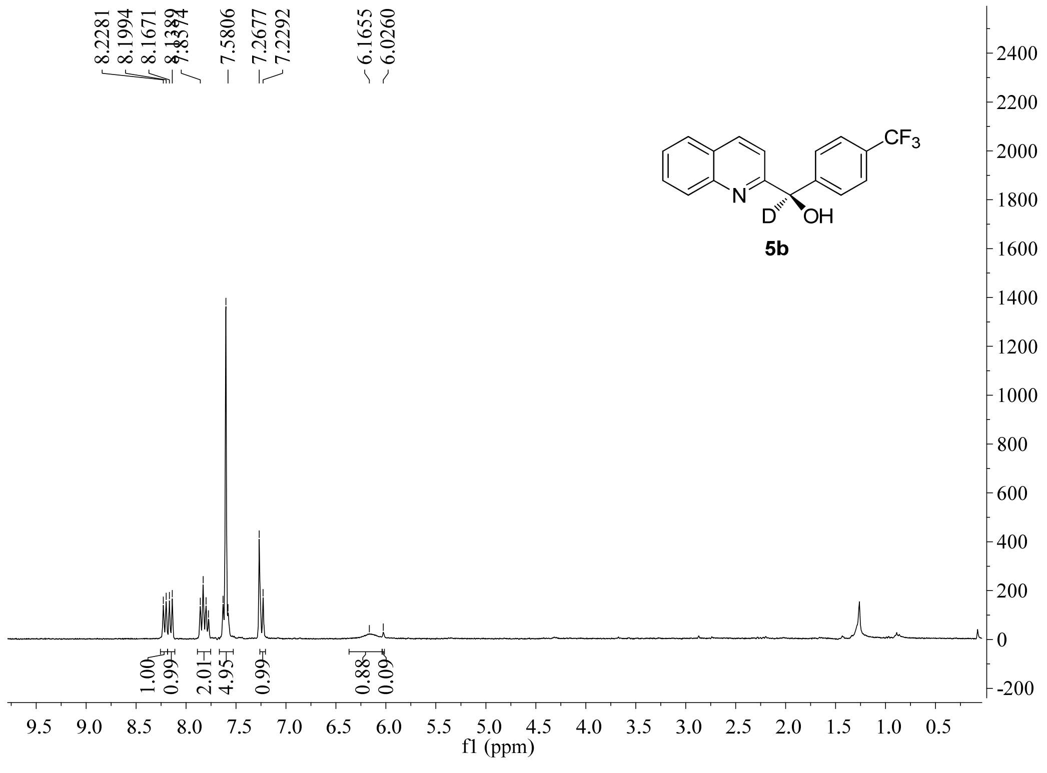

Figure S120. ^{1}H NMR spectrum for **5b**, related to **Figure 5**.

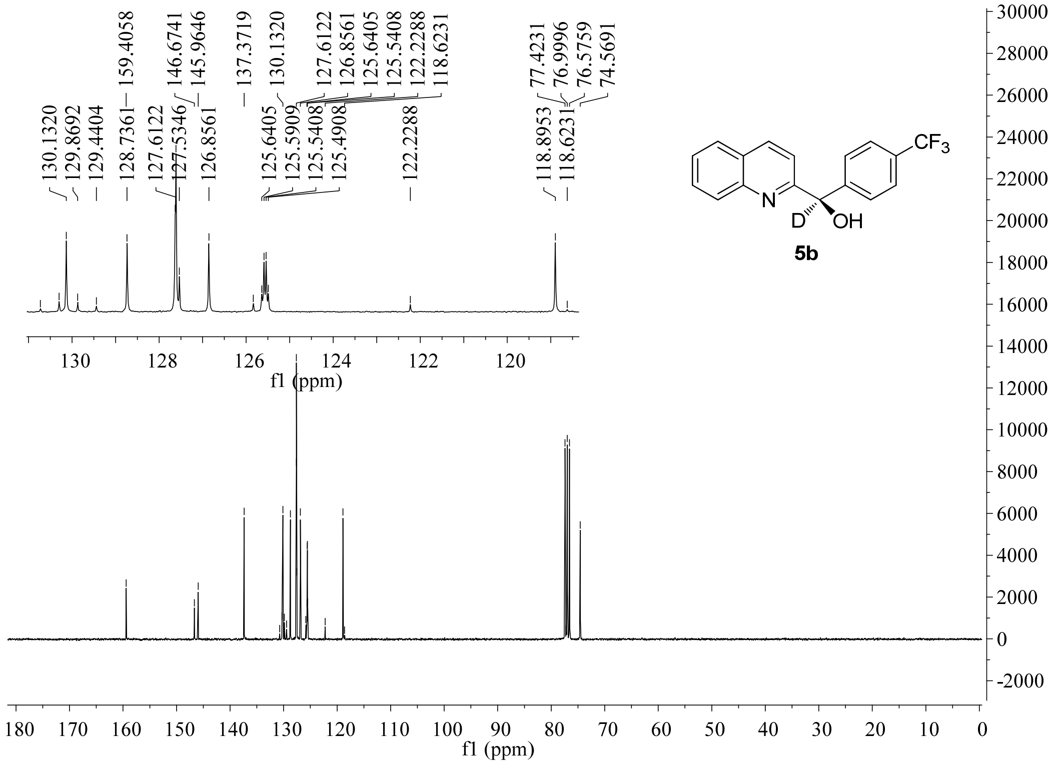

Figure S121. ^{13}C NMR spectrum for **5b**, related to **Figure 5**.

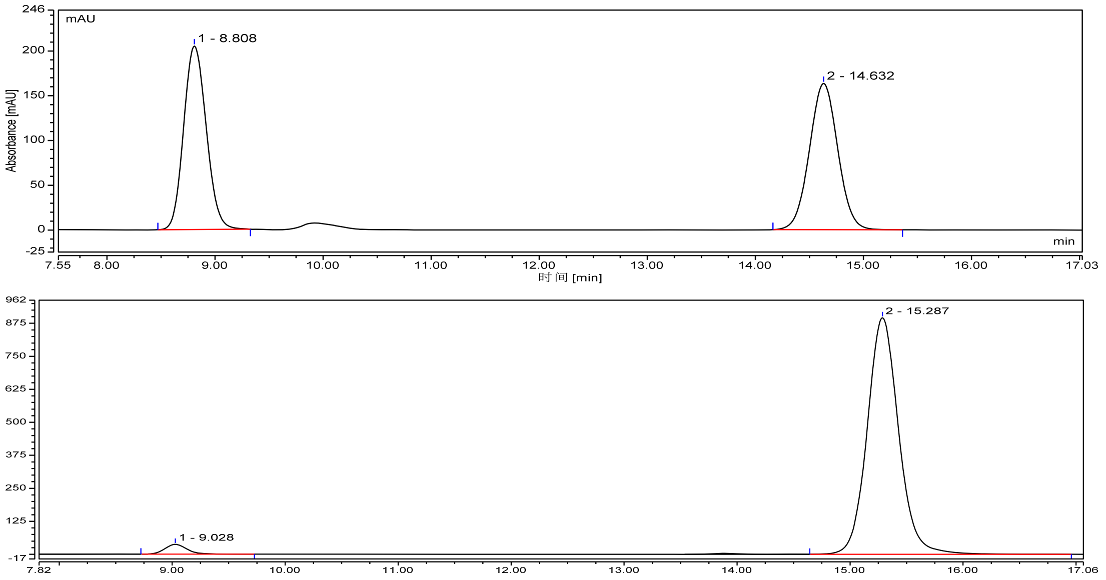

Figure S122. HPLC spectrum for **5b**, related to **Figure 5**.

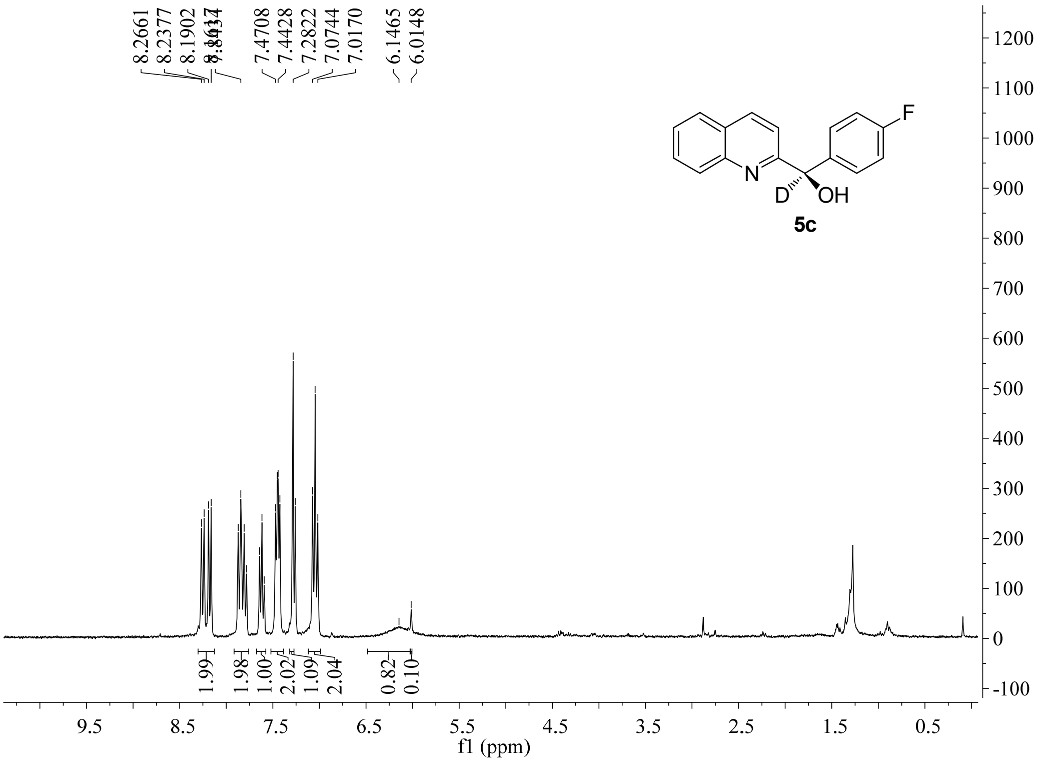

Figure S123. ^{1}H NMR spectrum for **5c**, related to **Figure 5**.

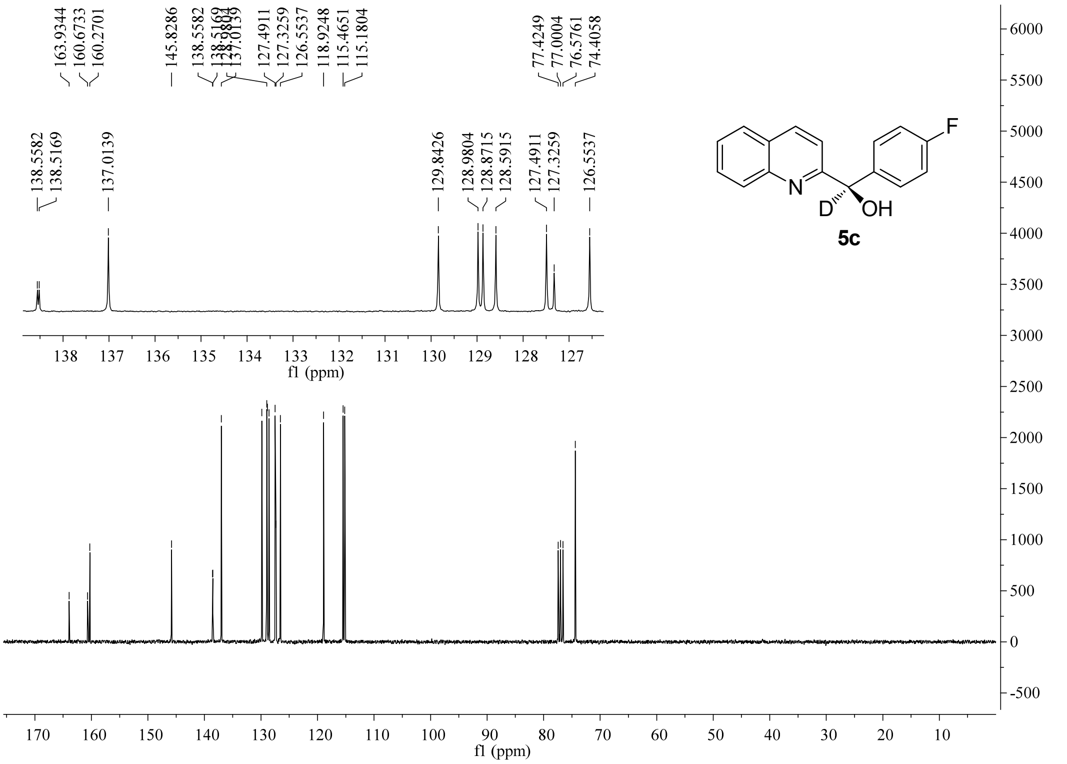

Figure S124. ^{13}C NMR spectrum for **5c**, related to **Figure 5**.

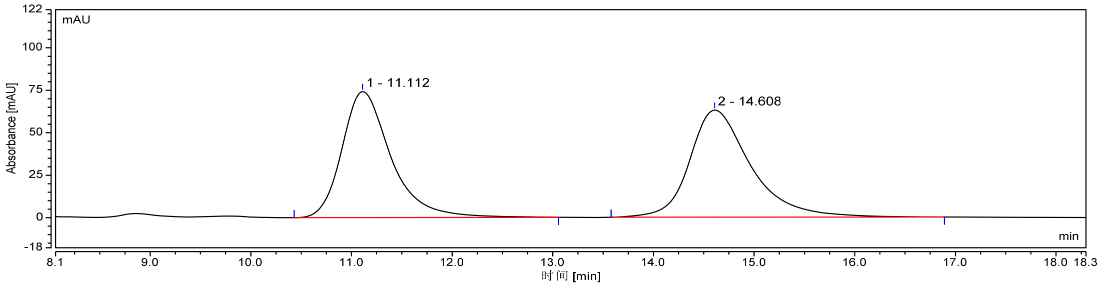

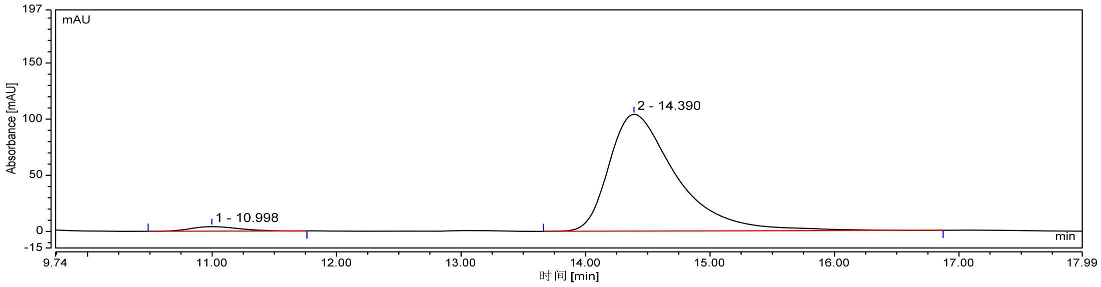

Figure S125. HPLC spectrum for **5c**, related to **Figure 5**.

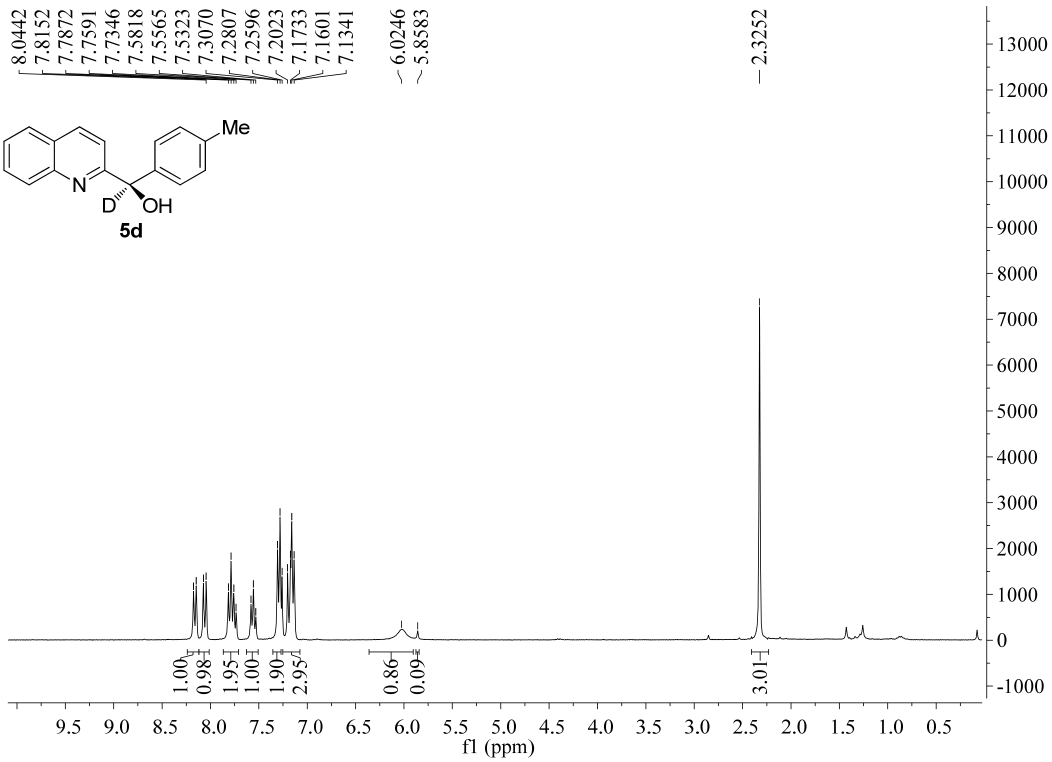

Figure S126. ^{1}H NMR spectrum for **5d**, related to **Figure 5**.

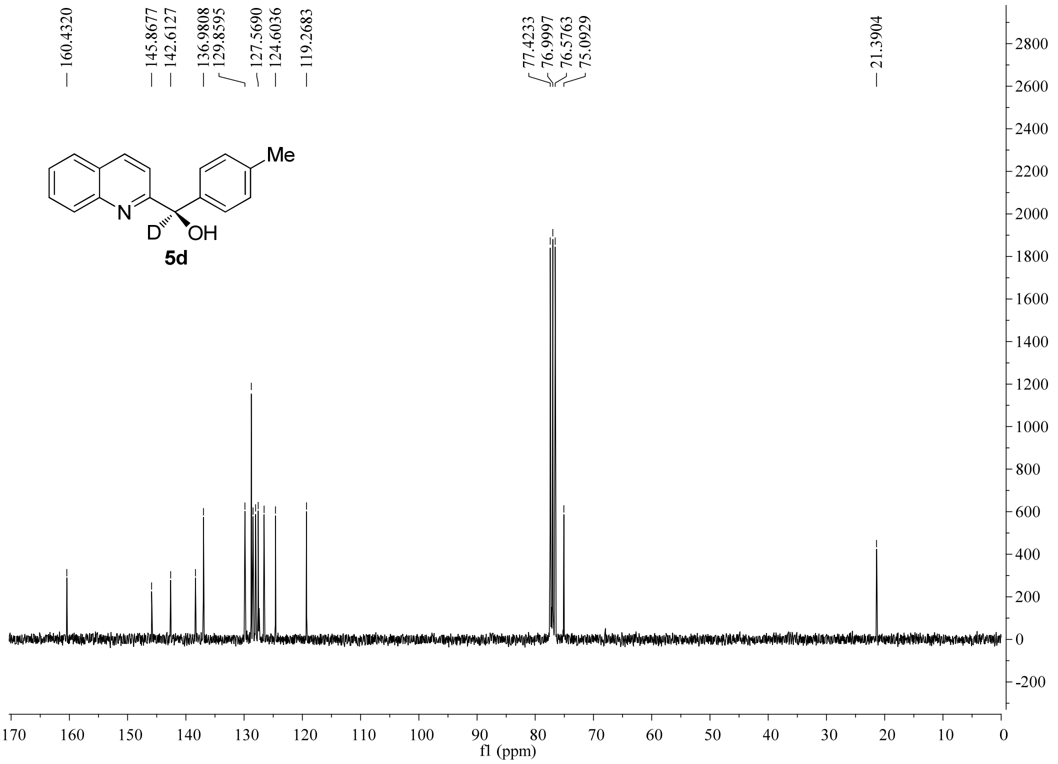

Figure S127. ^{13}C NMR spectrum for **5d**, related to **Figure 5**.

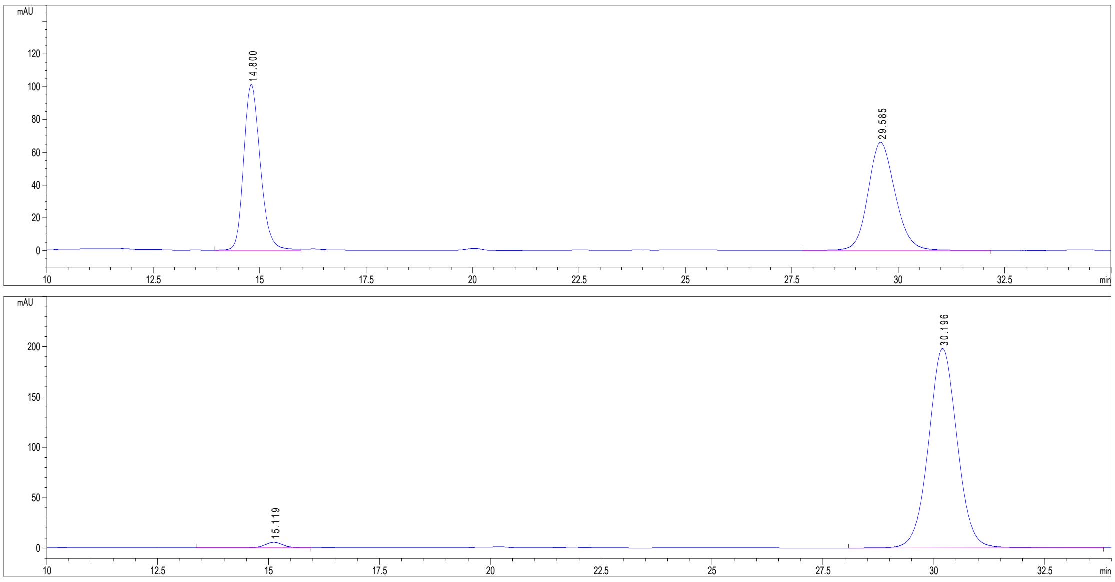

Figure S128. HPLC spectrum for **5d**, related to **Figure 5**.

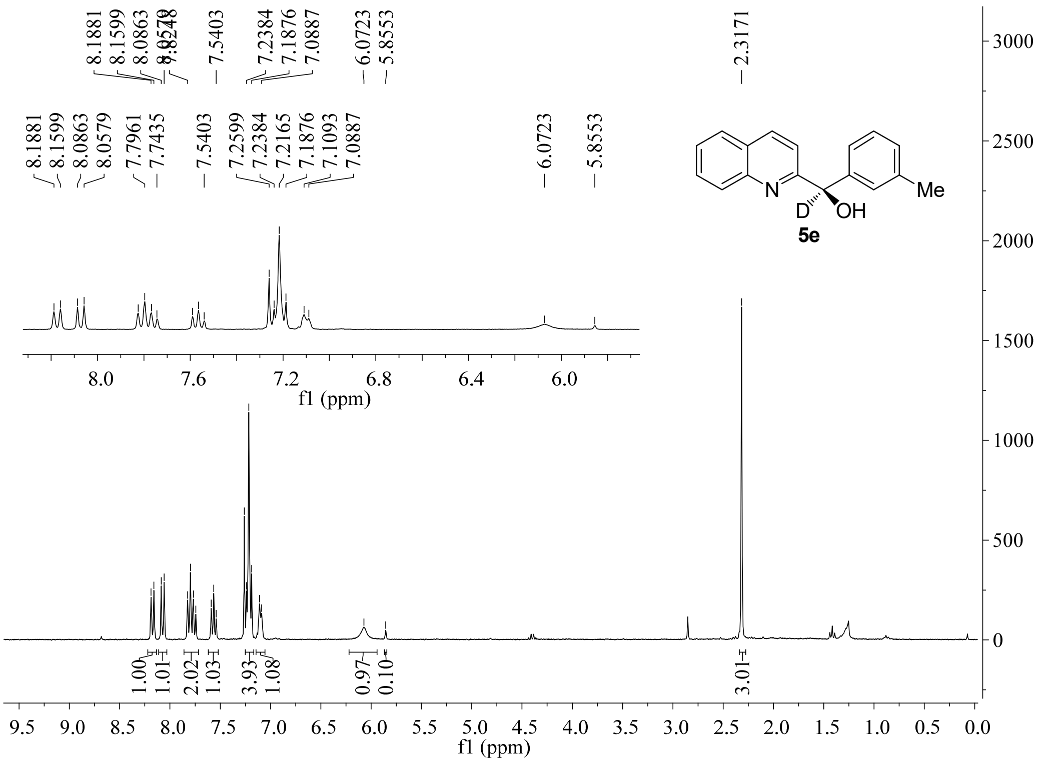

Figure S129. ^{1}H NMR spectrum for **5e**, related to **Figure 5**.

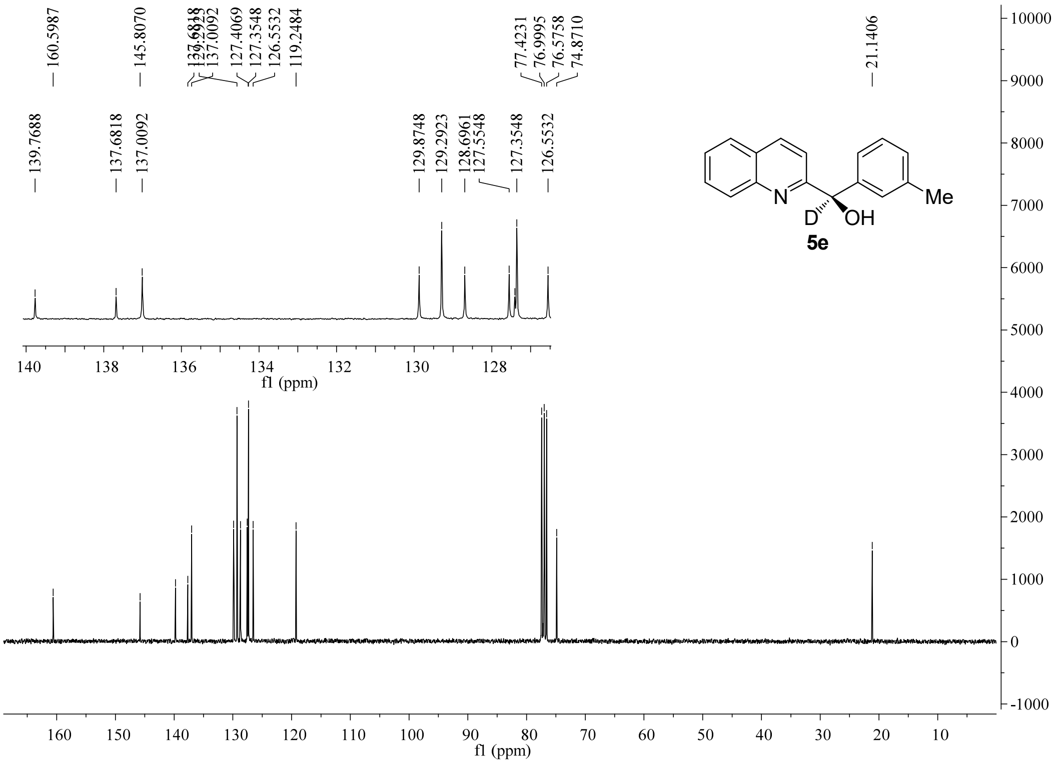

Figure S130. ^{13}C NMR spectrum for **5e**, related to **Figure 5**.

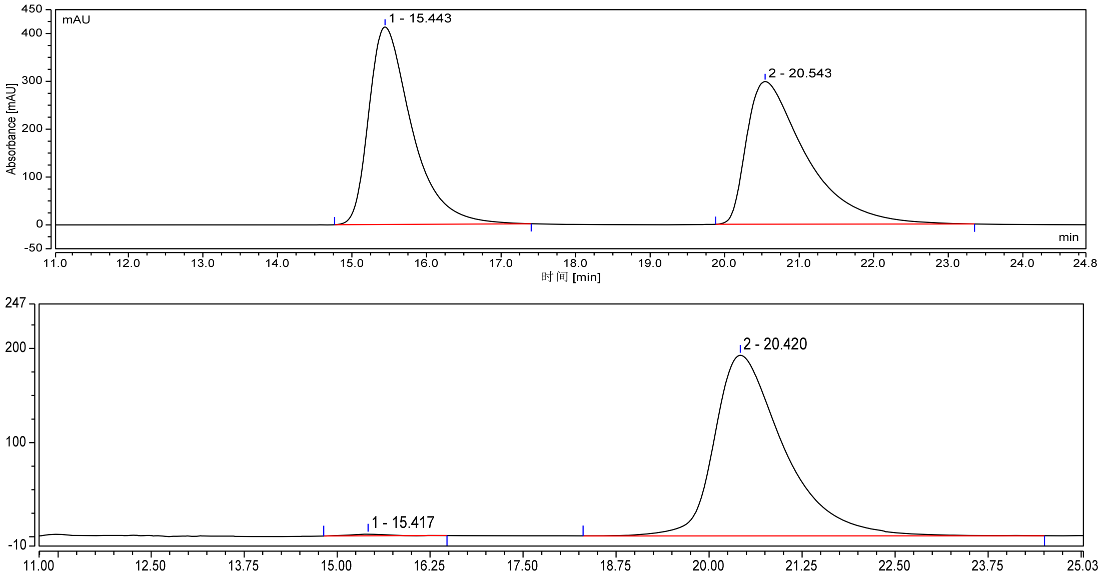

Figure S131. HPLC spectrum for **5e**, related to **Figure 5**.

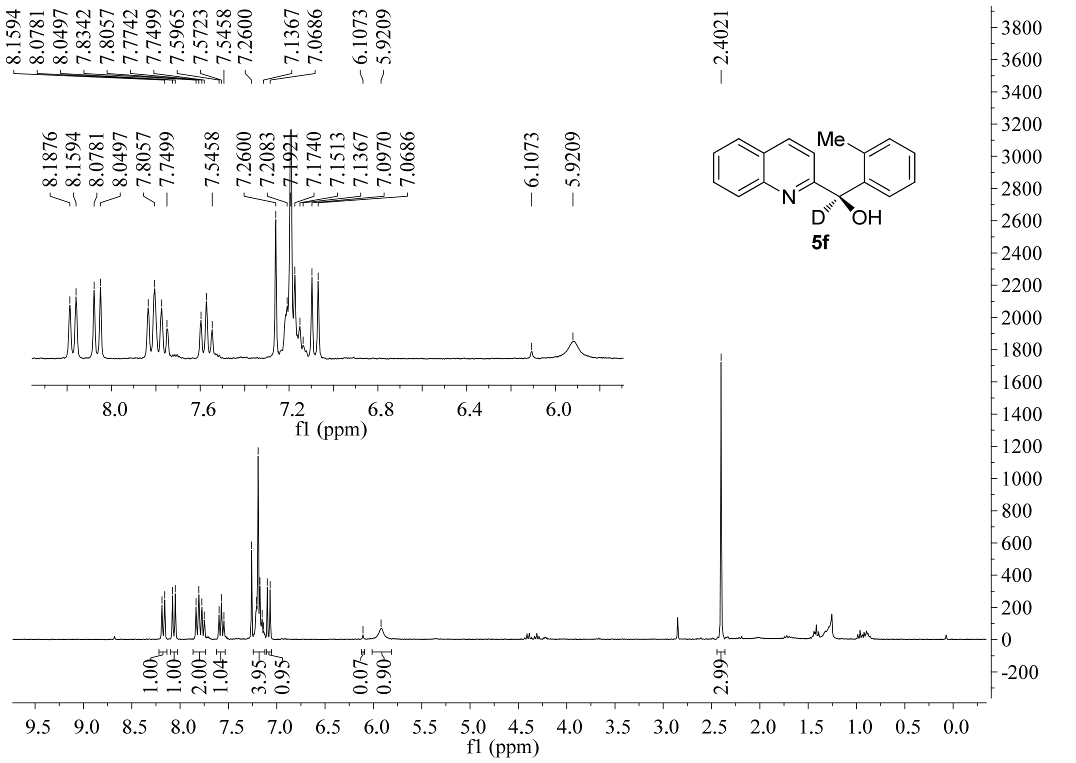

Figure S132. ^{1}H NMR spectrum for **5f**, related to **Figure 5**.

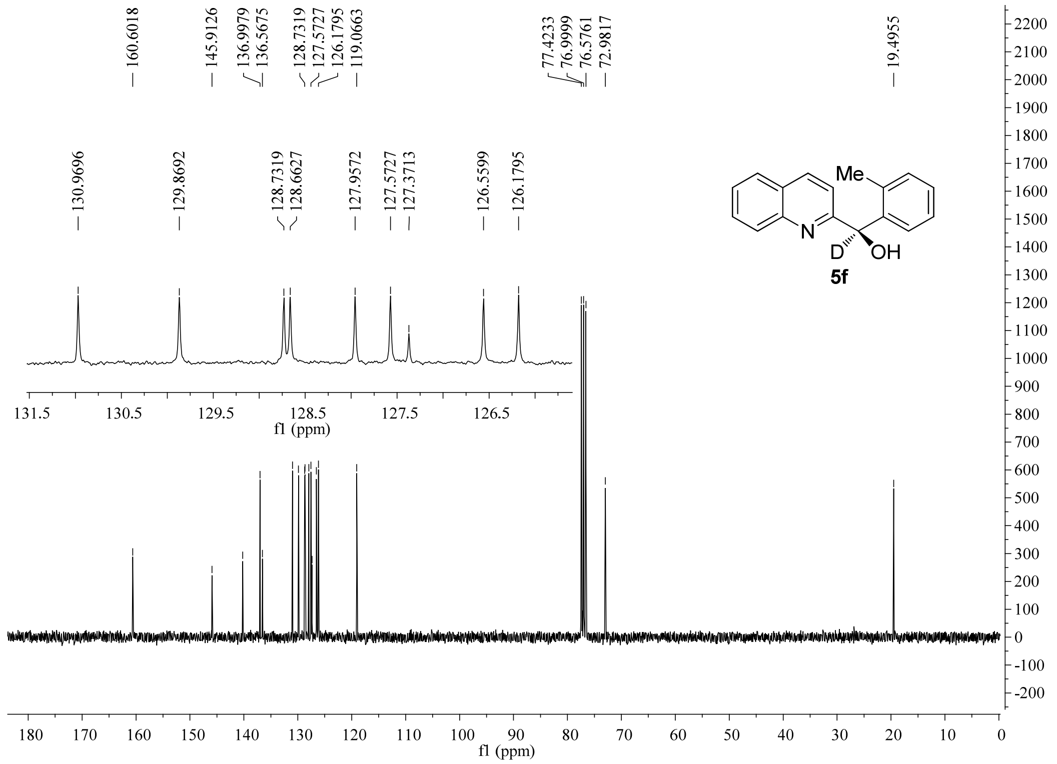

Figure S133. ^{13}C NMR spectrum for **5f**, related to **Figure 5**.

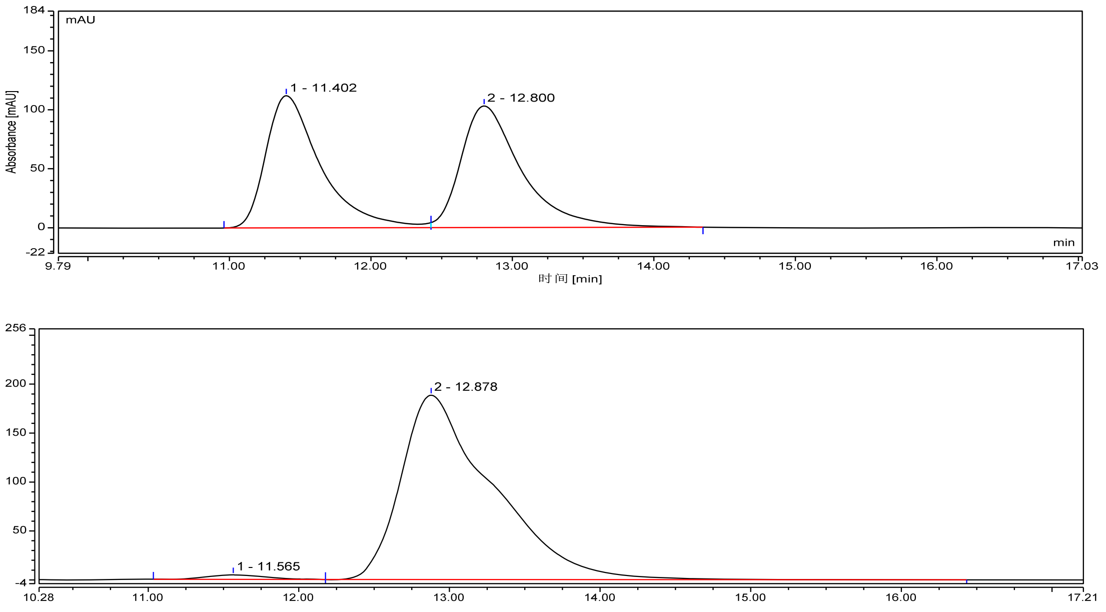

Figure S134. HPLC spectrum for **5f**, related to **Figure 5**.

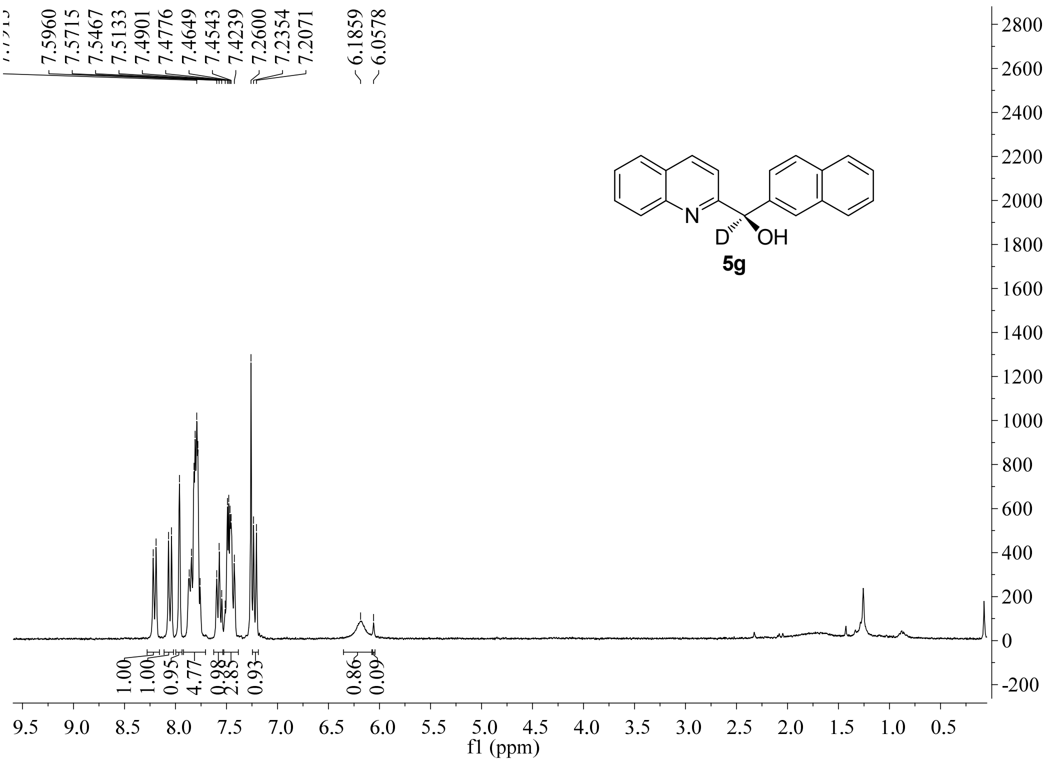

Figure S135. ^{1}H NMR spectrum for **5g**, related to **Figure 5**.

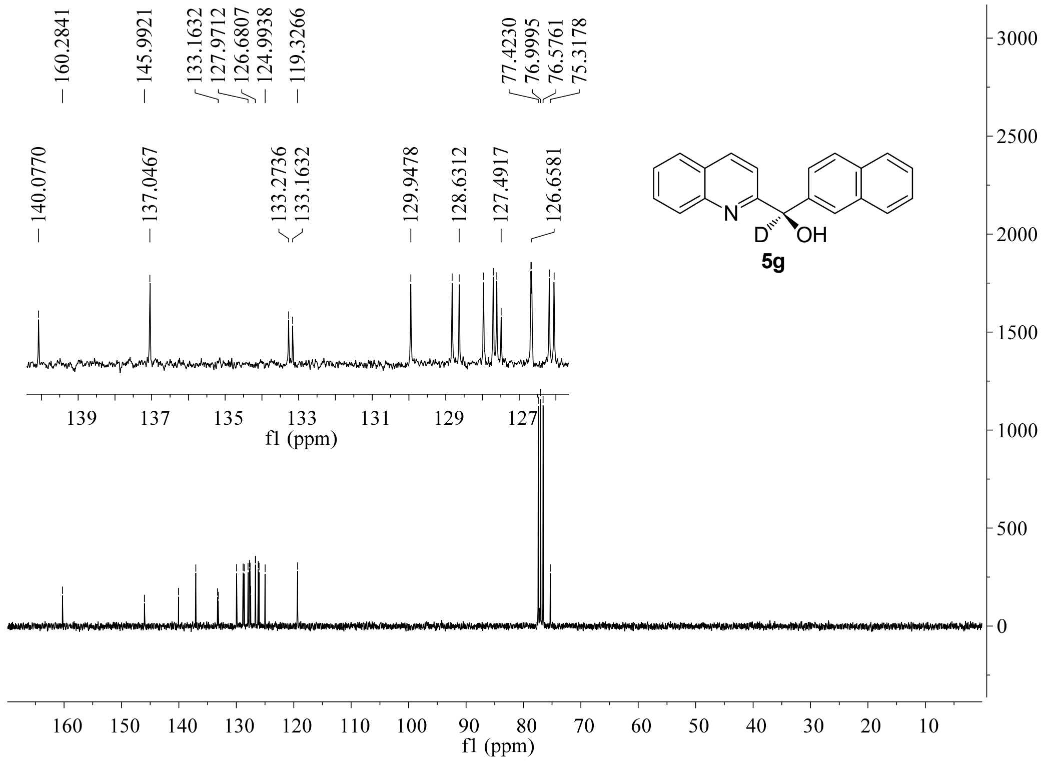

Figure S136. ^{13}C NMR spectrum for **5g**, related to **Figure 5**.

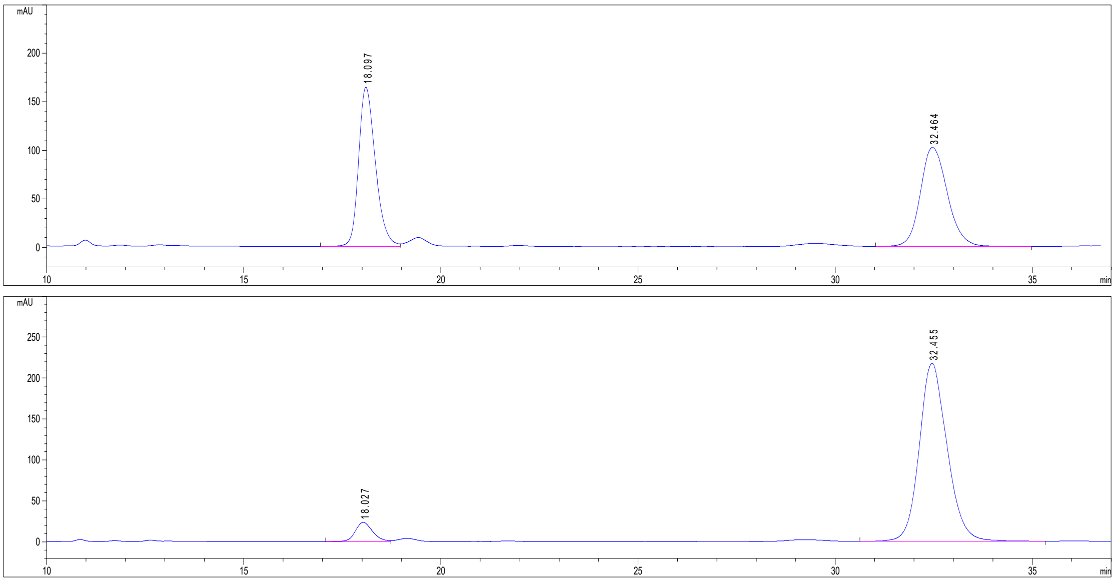

Figure S137. HPLC spectrum for **5g**, related to **Figure 5**.

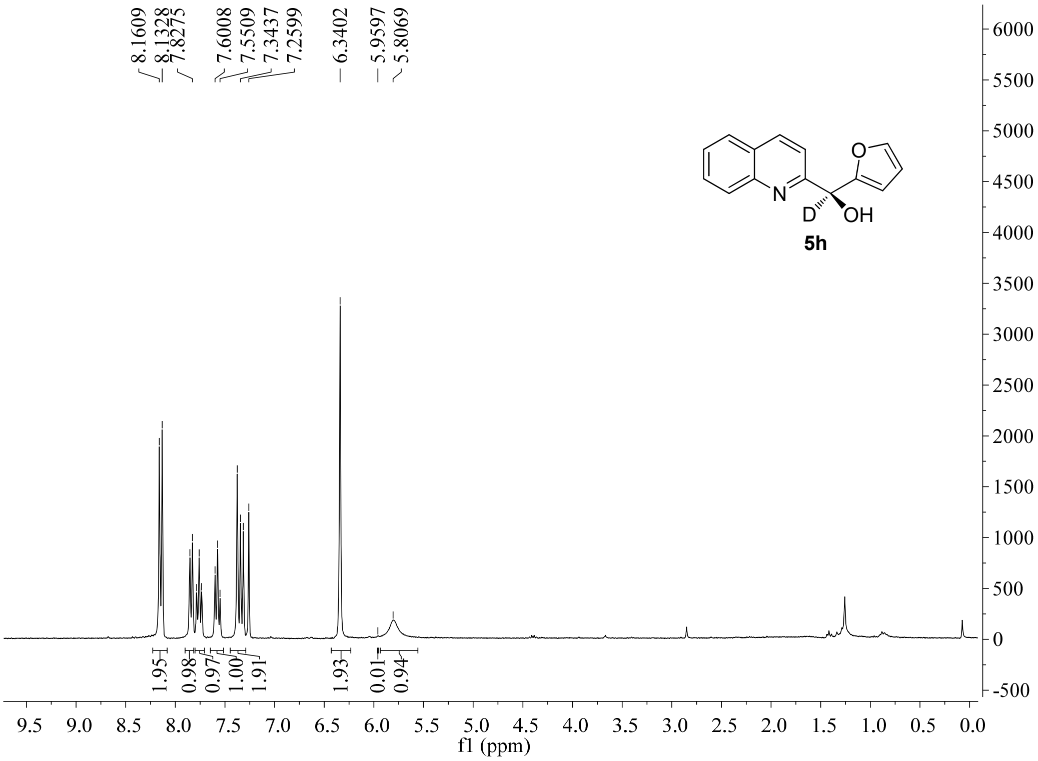

Figure S138. ^{1}H NMR spectrum for **5h**, related to **Figure 5**.

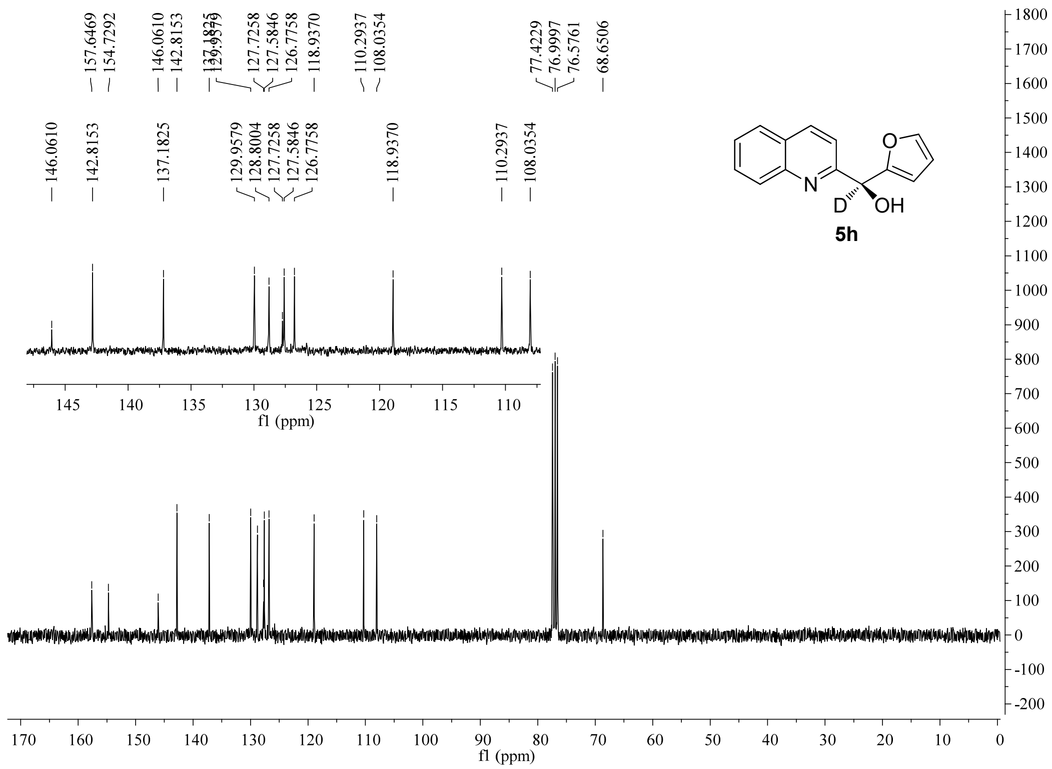

Figure S139. ^{13}C NMR spectrum for **5h**, related to **Figure 5**.

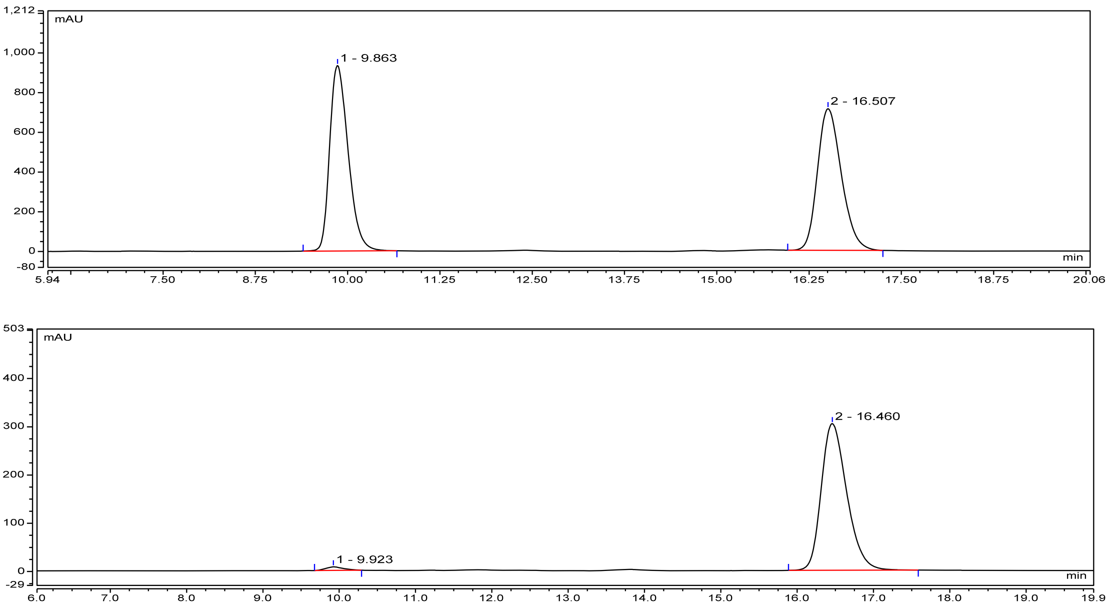

Figure S140. HPLC spectrum for **5h**, related to **Figure 5**.

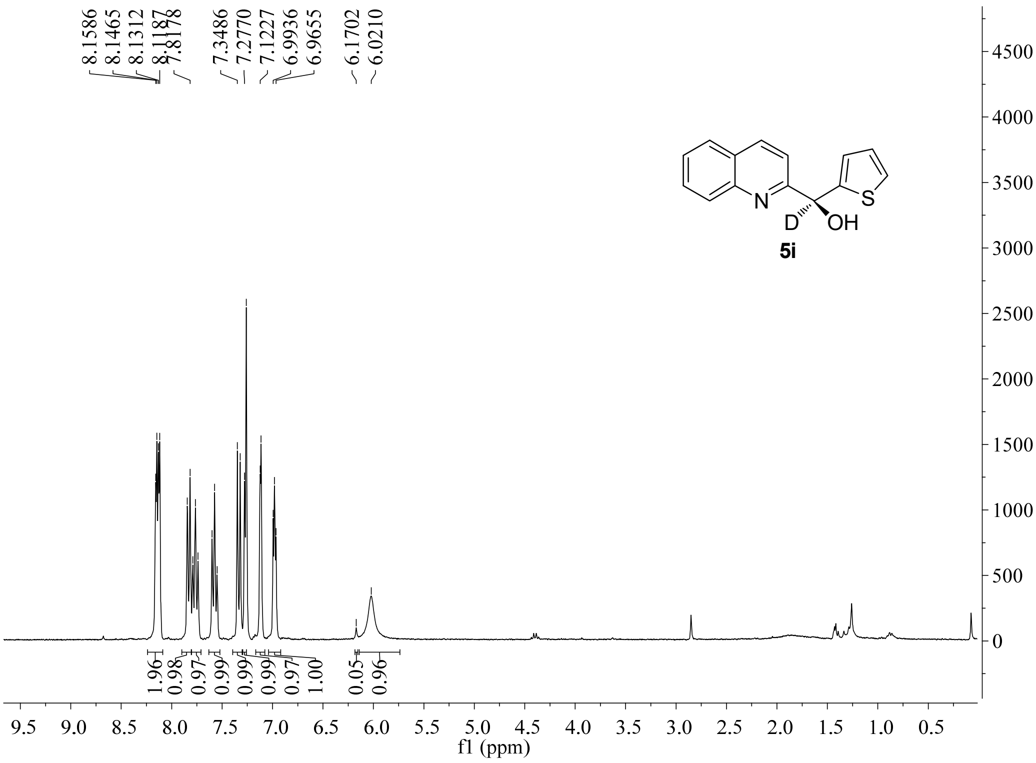

Figure S141. ^{1}H NMR spectrum for **5i**, related to **Figure 5**.

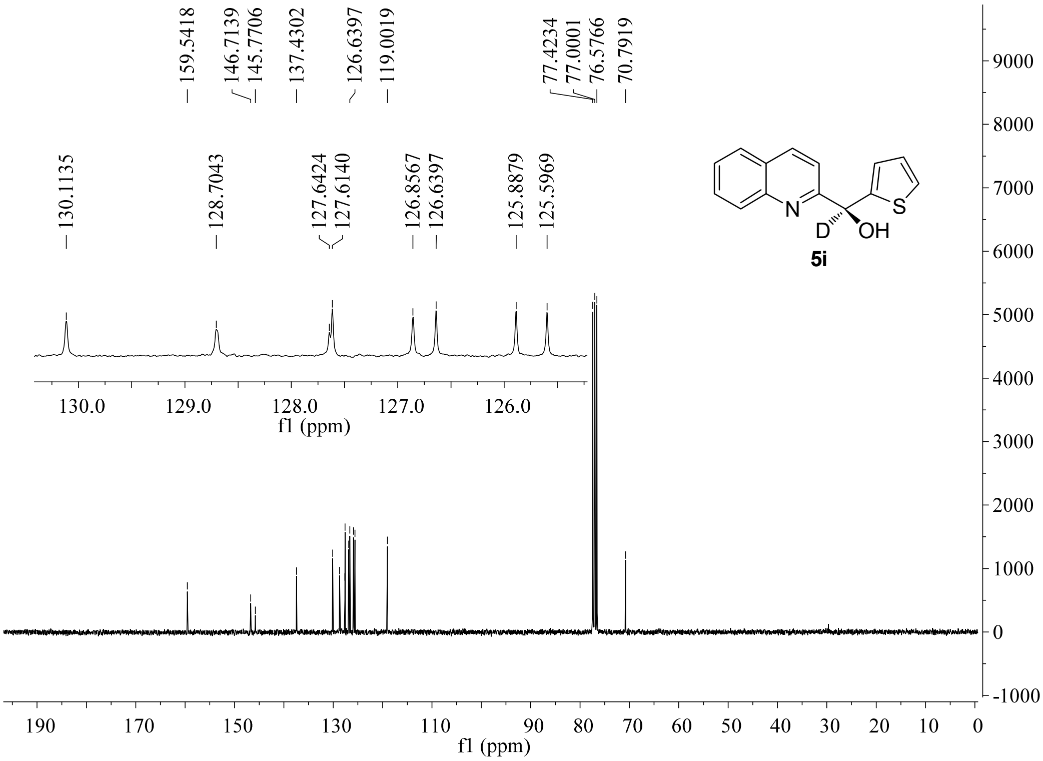

Figure S142. ^{13}C NMR spectrum for **5i**, related to **Figure 5**.

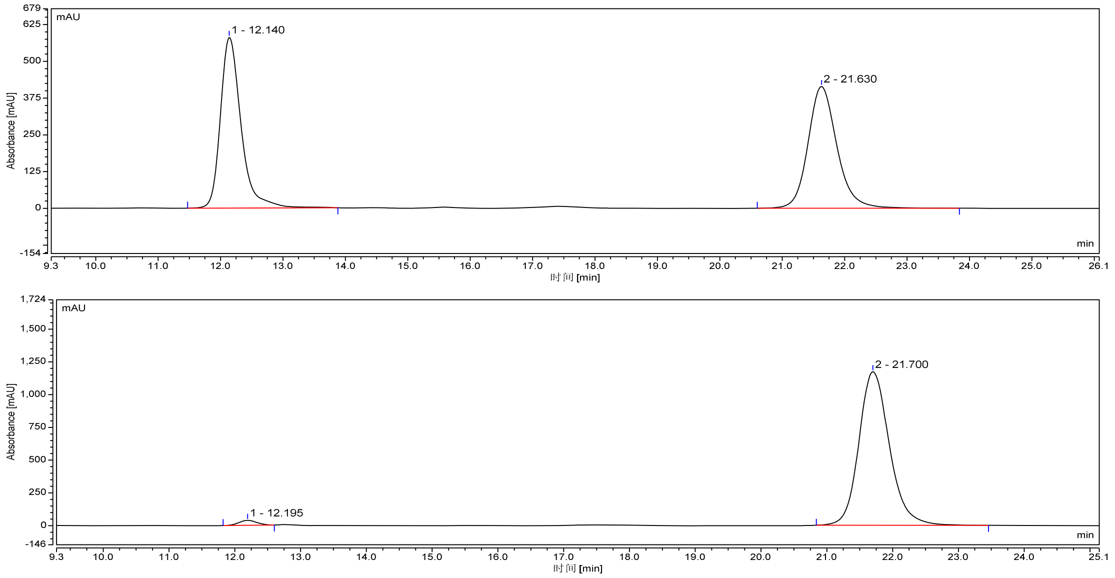

Figure S143. HPLC spectrum for **5i**, related to **Figure 5**.

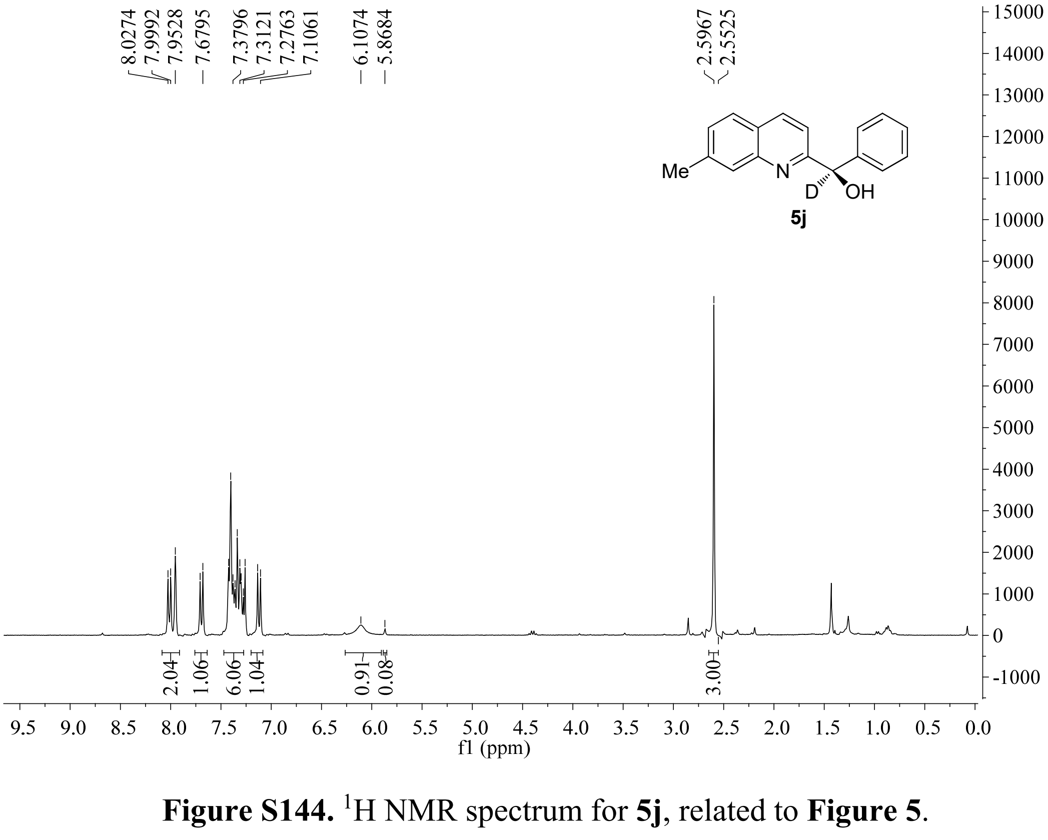

Figure S144. ^{1}H NMR spectrum for **5j**, related to **Figure 5**.

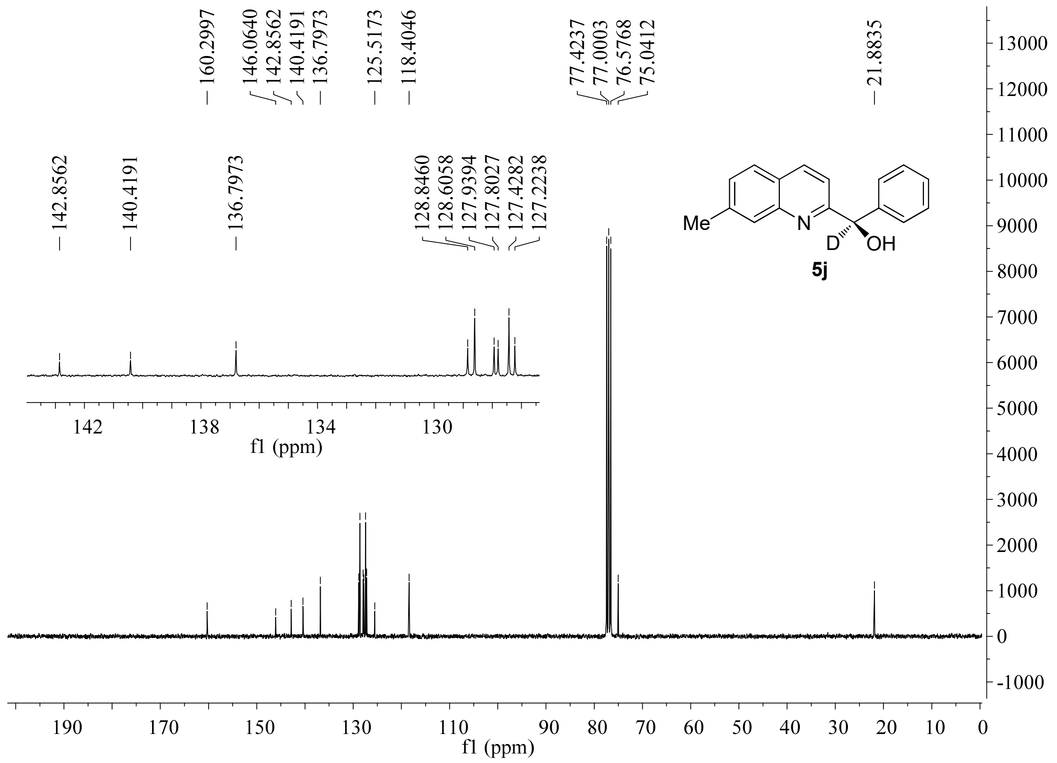

Figure S145. ^{13}C NMR spectrum for **5j**, related to **Figure 5**.

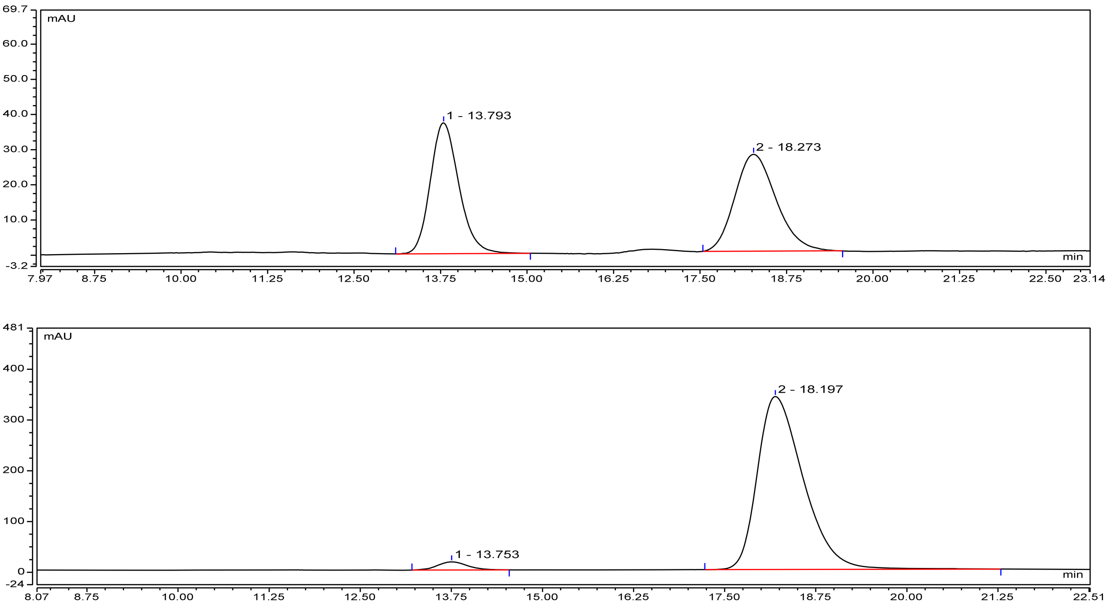

Figure S146. HPLC spectrum for **5j**, related to **Figure 5**.

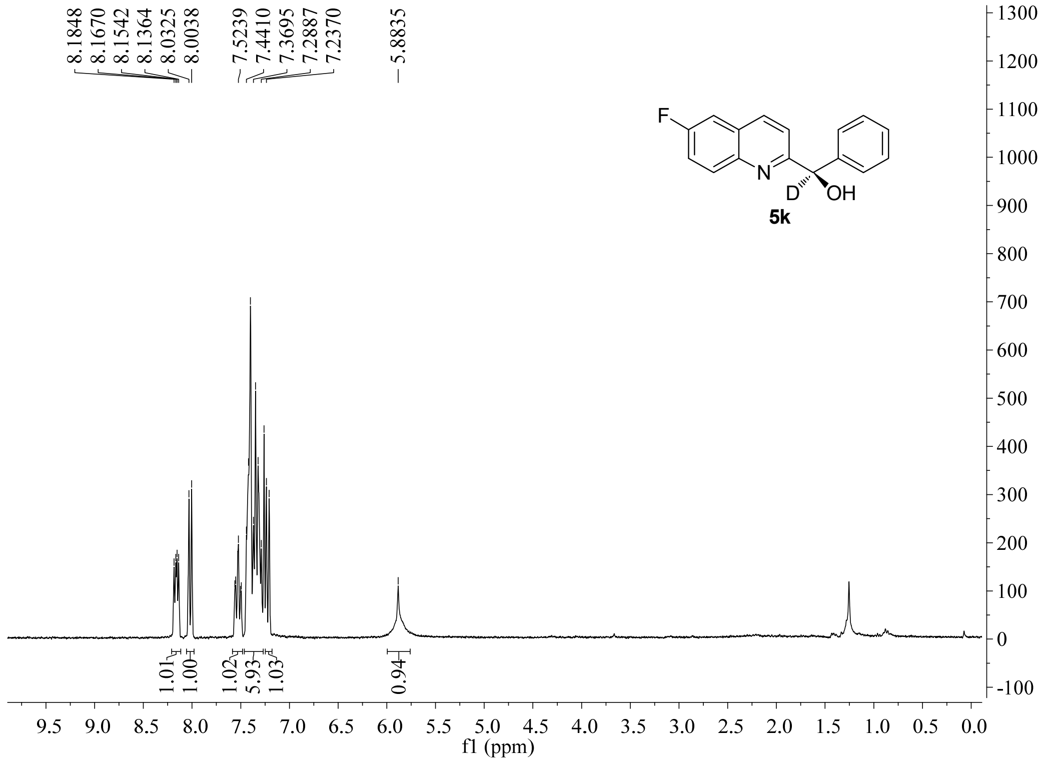

Figure S147. ^{1}H NMR spectrum for **5k**, related to **Figure 5**.

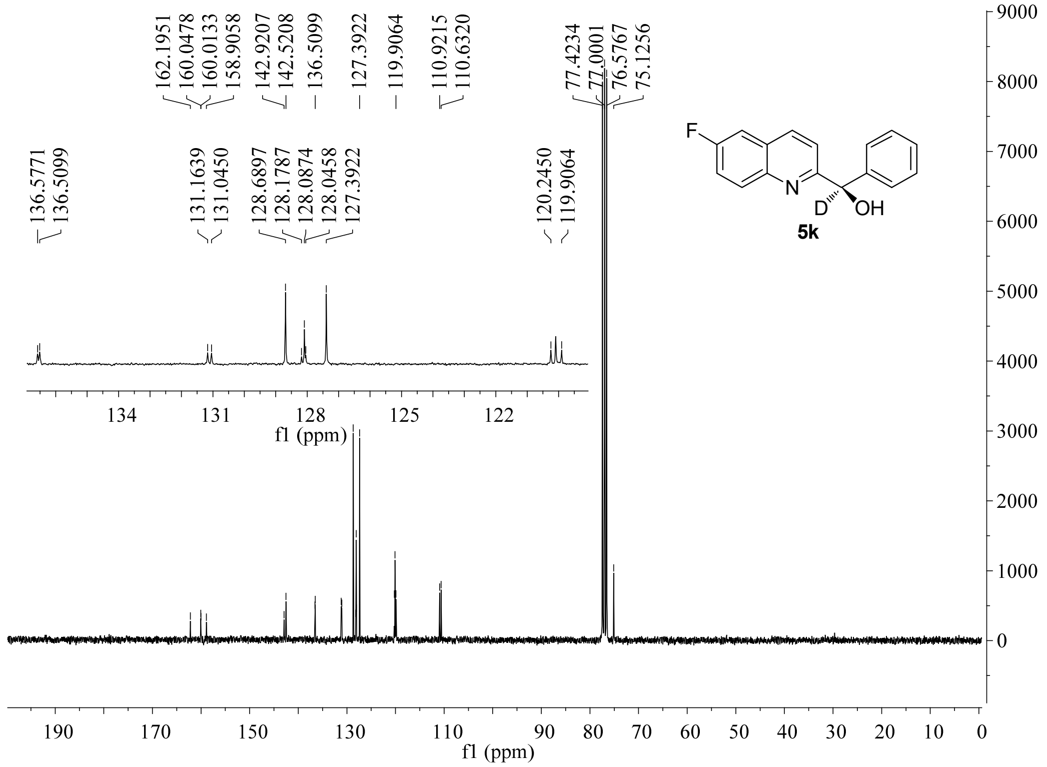

Figure S148. ^{13}C NMR spectrum for **5k**, related to **Figure 5**.

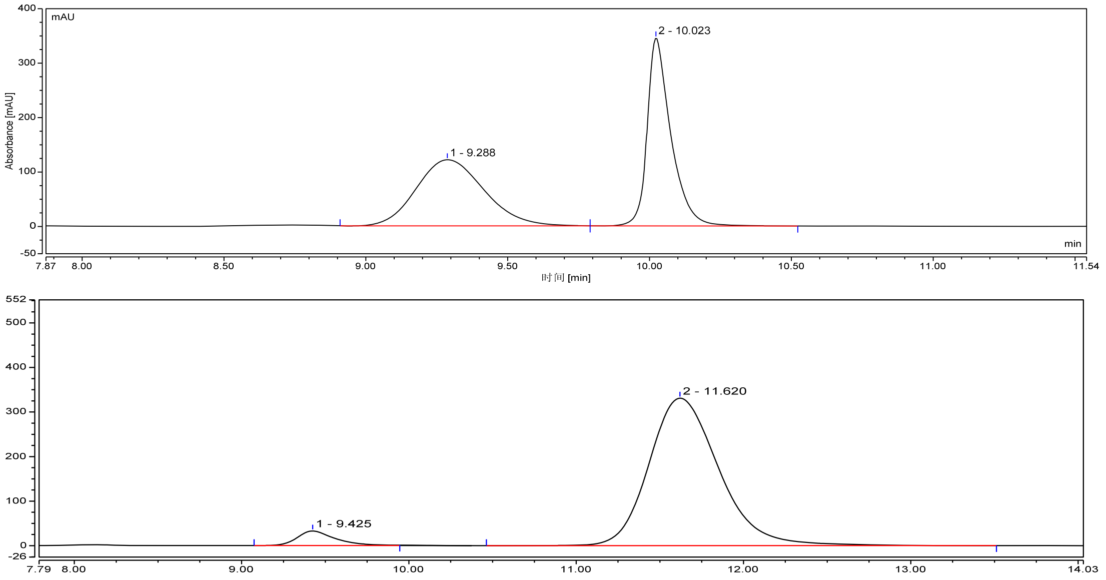

Figure S149. HPLC spectrum for **5k**, related to **Figure 5**.

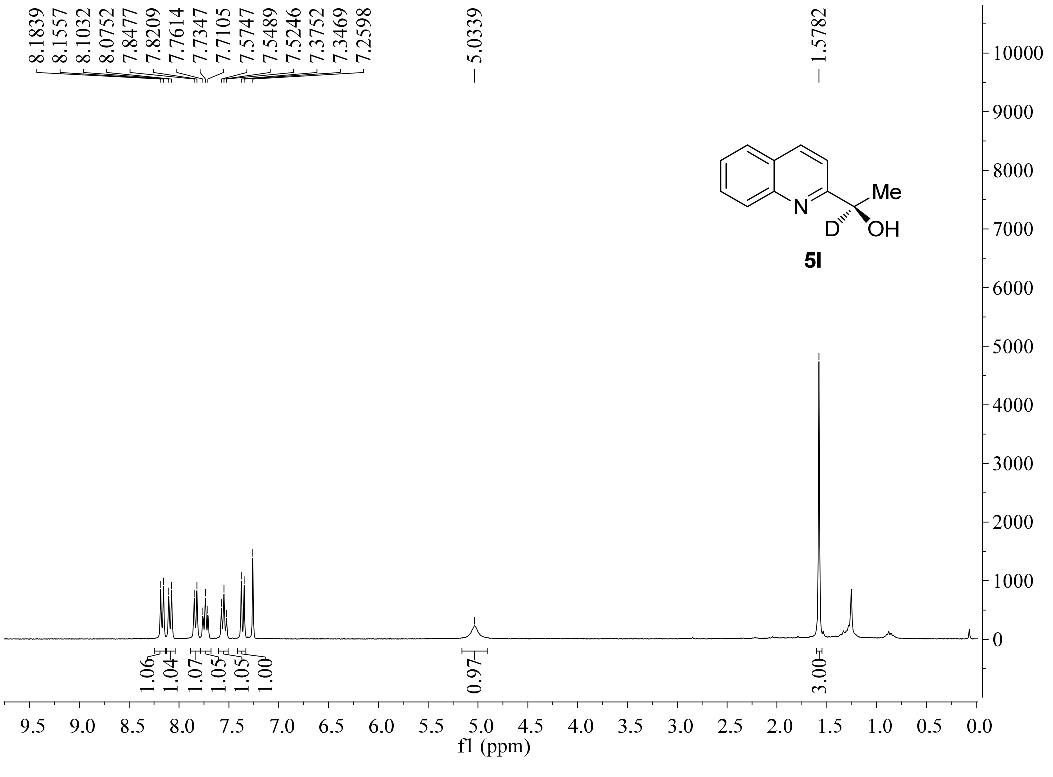

Figure S150. ^{1}H NMR spectrum for **5l**, related to **Figure 5**.

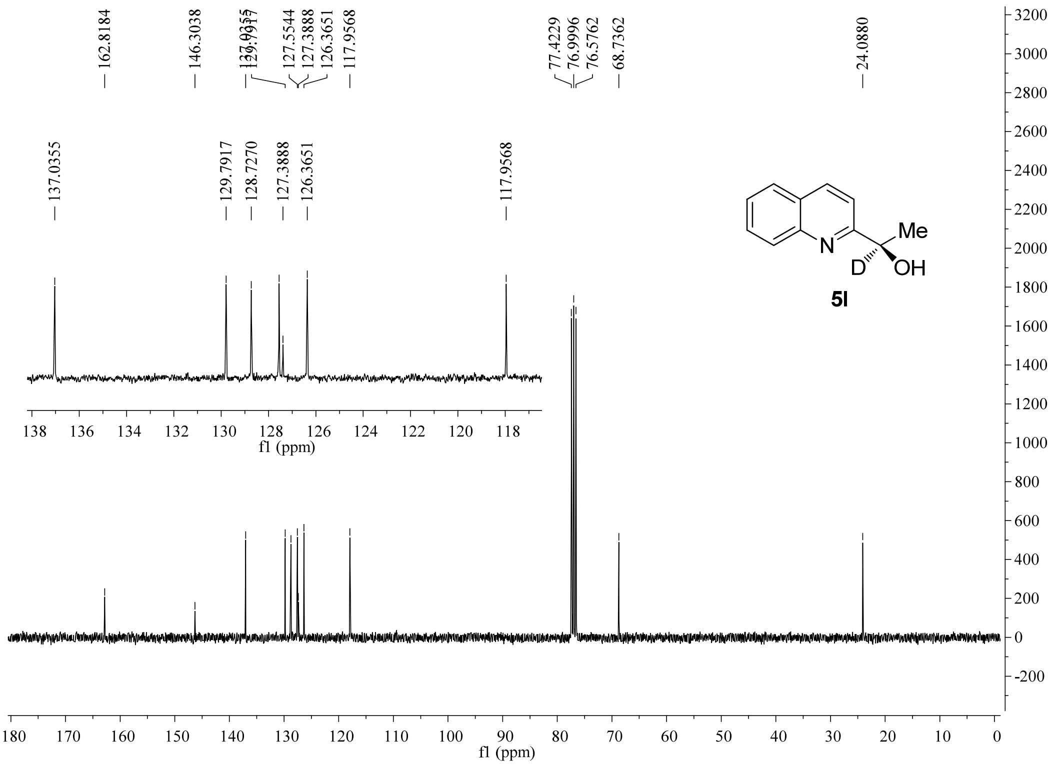

Figure S151. ^{13}C NMR spectrum for **5l**, related to **Figure 5**.

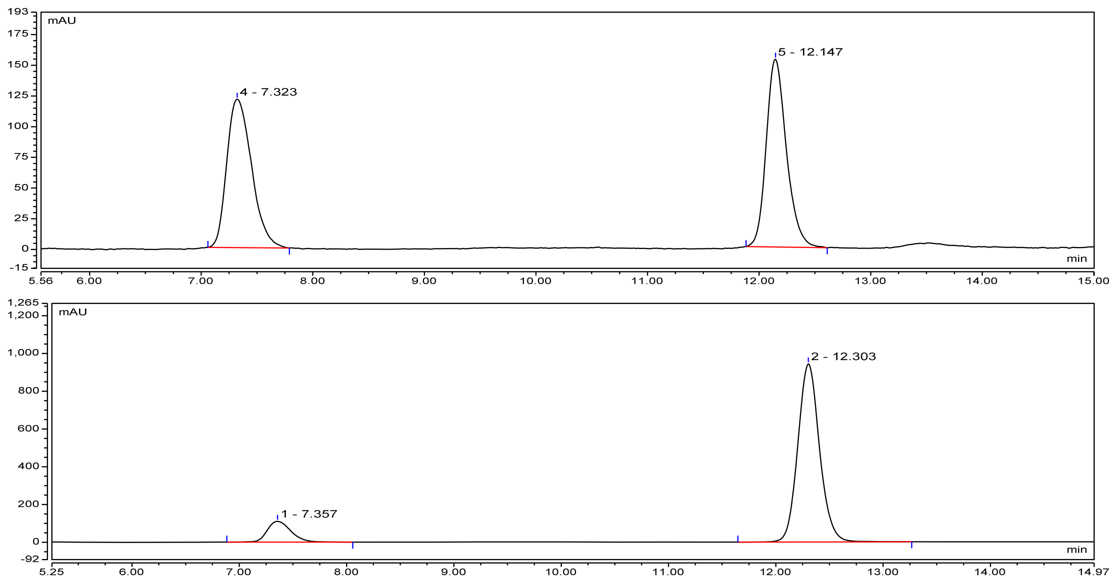

Figure S152. HPLC spectrum for **5l**, related to **Figure 5**.

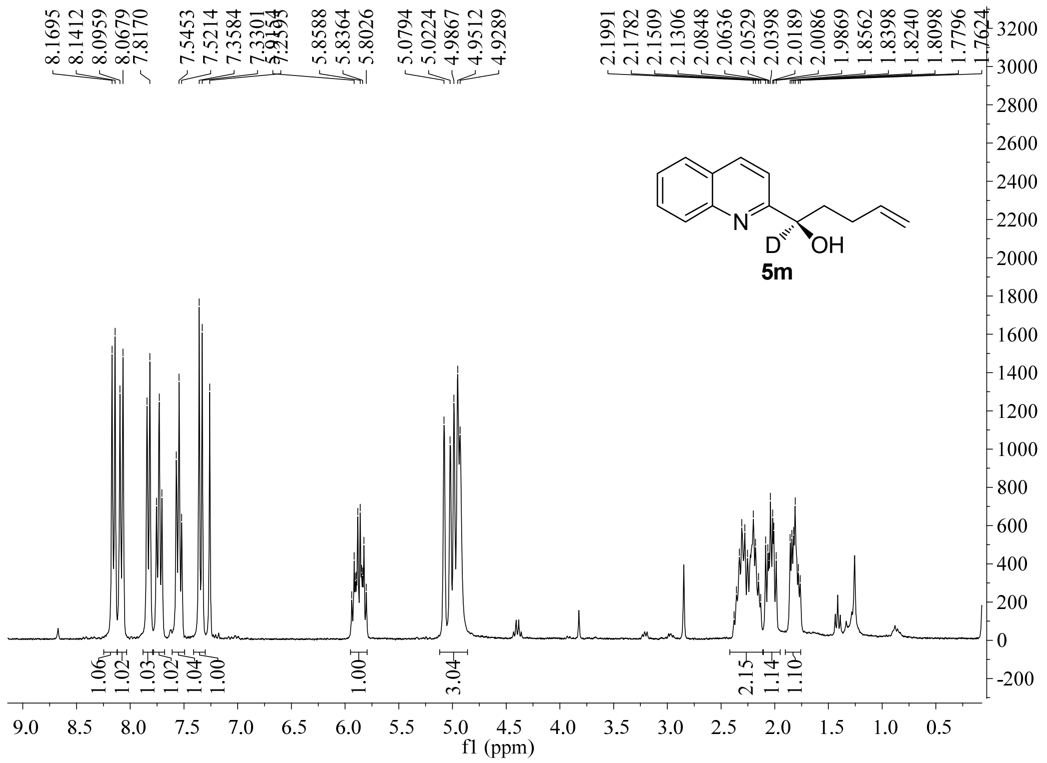

Figure S153. ^{1}H NMR spectrum for **5m**, related to **Figure 5**.

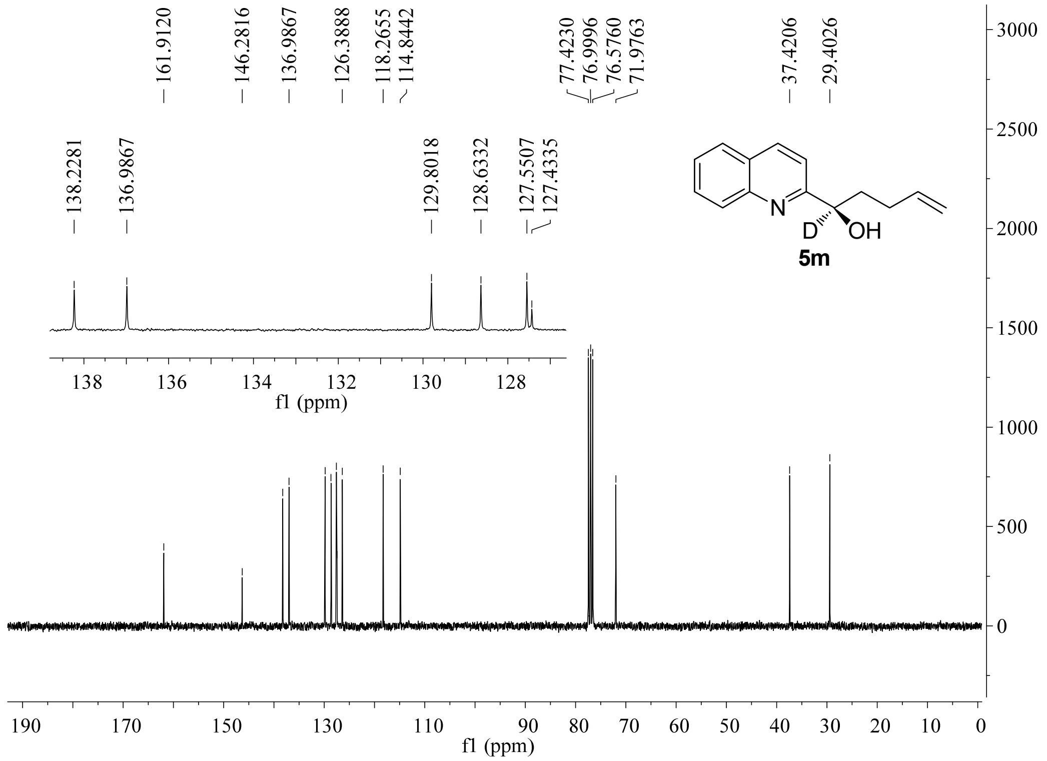

Figure S154. ^{13}C NMR spectrum for **5m**, related to **Figure 5**.

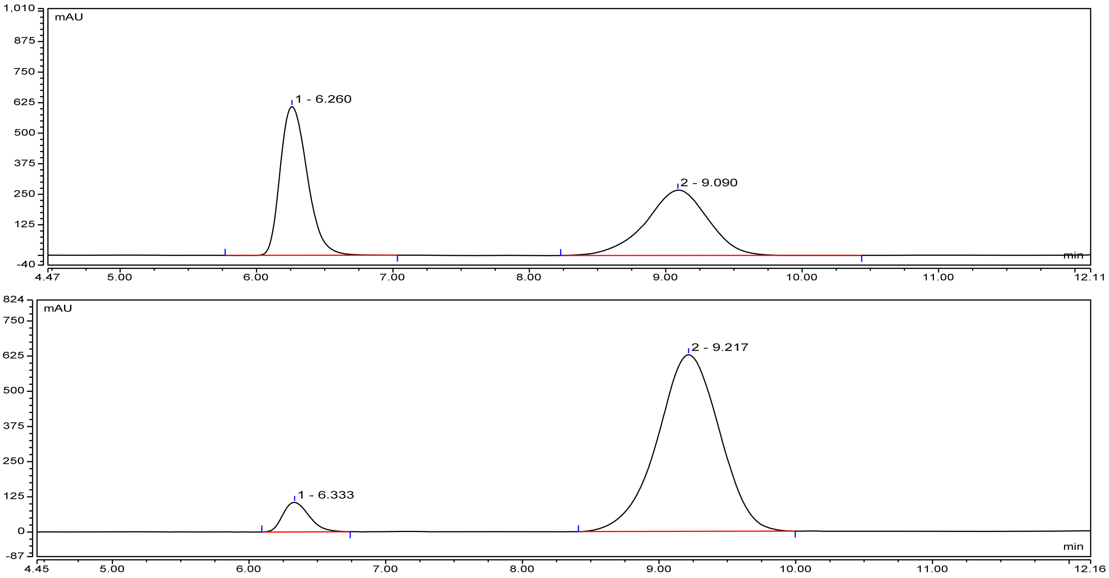

Figure S155. HPLC spectrum for **5m**, related to **Figure 5**.

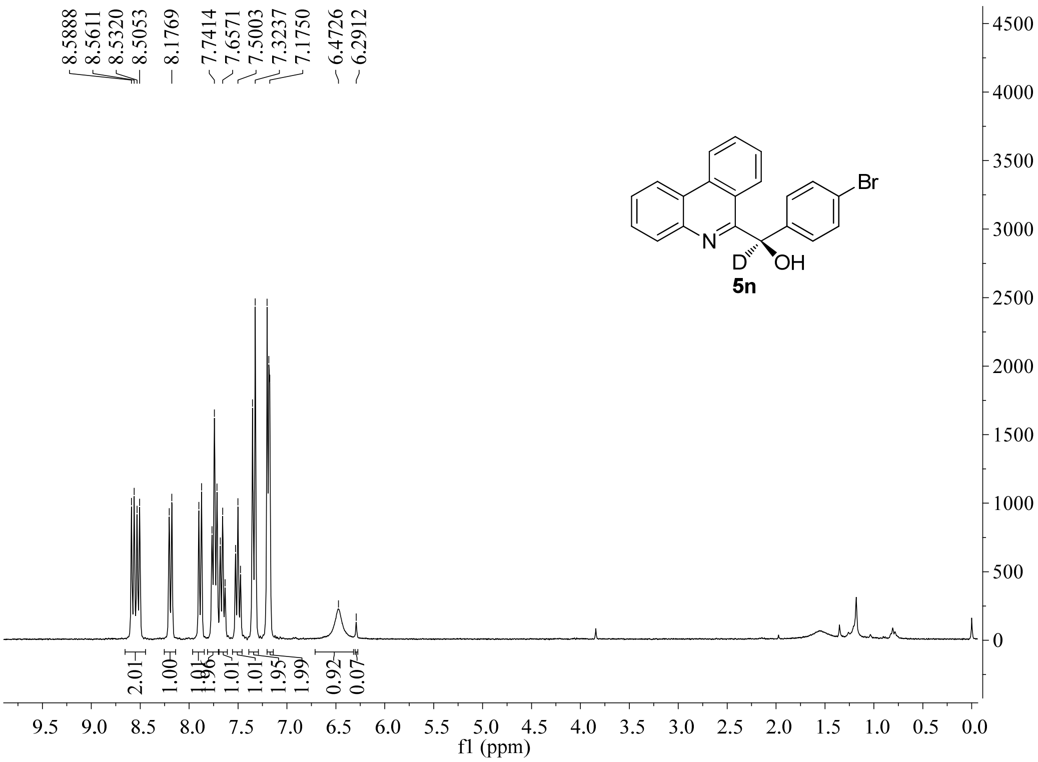

Figure S156. ^{1}H NMR spectrum for **5n**, related to **Figure 5**.

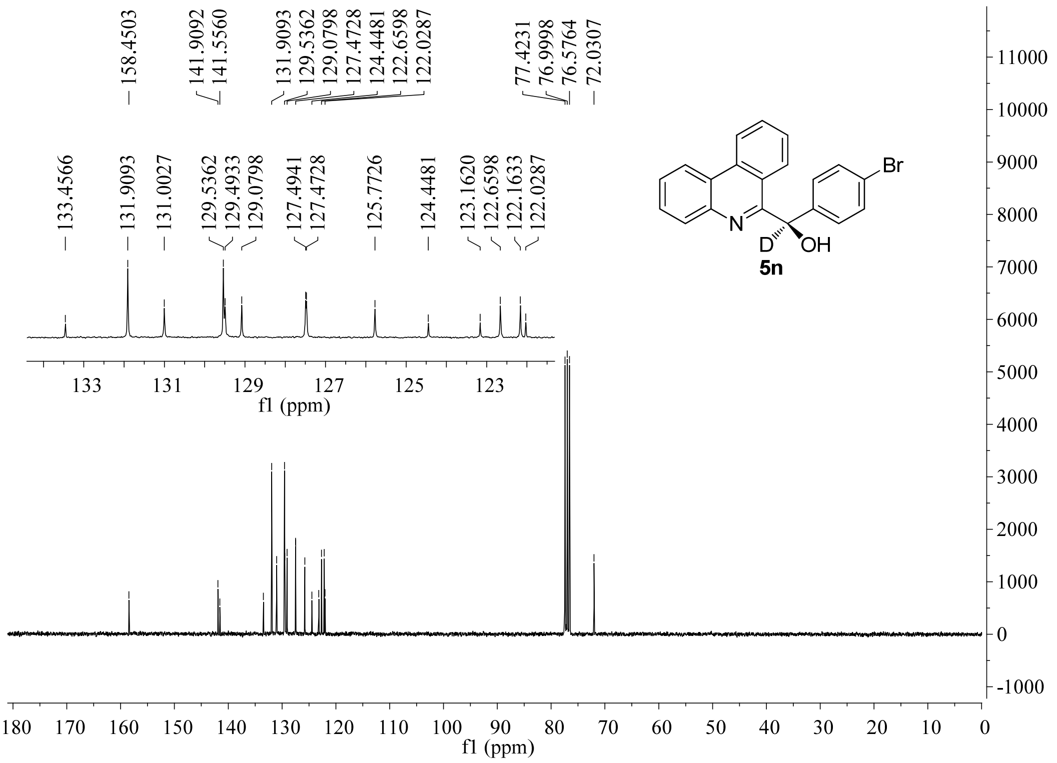

Figure S157. ^{13}C NMR spectrum for **5n**, related to **Figure 5**.

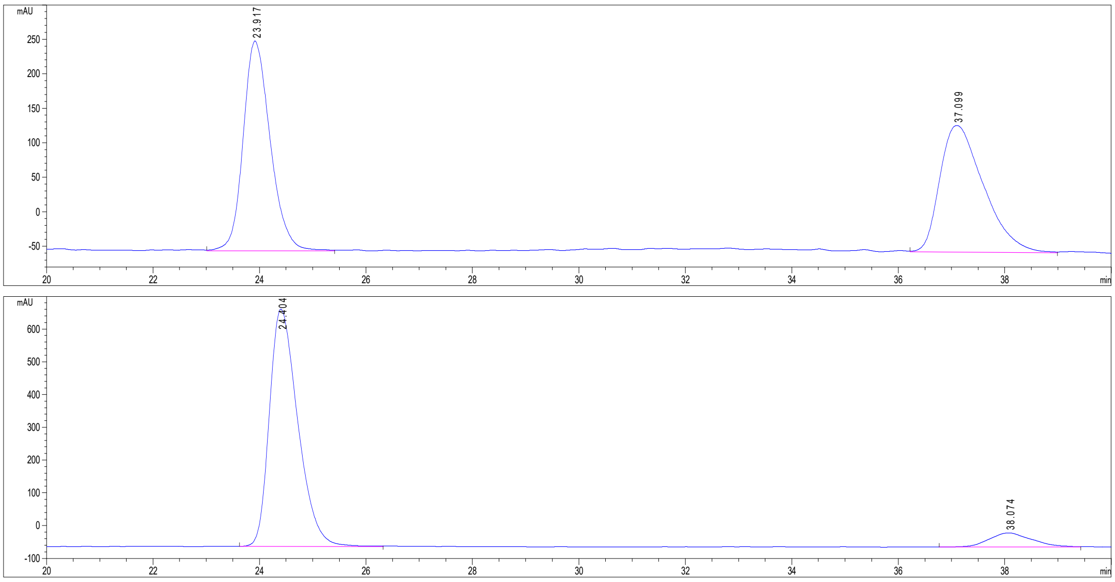

Figure S158. HPLC spectrum for **5n**, related to **Figure 5**.

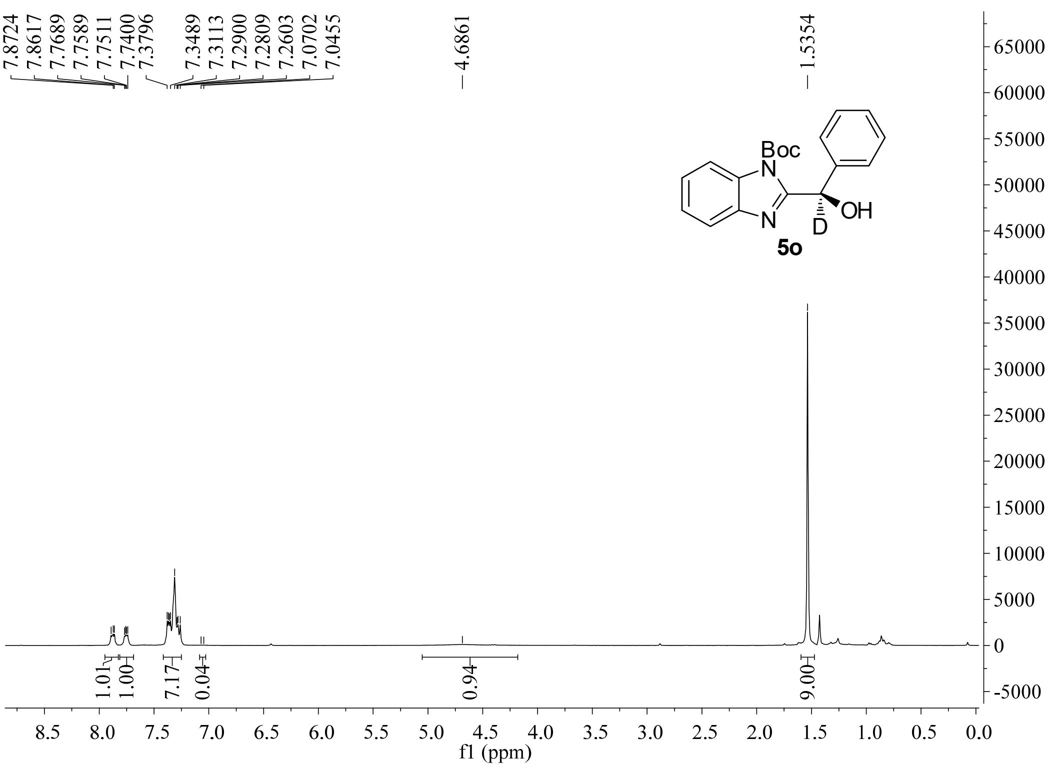

Figure S159. ^{1}H NMR spectrum for **5o**, related to **Figure 5**.

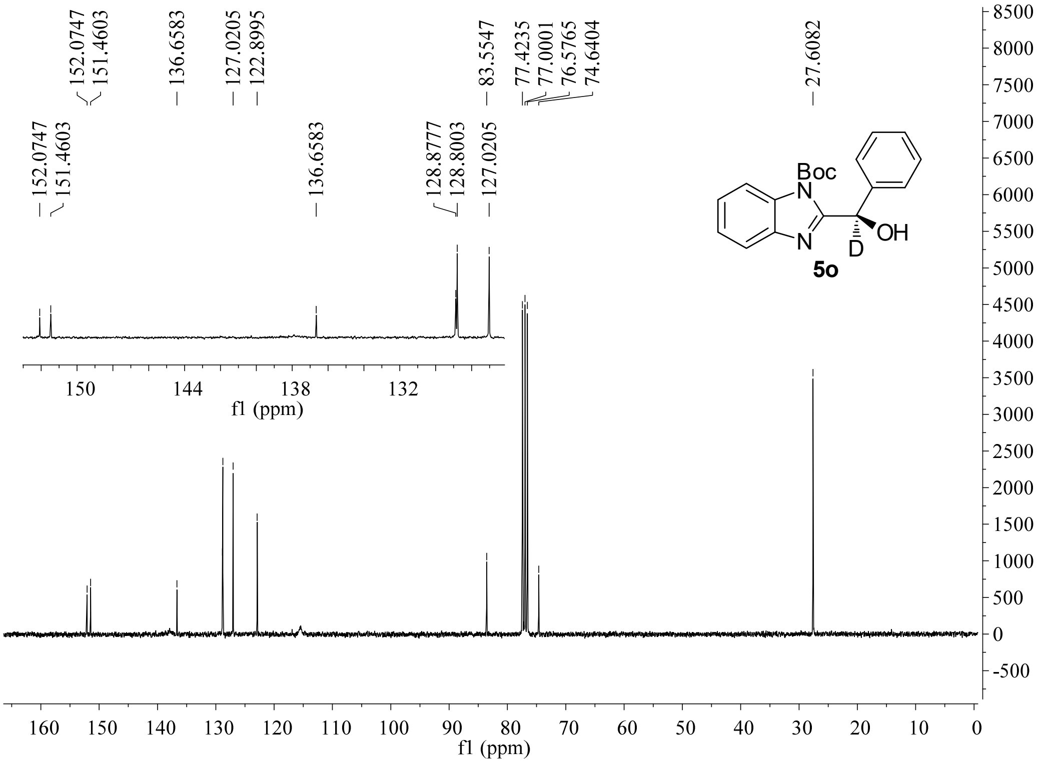

Figure S160. ^{13}C NMR spectrum for **5o**, related to **Figure 5**.

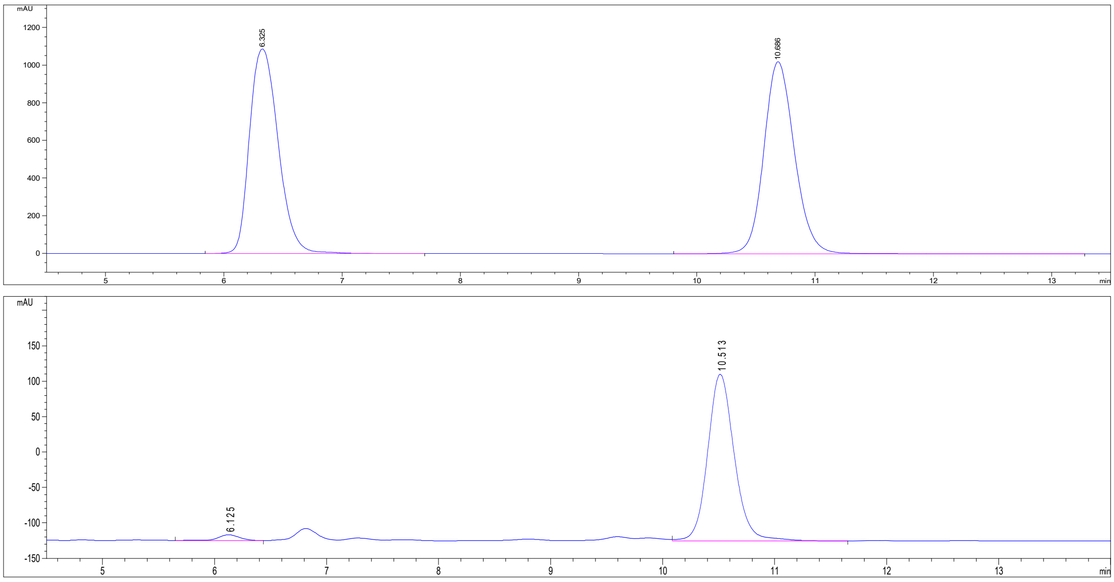

Figure S161. HPLC spectrum for **5o**, related to **Figure 5**.

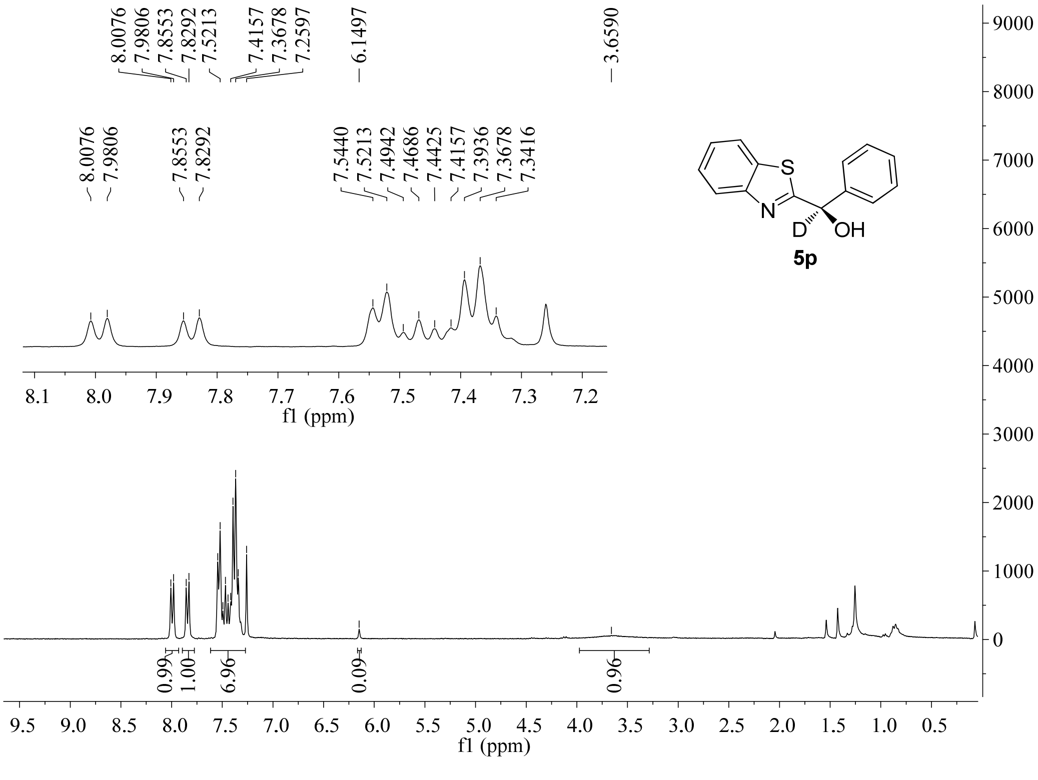

Figure S162. ^{1}H NMR spectrum for **5p**, related to **Figure 5**.

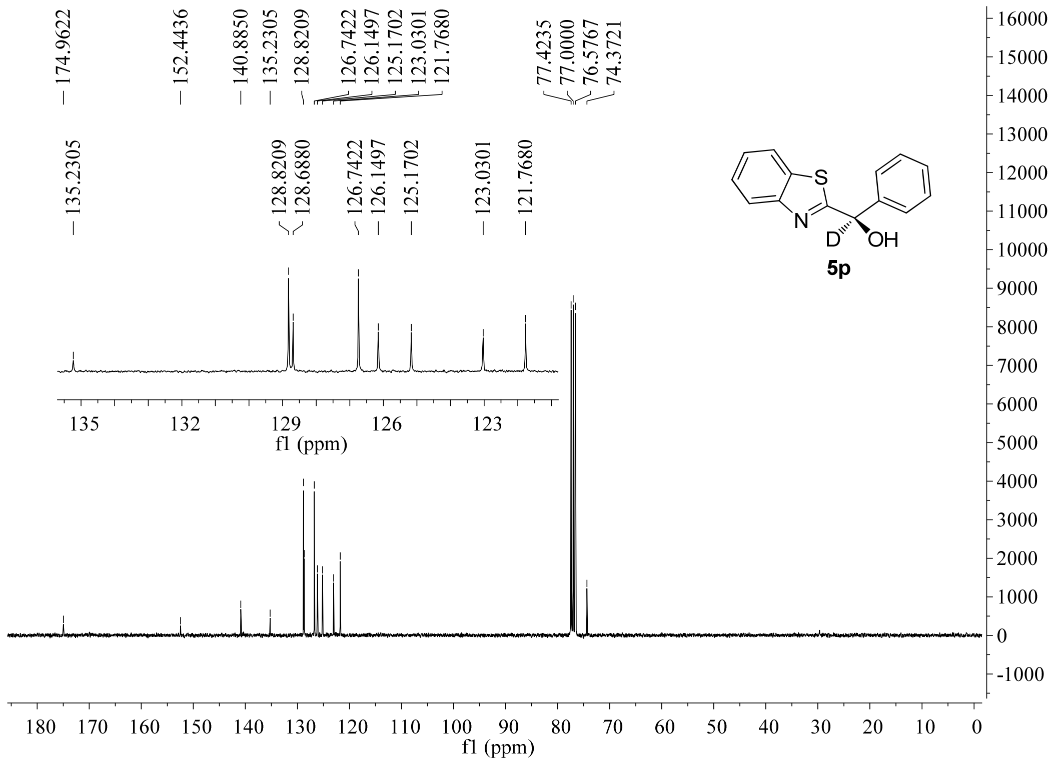

Figure S163. ^{13}C NMR spectrum for **5p**, related to **Figure 5**.

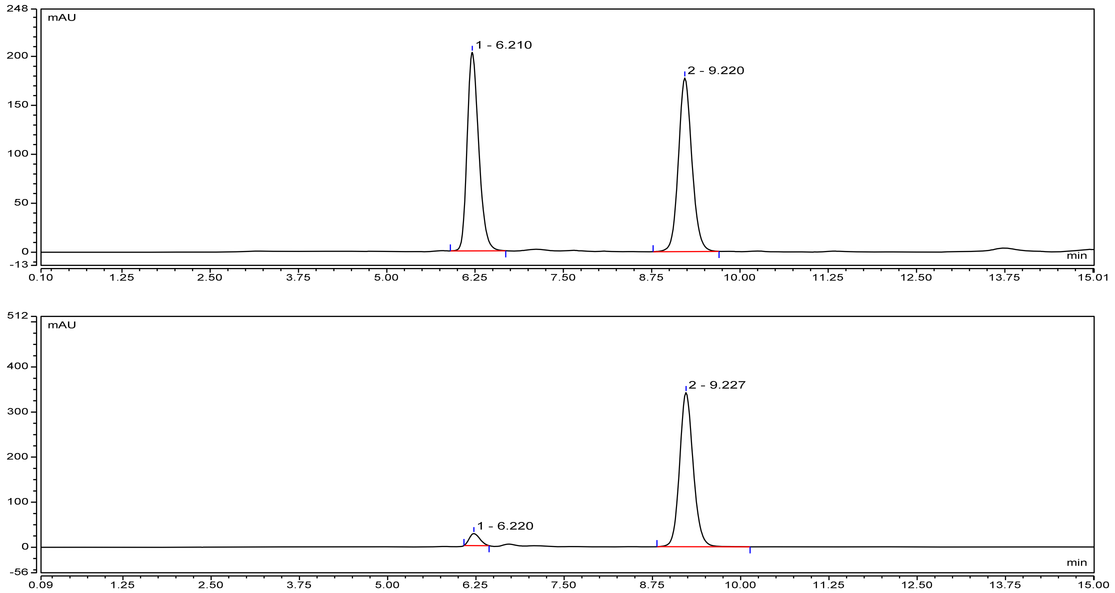

Figure S164. HPLC spectrum for **5p**, related to **Figure 5**.

Supplemental Figures for X-ray Structures of 8 and 5n

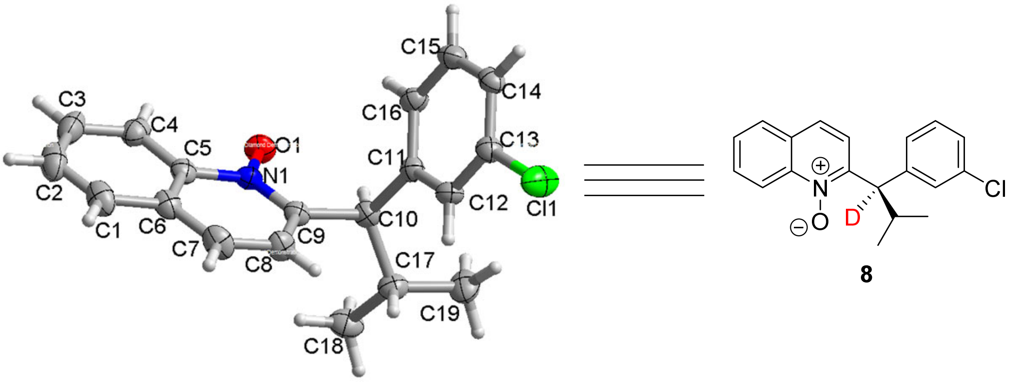

Figure S165. X-ray Structures of **8**, related to **Figure 2**.

The crystal was kept at 293(2) K during data collection. Using Olex2 (Olomanov et al., 2009), the structure was solved with the ShelXS (Sheldrick, 2008) structure solution program using Direct Methods and refined with the ShelXL (Heldrick, 2015) refinement package using Least Squares minimisation. **Crystal Data** for $C_{19}H_{18}ClNO$ (*M* =311.79 g/mol): orthorhombic, space group $P2_12_12_1$ (no. 19), *a* = 8.6267(4) Å, *b* = 11.8128(5) Å, *c* = 15.8702(8) Å, *V* = 1617.26(12) Å^3, *Z* = 4, *T* = 293(2) K, μ(CuKα) = 2.087 mm^{-1}, *Dcalc* = 1.281 g/cm^3, 5778 reflections measured ($9.332 \leq 2\Theta \leq 134.116$), 2890 unique ($R_{int}$ = 0.0306, R_{sigma} = 0.0395) which were used in all calculations. The final R_1 was 0.0408 (I > 2σ(I)) and wR_2 was 0.1044 (all data). CCDC 1893142 contains the supplementary crystallographic data for this paper.

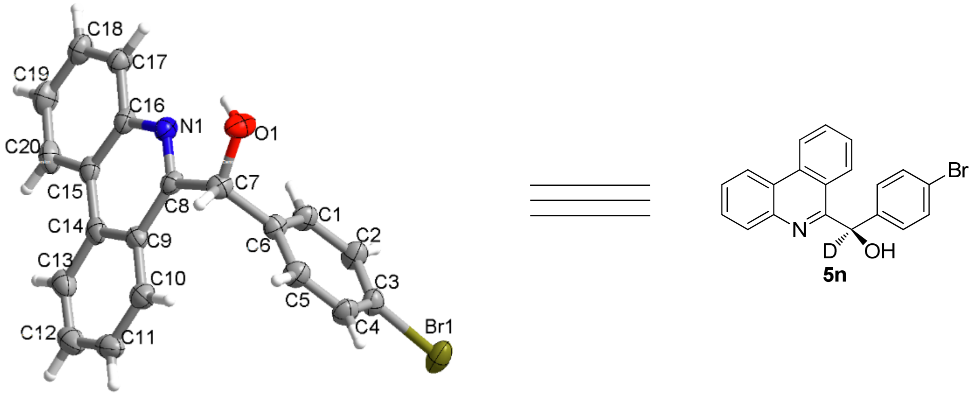

Figure S166. X-ray Structures of **5n**, related to **Figure 5**.

The crystal was kept at 293(2) K during data collection. Using Olex2, the structure was solved with the ShelXS structure solution program using Direct Methods and refined with the ShelXL refinement package using Least Squares minimisation. **Crystal Data** for $C_{20}H_{14}BrNO$ (*M* = 364.23 g/mol): orthorhombic, space group $P2_12_12_1$ (no. 19), *a* = 5.14605(13) Å, *b* =

11.5367(3) Å, c = 26.5758(5) Å, V = 1577.77(7) $Å^3$, Z = 4, T = 293(2) K, μ(CuKα) = 3.571 mm^{-1}, $Dcalc$ = 1.533 g/cm^3, 5862 reflections measured (8.356° ≤ 2Θ ≤ 141.52°), 2971 unique (R_{int} = 0.0302, R_{sigma} = 0.0405) which were used in all calculations. The final R_1 was 0.0392 (I > 2σ(I)) and wR_2 was 0.1066 (all data). CCDC 1902192 contains the supplementary crystallographic data for this paper.

Supplemental Figures for Excitation and Emission spectrums of DPZ and HE-1

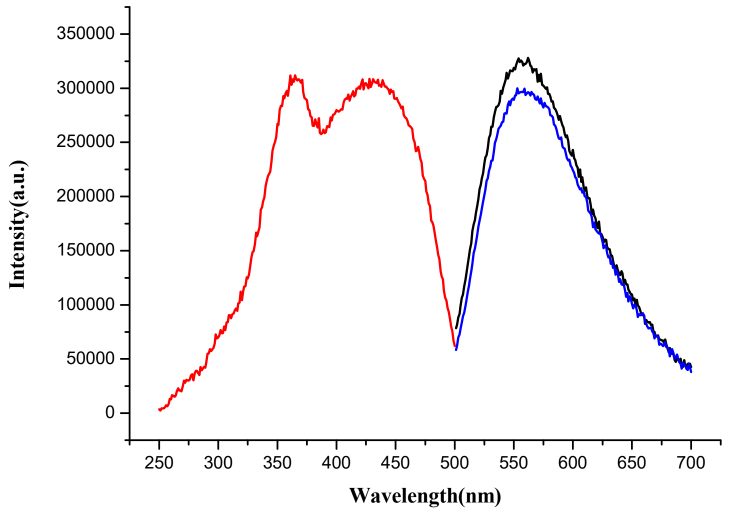

Figure S167. Excitation and emission spectrums of **DPZ**, related to **Figure 3b.**

Excitation *spectrum* was recorded on an EDINBURGH FLS 980 fluorescence spectrophotometer equipped with a monochromated 325 W Xe-arc excitation source and a visible detector (Hamamatsu R928P). Excitation spectra of **DPZ** (5.0×10^{-5} M in CH_2Cl_2) (red) and emission spectrum of **DPZ** (5.0×10^{-5} M in CH_2Cl_2) excitation wavelength as 415 nm (black) and of **DPZ** (5.0×10^{-5} M in CH_2Cl_2) excitation wavelength as 448 nm (blue) was shown in **Figure S167**.

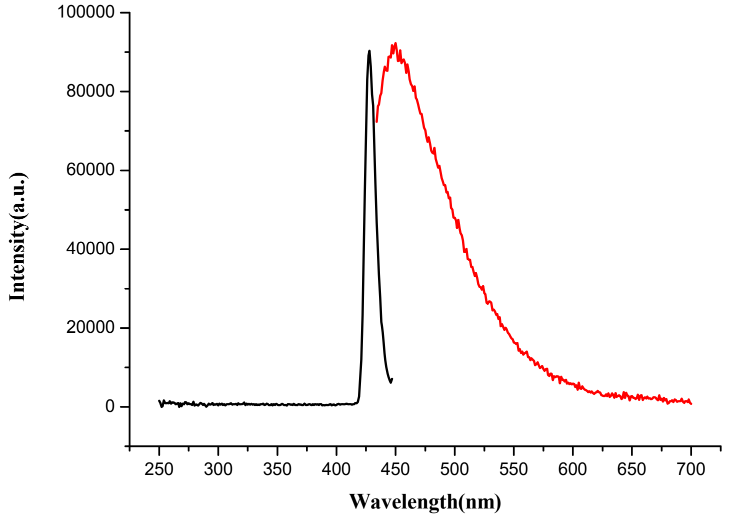

Figure S168. Excitation and emission spectrums of **HE-1**, related to **Figure 3b**.

Excitation *spectrum* was recorded on an EDINBURGH FLS 980 fluorescence spectrophotometer equipped with a monochromated 325 W Xe-arc excitation source and a visible detector (Hamamatsu R928P). The solution of **HE-1** (5.0×10^{-5} M in CH_2Cl_2) was excited at 415 nm and the emission intensity at 450 nm was observed. Based on the fluorescence spectrum of ***HE-1**, the excitation spectrum (black) and emission spectrum (red) of **HE-1** (5.0×10^{-5} M in CH_2Cl_2) was shown in **Figure S168**.

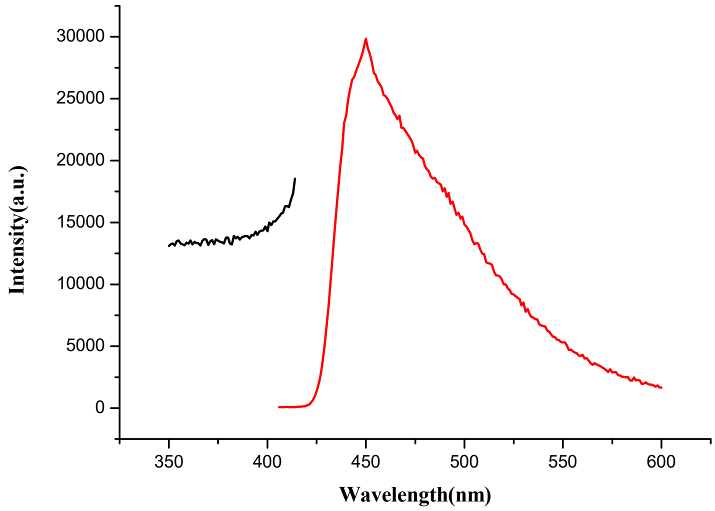

Figure S169. Excitation spectrum of **HE-1** at 380 nm, related to **Figure 3b**.

Excitation *spectrum* was recorded on an EDINBURGH FLS 980 fluorescence spectrophotometer equipped with a monochromated 325 W Xe-arc excitation source and a visible detector (Hamamatsu R928P). The solution of **HE-1** (5.0×10^{-5} M in CH_2Cl_2) was excited at 380 nm and the emission intensity at 449 nm was observed. Based on the fluorescence spectrum of ***HE-1**, the excitation spectrum (black) and emission spectrum (red) of HE-1 (5.0×10^{-5} M in CH_2Cl_2) was shown in **Figure S169**.

Supplemental Figures for the Luminescence Quenching Experiments of DPZ + HE-1, DPZ + 1a, DPZ + HE-1 + 1a, and DPZ + C21 + 1a (excitation wavelength = 448 nm)

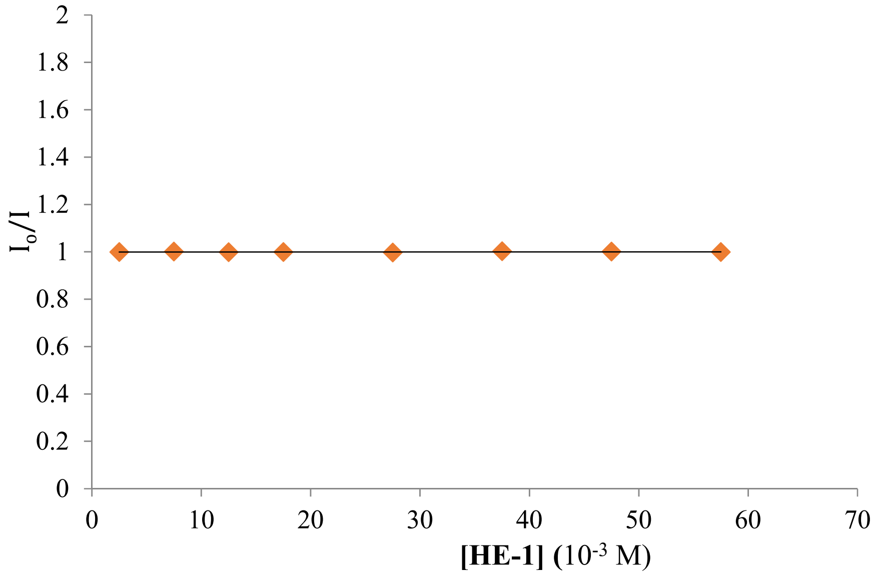

Figure S170. Stern–Volmer quenching experiment of DPZ + **HE-1** at 448 nm, related to **Figure 3b**.

Emission intensities were recorded on a spectrofluorometer. **DPZ** solution was excited at 448 nm and the emission intensity at 544 nm was observed. The appropriate amount of quencher was added to a CH_2Cl_2 solution of **DPZ** (5.0×10^{-5} M) in 3.0 mL volumetric flask under N_2. The solution was transferred to a 3.0 mL quartz cell and the emission spectrum of the sample was collected. Stern-Volmer experiment indicated that **HE-1** does not quench the luminescence of *DPZ in CH_2Cl_2 (**Figure S170**).

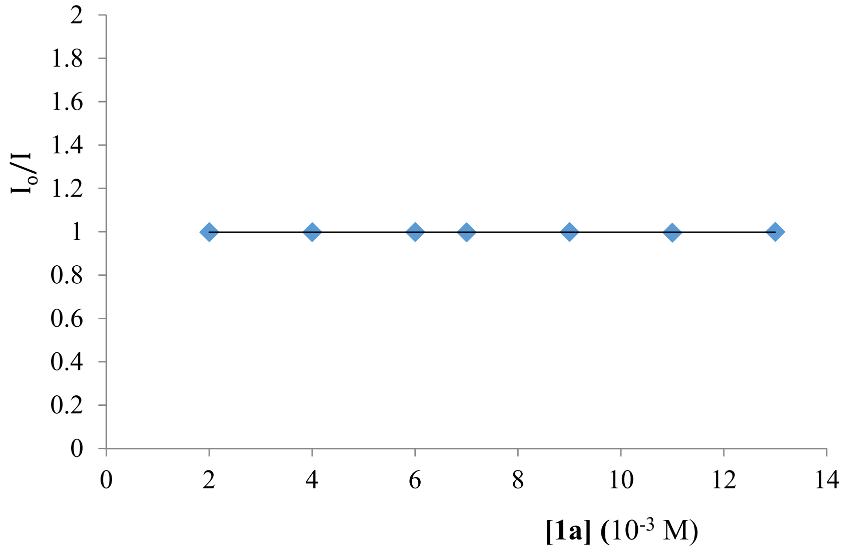

Figure S171. Stern–Volmer quenching experiment of DPZ + **1a** at 448 nm, related to **Figure 3b**.

Emission intensities were recorded on a spectrofluorometer. **DPZ** solution was excited at 448 nm and the emission intensity at 544 nm was observed. The appropriate amount of quencher was added to a CH_2Cl_2 solution of **DPZ** (5.0×10^{-5} M) in 3.0 mL volumetric flask under N_2. The solution was transferred to a 3.0 mL quartz cell and the emission spectrum of the sample was collected. Stern-Volmer experiment indicated that **1a** does not quench the luminescence of *DPZ in CH_2Cl_2 (**Figure S171**).

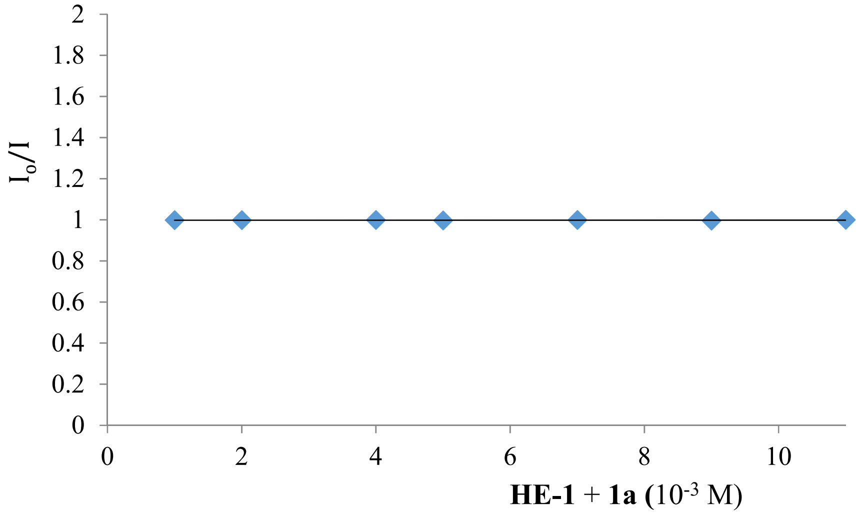

Figure S172. Stern–Volmer quenching experiment of DPZ + **HE-1**+ **1a** at 448 nm, related to **Figure 3b**.

Emission intensities were recorded on a spectrofluorometer. **DPZ** solution was excited at 448 nm and the emission intensity at 544 nm was observed. The appropriate amount of quencher was added to a CH_2Cl_2 solution of **DPZ** (5.0×10^{-5} M) in 3.0 mL volumetric flask under N_2. The solution was transferred to a 3.0 mL quartz cell and the emission spectrum of the sample was collected. Stern-Volmer experiment indicated that **HE-1**+**1a** does not quench the luminescence of *DPZ in CH_2Cl_2 (**Figure S172**).

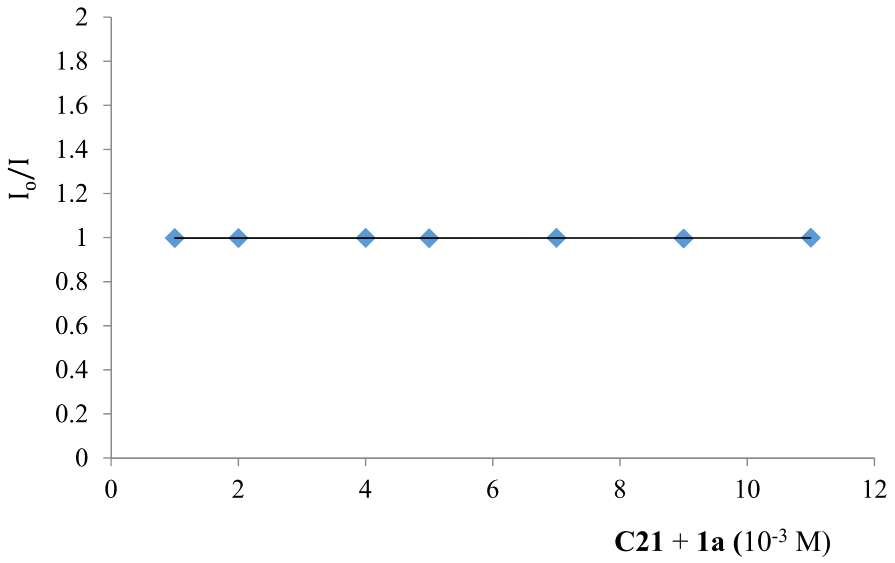

Figure S173. Stern–Volmer quenching experiment of DPZ + **C21 + 1a** at 448 nm, related to **Figure 3b**.

Emission intensities were recorded on a spectrofluorometer. **DPZ** solution was excited at 448 nm and the emission intensity at 544 nm was observed. The appropriate amount of quencher was added to a CH_2Cl_2 solution of **DPZ** (5.0×10^{-5} M) in 3.0 mL volumetric flask under N_2. The solution was transferred to a 3.0 mL quartz cell and the emission spectrum of the sample was collected. Stern-Volmer experiment indicated that **c21** + **1a** does not quench the luminescence of *DPZ in CH_2Cl_2 (**Figure S173**).

Supplemental Figures for the Luminescence Quenching Experiments of DPZ + HE-1, HE-1 + 1a, and DPZ + 1a (excitation wavelength = 415 nm)

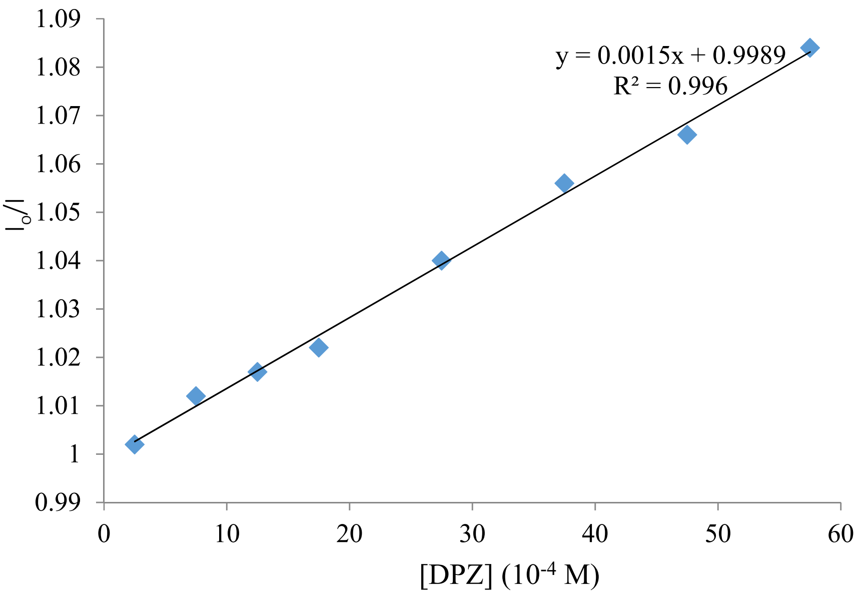

Figure S174. Stern–Volmer quenching experiment of DPZ + **HE-1** at 415 nm, related to **Figure 3b**.

Emission intensities were recorded on a spectrofluorometer. DPZ solution was excited at 415 nm and the emission intensity at 546 nm was observed. The appropriate amount of quencher was added to a CH_2Cl_2 solution of **DPZ** (5.0×10^{-5} M) in 3.0 mL volumetric flask under N_2. The solution was transferred to a 3.0 mL quartz cell and the emission spectrum of the sample was collected. Stern-Volmer experiment indicated that **HE-1** quenches the luminescence of *DPZ in CH_2Cl_2 (**Figure S174**).

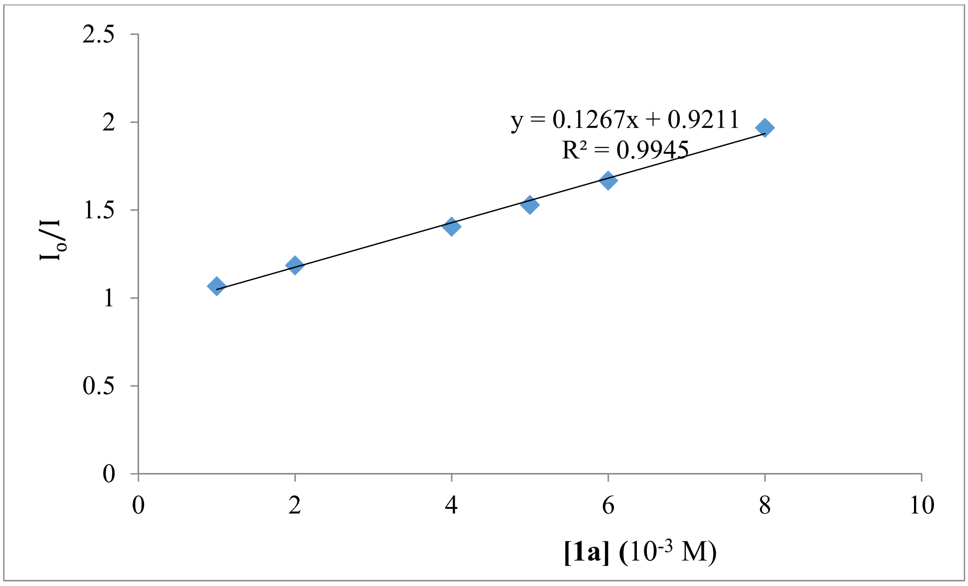

Figure S175. Stern–Volmer quenching experiment of **HE-1** + **1a** at 415 nm, related to **Figure 3b**.

Emission intensities were recorded on a spectrofluorometer. **HE-1** solution was excited at 230 nm and the emission intensity at 415 nm was observed. The appropriate amount of quencher was added to a CH_2Cl_2 solution of **HE-1** (5.0×10^{-3} M) in 3.0 mL volumetric flask under N_2. The solution was transferred to a 2.0 mL quartz cell and the emission spectrum of the sample was collected (**Figure S175**).

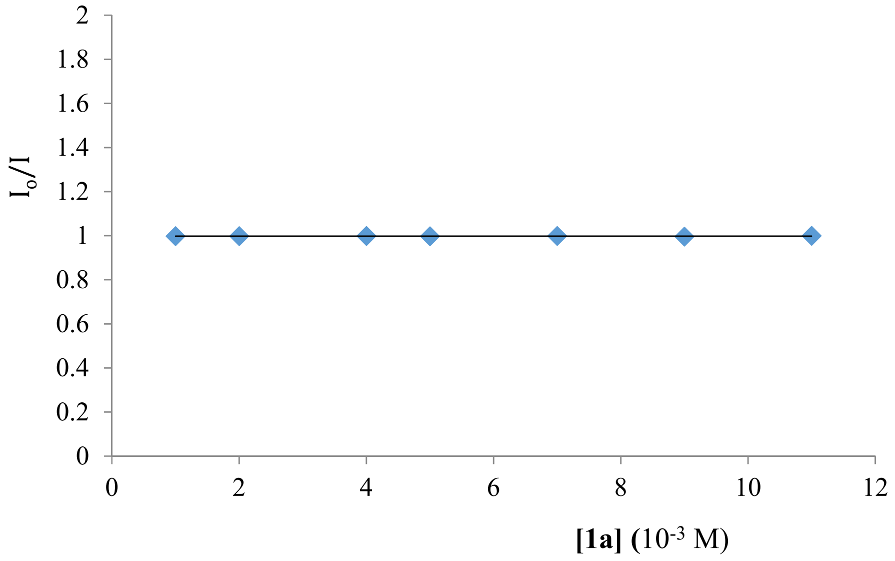

Figure S176. Stern–Volmer quenching experiment of **DPZ** + **1a** at 415 nm, related to **Figure 3b**.

Emission intensities were recorded on a spectrofluorometer. **DPZ** solution was excited at 230 nm and the emission intensity at 415 nm was observed. A solution of **1a** (5.0×10^{-3} M) in CH_2Cl_2 was added to the appropriate amount of quencher in 3.0 mL volumetric flask under N_2. The solution was transferred to a 2.0 mL quartz cell and the emission spectrum of the sample was collected (**Figure S176**).

Supplemental Figures for the Emission spectrums of the blue LED light

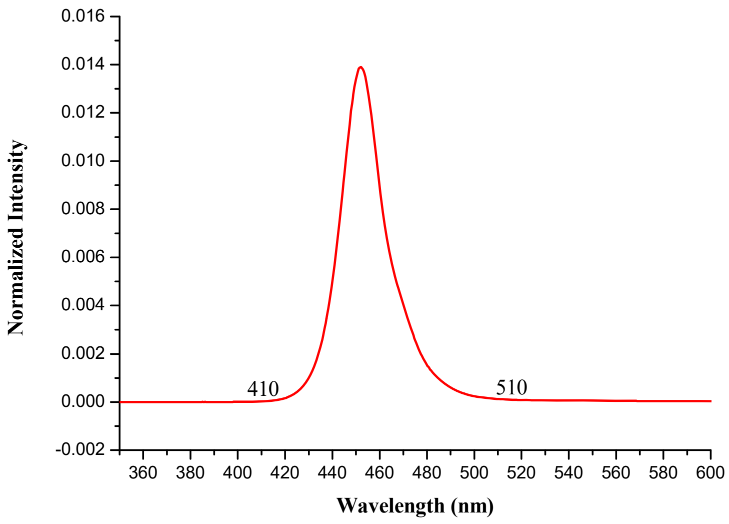

Figure S177. Emission spectrum of the used 3 W blue LED light, related to **Figure 3b**.

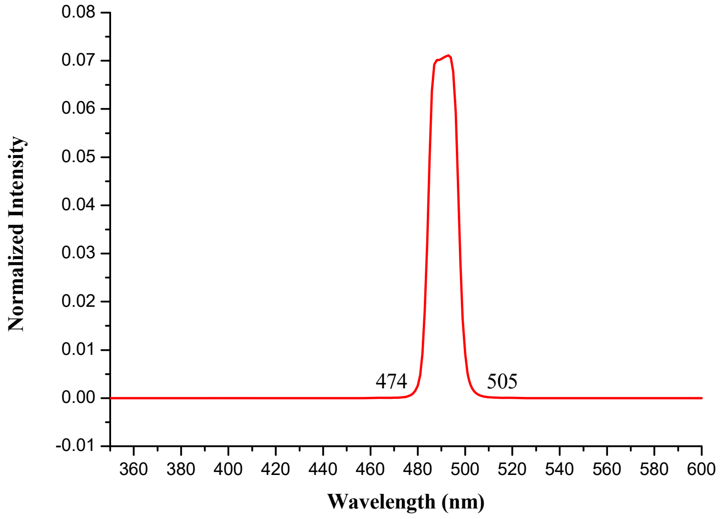

Figure S177. Emission spectrum of the LED light with the laser line filter *(CWL = 490 nm)*, related to **Figure 3b**.

Supplemental Figures for the Cyclic Voltammetry spectrums of ferrocene, 1a, 1ee, 1ff, and 4a

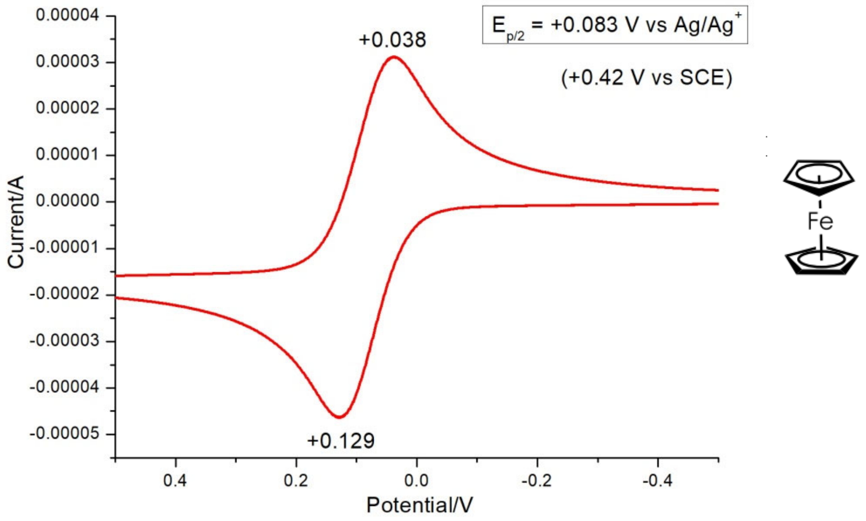

Figure S178. Cyclic voltammogram of ferrocene in MeCN referenced to Ag/$AgNO_3$, related to **Figure 3b**.

Electrochemical potentials were obtained with a standard set of conditions to main internal consistency. Cyclic voltammograms were collected with a potentiostat. Samples were prepared with 0.01 mmol of ferrocene in 10 mL anhydrous acetonitrile. Measurements employed a radium glassy carbon working electrode, platinum wire counter electrode, 0.1 M [Bu_4N][PF_6] in acetonitrile, 0.01 M silver-silver nitrate reference electrode. The obtained value was converted to SCE by adding 0.337 V.

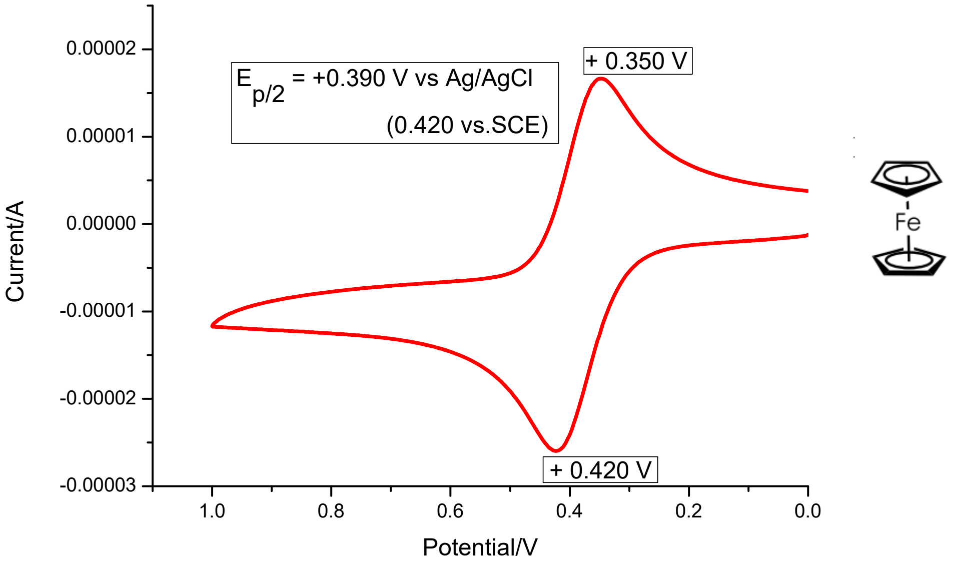

Figure S179. Cyclic voltammogram of ferrocene in MeCN referenced to Ag/AgCl, related to **Figure 3b**.

Electrochemical potentials were obtained with a standard set of conditions to main internal consistency. Cyclic voltammograms were collected with a potentiostat. Samples were prepared with 0.01 mmol of ferrocene in 10 mL anhydrous acetonitrile. Measurements employed a radium glassy carbon working electrode, platinum wire counter electrode, 0.1 M $[Bu_4N][PF_6]$ in acetonitrile, 0.01 M silver-silver chloride reference electrode. The obtained value was converted to SCE by adding 0.03 V.

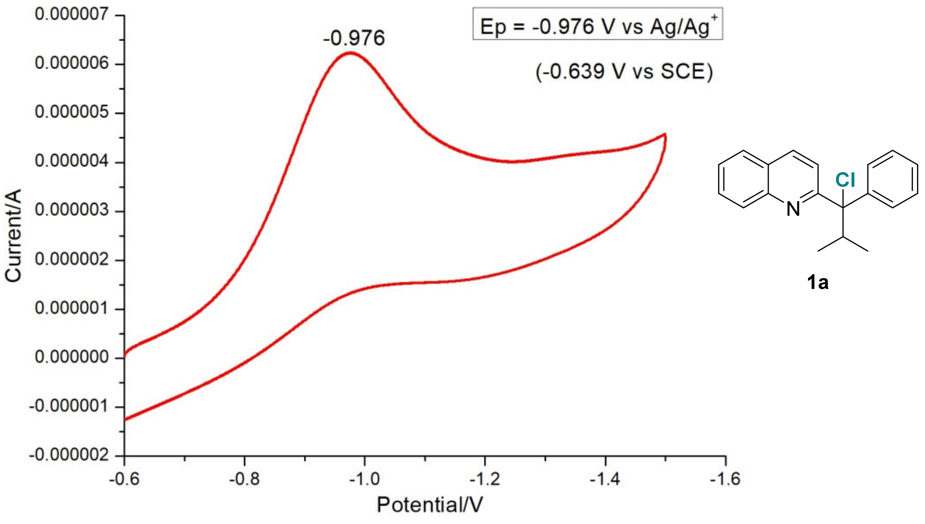

Figure S180. Cyclic voltammogram of **1a** in MeCN, related to **Figure 3b**.

Electrochemical potentials were obtained with a standard set of conditions to main internal consistency. Cyclic voltammograms were collected with a potentiostat. Samples were prepared with 0.01 mmol of **1a** in 10 mL anhydrous acetonitrile. Measurements employed a radium glassy carbon working electrode, platinum wire counter electrode, 0.1 M [Bu_4N][PF_6] in acetonitrile, 0.01 M silver-silver nitrate reference electrode. The value of **1a** converted to SCE was E_p = –0.639 V vs SCE.

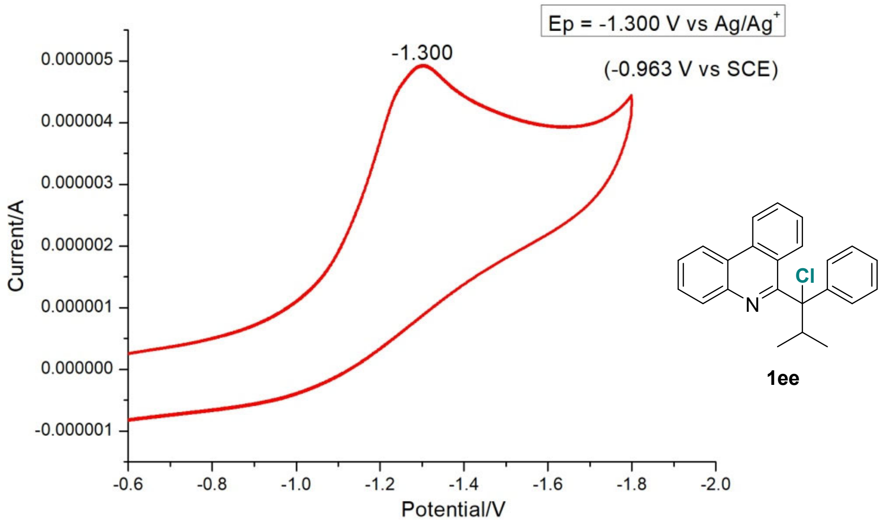

Figure S181. Cyclic voltammogram of **1ee** in MeCN, related to **Figure 3b**.

Electrochemical potentials were obtained with a standard set of conditions to main internal consistency. Cyclic voltammograms were collected with a potentiostat. Samples were prepared with 0.02 mmol of **1ee** in 10 mL anhydrous acetonitrile. Measurements employed a radium glassy carbon working electrode, platinum wire counter electrode, 0.1 M [Bu_4N][PF_6] in acetonitrile, 0.01 M silver-silver nitrate reference electrode. The obtained value was referenced to $Ag/AgNO_3$. The value of **1ee** converted to SCE was E_p = –0.963 V vs SCE.

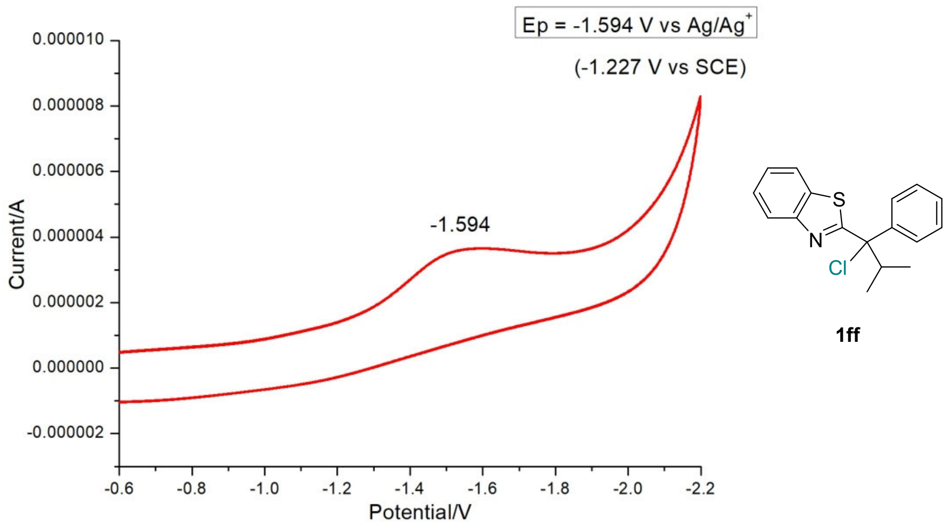

Figure S182. Cyclic voltammogram of **1ff** in MeCN, related to **Figure 3b**.

Electrochemical potentials were obtained with a standard set of conditions to main internal consistency. Cyclic voltammograms were collected with a potentiostat. Samples were prepared with 0.01 mmol of **1ff** in 10 mL anhydrous acetonitrile. Measurements employed a radium glassy carbon working electrode, platinum wire counter electrode, 0.1 M $[Bu_4N][PF_6]$ in acetonitrile, 0.01 M silver-silver nitrate reference electrode. The obtained value was referenced to $Ag/AgNO_3$. The value of **1ff** converted to SCE was $E_p = –1.227$ V vs SCE.

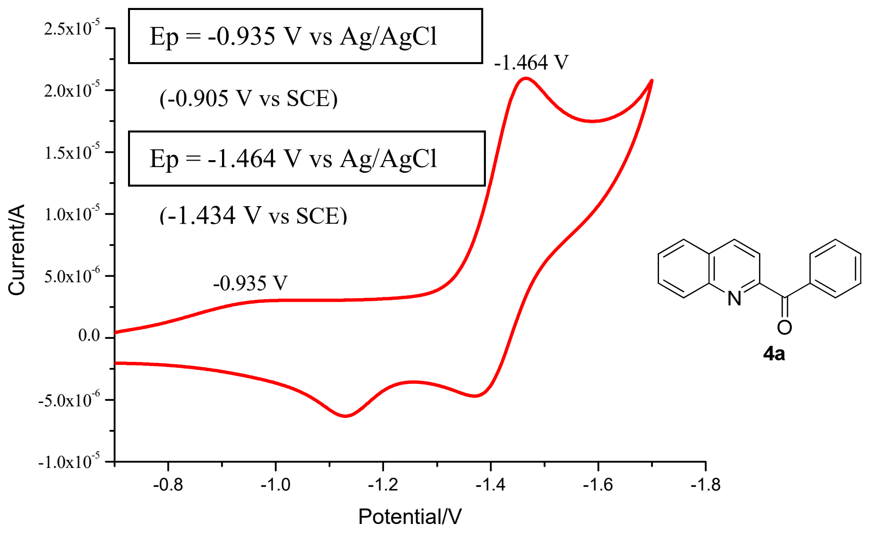

Figure S183. Cyclic voltammogram of **4a** in MeCN, related to **Figure 3b**.

Cyclic voltammetry experiments were performed on a CHI600E Workstation. Measurements were performed for anhydrous acetonitrile solutions ([**4a**] = 1.0 mM, [$(NBu_4)PF_6$] = 0.10 M) with a radium glassy carbon (working electrode) and platinum wire (counter electrode), and a Ag/AgCl reference electrode under N_2 at room temperature. The scan rate was 50 mV/s. Ferrocene (Cp_2Fe) was used as a reference. The value of **4a** converted to SCE was E_{p1} = –0.905 V vs SCE, E_{p2} = –0.935 V vs SCE.

DFT calculations

All the DFT calculations were performed with the Gaussian 09 program (Frisch et al., 1993). The geometries optimizations were performed at the B3LYP/6-31G(d) level. The vibrational frequencies were computed at the same level to check whether each optimized structure is an energy minimum or a transition state (TS) and to evaluate its thermal corrections at 298 K. The Gibbs free energies (ΔG) are used to discuss the reaction.

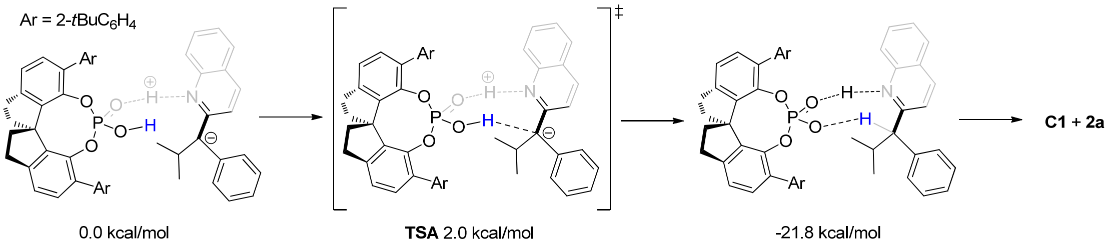

Figure S184. The calculated ΔG values (in kcal/mol) for the formation of (*R*)-**2a** through **TSA** transition state. The ΔG for the generation of **TSA** transition state are 2.0 kcal/mol and The ΔG of the formation of **C1** and **2a** are -21.8 kcal/mol, related to **Figures 3 and 4**.

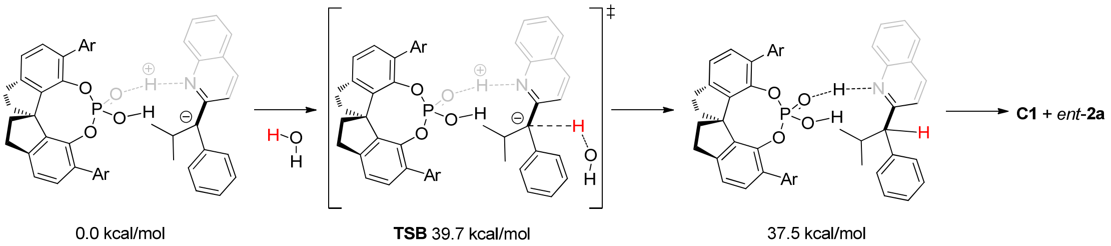

Figure S185. The calculated ΔG values (in kcal/mol) for the formation of (*ent*)-**2a** through **TSB** transition state. The ΔG for the generation of **TSB** transition state are 39.7 kcal/mol and The ΔG of the formation of **C1** and (*ent*)-**2a** are 37.5 kcal/mol, related to **Figures 3 and 4**.

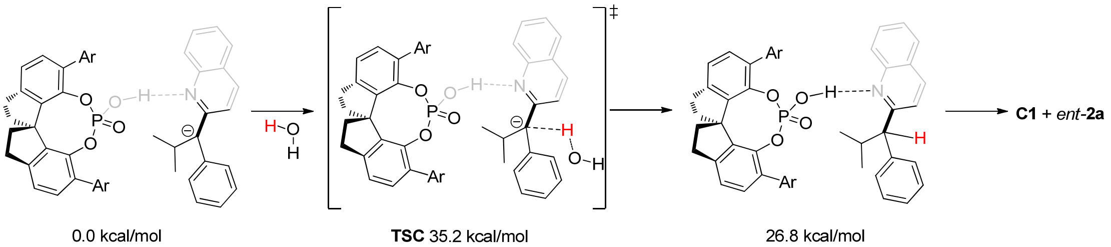

Figure S185. The calculated ΔG values (in kcal/mol) for the formation of (*ent*)-**2a** through **TSC** transition state. The ΔG for the generation of **TSC** transition state are 35.2 kcal/mol and The ΔG of the formation of **C1** and (*ent*)-**2a** are 26.8 kcal/mol, related to **Figures 3 and 4**.

Supplemental Item Legands

Table S1. **Evaluate the effect of BA, amine, base, and the solvent on the reductive dechlorination – deuteration of 1a**, related to **Table 1.**

Table S2. Evaluate the effect of PC and [D] source on the reductive dechlorination – deuteration of 1a, related to **Table 1.**

Table S3. Evaluate the effect of amine and time on the enantioselective reduction of 1a, related to **Table 1.**

Table S4. Optimization of asymmetric reduction – deuteration of 4a, related to **Figure 5**.

Table S5. Cartesian Coordinates for the formation of (*R*)-2a proceeding transferred proton of P=O–H of CPA via TSA process, related to **Figures 3 and 4.**

Table S6. Cartesian Coordinates for the formation of (*ent*)-2a proceeding transferred proton of P–O–H of CPA via TSB process, related to **Figures 3 and 4.**

Table S7. Cartesian Coordinates for the formation of (*ent*)-2a proceeding transferred proton of H_2O via TSC process, related to **Figures 3 and 4.**

Data S1. Crystal Data and structure Refinement for 8, related to **Figure 2.**

Data S2. Crystal Data and structure Refinement for 5n, related to **Figure 5.**

Transparent Methods

Proton nuclear magnetic resonance (^{1}H NMR) and carbon NMR (^{13}C NMR) were recorded in $CDCl_3$ otherwise stated. Chemical shifts are reported in parts per million (ppm), using the residual solvent signal as an internal standard: $CDCl_3$ (^{1}H NMR: δ 7.26, singlet; ^{13}C NMR: δ 77.0, triplet). Multiplicities were given as: *s* (singlet), *d* (doublet), *t* (triplet), *q* (quartet), *quintet*, *m* (multiplets), *dd* (doublet of doublets), *dt* (doublet of triplets), and *br* (broad). Coupling constants (*J*) were recorded in Hertz (Hz). The number of proton atoms (*n*) for a given resonance was indicated by *n*H. The number of carbon atoms (*n*) for a given resonance was indicated by *n*C. HRMS (Analyzer: TOF) was reported in units of mass of charge ratio (m/z). Mass samples were dissolved in CH_3CN (HPLC Grade) unless otherwise stated. Optical rotations were recorded on a polarimeter with a sodium lamp of wavelength 589 nm and reported as follows; $[\alpha]_{\lambda}^{T^{\circ}C}$ (c = g/100 mL, solvent). Melting points were determined on a melting point apparatus. Enantiomeric excesses were determined by chiral High Performance Liquid Chromatography (HPLC) analysis. UV detection was monitored at 254 nm and 210 nm at the same time. HPLC samples were dissolved in HPLC grade isopropanol (IPA) unless otherwise stated. All commercial reagents were purchased with the highest purity grade. They were used without further purification unless specified. All solvents used, mainly petroleum ether (PE) and ethyl acetate (EtOAc) were distilled. Anhydrous dichloromethane (DCM) was freshly distilled from CaH_2 and stored under N_2 atmosphere. Toluene and its derivatives were freshly distilled from sodium/benzophenone before use. All compounds synthesized were stored in a 0 °C freezer and light-sensitive compounds were protected with aluminium foil.

General Procedure for the Preparation of α-chloro-azaarenes

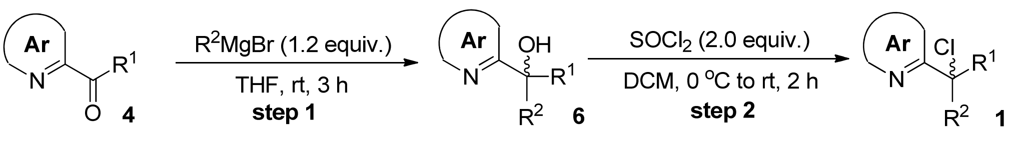

Step 1: To a flame dried flask was equipped in **4** (5.0 mmol), THF (20 mL) under N_2. To this mixture, *i*PrMgBr (6.0 mmol, 1.2 equiv.) was added with vigorous stirring at 0 °C. Subsequently, the mixture was warmed up to room temperature, and stirred for 2~5 h. Then quenched with saturated NH_4Cl solutions and extracted with Et_2O (3 x 10 mL), removed the solvent in *vacuo*, the reaction mixture was dissolved with toluene and loaded onto a short *silica gel* column, followed by gradient elution with petroleum ether/ethyl acetate (100/1 to 20/1 ratio). Removing the solvent in *vacuo*, afforded products **6** (e.g. **6a**, yield = 88%).

Step 2: To a solution of **6** (Terrasson et al., 2008) (2.0 mmol) in dry DCM (5 mL) was added $SOCl_2$ (4.0 mmol, 2.0 equiv.) at 0 °C. Subsequently, the mixture was warmed up to room temperature, and stirred for 2~10 h. TLC monitored until full conversion of **6**. Then cooled to 0 °C again, saturated $NaHCO_3$ solutions were added until no gas produced. Then extracted with DCM (3 x 10 mL), removed the solvent in *vacuo*, the reaction mixture was dissolved with toluene and loaded onto a short basified *silica gel* column, followed by gradient elution with petroleum ether. Removing the solvent in *vacuo*, afforded products **1**. (eg. **1a**, colorless oil, 65% yield; ^{1}H NMR (300 MHz, $CDCl_3$) δ 8.33 (d, J = 8.8 Hz, 1H), 8.08 (d, J = 8.8 Hz, 1H), 7.87 – 7.75 (m, 5H), 7.62 (t, J = 7.5 Hz, 1H), 7.45 – 7.30 (m, 3H), 4.06 – 3.79 (m, 1H), 1.44 (d, J = 6.3 Hz, 3H), 1.10 (d, J = 6.7 Hz, 3H); ^{13}C NMR (75 MHz, $CDCl_3$) δ 162.5, 146.2, 143.0, 136.1, 129.7, 129.3, 127.8, 127.2, 127.2, 127.1, 127.0, 126.6, 122.0, 85.8, 37.3, 19.5, 18.1).

General experimental procedures for the enantioselective reductive dechlorination – deuteration of α-chloro-azaarenes

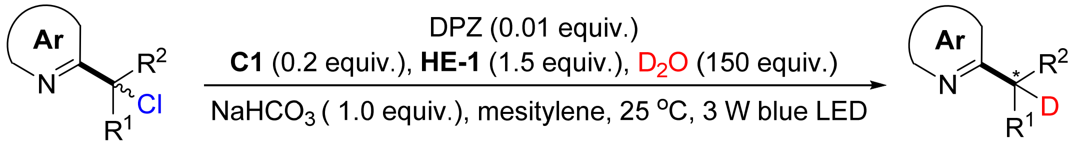

1a-dd, 1gg-hh **2a-dd, 1gg-hh**

To a flame dried Schlenk tube was sequentially added **1a–dd** or **1gg–hh** (0.10 mmol, 1.0 equiv.), DPZ (0.001 mmol, 0.01 equiv.), **C1** (0.02 mmol, 0.2 equiv.), **HE-1** (0.15 mmol, 1.5 equiv.), $NaHCO_3$ (0.10 mmol, 1.0 equiv.), mesitylene (2 mL), and D_2O (15 mmol, 150 equiv.). Then degassed three times by freeze-pump-thaw method. The reaction mixture was stirred under an argon atmosphere at 25 °C (the temperature was maintained in an incubator) for 5 min without light, then irradiated by a 3 W blue LED for another 20−60 min. The reaction was monitored by TLC. After completion of the reaction, the reaction mixture was directly loaded onto a short basified *silica gel* column, followed by gradient elution with petroleum ether/ethyl acetate (100/1 ratio). Removing the solvent in *vacuo*, afforded products **2a–dd or 2gg–hh**.

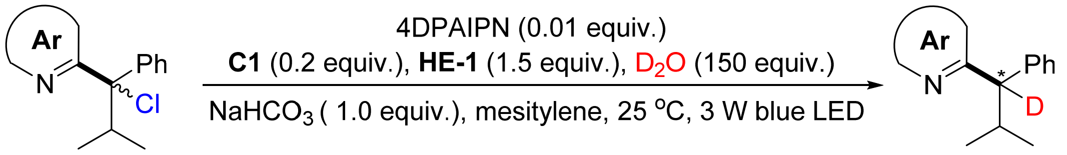

1ee-ff **2ee-ff**

To a flame dried Schlenk tube was sequentially added **1ee–ff** (0.10 mmol, 1.0 equiv.), 4DPAIPN (0.001 mmol, 0.01 equiv.), **C1** (0.02 mmol, 0.2 equiv.), **HE-1** (0.15 mmol, 1.5 equiv.), $NaHCO_3$ (0.10 mmol, 1.0 equiv.), mesitylene (2 mL), and D_2O (15 mmol, 150 equiv.). Then degassed three times by freeze-pump-thaw method. The reaction mixture was stirred under an argon atmosphere at 25 °C (the temperature was maintained in an incubator) for 5 min without light, then irradiated by a 3 W blue LED for another 20–60 min. The reaction was monitored by TLC. After completion of the reaction, the reaction mixture was directly loaded onto a short basified *silica gel* column, followed by gradient elution with petroleum ether/ethyl acetate (100/1 ratio). Removing the solvent in *vacuo*, afforded products **2ee–ff**.

General experimental procedures for the asymmetric reduction – deuteration of azaarene-substituted ketones

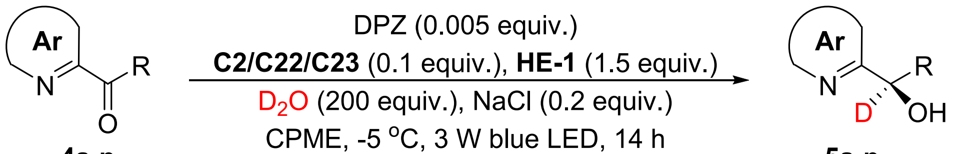

4a-p **5a-p**

To a flame dried Schlenk tube was sequentially added **4a–p** (0.10 mmol, 1.0 equiv.), DPZ (0.0005 mmol, 0.005 equiv.), **C2/C22/C23** (0.01 mmol, 0.1 equiv.), **HE-1** (0.15 mmol, 1.5 equiv.), NaCl (0.02 mmol, 0.2 equiv.), CPME (2 mL), and D_2O (20 mmol, 200 equiv.). Then degassed three times by freeze-pump-thaw method. The reaction mixture was stirred under an argon atmosphere at –5/15 °C (the temperature was maintained in an incubator) for 15 min without light, then irradiated by a 3 W blue LED for another 14–24 hours. The reaction was monitored by TLC. After completion of the reaction, the reaction mixture was directly loaded onto a short basified *silica gel* column, followed by gradient elution with petroleum ether/ethyl acetate (8/1 ratio). Removing the solvent in *vacuo*, afforded products **5a–p**.

Procedures for the Preparation of 8

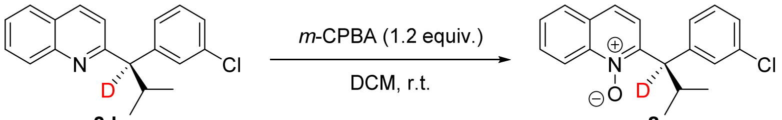

2d **8**

To a stirred solution of **2d** (0.1 mmol, 1.0 equiv.) in DCM 2.0 (mL) was added 3-chloroperoxy-benzoic acid (0.12 mmol, 1.2 equiv.) in portions. The mixture was stirred for 12 h at room temperature, sodium thiosulfate was added (5.0 mL) followed by saturated

sodium hydrogen carbonate (30 mL). The mixture was extracted with DCM (3 × 5.0 mL), the organic layers combined, dried with Na_2SO_4, the reaction mixture was directly loaded onto a short *silica gel* column, followed by gradient elution with petroleum ether/ethyl acetate (100/1–10/1 ratio). Removing the solvent in *vacuo*, afforded the N-O product **8** in 87% yield with 90% ee.

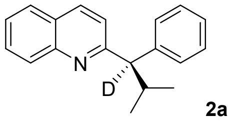

2a

White solid; Mp 104 – 106 °C; 19.7 mg, 75% yield; >95% D, 93% ee; $[\alpha]_D^{22}$ –38.3 (*c* 1.0, $CHCl_3$); ^{1}H NMR (300 MHz, $CDCl_3$) δ 8.17 – 7.90 (m, 2H), 7.78 – 7.57 (m, 2H), 7.54 – 7.39 (m, 3H), 7.34 (d, *J* = 8.5 Hz, 1H), 7.28 – 7.18 (m, 2H), 7.17 – 7.07 (m, 1H), 2.86 – 2.67 (m, 1H), 1.00 – 0.75 (m, 6H); ^{13}C NMR (75 MHz, $CDCl_3$) δ 163.9, 142.8, 136.3, 129.2, 129.1, 128.5, 128.4, 127.4, 126.8, 126.4, 125.8, 121.1, 31.9, 21.7, 21.4; HRMS (ESI) m/z 263.1647 (M+H^+), calc. for $C_{19}H_{19}DN$ 263.1654. The ee was determined by HPLC analysis: CHIRALPAK IG (4.6 mm i.d. x 250 mm); hexane/2-propanol = 97/3; flow rate 1.2 mL/min; 25 °C; 230 nm; retention time: 4.6 min (major) and 9.2 min (minor).

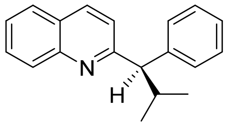

2a'

White solid; Mp 104 – 106 °C; 18.9 mg, 73% yield; ^{1}H NMR (300 MHz, $CDCl_3$) δ 8.02 (d, *J* = 8.5 Hz, 1H), 7.90 (d, *J* = 8.5 Hz, 1H), 7.66 – 7.53 (m, 2H), 7.44 – 7.32 (m, 3H), 7.26 (d, *J* = 8.5 Hz, 1H), 7.18 (t, *J* = 7.4 Hz, 2H), 7.06 (t, *J* = 7.3 Hz, 1H), 3.75 (d, *J* = 10.9 Hz, 1H), 2.78 – 2.66 (m, 1H), 0.83 (t, *J* = 6.0 Hz, 6H); ^{13}C NMR (75 MHz, $CDCl_3$) δ 164.0, 147.8, 142.9, 136.2, 129.2, 129.2, 128.5, 128.3, 127.4, 126.8, 126.3, 125.7, 121.2, 63.2, 32.0, 21.7, 21.4.

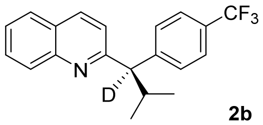

2b

Colorless oil; 19.2 mg, 58% yield; >95% D, 95% ee; $[\alpha]_D^{22}$ –111.4 (*c* 1.0, $CHCl_3$); ^{1}H NMR (300 MHz, $CDCl_3$) δ 8.28 – 7.95 (m, 2H), 7.81 – 7.59 (m, 4H), 7.51 – 7.45 (m, 3H), 7.34 (d, *J* = 8.4 Hz, 1H), 2.89 – 2.85 (m, 1H), 0.93 – 0.85 (m, 6H); ^{13}C NMR (75 MHz, $CDCl_3$) δ 162.8, 147.0, 136.5, 129.4, 129.2, 128.8, 128.4, 127.4, 126.9, 126.0, 125.3, 125.3, 125.2, 125.2, 122.4, 121.2, 32.2, 21.6, 21.3; HRMS (ESI) m/z 331.1519 (M+H^+), calc. for $C_{20}H_{18}DF_3N$ 331.1527. The ee was determined by HPLC analysis: CHIRALPAK ODH x 2 (4.6 mm i.d. x

250 mm); hexane/2-propanol = 98/2; flow rate 1.0 mL/min; 25 ºC; 230 nm; retention time: 9.3 min (minor) and 11.3 min (major).

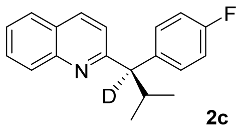

2c

White solid; Mp 92 – 94 ºC; 17.4 mg, 62% yield; 95% D, 91% ee; $[\alpha]_D^{22}$ –52.1 (*c* 1.0, $CHCl_3$); ^1H NMR (300 MHz, $CDCl_3$) δ 8.10 – 7.86 (m, 2H), 7.72 – 7.55 (m, 2H), 7.46 – 7.31 (m, 3H), 7.29 – 7.14 (m, 1H), 6.88 (t, *J* = 8.7 Hz, 2H), 3.73 (d, *J* = 10.0 Hz, 0.05H), 2.80 – 2.56 (m, 1H), 0.87 – 0.73 (m, 6H); ^{13}C NMR (75 MHz, $CDCl_3$) δ 163.6, 163.1, 159.9, 138.5, 136.6, 129.9, 129.8, 129.4, 129.0, 127.4, 126.8, 126.0, 121.1, 115.3, 115.0, 32.2, 21.6, 21.3; HRMS (ESI) m/z 281.1553 ($M+H^+$), calc. for $C_{19}H_{18}DFN$ 281.1559. The ee was determined by HPLC analysis: CHIRALPAK IG (4.6 mm i.d. x 250 mm); hexane/2-propanol = 97/3; flow rate 1.2 mL/min; 25 ºC; 230 nm; retention time: 4.5 min (major) and 6.6 min (minor).

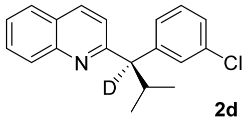

2d

White solid; Mp 91 – 93 ºC; 18.4 mg, 62% yield; >95% D, 93% ee; $[\alpha]_D^{22}$ –65.8 (*c* 1.0, $CHCl_3$); ^1H NMR (300 MHz, $CDCl_3$) δ 8.12 – 8.01 (m, 2H), 7.76 – 7.65 (m, 2H), 7.56 – 7.44 (m, 2H), 7.41 – 7.30 (m, 2H), 7.24 – 7.08 (m, 2H), 2.87 – 2.67 (m, 1H), 0.96 – 0.84 (m, 6H); ^{13}C NMR (75 MHz, $CDCl_3$) δ 163.1, 145.0, 136.4, 134.1, 129.6, 129.4, 129.2, 128.5, 127.4, 126.9, 126.8, 126.6, 126.0, 121.2, 32.1, 21.6, 21.4; HRMS (ESI) m/z 297.1257 ($M+H^+$), calc. for $C_{19}H_{18}DClN$ 297.1263. The ee was determined by HPLC analysis: CHIRALPAK IG (4.6 mm i.d. x 250 mm); hexane/2-propanol = 97/3; flow rate 1.2 mL/min; 25 ºC; 230 nm; retention time: 4.1 min (major) and 5.7 min (minor).

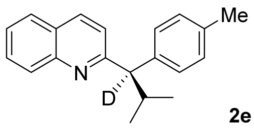

2e

Colorless oil; 21.6 mg, 78% yield; >95% D, 93% ee; $[\alpha]_D^{22}$ +17.3 (*c* 1.0, $CHCl_3$); ^1H NMR (300 MHz, $CDCl_3$) δ 8.12 (d, *J* = 8.3 Hz, 1H), 8.01 (d, *J* = 8.5 Hz, 1H), 7.81 – 7.61 (m, 2H), 7.52 – 7.42 (m, 1H), 7.42 – 7.32 (m, 3H), 7.14 – 7.03 (m, 2H), 3.84 (d, *J* = 10.1 Hz, 0.05H), 2.88 – 2.67 (m, 1H), 2.28 (s, 3H), 1.01 – 0.80 (m, 6H); ^{13}C NMR (75 MHz, $CDCl_3$) δ 164.2, 147.6, 139.8, 136.4, 135.9, 129.2, 129.1, 128.3, 127.4, 126.8, 125.8, 121.0, 31.8, 21.7, 21.4, 21.0; HRMS (ESI) m/z 277.1803 ($M+H^+$), calc. for $C_{20}H_{21}DN$ 277.1810. The ee was determined by HPLC analysis: CHIRALPAK IG (4.6 mm i.d. x 250 mm); hexane/2-propanol

= 97/3; flow rate 1.2 mL/min; 25 °C; 230 nm; retention time: 4.9 min (major) and 8.7 min (minor).

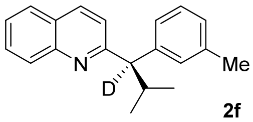

2f

White solid; Mp 89 – 91 °C; 20.2 mg, 73% yield; 95% D, 91% ee; $[\alpha]_D^{22}$ −13.3 (*c* 1.0, $CHCl_3$); ^1H NMR (300 MHz, $CDCl_3$) δ 8.19 – 7.90 (m, 2H), 7.75 – 7.55 (m, 2H), 7.48 – 7.37 (m, 1H), 7.33 (d, *J* = 8.5 Hz, 1H), 7.23 (t, *J* = 8.5 Hz, 2H), 7.11 (t, *J* = 7.5 Hz, 1H), 6.96 – 6.85 (m, 1H), 3.78 (d, *J* = 9.0 Hz, 0.05H), 2.25 (s, 3H), 0.96 – 0.72 (m, 6H); ^{13}C NMR (75 MHz, $CDCl_3$) δ 164.0, 147.5, 142.7, 137.9, 136.3, 129.3, 129.2, 129.1, 128.2, 127.4, 127.1, 126.8, 125.8, 125.3, 121.0, 31.7, 21.7, 21.5, 21.5; HRMS (ESI) m/z 277.1804 ($M+H^+$), calc. for $C_{20}H_{21}DN$ 277.1810. The ee was determined by HPLC analysis: CHIRALPAK IG (4.6 mm i.d. x 250 mm); hexane/2-propanol = 97/3; flow rate 1.2 mL/min; 25 °C; 230 nm; retention time: 4.0 min (major) and 6.6 min (minor).

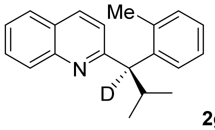

2g

White solid; Mp 89 – 91 °C; 18.8 mg, 68% yield; 94% D, 88% ee; $[\alpha]_D^{22}$ +38.7 (*c* 1.0, $CHCl_3$); ^1H NMR (300 MHz, $CDCl_3$) δ 8.09 (d, *J* = 7.9 Hz, 1H), 7.98 (d, *J* = 8.4 Hz, 1H), 7.77 – 7.58 (m, 3H), 7.46 (t, *J* = 7.4 Hz, 1H), 7.32 (d, *J* = 8.5 Hz, 1H), 7.19 (t, *J* = 7.3 Hz, 1H), 7.12 – 7.02 (m, 2H), 4.18 – 4.15 (m, 0.06H), 2.88 – 2.75 (m, 1H), 2.49 (s, 3H), 0.96 – 0.89 (m, 6H); ^{13}C NMR (75 MHz, $CDCl_3$) δ 163.8, 147.7, 141.1, 137.0, 136.1, 130.3, 129.2, 129.1, 127.3, 127.1, 126.7, 126.1, 126.0, 125.7, 121.1, 32.1, 21.7, 21.1, 20.5; HRMS (ESI) m/z 277.1803 ($M+H^+$), calc. for $C_{20}H_{21}DN$ 277.1810. The ee was determined by HPLC analysis: CHIRALPAK IG (4.6 mm i.d. x 250 mm); hexane/2-propanol = 97/3; flow rate 1.2 mL/min; 25 °C; 230 nm; retention time: 3.8 min (major) and 10.0 min (minor).

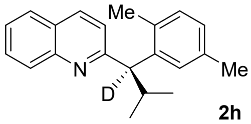

2h

Colorless oil; 19.7 mg, 68% yield; 93% D, 85% ee; $[\alpha]_D^{22}$ +138.9 (*c* 1.0, $CHCl_3$); ^1H NMR (300 MHz, $CDCl_3$) δ 8.09 (d, *J* = 8.4 Hz, 1H), 7.98 (d, *J* = 8.5 Hz, 1H), 7.74 – 7.62 (m, 2H), 7.49 – 7.40 (m, 2H), 7.31 (d, *J* = 8.5 Hz, 1H), 6.99 (d, *J* = 7.6 Hz, 1H), 6.87 (d, *J* = 7.5 Hz, 1H), 4.14 (d, *J* = 11.1 Hz, 0.07H), 2.86 – 2.76 (m, 1H), 2.45 (s, 3H), 2.31 (s, 3H), 0.96 – 0.89 (m, 6H); ^{13}C NMR (75 MHz, $CDCl_3$) δ 163.9, 147.6, 140.8, 136.1, 135.3, 133.9, 130.2, 129.2,

129.0, 127.7, 127.3, 126.7, 125.7, 121.1, 32.0, 21.7, 21.3, 21.2, 20.1; HRMS (ESI) m/z 291.1958 (M+H$^+$), calc. for $C_{21}H_{23}DN$ 291.1966. The ee was determined by HPLC analysis: CHIRALPAK IG (4.6 mm i.d. x 250 mm); hexane/2-propanol = 98/2; flow rate 1.0 mL/min; 25 °C; 230 nm; retention time: 3.9 min (major) and 11.7 min (minor).

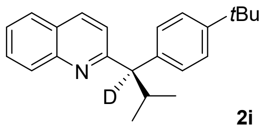

2i

White solid; Mp 94 – 97 °C; 21.7 mg, 68% yield; 95% D, 90% ee; $[\alpha]_D^{22}$ +25.3 (*c* 1.0, $CHCl_3$); ^{1}H NMR (300 MHz, $CDCl_3$) δ 8.11 (d, *J* = 8.2 Hz, 1H), 8.01 (d, *J* = 8.6 Hz, 1H), 7.75 – 7.61 (m, 2H), 7.50 – 7.34 (m, 4H), 7.29 – 7.24 (m, 2H), 2.81 – 2.75 (m, 1H), 1.26 (s, 9H), 0.93 (d, *J* = 6.5 Hz, 3H), 0.89 (d, *J* = 6.5 Hz, 3H); ^{13}C NMR (75 MHz, $CDCl_3$) δ 164.3, 149.0, 147.7, 139.7, 136.2, 129.1, 128.0, 127.4, 126.8, 125.7, 125.2, 121.1, 34.3, 31.9, 31.3, 21.7, 21.5; HRMS (ESI) m/z 319.2272 (M+H$^+$), calc. for $C_{23}H_{27}DN$ 319.2279. The ee was determined by HPLC analysis: CHIRALPAK ODH x 2 (4.6 mm i.d. x 250 mm); hexane/2-propanol = 98/2; flow rate 1.2 mL/min; 25 °C; 230 nm; retention time: 8.4 min (minor) and 9.9 min (major).

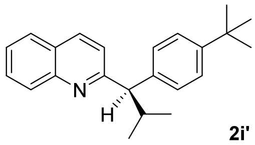

2i'

White solid; Mp 94 – 97 °C; 22.4 mg, 70% yield; ^{1}H NMR (300 MHz, $CDCl_3$) δ 8.03 (d, *J* = 8.5 Hz, 1H), 7.93 (d, *J* = 8.5 Hz, 1H), 7.68 – 7.54 (m, 2H), 7.41 – 7.27 (m, 4H), 7.21 – 7.16 (m, 2H), 3.75 (d, *J* = 10.8 Hz, 1H), 2.78 – 2.60 (m, 1H), 1.18 (s, 9H), 0.86 (d, *J* = 6.6 Hz, 3H), 0.81 (d, *J* = 6.5 Hz, 3H); ^{13}C NMR (75 MHz, $CDCl_3$) δ 164.3, 149.0, 139.7, 136.3, 129.2, 129.1, 128.0, 127.4, 126.8, 125.8, 125.2, 121.1, 62.7, 34.3, 32.0, 31.3, 21.7, 21.5.

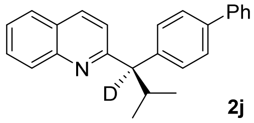

2j

Colorless oil; 26.4 mg, 78% yield; >95% D, 91% ee; $[\alpha]_D^{22}$ –7.2 (*c* 1.0, $CHCl_3$); ^{1}H NMR (300 MHz, $CDCl_3$) δ 8.15 – 7.98 (m, 2H), 7.77 – 7.65 (m, 2H), 7.62 – 7.27 (m, 11H), 2.94 – 2.71 (m, 1H), 0.99 – 0.90 (m, 6H); ^{13}C NMR (75 MHz, $CDCl_3$) δ 163.9, 147.8, 142.0, 140.9, 139.2, 136.3, 129.2, 128.9, 128.6, 127.4, 127.1, 127.0, 126.9, 126.8, 125.8, 121.2, 32.0, 21.7, 21.5; HRMS (ESI) m/z 339.1958 (M+H$^+$), calc. for $C_{25}H_{23}DN$ 339.1966. The ee was determined by HPLC analysis: CHIRALPAK IG (4.6 mm i.d. x 250 mm); hexane/2-propanol = 97/3; flow rate 1.2 mL/min; 25 °C; 230 nm; retention time: 6.6 min (major) and 13.5 min (minor).

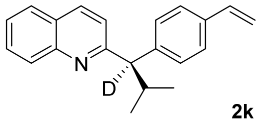

2k

Colorless oil; 23.4 mg, 81% yield; >95% D, 93% ee; $[\alpha]_D^{22}$ –23.7 (*c* 1.0, $CHCl_3$); ^1H NMR (300 MHz, $CDCl_3$) δ 8.09 (d, *J* = 8.4 Hz, 1H), 8.00 (d, *J* = 8.5 Hz, 1H), 7.79 – 7.59 (m, 2H), 7.53 – 7.40 (m, 3H), 7.36 – 7.30 (m, 3H), 6.65 (dd, *J* = 17.6, 10.9 Hz, 1H), 5.67 (d, *J* = 17.5 Hz, 1H), 5.16 (d, *J* = 11.0 Hz, 1H), 2.90 – 2.68 (m, 1H), 0.98 – 0.86 (m, 6H); ^{13}C NMR (75 MHz, $CDCl_3$) δ 163.8, 147.8, 142.6, 136.5, 136.3, 135.7, 129.2, 128.6, 127.4, 126.8, 126.2, 125.8, 121.1, 113.2, 31.9, 21.7, 21.4; HRMS (ESI) m/z 289.1802 ($M+H^+$), calc. for $C_{21}H_{21}DN$ 289.1810. The ee was determined by HPLC analysis: CHIRALPAK IG (4.6 mm i.d. x 250 mm); hexane/2-propanol = 97/3; flow rate 1.2 mL/min; 25 °C; 230 nm; retention time: 5.4 min (major) and 9.7 min (minor).

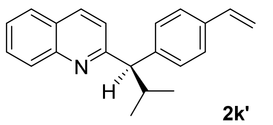

2k'

Colorless oil; 23.4 mg, 81% yield; ^1H NMR (300 MHz, $CDCl_3$) δ 8.02 (d, *J* = 8.5 Hz, 1H), 7.92 (d, *J* = 8.5 Hz, 1H), 7.67 – 7.52 (m, 2H), 7.42 – 7.35 (m, 3H), 7.30 – 7.15 (m, 3H), 6.65 – 6.50 (m, 1H), 5.64 – 5.53 (m, 1H), 5.12 – 5.06 (m, 1H), 3.75 (d, *J* = 10.9 Hz, 1H), 2.87 – 2.57 (m, 1H), 0.89 – 0.79 (m, 6H); ^{13}C NMR (75 MHz, $CDCl_3$) δ 163.8, 147.6, 142.6, 136.5, 136.4, 135.7, 129.3, 129.1, 128.6, 127.4, 126.8, 126.2, 125.8, 121.1, 113.2, 62.8, 31.9, 21.7, 21.4.

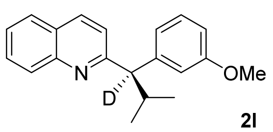

2l

Colorless oil; 20.8 mg, 71% yield; >95% D, 93% ee; $[\alpha]_D^{22}$ –24.4 (*c* 1.0, $CHCl_3$); ^1H NMR (300 MHz, $CDCl_3$) δ 8.11 (d, *J* = 8.4 Hz, 1H), 8.01 (d, *J* = 8.5 Hz, 1H), 7.77 – 7.62 (m, 2H), 7.46 (t, *J* = 7.5 Hz, 1H), 7.37 (d, *J* = 8.5 Hz, 1H), 7.19 (t, *J* = 7.8 Hz, 1H), 7.08 (d, *J* = 7.6 Hz, 2H), 6.73 – 6.67 (m, 1H) 3.78 (s, 3H), 2.90 – 2.70 (m, 1H), 0.96 – 0.86 (m, 6H); ^{13}C NMR (75 MHz, $CDCl_3$) δ 163.8, 159.5, 147.8, 144.5, 136.2, 129.2, 129.2, 127.4, 126.8, 125.7, 121.1, 120.9, 114.4, 111.4, 55.1, 31.9, 21.7, 21.4; HRMS (ESI) m/z 293.1752 ($M+H^+$), calc. for $C_{20}H_{21}DNO$ 293.1759. The ee was determined by HPLC analysis: CHIRALPAK IG (4.6 mm i.d. x 250 mm); hexane/2-propanol = 97/3; flow rate 1.2 mL/min; 25 °C; 230 nm; retention time: 5.9 min (major) and 12.1 min (minor).

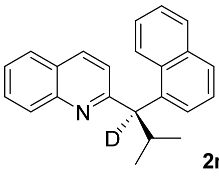

2m

Colorless oil; 20.3 mg, 65% yield; >95% D, 80% ee; $[\alpha]_D^{22}$ −165.9 (*c* 1.0, $CHCl_3$); ^1H NMR (300 MHz, $CDCl_3$) δ 8.61 (d, *J* = 8.5 Hz, 1H), 8.15 (d, *J* = 8.2 Hz, 1H), 7.92 (d, *J* = 8.5 Hz, 1H), 7.82 (t, *J* = 7.4 Hz, 2H), 7.76 – 7.60 (m, 3H), 7.57 – 7.30 (m, 5H), 3.04 – 2.96 (m, 1H), 1.02 – 0.97 (m, 6H); ^{13}C NMR (75 MHz, $CDCl_3$) δ 163.8, 138.7, 136.5, 134.0, 132.7, 129.2, 128.7, 127.4, 127.0, 126.7, 125.9, 125.8, 125., 125.3, 124.7, 124.3, 120.9, 32.0, 21.8, 21.5; HRMS (ESI) m/z 313.1802 (M+H^+), calc. for $C_{23}H_{21}DN$ 313.1810. The ee was determined by HPLC analysis: CHIRALPAK ODH x 2 (4.6 mm i.d. x 250 mm); hexane/2-propanol = 98/2; flow rate 1.2 mL/min; 25 °C; 230 nm; retention time: 12.6 min (minor) and 13.9 min (major).

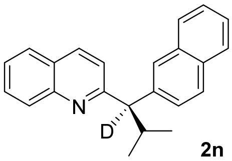

2n

Colorless oil; 22.5 mg, 72% yield; >95% D, 88% ee; $[\alpha]_D^{22}$ −182.9 (*c* 1.0, $CHCl_3$); ^1H NMR (300 MHz, $CDCl_3$) δ 8.13 (d, *J* = 8.3 Hz, 1H), 8.00 (d, *J* = 8.6 Hz, 1H), 7.91 (s, 1H), 7.84 – 7.63 (m, 6H), 7.50 – 7.33 (m, 4H), 3.00 – 2.90 (m, 1H), 0.96 (d, *J* = 6.4 Hz, 6H); ^{13}C NMR (75 MHz, $CDCl_3$) δ 163.7, 147.6, 140.4, 136.3, 133.5, 132.3, 129.3, 129.1, 128.0, 127.7, 127.5, 127.4, 127.0, 126.8, 126.8, 125.8, 125.4, 121.3, 31.7, 21.7, 21.5; HRMS (ESI) m/z 313.1802 (M+H^+), calc. for $C_{23}H_{21}DN$ 313.1810. The ee was determined by HPLC analysis: CHIRALPAK IG (4.6 mm i.d. x 250 mm); hexane/2-propanol = 97/3; flow rate 1.2 mL/min; 25 °C; 230 nm; retention time: 5.9 min (major) and 11.0 min (minor).

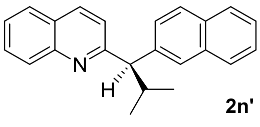

2n'

Colorless oil; 22.9 mg, 72% yield; ^1H NMR (300 MHz, $CDCl_3$) δ 8.07 (d, *J* = 8.4 Hz, 1H), 7.91 (d, *J* = 8.5 Hz, 1H), 7.82 (s, 1H), 7.75 – 7.50 (m, 6H), 7.44 – 7.26 (m, 4H), 3.94 (d, *J* = 10.8 Hz, 1H), 2.92 – 2.71 (m, 1H), 0.91 – 0.82 (m, 6H); ^{13}C NMR (75 MHz, $CDCl_3$) δ 163.8, 147.6, 140.4, 136.4, 133.5, 132.3, 129.3, 129.1, 128.0, 127.7, 127.5, 127.4, 127.0, 126.8, 125.9, 125.8, 125.4, 121.3, 63.1, 31.8, 21.7, 21.5.

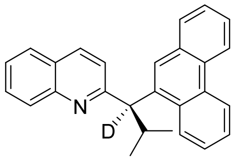

2o

White solid; Mp 160 – 162 °C; 21.7 mg, 66% yield; >95% D, 80% ee; $[\alpha]^{22}_{D}$ +492.8 (*c* 1.0, $CHCl_3$); ^{1}H NMR (300 MHz, $CDCl_3$) δ 8.78 – 8.59 (m,3H), 8.18 (d, *J* = 8.6 Hz, 1H), 8.06 (s, 1H), 8.01 – 7.84 (m, 2H), 7.73 – 7.55 (m, 6H), 7.48 – 7.35 (m, 2H), 3.10 – 3.05 (m, 1H), 1.09 (d, *J* = 6.4 Hz, 3H), 1.02 (d, *J* = 6.5 Hz, 3H); ^{13}C NMR (75 MHz, $CDCl_3$) δ 163.7, 147.57, 136.9, 136.1, 131.9, 131.7, 130.7, 129.6, 129.2, 128.6, 127.4, 126.7, 126.6, 126.5, 126.3, 126.1, 125.8, 125.4, 125.2, 123.0, 122.4, 120.6, 31.7, 21.8, 21.7; HRMS (ESI) m/z 363.1956 ($M+H^+$), calc. for $C_{27}H_{23}DN$ 363.1966. The ee was determined by HPLC analysis: CHIRALPAK IG (4.6 mm i.d. x 250 mm); hexane/2-propanol = 97/3; flow rate 1.2 mL/min; 25 °C; 230 nm; retention time: 6.5 min (major) and 8.2 min (minor).

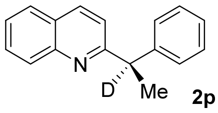

2p

Colorless oil; 19.4 mg, 83% yield; >95% D, 88% ee; $[\alpha]^{22}_{D}$ +15.0 (*c* 1.0, $CHCl_3$); ^{1}H NMR (300 MHz, $CDCl_3$) δ 8.03 (d, *J* = 8.4 Hz, 1H), 7.90 (d, *J* = 8.5 Hz, 1H), 7.68 – 7.52 (m, 2H), 7.40 (t, *J* = 7.5 Hz, 1H), 7.31 – 7.05 (m, 6H), 4.42 (q, *J* = 7.1 Hz, 0.1H), 1.71 (s, 3H); ^{13}C NMR (75 MHz, $CDCl_3$) δ 165.1, 147.5, 144.5, 136.3, 129.3, 129.1, 128.5, 127.8, 127.4, 126.8, 126.4, 125.9, 120.6, 48.0, 47.8, 47.6, 47.3, 20.3; HRMS (ESI) m/z 235.1334 ($M+H^+$), calc. for $C_{17}H_{15}DN$ 235.1340. The ee was determined by HPLC analysis: CHIRALPAK IG (4.6 mm i.d. x 250 mm); hexane/2-propanol = 97/3; flow rate 1.2 mL/min; 25 °C; 230 nm; retention time: 6.0 min (major) and 10.3 min (minor).

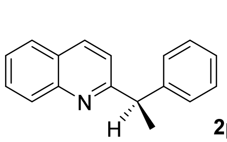

2p'

Colorless oil; 20.2 mg, 84% yield; ^{1}H NMR (300 MHz, $CDCl_3$) δ 8.04 (d, *J* = 8.4 Hz, 1H), 7.91 (d, *J* = 8.5 Hz, 1H), 7.68 – 7.55 (m, 2H), 7.40 (t, *J* = 7.1 Hz, 1H), 7.32 – 7.06 (m, 6H), 4.50 – 4.26 (m, 1H), 1.72 (d, *J* = 7.2 Hz, 3H); ^{13}C NMR (75 MHz, $CDCl_3$) δ 165.1, 147.6, 144.6, 136.3, 129.3, 129.2, 128.5, 127.8, 127.4, 126.8, 126.4, 125.9, 120.7, 48.0, 20.4.

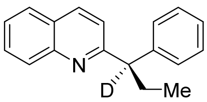

2q

Colorless oil; 21.1 mg, 85% yield; >95% D, 87% ee; $[\alpha]_D^{22}$ +31.7 (*c* 1.0, $CHCl_3$); ^1H NMR (300 MHz, $CDCl_3$) δ 8.11 (d, *J* = 8.4 Hz, 1H), 8.00 (d, *J* = 8.6 Hz, 1H), 7.79 – 7.63 (m, 2H), 7.48 (t, *J* = 7.5 Hz, 1H), 7.40 (d, *J* = 7.3 Hz, 2H), 7.34 – 7.13 (m, 4H), 4.20 – 4.18 (m, 0.05H), 2.43 – 2.32 (m, 1H), 2.27 – 2.15 (m, 1H), 0.95 (t, *J* = 7.3 Hz, 3H); ^{13}C NMR (75 MHz, $CDCl_3$) δ 164.1, 147.6, 143.2, 136.4, 129.3, 129.1, 128.4, 128.2, 127.4, 126.8, 126.4, 125.9, 120.9, 27.7, 12.6; HRMS (ESI) m/z 249.1491 ($M+H^+$), calc. for $C_{18}H_{17}DN$ 249.1497. The ee was determined by HPLC analysis: CHIRALPAK IG (4.6 mm i.d. x 250 mm); hexane/2-propanol = 97/3; flow rate 1.2 mL/min; 25 °C; 230 nm; retention time: 5.5 min (major) and 9.9 min (minor).

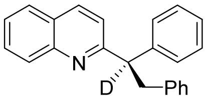

2r

White solid; Mp 54 – 56 °C; 27.6 mg, 89% yield; >95% D, 84% ee; $[\alpha]_D^{22}$ –83.8 (*c* 1.0, $CHCl_3$); ^1H NMR (300 MHz, $CDCl_3$) δ 8.16 (d, *J* = 8.1 Hz, 1H), 7.98 (d, *J* = 8.5 Hz, 1H), 7.76 – 7.65 (m, 2H), 7.49 (t, *J* = 7.5 Hz, 1H), 7.38 – 7.33 (m, 2H), 7.29 – 7.20 (m, 3H), 7.20 – 7.06 (m, 6H), 3.86 (d, *J* = 13.7 Hz, 1H), 3.44 (d, *J* = 13.8 Hz, 1H); ^{13}C NMR (75 MHz, $CDCl_3$) δ 162.9, 142.9, 140.4, 136.4, 129.4, 129.2, 128.4, 128.3, 128.0, 127.4, 126.8, 126.5, 126.0, 125.8, 121.6, 40.9; HRMS (ESI) m/z 311.1645 ($M+H^+$), calc. for $C_{23}H_{19}DN$ 311.1653. The ee was determined by HPLC analysis: CHIRALPAK IG (4.6 mm i.d. x 250 mm); hexane/2-propanol = 97/3; flow rate 1.2 mL/min; 25 °C; 230 nm; retention time: 7.0 min (major) and 10.0 min (minor).

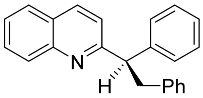

2r'

White solid; Mp 54 – 56 °C; 26.1 mg, 85% yield; ^1H NMR (300 MHz, $CDCl_3$) δ 8.07 (d, *J* = 8.3 Hz, 1H), 7.89 (d, *J* = 8.4 Hz, 1H), 7.68 – 7.51 (m, 2H), 7.40 (t, *J* = 7.5 Hz, 1H), 7.26 (d, *J* = 7.2 Hz, 2H), 7.19 – 7.10 (m, 3H), 7.11 – 6.98 (m, 6H), 4.55 – 4.46 (m, 1H), 3.81 – 3.65 (m, 1H), 3.41 – 3.30 (m, 1H); ^{13}C NMR (75 MHz, $CDCl_3$) δ 163.0, 147.7, 143.12, 143.1, 140.5, 136.1, 129.2, 128.4, 128.3, 128.0, 127.4, 126.9, 126.5, 125.9, 125.8, 121.7, 56.0, 41.0.

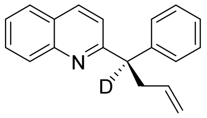

2s

Colorless oil; 18.5 mg, 71% yield; >95% D, 82% ee; $[\alpha]_D^{22}$ -45.3 (*c* 1.0, $CHCl_3$); ^1H NMR (300 MHz, $CDCl_3$) δ 8.13 (d, *J* = 8.1 Hz, 1H), 8.01 (d, *J* = 8.4 Hz, 1H), 7.74 – 7.65 (m, 2H), 7.49 (t, *J* = 7.5 Hz, 1H), 7.39 (d, *J* = 7.3 Hz, 2H), 7.35 – 7.07 (m, 4H), 5.88 – 5.71 (m, 1H), 5.10 – 5.01 (m, 1H), 4.97 – 4.90 (m, 1H), 3.17 (dd, *J* = 14.1, 6.7 Hz, 1H), 2.94 (dd, *J* = 14.2, 6.6 Hz, 1H); ^{13}C NMR (75 MHz, $CDCl_3$) δ 163.3, 147.7, 142.8, 136.7, 136.3, 129.3, 128.5, 128.2, 127.4, 126.9, 126.5, 125.9, 121.2, 116.4, 38.8; HRMS (ESI) m/z 261.1491 ($M+H^+$), calc. for $C_{19}H_{17}DN$ 261.1497. The ee was determined by HPLC analysis: CHIRALPAK IG (4.6 mm i.d. x 250 mm); hexane/2-propanol = 97/3; flow rate 1.0 mL/min; 25 °C; 230 nm; retention time: 5.8 min (major) and 9.4 min (minor).

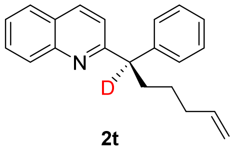

2t

Colorless oil; 19.4 mg, 71% yield; >95% D, 80% ee; $[\alpha]_D^{22}$ –73.2 (*c* 1.0, $CHCl_3$); ^1H NMR (300 MHz, $CDCl_3$) δ 8.11 (d, *J* = 8.5 Hz, 1H), 7.99 (d, *J* = 8.5 Hz, 1H), 7.74 – 7.64 (m, 2H), 7.47 (t, *J* = 7.5 Hz, 1H), 7.39 (d, *J* = 7.1 Hz, 2H), 7.33 – 7.12 (m, 4H), 5.84 – 5.68 (m, 1H), 5.01 – 4.87 (m, 2H), 2.42 – 2.01 (m, 4H), 1.51 – 1.28 (m, 2H); ^{13}C NMR (75 MHz, $CDCl_3$) δ 164.1, 147.8, 143.4, 138.7, 136.2, 129.3, 129.2, 128.5, 128.1, 127.4, 126.8, 126.4, 125.8, 120.9, 114.5, 34.1, 33.8, 27.2.HRMS (ESI) m/z 289.1804 ($M+H^+$), calc. for $C_{21}H_{21}DN$ 289.1810. The ee was determined by HPLC analysis: CHIRALPAK IG (4.6 mm i.d. x 250 mm); hexane/2-propanol = 97/3; flow rate 1.0 mL/min; 25 °C; 230 nm; retention time: 6.1 min (major) and 9.0 min (minor).

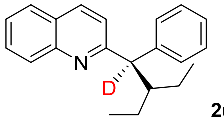

2u

Colorless oil; 21.8 mg, 75% yield; 94% D, 90% ee; $[\alpha]_D^{22}$ –58.9 (*c* 1.0, $CHCl_3$); ^1H NMR (300 MHz, $CDCl_3$) δ 8.10 (d, *J* = 7.4 Hz, 1H), 8.01 (d, *J* = 8.4 Hz, 1H), 7.75 – 7.62 (m, 2H), 7.56 – 7.34 (m, 4H), 7.29 – 7.23 (m, 2H), 7.19 – 7.11 (m, 1H), 4.15 – 4.03 (m, 0.06H), 2.59 (brs, 1H), 1.49 – 1.13 (m, 4H), 0.86 – 0.76 (m, 6H); ^{13}C NMR (75 MHz, $CDCl_3$) δ 163.9, 147.9, 142.7, 136.1, 129.3, 129.1, 128.6, 128.3, 127.3, 126.8, 126.3, 125.7, 121.2, 42.7, 22.1, 21.6, 10.1, 9.7; HRMS (ESI) m/z 291.1957 ($M+H^+$), calc. for $C_{21}H_{23}DN$ 291.1966. The ee was

determined by HPLC analysis: CHIRALPAK IG (4.6 mm i.d. x 250 mm); hexane/2-propanol = 97/3; flow rate 1.2 mL/min; 25 °C; 230 nm; retention time: 4.2 min (major) and 10.3 min (minor).

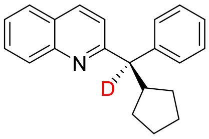

2v

White solid; Mp 103 – 105 °C; 17.9 mg, 62% yield; 92% D, 87% ee; $[\alpha]_D^{22}$ –7.2 (*c* 1.0, $CHCl_3$); ^{1}H NMR (300 MHz, $CDCl_3$) δ 8.09 (d, *J* = 8.5 Hz, 1H), 7.99 (d, *J* = 8.5 Hz, 1H), 7.68 (dd, *J* = 18.9, 7.9 Hz, 2H), 7.49 – 7.40 (m, 3H), 7.34 (d, *J* = 8.5 Hz, 1H), 7.29 – 7.22 (m, 2H), 7.15 (t, *J* = 7.3 Hz, 1H), 3.99 (d, *J* = 11.4 Hz, 0.08H), 3.03 – 2.93 (m, 1H), 1.78 – 1.47 (m, 6H), 1.34 – 1.09 (m, 2H); ^{13}C NMR (75 MHz, $CDCl_3$) δ 164.2, 147.8, 143.4, 136.1, 129.3, 129.1, 128.3, 127.4, 126.8, 126.3, 125.7, 121.0, 44.0, 31.9, 31.7, 25.4, 25.3; HRMS (ESI) m/z 289.1805 (M+H$^+$), calc. for $C_{21}H_{21}DN$ 289.1810. The ee was determined by HPLC analysis: CHIRALPAK IG (4.6 mm i.d. x 250 mm); hexane/2-propanol = 97/3; flow rate 1.2 mL/min; 25 °C; 230 nm; retention time: 5.4 min (major) and 13.1 min (minor).

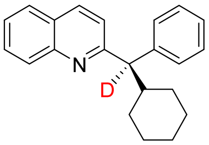

2w

White solid; Mp 98 – 100 °C; 18.7 mg, 62% yield; 95% D, 82% ee; $[\alpha]_D^{22}$ –12.8 (*c* 1.0, $CHCl_3$); ^{1}H NMR (300 MHz, $CDCl_3$) δ 8.09 (d, *J* = 8.2 Hz, 1H), 8.00 (d, *J* = 8.6 Hz, 1H), 7.77 – 7.61 (m, 2H), 7.51 – 7.41 (m, 3H), 7.36 (d, *J* = 8.5 Hz, 1H), 7.32 – 7.21 (m, 2H), 7.15 (t, *J* = 7.3 Hz, 1H), 3.93 – 3.87 (m, 0.05H), 2.49 – 2.40 m, 1H), 1.78 – 1.44 (m, 5H), 1.32 – 1.11 (m, 3H), 1.01 – 0.87 (m, 2H); ^{13}C NMR (75 MHz, $CDCl_3$) δ 163.7, 147.8, 142.4, 136.2, 136.2, 129.2, 128.6, 128.3, 127.4, 126.8, 126.3, 125.7, 121.2, 41.2, 32.0, 31.6, 26.5, 26.3, 26.2; HRMS (ESI) m/z 303.1958 (M+H$^+$), calc. for $C_{22}H_{23}DN$ 303.1966. The ee was determined by HPLC analysis: CHIRALPAK IG (4.6 mm i.d. x 250 mm); hexane/2-propanol = 97/3; flow rate 1.2 mL/min; 25 °C; 230 nm; retention time: 5.0 min (major) and 11.6 min (minor).

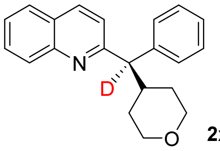

2x

Colorless oil; 21.9 mg, 72% yield; >95% D, 85% ee; $[\alpha]_D^{22}$ –84.2 (*c* 1.0, $CHCl_3$); ^{1}H NMR (300 MHz, $CDCl_3$) δ 8.04 (d, *J* = 7.9 Hz, 1H), 7.94 (d, *J* = 8.4 Hz, 1H), 7.67 – 7.60 (m, 2H), 7.42 – 7.38 (m, 3H), 7.29 – 7.06 (m, 4H), 4.35 – 4.29 (m, 0.03H), 3.84 (t, *J* = 11.9 Hz, 2H),

3.35 (t, J = 11.5 Hz, 2H), 2.72 – 2.65 (m, 1H), 1.48 – 1.18 (m, 4H); ^{13}C NMR (75 MHz, $CDCl_3$) δ 162.4, 147.8, 141.4, 141.4, 136.3, 129.3, 128.6, 128.5, 127.4, 126.8, 126.6, 125.9, 121.7, 68.1, 67.9, 38.7, 32.0, 31.6; HRMS (ESI) m/z 305.1752 (M+H$^+$), calc. for $C_{21}H_{21}DNO$ 305.1759. The ee was determined by HPLC analysis: CHIRALPAK IG (4.6 mm i.d. x 250 mm); hexane/2-propanol = 80/20; flow rate 1.0 mL/min; 25 °C; 230 nm; retention time: 14.3 min (major) and 15.8 min (minor).

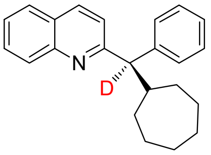

2y

Colorless oil; 20.3 mg, 64% yield; 94% D, 81% ee; $[\alpha]_D^{22}$ –8.7 (c 1.0, $CHCl_3$); ^{1}H NMR (300 MHz, $CDCl_3$) δ 8.08 (d, J = 8.4 Hz, 1H), 7.99 (d, J = 8.5 Hz, 1H), 7.75 – 7.63 (m, 2H), 7.49 – 7.42 (m, 3H), 7.36 (d, J = 8.5 Hz, 1H), 7.30 – 7.22 (m, 2H), 7.15 (t, J = 7.1 Hz, 1H), 4.00 (d, J = 11.5 Hz, 0.06H), 2.75 – 2.72 (m, 1H), 1.71 – 1.35 (m, 10H), 1.33 – 1.00 (m, 2H); ^{13}C NMR (75 MHz, $CDCl_3$) δ 164.1, 147.7, 143.0, 136.2, 129.2, 128.6, 128.4, 127.3, 126.7, 126.3, 125.7, 121.1, 42.1, 33.0, 32.3, 28.5, 28.5, 26.5, 26.5; HRMS (ESI) m/z 317.2114 (M+H$^+$), calc. for $C_{23}H_{25}DN$ 317.2123. The ee was determined by HPLC analysis: CHIRALPAK IG (4.6 mm i.d. x 250 mm); hexane/2-propanol = 97/3; flow rate 1.2 mL/min; 25 °C; 230 nm; retention time: 5.6 min (major) and 14.8 min (minor).

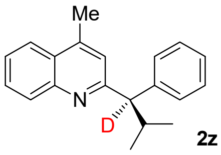

2z

White solid; Mp 66 – 68 °C; 19.6 mg, 71% yield; >95% D, 92% ee; $[\alpha]_D^{22}$ –37.0 (c 1.0, $CHCl_3$); ^{1}H NMR (300 MHz, $CDCl_3$) δ 8.11 (d, J = 7.7 Hz, 1H), 7.90 (d, J = 8.4 Hz, 1H), 7.67 (t, J = 7.6 Hz, 1H), 7.50 – 7.44 (m, 3H), 7.31 – 7.10 (m, 4H), 3.76 (brs, 0.02 H), 2.86 – 2.75 (m, 1H), 2.62 (s, 3H), 0.93 – 0.88 (m, 6H); ^{13}C NMR (75 MHz, $CDCl_3$) δ 163.5, 147.6, 144.2, 143.0, 129.7, 128.9, 128.5, 128.3, 126.9, 126.3, 125.5, 123.5, 121.8, 31.8, 21.7, 21.4, 18.8; HRMS (ESI) m/z 277.1803 (M+H$^+$), calc. for $C_{20}H_{21}DN$ 277.1810. The ee was determined by HPLC analysis: CHIRALPAK IG x 2 (4.6 mm i.d. x 250 mm); hexane/2-propanol = 98/2; flow rate 1.0 mL/min; 25 °C; 230 nm; retention time: 10.4 min (major) and 11.6 min (minor).

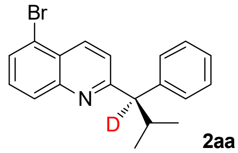

2aa

Colorless oil; 22.2 mg, 65% yield; 95% D, 90% ee; $[\alpha]_D^{22}$ –95.0 (*c* 1.0, $CHCl_3$); ^1H NMR (300 MHz, $CDCl_3$) δ 8.36 (d, *J* = 8.8 Hz, 1H), 8.06 (d, *J* = 8.4 Hz, 1H), 7.72 (d, *J* = 7.2 Hz, 1H), 7.53 – 7.41 (m, 4H), 7.28 – 7.22 (m, 2H), 7.15 (t, *J* = 7.3 Hz, 1H), 3.83 (d, *J* = 10.8 Hz, 0.05H), 2.87 – 2.75 (m, 1H), 0.93 – 0.86 (m, 6H); ^{13}C NMR (75 MHz, $CDCl_3$) δ 164.8, 148.6, 142.5, 135.6, 129.5, 129.4, 129.2, 128.4, 128.4, 126.5, 126.2, 122.5, 121.7, 31.9, 21.7, 21.4; HRMS (ESI) m/z 341.0750 (M+H$^+$), calc. for $C_{19}H_{18}DBrN$ 341.0758. The ee was determined by HPLC analysis: CHIRALPAK IG x 2 (4.6 mm i.d. x 250 mm); hexane/2-propanol = 98/2; flow rate 1.0 mL/min; 25 ºC; 230 nm; retention time: 12.4 min (major) and 14.3 min (minor).

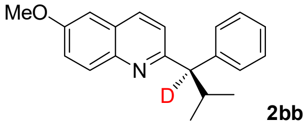

2bb

Colorless oil; 21.9 mg, 75% yield; >95% D, 90% ee; $[\alpha]_D^{22}$ –69.7 (*c* 1.0, $CHCl_3$); ^1H NMR (300 MHz, $CDCl_3$) δ 7.98 (d, *J* = 9.2 Hz, 1H), 7.89 (d, *J* = 8.5 Hz, 1H), 7.48 (d, *J* = 7.3 Hz, 2H), 7.35 – 7.20 (m, 4H), 7.15 (t, *J* = 7.3 Hz, 1H), 6.98 (d, *J* = 2.6 Hz, 1H), 3.88 (s, 3H), 2.87 – 2.65 (m, 1H), 0.93 – 0.86 (m, 6H); ^{13}C NMR (75 MHz, $CDCl_3$) δ 161.5, 157.2, 143.8, 143.2, 135.0, 130.6, 128.4, 128.3, 127.6, 126.2, 121.6 , 121.3, 105.0, 55.5, 32.0, 21.7, 21.4; HRMS (ESI) m/z 293.1752 (M+H$^+$), calc. for $C_{20}H_{21}DNO$ 293.1759. The ee was determined by HPLC analysis: CHIRALPAK ODH x 2 (4.6 mm i.d. x 250 mm); hexane/2-propanol = 98/2; flow rate 1.2 mL/min; 25 ºC; 230 nm; retention time: 6.6 min (minor) and 8.2 min (major).

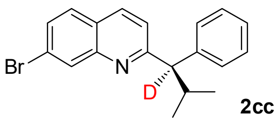

2cc

White solid; Mp 76 – 78 ºC; 21.5 mg, 63% yield; 94% D, 92% ee; $[\alpha]_D^{22}$ –114.1 (*c* 1.0, $CHCl_3$); ^1H NMR (300 MHz, $CDCl_3$) δ 8.23 (s, 1H), 7.88 (d, *J* = 8.5 Hz, 1H), 7.52 – 7.44 (m, 2H), 7.39 (d, *J* = 7.3 Hz, 2H), 7.27 (d, *J* = 8.5 Hz, 1H), 7.20 – 7.15 (m, 2H), 7.11 – 7.05 (m, 1H), 3.70 (d, *J* = 10.4 Hz, 0.06H), 2.79 – 2.70 (m, 1H), 0.84 – 0.80 (m, 6H); ^{13}C NMR (75 MHz, $CDCl_3$) δ 165.0, 148.4, 142.6, 136.0, 131.7, 131.6, 129.3, 128.6, 128.5, 128.4, 126.5, 125.4, 121.8, 31.94, 21.7, 21.3; HRMS (ESI) m/z 341.0751 (M+H$^+$), calc. for $C_{19}H_{18}DBrN$ 341.0758. The ee was determined by HPLC analysis: CHIRALPAK IG x 2 (4.6 mm i.d. x 250

mm); hexane/2-propanol = 98/2; flow rate 1.0 mL/min; 25 °C; 230 nm; retention time: 10.3 min (major) and 11.6 min (minor).

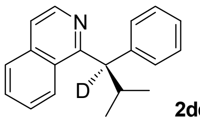

2dd

White solid; Mp 45 – 47 °C; 20.5 mg, 78% yield; 93% D, 82% ee; $[\alpha]_D^{22}$ +27.6 (*c* 1.0, $CHCl_3$); ^1H NMR (300 MHz, $CDCl_3$) δ 8.59 (d, *J* = 5.6 Hz, 1H), 8.36 (d, *J* = 8.1 Hz, 1H), 7.77 (d, *J* = 7.8 Hz, 1H), 7.64 – 7.45 (m, 5H), 7.29 – 7.18 (m, 2H), 7.15 – 7.08 (m, 1H), 4.45 (d, *J* = 10.9 Hz, 0.08H), 3.14 – 2.84 (m, 1H), 0.93 – 0.85 (m, 6H); ^{13}C NMR (75 MHz, $CDCl_3$) δ 162.6, 142.9, 141.8, 136.4, 129.5, 128.6, 128.2, 127.5, 127.5, 127.0, 126.2, 124.8, 119.0, 32.7, 22.0, 21.5; HRMS (ESI) m/z 263.1646 ($M+H^+$), calc. for $C_{19}H_{19}DN$ 263.1653. The ee was determined by HPLC analysis: CHIRALPAK INB + ODH + ODH (4.6 mm i.d. x 250 mm); hexane/2-propanol = 98/2; flow rate 1.0 mL/min; 25 °C; 230 nm; retention time: 16.1 min (major) and 17.1 min (minor).

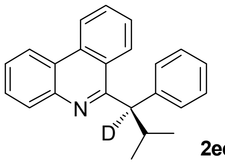

2ee

White solid; Mp 135 – 137 °C; 25.3 mg, 81% yield; 90% D, 83% ee; $[\alpha]_D^{22}$ –380.5 (*c* 1.0, $CHCl_3$); ^1H NMR (300 MHz, $CDCl_3$) δ 8.61 (d, *J* = 8.2 Hz, 1H), 8.53 (d, *J* = 8.1 Hz, 1H), 8.43 (d, *J* = 8.2 Hz, 1H), 8.25 (d, *J* = 8.1 Hz, 1H), 7.81 – 7.70 (m, 2H), 7.67 – 7.54 (m, 4H), 7.28 – 7.19 (m, 2H), 7.12 (t, *J* = 7.3 Hz, 1H), 4.50 (d, *J* = 10.1 Hz, 0.15H), 3.25 – 3.14 (m, 1H), 0.98 (d, *J* = 6.4 Hz, 3H), 0.92 (d, *J* = 6.7 Hz, 3H); ^{13}C NMR (75 MHz, $CDCl_3$) δ 162.0, 143.8, 142.8, 133.0, 130.2, 129.8, 128.8, 128.3, 128.1, 127.1, 126.3, 126.2, 125.9, 125.7, 123.3, 122.4, 121.8, 56.7, 32.6, 22.2, 21.4; HRMS (ESI) m/z 313.1801 ($M+H^+$), calc. for $C_{23}H_{21}DN$ 313.1810. The ee was determined by HPLC analysis: CHIRALPAK ODH (4.6 mm i.d. x 250 mm); hexane/2-propanol = 98/2; flow rate 1.0 mL/min; 25 °C; 230 nm; retention time: 6.2 min (minor) and 11.7 min (major).

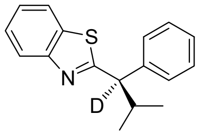

2ff

White solid; Mp 62 – 64 °C; 19.1 mg, 71% yield; 93% D, 80% ee; $[\alpha]_D^{22}$ –47.0 (*c* 1.0, $CHCl_3$); ^1H NMR (300 MHz, $CDCl_3$) δ 7.93 (d, *J* = 8.1 Hz, 1H), 7.72 (d, *J* = 7.8 Hz, 1H), 7.41 – 7.31

(m, 3H), 7.28 – 7.07 (m, 4H), 3.92 (d, J = 10.5 Hz, 0.07H), 2.68 – 2.54 (m, 1H), 0.97 (d, J = 6.5 Hz, 3H), 0.82 (d, J = 6.6 Hz, 3H); ^{13}C NMR (75 MHz, $CDCl_3$) δ 174.5, 153.0, 141.0, 135.0, 128.6, 128.5, 127.1, 125.8, 124.6, 122.8, 121.4, 33.6, 21.6, 21.2; HRMS (ESI) m/z 269.1211 (M+H$^+$), calc. for $C_{17}H_{17}DNS$ 269.1217. The ee was determined by HPLC analysis: CHIRALPAK IG (4.6 mm i.d. x 250 mm); hexane/2-propanol = 80/20; flow rate 1.0 mL/min; 25 °C; 230 nm; retention time: 5.6 min (major) and 11.3 min (minor).

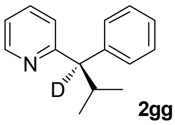

2gg

Colorless oil; 13.8 mg, 65% yield; 85% D, 53% ee; ^{1}H NMR (300 MHz, $CDCl_3$) δ 8.48 (d, J = 4.3 Hz, 1H), 7.46 (m, 7.51 – 7.40, 1H), 7.38 – 7.30 (m, 2H), 7.21 – 7.03 (m, 4H), 7.01 – 6.95 (m, 1H), 3.49 (d, J = 10.8 Hz, 0.15H), 2.62 – 2.57 (m, 1H), 0.78 (d, J = 6.5 Hz, 1H); ^{13}C NMR (75 MHz, $CDCl_3$) δ 163.7, 149.2, 143.4, 143.4, 136.2, 128.3, 126.2, 123.1, 123.1, 121.1, 62.7, 62.5, 62.2, 32.2, 32.1, 21.7, 21.4. The ee was determined by HPLC analysis: CHIRALPAK ODH x 3 (4.6 mm i.d. x 250 mm); hexane/2-propanol = 96/4; flow rate 0.7 mL/min; 25 °C; 230 nm; retention time: 23.5 min (major) and 24.5 min (minor).

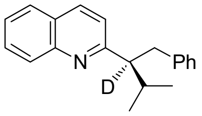

2hh

Colorless oil; 13.8 mg, 65% yield; >95% D, 38% ee; ^{1}H NMR (300 MHz, $CDCl_3$) δ 8.07 (d, J = 8.4 Hz, 1H), 7.92 (d, J = 8.4 Hz, 1H), 7.79 – 7.60 (m, 2H), 7.46 (t, J = 7.5 Hz, 1H), 7.12 – 6.89 (m, 6H), 4.24 – 4.21 (M, 0.03H), 3.23 (s, 2H), 2.31 – 2.05 (m, 1H), 1.12 (d, J = 6.7 Hz, 3H), 0.85 (d, J = 6.7 Hz, 3H); ^{13}C NMR (75 MHz, $CDCl_3$) δ 164.1, 147.8, 141.1, 135.2, 129.2, 129.0, 129.0, 127.9, 127.4, 126.8, 125.5, 125.5, 122.3, 38.1, 32.6, 20.8, 20.8. The ee was determined by HPLC analysis: Lux 5u Cellulose-3 & Chiral MJ (2) (4.6 mm i.d. x 250 mm); hexane/2-propanol = 98/2; flow rate 1.0 mL/min; 25 °C; 254 nm; retention time: 10.0 min (major) and 10.9 min (minor).

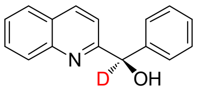

5a: white solid, Mp 50 – 52 °C, 22.7 mg, 96% yield, 91% D, 91% ee, $[\alpha]_D^{22}$ –88.6 (c 1.0, $CHCl_3$); ^{1}H NMR (300 MHz, $CDCl_3$) δ 8.18 (d, J = 8.4 Hz, 1H), 8.08 (d, J = 8.5 Hz, 1H), 7.78 (dd, J = 16.1, 8.0 Hz, 2H), 7.57 (t, J = 7.5 Hz, 1H), 7.42 (d, J = 6.9 Hz, 2H), 7.33 (dd, J = 16.2, 8.7 Hz, 3H), 7.20 (d, J = 8.5 Hz, 1H), 6.09 (s, 1H), 5.90 (s, 0.09H); ^{13}C NMR (75

MHz, $CDCl_3$) δ 160.4, 145.9, 142.7, 137.0, 129.9, 128.8, 128.6, 128.0, 127.6, 127.4, 126.6, 119.2, 75.1; HRMS (ESI) m/z 237.1131 ($M+H^+$), calc. for $C_{16}H_{13}DNO$ 237.1133. The ee was determined by HPLC analysis: CHIRALPAK INB (4.6 mm i.d. x 250 mm); Hexane/2-propanol = 90/10; flow rate 1.0 mL/min; 25 ºC; 230 nm; retention time: 10.9 min (minor) and 22.0 min (major).

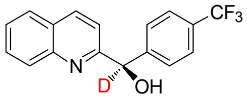

5b: white solid, Mp 120 – 122 ºC, 24.9 mg, 82% yield, 91% D, 94% ee, $[\alpha]_D^{22}$ −143.8 (*c* 1.0, $CHCl_3$); ^1H NMR (300 MHz, $CDCl_3$) δ 8.21 (d, *J* = 8.6 Hz, 1H), 8.15 (d, *J* = 8.5 Hz, 1H), 7.82 (dd, *J* = 16.6, 8.0 Hz, 2H), 7.60 (m, 5H), 7.23 (s, 1H), 6.17 (s, 1H), 6.03 (s, 0.09 H); ^{13}C NMR (75 MHz, $CDCl_3$) δ 159.4, 146.7, 146.0, 137.4, 130.1, 130.1 (q, *J* = 32.4 Hz), 128.7, 127.6, 127.6, 127.5, 126.9, 125.6 (q, *J* = 3.8 Hz), 124.0 (q, *J* = 272.1 Hz), 118.9, 74.6; ^{19}F NMR (376 MHz, $CDCl_3$) δ -63.07; HRMS (ESI) m/z 305.0997 ($M+H^+$), calc. for $C_{17}H_{12}DF_3NO$ 305.1007. The ee was determined by HPLC analysis: CHIRALPAK INB (4.6 mm i.d. x 250 mm); Hexane/2-propanol = 90/10; flow rate 1.0 mL/min; 25 ºC; 230 nm; retention time: 9.0 min (minor) and 15.3 min (major).

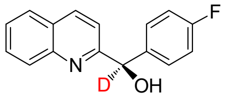

5c: white solid, Mp 74 – 75 ºC, 24.1 mg, 95% yield, 90% D, 92% ee, $[\alpha]_D^{22}$ −27.5 (*c* 1.0, $CHCl_3$); ^1H NMR (300 MHz, $CDCl_3$) δ 8.21 (dd, *J* = 22.8, 8.5 Hz, 2H), 7.83 (dd, *J* = 18.7, 7.9 Hz, 2H), 7.62 (t, *J* = 7.6 Hz, 1H), 7.45 (dd, *J* = 8.4, 5.5 Hz, 2H), 7.28 (s, 1H), 7.05 (t, *J* = 8.6 Hz, 2H), 6.15 (s, 1H), 6.01 (s, 0.1 H); ^{19}F NMR (376 MHz, $CDCl_3$) δ -105.11; ^{13}C NMR (75 MHz, $CDCl_3$) δ 162.3 (d, *J* = 246.1 Hz), 160.3, 145.8, 138.5 (d, *J* = 3.1 Hz), 137.0, 129.8, 128.9 (d, *J* = 8.2 Hz), 128.6, 127.5, 127.3, 126.6, 118.9, 115.3 (d, *J* = 21.5 Hz), 74.4; HRMS (ESI) m/z 255.1032 ($M+H^+$), calc. for $C_{16}H_{12}DFNO$ 255.1038. The ee was determined by HPLC analysis: CHIRALPAK OZ–H (4.6 mm i.d. x 250 mm); Hexane/2-propanol = 94/6; flow rate 1.0 mL/min; 25 ºC; 254 nm; retention time: 11.3 min (minor) and 14.7 min (major).

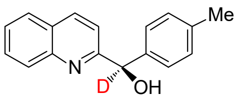

5d: white solid, Mp 67 – 69 °C, 24.8 mg, 99% yield, 91% D, 97% ee, $[\alpha]_D^{22}$ −156.9 (*c* 1.0, $CHCl_3$); ^1H NMR (300 MHz, $CDCl_3$) δ 8.16 (d, *J* = 8.4 Hz, 1H), 8.06 (d, *J* = 8.5 Hz, 1H), 7.77 (dd, *J* = 16.3, 7.9 Hz, 2H), 7.56 (t, *J* = 7.4 Hz, 1H), 7.29 (d, *J* = 7.9 Hz, 2H), 7.17 (dd, *J* = 12.2, 8.2 Hz, 3H), 6.02 (s, 1H), 5.86 (s, 0.09H), 2.33 (s, 3H); ^{13}C NMR (75 MHz, $CDCl_3$) δ 160.4, 145.9, 142.6, 138.3, 137.0, 129.9, 128.8, 128.5, 128.0, 127.6, 126.6, 124.6, 119.3, 75.1, 21.4; HRMS (ESI) m/z 251.1293 ($M+H^+$), calc. for $C_{17}H_{15}DNO$ 251.1289. The ee was determined by HPLC analysis: CHIRALPAK INB (4.6 mm i.d. x 250 mm); Hexane/2-propanol = 94/6; flow rate 1.0 mL/min; 25 °C; 254 nm; retention time: 15.1 min (minor) and 30.2 min (major).

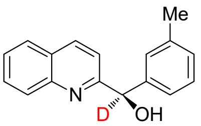

5e: white solid, Mp 79 – 81 °C, 22.8 mg, 91% yield, 90% D, 99% ee, $[\alpha]_D^{22}$ −76.1 (*c* 1.0, $CHCl_3$); ^1H NMR (300 MHz, $CDCl_3$) δ 8.17 (d, *J* = 8.4 Hz, 1H), 8.07 (d, *J* = 8.5 Hz, 1H), 7.78 (dd, *J* = 16.5, 7.9 Hz, 2H), 7.57 (t, *J* = 7.5 Hz, 1H), 7.21 (m, 4H), 7.10 (d, *J* = 6.2 Hz, 1H), 6.07 (s, 1H), 5.86 (s, 0.1 H), 2.32 (s, 3H); ^{13}C NMR (75 MHz, $CDCl_3$) δ 160.6, 145.8, 139.8, 137.7, 137.0, 129.9, 129.3, 128.7, 127.6, 127.4, 127.4, 126.6, 119.3, 74.9, 21.1; HRMS (ESI) m/z 251.1284 ($M+H^+$), calc. for $C_{17}H_{15}DNO$ 251.1289. The ee was determined by HPLC analysis: CHIRALPAK OZ–H (4.6 mm i.d. x 250 mm); Hexane/2-propanol = 94/6; flow rate 1.0 mL/min; 25 °C; 254 nm; retention time: 15.4 min (minor) and 20.4 min (major).

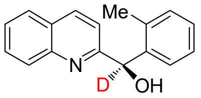

5f: white solid, Mp 82 – 84 °C, 20.7 mg, 83% yield, 93% D, 97% ee, $[\alpha]_D^{22}$ −171.8 (*c* 1.0, $CHCl_3$); ^1H NMR (300 MHz, $CDCl_3$) δ 8.17 (d, *J* = 8.5 Hz, 1H), 8.06 (d, *J* = 8.5 Hz, 1H), 7.79 (dd, *J* = 17.4, 7.9 Hz, 2H), 7.57 (t, *J* = 7.6 Hz, 1H), 7.17 (m, 4H), 7.08 (d, *J* = 8.5 Hz, 1H), 6.11 (s, 0.07 H), 5.92 (s, 1H), 2.40 (s, 3H); ^{13}C NMR (75 MHz, $CDCl_3$) δ 160.4 (d, *J* = 246.2 Hz), 159.7, 145.9, 137.4, 130.0, 129.8, δ 129.4 (d, *J* = 8.3 Hz), 128.8 (d, *J* = 4.0 Hz),

128.7, 127.6, 127.6, 126.7, 124.5 (d, J = 3.5 Hz), 118.9 (d, J = 3.1 Hz), 115.5 (d, J = 21.9 Hz), 68.1 (d, J = 4.0 Hz); HRMS (ESI) m/z 251.1288 (M+H$^+$), calc. for $C_{17}H_{15}DNO$ 251.1289. The ee was determined by HPLC analysis: CHIRALPAK OZ–H (4.6 mm i.d. x 250 mm); Hexane/2-propanol = 94/6; flow rate 1.0 mL/min; 25 °C; 254 nm; retention time: 11.6 min (minor) and 12.9 min (major).

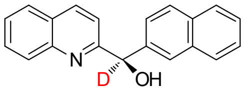

5g: white solid, Mp 132 – 133 °C, 24.6 mg, 86% yield, 91% D, 90% ee, $[\alpha]_D^{22}$ −91.3 (c 1.0, $CHCl_3$); ^1H NMR (300 MHz, $CDCl_3$) δ 8.20 (d, J = 8.5 Hz, 1H), 8.05 (d, J = 8.5 Hz, 1H), 7.96 (s, 1H), 7.81 (m, 5H), 7.57 (t, J = 7.4 Hz, 1H), 7.47 (dt, J = 12.3, 8.0 Hz, 3H), 7.22 (d, J = 8.5 Hz, 1H), 6.19 (s, 1H), 6.06 (s, 0.09H); ^{13}C NMR (75 MHz, $CDCl_3$) δ 160.3, 146.0, 140.1, 137.0, 133.3, 133.2, 130.0, 128.8, 128.6, 128.0, 127.7, 127.6, 127.5, 126.7, 126.7, 126.2, 126.0, 125.0, 119.3, 75.3; HRMS (ESI) m/z 287.1286 (M+H$^+$), calc. for $C_{20}H_{15}DNO$ 287.1289. The ee was determined by HPLC analysis: CHIRALPAK INB (4.6 mm i.d. x 250 mm); Hexane/2-propanol = 94/6; flow rate 1.0 mL/min; 25 °C; 254 nm; retention time: 12.2 min (minor) and 15.2 min (major).

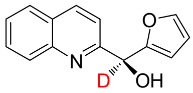

5h: white solid, Mp 120 – 122 °C, 16.3 mg, 72% yield, >95% D, 97% ee, $[\alpha]_D^{22}$ +78.8 (c 1.0, $CHCl_3$); ^1H NMR (300 MHz, $CDCl_3$) δ 8.15 (d, J = 8.4 Hz, 2H), 7.84 (d, J = 8.0 Hz, 1H), 7.76 (t, J = 7.6 Hz, 1H), 7.58 (t, J = 7.5 Hz, 1H), 7.35 (m, 2H), 6.34 (s, 2H), 5.96 (s, 0.01H), 5.81 (s, 1H); ^{13}C NMR (75 MHz, $CDCl_3$) δ 157.6, 154.7, 146.1, 142.8, 137.2, 130.0, 128.8, 127.7, 127.6, 126.8, 118.9, 110.3, 108.0, 68.6; HRMS (ESI) m/z 227.0927 (M+H$^+$), calc. for $C_{14}H_{11}DNO_2$ 227.0926. The ee was determined by HPLC analysis: CHIRALPAK INB (4.6 mm i.d. x 250 mm); Hexane/2-propanol = 90/10; flow rate 1.0 mL/min; 25 °C; 254 nm; retention time: 9.9 min (minor) and 16.5 min (major).

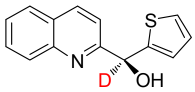

5i: white solid, Mp 118 – 119 °C, 24.1 mg, 99% yield, 95% D, 96% ee, $[\alpha]_D^{22}$ −121.3 (*c* 1.0, $CHCl_3$); ^1H NMR (300 MHz, $CDCl_3$) δ 8.14 (dd, *J* = 8.3, 3.7 Hz, 2H), 7.83 (d, *J* = 8.1 Hz, 1H), 7.77 (t, *J* = 7.6 Hz, 1H), 7.57 (t, *J* = 7.4 Hz, 1H), 7.33 (d, *J* = 8.5 Hz, 1H), 7.27 (d, *J* = 5.0 Hz, 1H), 7.12 (d, *J* = 2.6 Hz, 1H), 6.98 (m, 1H), 6.17 (s, 0.05H), 6.02 (s, 1H); ^{13}C NMR (75 MHz, $CDCl_3$) δ 159.5, 146.7, 145.8, 137.4, 130.1, 128.7, 127.6, 127.6, 126.9, 126.6, 125.9, 125.6, 119.0, 70.8; HRMS (ESI) m/z 243.0695 ($M+H^+$), calc. for $C_{14}H_{11}DNOS$ 243.0697. The ee was determined by HPLC analysis: CHIRALPAK INB (4.6 mm i.d. x 250 mm); Hexane/2-propanol = 90/10; flow rate 1.0 mL/min; 25 °C; 210 nm; retention time: 12.2 min (minor) and 21.7 min (major).

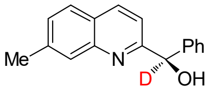

5j: white solid, Mp 120 – 122 °C, 20.5 mg, 82% yield, 92% D, 94% ee, $[\alpha]_D^{22}$ −173.8 (*c* 1.0, $CHCl_3$); ^1H NMR (300 MHz, $CDCl_3$) δ 7.99 (m, 2H), 7.69 (d, *J* = 8.3 Hz, 1H), 7.34 (m, 2H), 7.12 (d, *J* = 8.4 Hz, 1H), 6.11 (s, 0.08H), 5.87 (s, 1H), 2.57 (d, *J* = 13.3 Hz, 3H); ^{13}C NMR (75 MHz, $CDCl_3$) δ 160.9, 144.4, 142.4, 136.1, 132.4, 130.8, 130.4, 128.7, 128.1, 128.1, 127.4, 126.3, 120.2, 75.2; HRMS (ESI) m/z 251.1291 ($M+H^+$), calc. for $C_{17}H_{15}DNO$ 251.1289. The ee was determined by HPLC analysis: CHIRALPAK INB (4.6 mm i.d. x 250 mm); Hexane/2-propanol = 85/15; flow rate 1.0 mL/min; 25 °C; 230 nm; retention time: 13.8 min (minor) and 18.2 min (major).

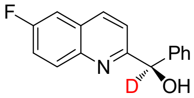

5k: white solid, Mp 126 – 127 °C, 16.5 mg, 65% yield, 92% D, 90% ee, $[\alpha]_D^{22}$ −113.3 (*c* 1.0, $CHCl_3$); ^1H NMR (300 MHz, $CDCl_3$) δ 8.16 (dd, *J* = 9.2, 5.3 Hz, 1H), 8.02 (d, *J* = 8.6 Hz, 1H), 7.53 (m, 1H), 7.37 (m, 6H), 7.22 (d, *J* = 8.6 Hz, 1H), 5.88 (s, 1H), 5.88 (s, 0.08 H); ^{19}F NMR (376 MHz, $CDCl_3$) δ -112.87. ^{13}C NMR (75 MHz, $CDCl_3$) δ 159.4, 146.7, 146.0, 137.4, 130.1, 130.1 (q, *J* = 32.4 Hz), 128.7, 127.6, 127.6, 127.5, 126.9, 125.6 (q, *J* = 3.8 Hz), 124.0

(q, J = 272.1 Hz), 118.9, 74.6; ^{19}F NMR (376 MHz, $CDCl_3$) δ -112.87; HRMS (ESI) m/z 255.1035 ($M+H^+$), calc. for $C_{16}H_{12}DFNO$ 255.1038. The ee was determined by HPLC analysis: CHIRALPAK INB (4.6 mm i.d. x 250 mm); Hexane/2-propanol = 90/10; flow rate 1.0 mL/min; 25 ºC; 254 nm; retention time: 9.4 min (minor) and 11.6 min (major).

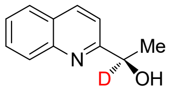

5l: yellow solid, Mp 84 – 85 ºC, 15.8 mg, 91% yield, > 95% D, 80% ee, $[\alpha]_D^{22}$ +18.1 (c 1.0, $CHCl_3$); ^{1}H NMR (300 MHz, $CDCl_3$) δ 8.17 (d, J = 8.5 Hz, 1H), 8.09 (d, J = 8.4 Hz, 1H), 7.83 (d, J = 8.1 Hz, 1H), 7.74 (t, J = 7.6 Hz, 1H), 7.55 (t, J = 7.5 Hz, 1H), 7.36 (d, J = 8.5 Hz, 1H), 5.03 (s, 1H), 1.58 (s, 3H); ^{13}C NMR (75 MHz, $CDCl_3$) δ 162.8, 146.3, 137.0, 129.8, 128.7, 127.6, 127.4, 126.4, 118.0, 68.7, 24.1; HRMS (ESI) m/z 175.0980 ($M+H^+$), calc. for $C_{11}H_{11}DNO$ 175.0976. The ee was determined by HPLC analysis: CHIRAL INB (4.6 mm i.d. x 250 mm); Hexane/2-propanol = 90/10; flow rate 1.0 mL/min; 25 ºC; 230 nm; retention time: 7.4 min (minor) and 12.3 min (major).

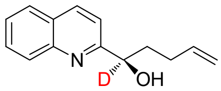

5m: white solid, Mp 120 – 122 ºC, 21.2 mg, 99% yield, >95% D, 88% ee, $[\alpha]_D^{22}$ −26.3 (c 1.0, $CHCl_3$); ^{1}H NMR (300 MHz, $CDCl_3$) δ 8.16 (d, J = 8.5 Hz, 1H), 8.08 (d, J = 8.4 Hz, 1H), 7.83 (d, J = 8.0 Hz, 1H), 7.73 (t, J = 7.6 Hz, 1H), 7.55 (t, J = 7.5 Hz, 1H), 7.34 (d, J = 8.5 Hz, 1H), 5.87 (ddt, J = 16.9, 10.2, 6.6 Hz, 1H), 4.99 (m, 3H), 2.26 (m, 2H), 2.04 (ddd, J = 15.9, 9.6, 6.4 Hz, 1H), 1.81 (m, 1H); ^{13}C NMR (75 MHz, $CDCl_3$) δ 161.9, 146.3, 138.2, 137.0, 129.8, 128.6, 127.6, 127.4, 126.4, 118.3, 114.8, 72.0, 37.4, 29.4; HRMS (ESI) m/z 214.1291 ($M+H^+$), calc. for $C_{14}H_{15}DNO$ 215.1289. The ee was determined by HPLC analysis: CHIRALPAK INB (4.6 mm i.d. x 250 mm); Hexane/2-propanol = 90/10; flow rate 1.0 mL/min; 25 ºC; 210 nm; retention time: 6.3 min (minor) and 9.2 min (major).

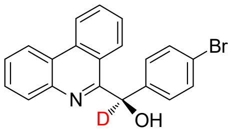

5n: white solid, Mp 160 – 161 °C, 35.9 mg, 99% yield, 93% D, 88% ee, $[\alpha]_D^{22}$ −118.9 (*c* 1.0, $CHCl_3$); ^{1}H NMR (300 MHz, $CDCl_3$) δ 8.55 (dd, *J* = 16.9, 8.2 Hz, 2H), 8.19 (d, *J* = 7.9 Hz, 1H), 7.89 (d, *J* = 8.3 Hz, 1H), 7.74 (t, *J* = 7.6 Hz, 2H), 7.66 (t, *J* = 7.5 Hz, 1H), 7.50 (t, *J* = 7.7 Hz, 1H), 7.34 (d, *J* = 8.3 Hz, 2H), 7.19 (m, 2H), 6.47 (s, 1H), 6.29 (s, 0.07H); ^{13}C NMR (75 MHz, $CDCl_3$) δ 158.4, 141.9, 141.6, 133.5, 131.9, 131.0, 129.5, 129.5, 129.1, 127.5, 127.5, 125.8, 124.4, 123.2, 122.7, 122.2, 122.0, 72.0; HRMS (ESI) m/z 386.0392 ($M+H^+$), calc. for $C_{20}H_{14}DBrNO$ 365.0395. The ee was determined by HPLC analysis: CHIRALPAK IE (4.6 mm i.d. x 250 mm); Hexane/2-propanol = 90/10; flow rate 1.0 mL/min; 25 °C; 210 nm; retention time: 24.4 min (major) and 38.1 min (minor).

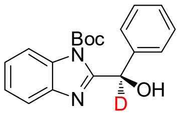

5o: white solid, Mp 120 – 122 °C, 22.0 mg, 83% yield, >95% D, 95% ee, $[\alpha]_D^{22}$ +23.4 (*c* 1.0, $CHCl_3$); ^{1}H NMR (300 MHz, $CDCl_3$) δ 7.88 (m, 1H), 7.75 (dd, *J* = 5.5, 3.2 Hz, 1H), 7.33 (ddd, *J* = 15.3, 7.4, 4.7 Hz, 7H), 7.06 (s, 0.04H), 4.69 (s, 1H), 1.54 (s, 9H); ^{13}C NMR (75 MHz, $CDCl_3$) δ 152.1, 151.5, 136.7, 128.9, 128.8, 127.0, 122.9, 83.6, 74.6, 27.6; HRMS (ESI) m/z 326.1612 ($M+H^+$), calc. for $C_{19}H_{20}DN_2O_3$ 326.1610. The ee was determined by HPLC analysis: cell-4 (4.6 mm i.d. x 250 mm); Hexane/2-propanol = 60/40; flow rate 1.0 mL/min; 25 °C; 254 nm; retention time: 6.1 min (minor) and 10.5 min (major).

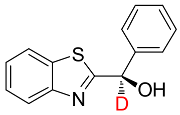

5p: yellow oil, 22.5 mg, 93% yield, 91% D, 89% ee, $[\alpha]_D^{22}$ −13.1 (*c* 1.0, $CHCl_3$); ^{1}H NMR (300 MHz, $CDCl_3$) δ 7.99 (d, *J* = 8.1 Hz, 1H), 7.84 (d, *J* = 7.8 Hz, 1H), 7.44 (m, 7H), 6.15 (s, 0.09H), 3.66 (s, 1H); ^{13}C NMR (75 MHz, $CDCl_3$) δ 175.0, 152.4, 140.9, 135.2, 128.8, 128.7, 126.7, 126.2, 125.2, 123.0, 121.8, 74.4; HRMS (ESI) m/z 243.0688 ($M+H^+$), calc. for $C_{14}H_{15}DNOS$ 243.0697.

The ee was determined by HPLC analysis: CHIRAL INB (4.6 mm i.d. x 250 mm); Hexane/2-propanol = 80/20; flow rate 1.0 mL/min; 25 ºC; 230 nm; retention time: 6.2 min (minor) and 9.2 min (major).

Supplemental References:

Olomanov, O. V., Bourhis, L. J., Gildea, R. J., Howard, J. A., and Puschmann, H. (2009). OLEX2: a complete structure solution, refinement and analysis program. J. Appl. Cryst. *42*, 339–341.

Sheldrick, G. M. (2008). A short history of SHELX. Acta Cryst. A: Foundations of Crystallography *64*, 112–122.

Heldrick, G. M. (2015). Crystal structure refinement with SHELXL. Acta Cryst. C: Structural Chemistry *71*, 3–8.

Frisch, M., Trucks, G., Schlegel, H., Scuseria, G., Robb, M., Cheeseman, J., Scalmani, G., Barone, V., Mennucci, B., and Petersson, G. (1993). Gaussian 09, Revision D. 01, Gaussian, Inc., Wallingford CT, 2013 Search PubMed; (b) AD Becke. J Chem Phys *98*, 785–789.

Terrasson, V., Planas, J. G., Prim, D., Vinas, C., Teixidor, F., Light, M. E., and Hursthouse, M. B. (2008). Cooperative Effect of Carborane and Pyridine in the Reaction of Carboranyl Alcohols with Thionyl Chloride: Halogenation versus Oxidation. J. Org. Chem. *73*, 9140–9143.
